# Label-free cell segmentation of diverse lymphoid tissues in 2D and 3D

**DOI:** 10.1016/j.crmeth.2023.100398

**Published:** 2023-02-02

**Authors:** John W. Wills, Jack Robertson, Pani Tourlomousis, Clare M.C. Gillis, Claire M. Barnes, Michelle Miniter, Rachel E. Hewitt, Clare E. Bryant, Huw D. Summers, Jonathan J. Powell, Paul Rees

**Affiliations:** 1 Department of Veterinary Medicine, Cambridge University, Madingley Road, Cambridge CB3 0ES, UK; 2 Department of Biomedical Engineering, Swansea University, Fabian Way, Crymlyn Burrows, Swansea SA1 8EN, Wales, UK; 3 Imaging Platform, Broad Institute of MIT and Harvard, 415 Main Street, Boston, Cambridge, MA 02142, USA

**Keywords:** label free, cell segmentation, tissue, confocal microscopy, immunofluorescence, single-cell, quantitative, 2D, 3D, digital pathology

## Abstract

Unlocking and quantifying fundamental biological processes through tissue microscopy requires accurate, *in situ* segmentation of all cells imaged. Currently, achieving this is complex and requires exogenous fluorescent labels that occupy significant spectral bandwidth, increasing the duration and complexity of imaging experiments while limiting the number of channels remaining to address the study’s objectives. We demonstrate that the excitation light reflected during routine confocal microscopy contains sufficient information to achieve accurate, label-free cell segmentation in 2D and 3D. This is achieved using a simple convolutional neural network trained to predict the probability that reflected light pixels belong to either nucleus, cytoskeleton, or background classifications. We demonstrate the approach across diverse lymphoid tissues and provide video tutorials demonstrating deployment in Python and MATLAB or via standalone software for Windows.

## Highlights

• Cell segmentation of tissues can be achieved from reflected laser excitation light
• Single-cell information is freely established for many tissue microscopy studies
• Windows software is provided alongside extensive video tutorials and data

## Motivation

Across the biomedical sciences, there is an urgent need to move beyond qualitative imaging to quantitative, cell-based reporting of tissue microscopy data. Typically, cell segmentation requires fluorescent labeling of nucleus and cytoplasm, which limits the spectral bandwidth available for other reporter molecules. However, recent advances in deep-learning algorithms have transformed automated image classification, and this raises the possibility of proceeding with reduced image information. Here, we show that 2D and 3D cell segmentation of lymphoid tissues can be freely established from the reflected laser excitation light always present during routine confocal microscopy using entirely standard equipment.

## Graphical abstract

**Figure:**
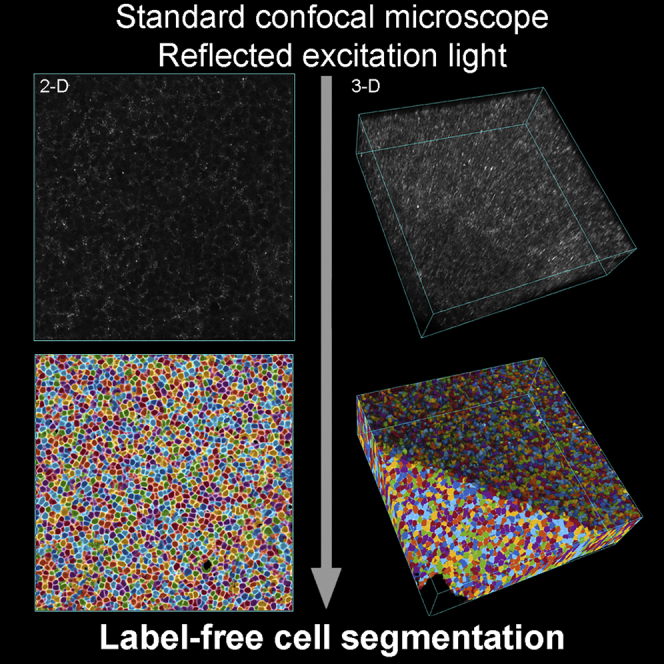

## Teaser

Cell segmentation is an essential step in quantitative tissue microscopy. Wills et al. show this can be achieved simply using the reflected laser light always present during routine imaging by confocal microscopy. This frees up microscope channels and establishes single-cell information as an attainable start point for many tissue microscopy experiments.

## Introduction

The analysis of tissues using fluorescence labeling and confocal microscopy represents a mainstay biomedical technique that is used worldwide to understand the biology of cells *in situ*.^1^^,^^2^^,^^3^^,^^4^^,^^5^ However, despite the accessibility of confocal microscopy and its ability to provide sensitive, quantifiable data with subcellular resolution in both 2D and 3D, the number of channels that can be successfully imaged is often limited in practice.^2^^,^^6^ Moving beyond qualitative observations to cell-based quantifications for every cell in a tissue specimen requires fluorescent staining (e.g., nuclei, cell membrane, or cluster of differentiation [CD] markers) to enable cell segmentation.^2^^,^^3^^,^^4^^,^^5^ However, these stains occupy channels that are often needed to fully address the bioclinical question.^2^^,^^3^^,^^5^ At the same time, as the number of fluorescence channels increases, so does the complexity, time requirement, and potential for channel cross-talk.^5^^,^^7^ Correcting this complicates analysis and downstream data processing, increasing the expertise required and the risk of error.^3^^,^^5^^,^^7^^,^^8^

Recognizing these complexities in addition to the need to avoid phototoxicity and temporal errors during live-cell experiments with monolayer cells *in vitro*, microscopy techniques that harness endogenous contrast (i.e., label free) have been developed (e.g., phase/differential interference contrast, etc.).^8^^,^^9^^,^^10^^,^^11^ However, a recognized difficulty for subsequent, cell-based image analysis is that label-free cell segmentation accuracy decreases as cultures become confluent and cell-to-cell contact is established.^9^^,^^10^ In this regard, tissue environments are inherently complex and challenging as they are almost entirely comprised of contacting cells in 3D, layer-upon-layer arrangements.^2^^,^^3^^,^^5^

An often overlooked capability of nearly all laser scanning confocal microscopes is the ability to capture reflected laser excitation light. Importantly, unlike transmitted light, this label-free signal is filtered by the confocal aperture enabling capture as 3D “z stacks” of optically isolated sections that can be simultaneously acquired alongside fluorescence information.^7^^,^^8^ Using diverse lymphoid tissues as an exemplar, here we demonstrate how this signal can be harnessed to provide accurate, label-free cell segmentation in 2D and 3D. We show the approach can be deployed in conjunction with the user-friendly, open-source CellProfiler software,^12^ enabling single-cell data extraction for image-based cell profiling in addition to reproducible workflow dissemination. The approach provides every cell and nearest-cell neighbor relationship *in situ* with high precision, leaving a spectrally unencumbered landscape for subsequent interrogation. To support uptake, we provide extensive video tutorials and data, demonstrating deployment using Python, MATLAB, or standalone software for Windows.

## Results

**Figure 1:**
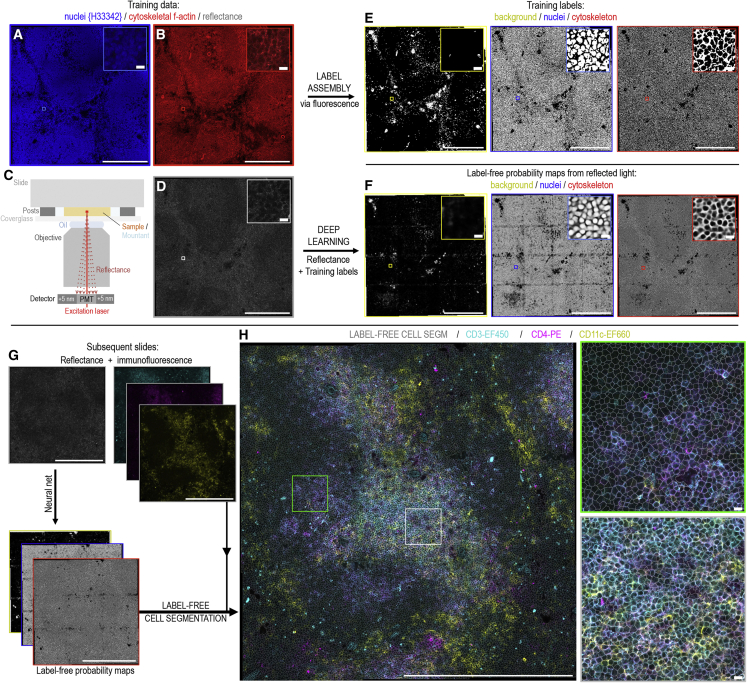
Label-free cell segmentation of tissue microscopy image data collected by routine confocal microscopy (A–D) Image data (here, mouse splenic tissue) for initial network training are obtained from serial tissue sections stained for (A) nuclei (Hoechst 33342) and (B) cytoskeletal f-actin (phalloidin-AlexaFluor 647) while simultaneously collecting (C and D) reflected laser excitation light by detector placement close (± 5 nm) to the excitation wavelength. (E) Binary pixel-classification labels representing “background,” “nuclei,” and “cytoskeleton” classes are created by thresholding the fluorescence data. (F) A neural network using a simple U-Net architecture is trained to output the probability that pixels in the reflectance image belong to each of these classes. (A)–(F) show zoomed insets of the exact same image region. Comparing across these insets, the outputted probability maps (F) exhibit consistent intensities across each image field, with clear gradients that flow between the individual classifications. This enables easy, consistent instance segmentations of individual cell objects using routine watershed approaches. (G and H) For subsequent slides, nuclei and actin stains are no longer required as the cell segmentation is achieved direct from the reflectance information via the probability map images. This establishes the cell segmentation while leaving the entire detection spectrum free for fluorescence-based analyses. For example, (H) shows the approach operating with CD3-eFluor450, CD4-PE, and CD11c-eFluor660 immunofluorescence conjugates utilizing the spectral bandwidth previously occupied by the nuclei (Hoechst 33342) and actin (phalloidin-AlexaFluor 647) stains. The label-free cell segmentation is overlaid. (H) Insets demonstrate successful label-free cell segmentation of both CD marker-stained and entirely unstained cells in both red (green inset) and white (gray inset) pulp tissue regions. (A–H) Main image scale bars: 250 μm, and inset image scale bars: 10 μm.

Figure 1 shows our strategy using mouse splenic tissue. Training data, exemplifying the cellular structure of the tissue, are collected from parallel tissue sections using simple, antibody-independent fluorescent staining for cell nuclei and cytoskeletal F-actin (Figures 1A and 1B). During imaging, both fluorescence data and the backscattered reflectance signal from one of the excitation lasers are captured (Figures 1C and 1D; reflectance imaging setup shown in Method S1; laser invariance demonstrated in Figure S1). From these training data, ground-truth pixel-classification labels representing “background,” “nuclei,” and “cytoskeleton” classes are easily assembled by binary thresholding the training slide’s fluorescence information (Figure 1E). These labels are then used to train a simple U-Net neural network^13^ (Method S1; Figure S2) to output the probability that pixels in the reflectance image belong to each of the classifications (demonstrated using MATLAB, Python, or standalone Windows software; Method S1). Because of pixel-wise averaging of any error in the binary representations of staining used as the ground truth, the probability maps outputted by the network exhibit smooth intensity gradients that flow between classifications (Figure 1F). The nature of these images enables them to serve directly as inputs for segmentation of individual cell objects (Figures 1G and 1H; Video S1). This process can be achieved using the user-friendly CellProfiler software,^12^ providing a flexible and accessible route to bioimage analysis, feature extraction, and reproducible workflow dissemination. After training, subsequent experimental samples only require the reflectance image to obtain the cell segmentation, leaving the fluorescence spectrum entirely available (Figure S3) for any form of spectral interrogation or combination of fluorescent markers (Figure 1H).

Video S1. Training the 2D U-Net network to achieve label-free cell segmentation using reflectance input data, related to Figure 1 Left, nuclei and actin fluorescence staining. Middle, reflectance input data as seen by the network. Right, label-free predictions and subsequent cell-instance segmentations. The video displays network predictions across 50 epochs of training progress. Unseen data is used as input.

**Figure 2:**
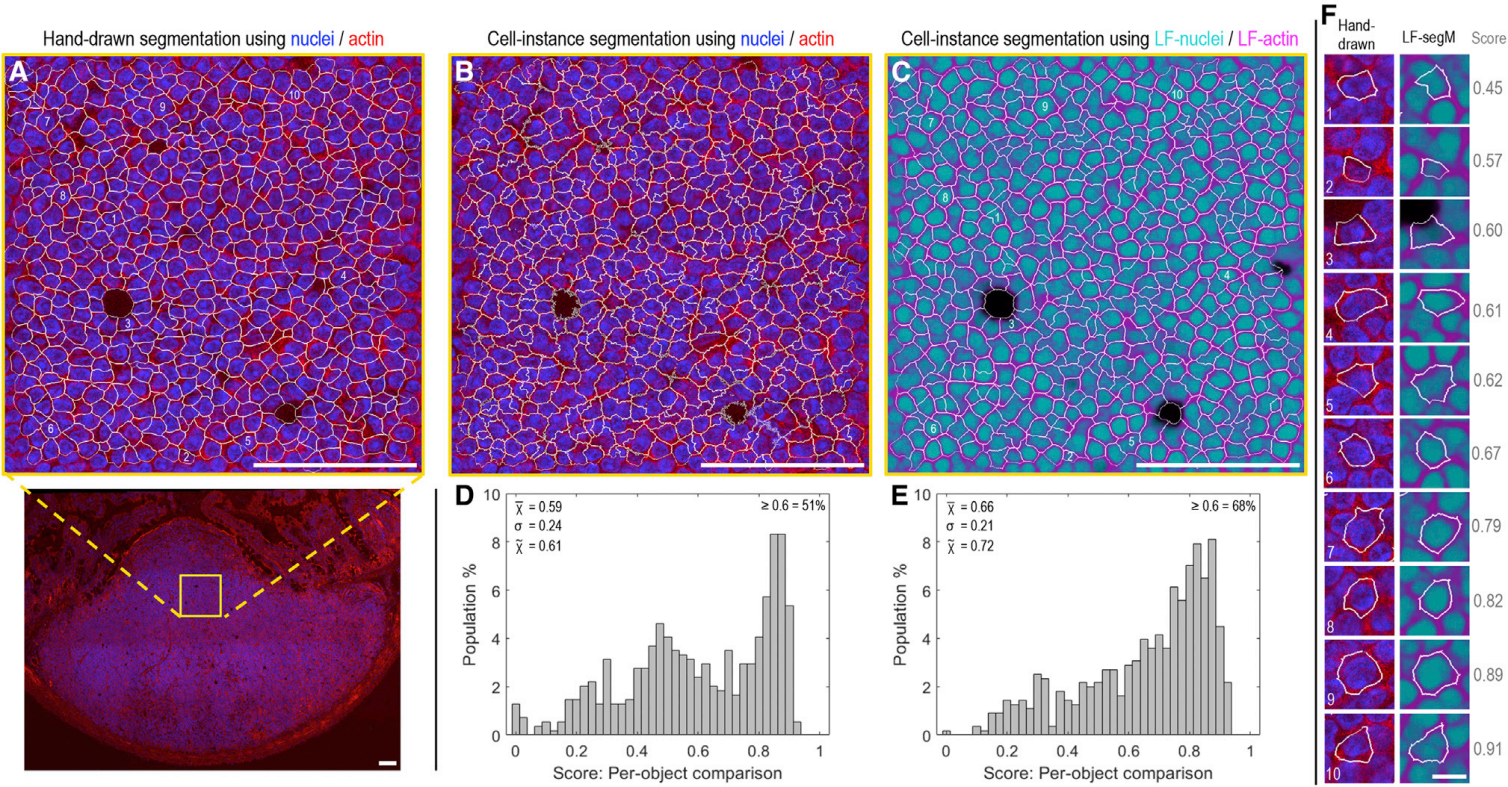
Assessing cell segmentation accuracies using mouse Peyer’s patch tissue (A) Hand-drawn cell segmentation performed using nuclei/actin fluorescence information for the tissue region indicated by the yellow box in the wider, tile-scanned image. (B and C) Automated cell instance segmentations for the same image-region as (A) using either (B) the raw nuclei and actin fluorescence data or (C) the label-free probability maps obtained from the neural network using reflectance alone as input (image data from this tissue section were unseen during training). (D and E) Cell-object intersection-over-union (IOU) score distributions comparing the hand-drawn cell segmentations shown in (A) against the automated cell segmentations shown in (B and C) using either (D) fluorescence or (E) label-free information. (F) Example hand-drawn versus label-free cell segmentation comparisons and IOU scores. The positions of each cell in the source images are shown by the cell-object numberings in (A), (C), and (F). An IOU score of 1 represents perfect per-pixel overlap between hand-drawn and automated cell segmentations. (F) Within the comparison presented here, scores ≥0.6 are seen to represent a good match, approaching the limit of hand-drawing accuracy given the relatively low resolution of the source image data. (A–C) Scale bars, 100 μm. (F) Scale bar, 10 μm.

To probe the accuracy of the cell segmentation achieved while exploring the compatibility of the approach with different tissue types, we moved on to tissue sections from intestinal Peyer’s patches (Figure 2A), which are key players in the orchestration of mucosal and systemic antibody responses for the microbiome, food, and oral vaccines. To do this, the cell segmentation achieved in CellProfiler using either the fluorescence information (Figure 2B) or the label-free probability maps from the reflectance data (Figure 2C) was compared against the results of careful manual annotation (Figure 2A) using the intersection-over-union (IOU) metric. The label-free approach outperformed (Figures 2D–2F) the results obtainable direct from the fluorescence information (median IOU 0.72 versus 0.61, respectively) while additionally saving two channels.

**Figure 3:**
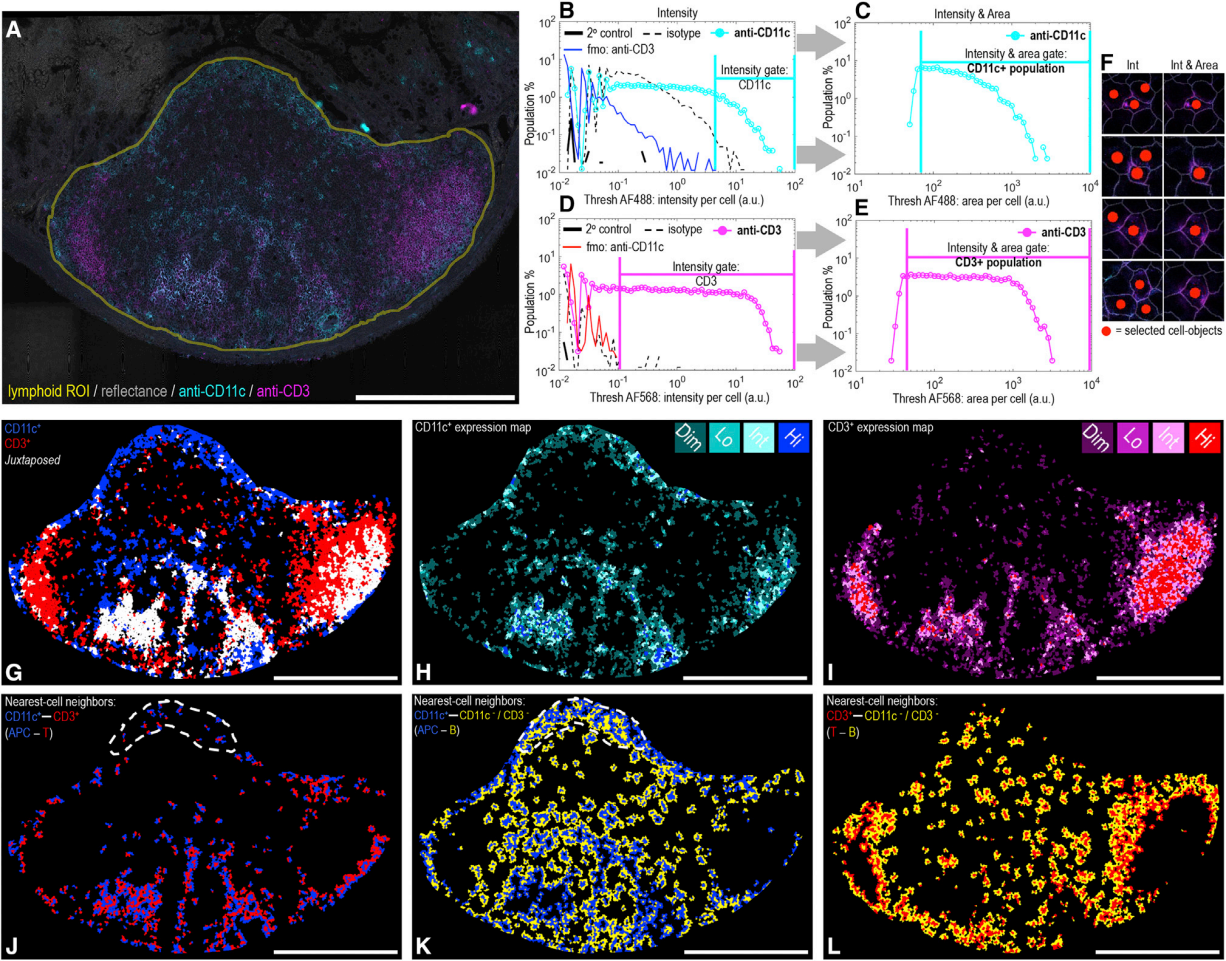
Label-free cell segmentation enables image-based cell profiling (A) Tile-scanned mouse Peyer’s patch tissue section imaged for reflectance in addition to immunofluorescence markers for CD11c (i.e., mononuclear phagocyte antigen-presenting cells) and CD3 (T lymphocytes). The yellow region of interest (ROI) represents the lymphoid tissue upon which the label-free cell segmentation approach was deployed (∼16,000 cells). Outside of the ROI, the reflectance image is seen to still provide interpretable histological context. (B–E) Flow cytometry-type gating to establish CD3^+^ and CD11c^+^ cell populations informed by secondary only, fluorescence-minus-one (fmo) and isotype single-cell fluorescence distributions obtained from label-free cell object data collected from adjacent, serial tissue sections. Due to the dense cellular packing of lymphoid tissue, (C and E) second sequential gates on the fluorescence area occupied per cell object helped to reduce (F) bystander-positive events caused by fluorescence overlap into neighboring cells. (G) Cell map view showing the gated cell populations *in situ* using flood filling of label-free cell-objects. Juxtaposed CD11c-CD3 neighboring cells that still identified positive for both CD markers after bystander removal are shown in white. (H and I) CD11c and CD3 expression maps with cell objects shaded into four levels (dim, low, intermediate, high) according to each segmented cell’s level of immunofluorescence. (J–L) Nearest-cell-neighbor maps simplifying the view shown in (A) to only show touching groups of cell objects according the combinations (J) CD11c^+^-CD3^+^ (i.e., APC-T), (K) CD11c^+^-CD3^-^/CD11c^−^ (i.e., APC-B), and (L) CD3^+^-CD3^−^/CD11c^−^ (i.e., T-B). In this way, the views give a sense of key cell types within interactive distances of one another. The dashed line in (J) and (K) indicates the subepithelial dome tissue region. Scale bars: 500 μm.

Using a parallel tissue section to Figure 2 (i.e., with cell segmentation accuracy established), we next considered the ability of the approach to simplify image-based cell profiling. As such, we replicated a recently published experiment^2^ that had previously required dedicated nuclei and actin fluorescent stains to achieve accurate single-cell and nearest-cell-neighbor measurements. Mouse Peyer’s patch tissue sections were dual immunolabeled for CD11c (identifying mononuclear phagocytes, typically antigen-presenting cells) and for CD3 as a pan T lymphocyte marker. Alongside this, using tissue-matched serial sections, secondary-only controls, isotype controls, and fluorescence-minus-one controls were prepared to inform on background, non-specific antibody binding and fluorescence cross-talk, respectively (see STAR Methods). In half of the previously required imaging time, each tissue section was tile scanned for fluorescence information with concomitant collection of reflected light. Figure 3A exemplifies the outcome, with a region of interest placed around the lymphoid tissue. Guided by the control data, CD11c^+^ and CD3^+^ cell populations were built using single-cell fluorescence measurements and simple, flow cytometry-type gating (Figures 3B–3E). As found previously,^2^ a second sequential gate on the area occupied by fluorescence within each cell (Figures 3C and 3E) helped to reduce “bystander-positive” events caused by the surface-located fluorescence spanning segmented cell outlines into immediately adjacent cell objects (Figure 3F). From this simplified experiment—now using just two labels instead of the four previously required—diverse information regarding cell location, expression, and nearest-cell-neighbor relationships was obtainable (Figures 3G–3L). Interestingly, a population of highly juxtaposed, CD11c-CD3 neighboring cells that still identified positive for both markers after bystander removal were identifiable, suggesting a high likelihood of cell-cell interaction (Figure 3G). Visually intuitive cell expression maps for CD11c and CD3 could also be assembled (Figures 3H and 3I). Use of the label-free approach also enabled identification and segmentation of all of the unlabeled (i.e., CD11c^–^/CD3^–^) cells, which, in the Peyer’s patch environment, predominantly represent B lymphocytes.^14^ Hence, the spatial distribution of antigen-presenting cell (APC)-T, APC-B, and T-B lymphocytes that were within interactive distances of one another as nearest-cell neighbors could also be isolated and mapped from the label-free objects (Figure 3J–3L). In this regard, comparing Figures 3J and 3K, a predominance of APC-B interactions, as opposed to APC-T interactions, were observed within the immunoactive subepithelial dome region of the tissue.^15^

In addition to frozen samples, tissue specimens are also commonly archived in formalin-fixed paraffin-embedded (FFPE) format. As a final 2D experiment, we therefore considered if the approach was transferable to this section type. Of note, F-actin staining using phalloidin conjugates is known to fail in FFPE sections because the actin cytoskeleton is degraded by solvent exposures incurred during FFPE processing.^2^ The cell outline ground-truth fluorescence labeling on the training slide was therefore switched to cell membrane (i.e., phospholipid) staining using fluorescently conjugated wheat germ agglutinin (WGA). Despite a notably different reflectance signal (presumably due to cytoskeletal degradation), a segmentable relationship between the reflectance information and the WGA staining was learnable (Figure S4). Encouragingly, similar IOU scores (FFPE median score = 0.74) were attainable to those achieved using frozen sections (0.72) (Figures S4 and 2). In this way, segmentations based on training exemplifications from a different fluorescence label unlocked this important section type to the label-free technique.

**Figure 4:**
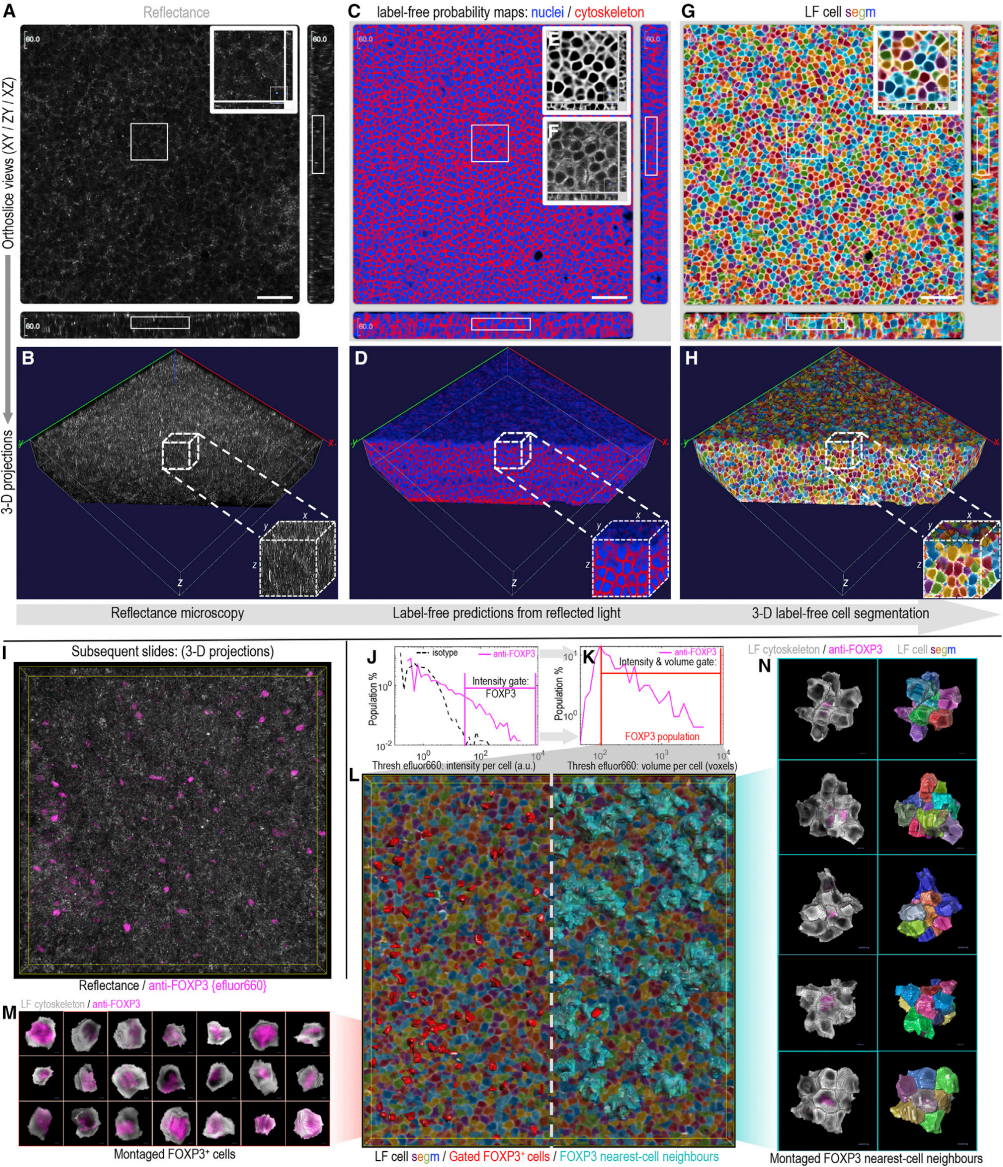
3D label-free cell segmentation of tissue microscopy image data collected by routine confocal microscopy (A–H) Stepwise exemplification of the 3D strategy using z stack image data of mouse mesenteric lymph node tissue. Outcomes at each step are displayed by (A, C, and G) orthoslice and (B, D, and H) 3D volumetric projection views, with the latter cut away to better display outcomes along the z dimension. (A and B) 3D reflectance signal. (C–F) Label-free probability maps outputted from a 3D U-Net neural network (C and D) with insets demonstrating the (E) label-free probability map representation for the cytoskeleton compared with (F) fluorescent cytoskeletal F-actin staining. (G and H) 3D label-free cell segmentation results where (repeated) filled colors represent individual cell objects. (I–N) Validation of the 3D label-free approach using tissue sections immunolabelled for FOXP3 or matched isotype control with no nuclei or actin staining present. (J and K) Flow cytometry-type gating using cell-object fluorescence distributions to establish FOXP3^+^ events from cell intensity and fluorescence volume information. (L) 3D projection of the label-free cell segmentation results. On the left, gated FOXP3^+^ cells are identified using red surface overlays. On the right, both FOXP3^+^ events (red) and their touching nearest-cell neighbors (cyan) are shown *in situ*. (M), 3D projections of individual FOXP3^+^ cell objects cut out and montaged from the label-free segmentation. An intranuclear core of FOXP3 staining is visible surrounded by the label-free probability map for the cytoskeleton classification. (N) 3D projections of individual FOXP3^+^ cell objects and their touching nearest-cell neighbors cut out and montaged using the label-free cell segmentation. (A, C, and G) Scale bars: 50 μm*.*

With the capability of our approach established in 2D, we moved forward to 3D imaging with the goal of retrieving entirely label-free segmentations for all cells in imaged volumes (Figure 4). Previously, T cell clustering in secondary lymphoid tissues (lymph nodes) and the role that FOXP3^+^ regulatory T cells play in suppressing potentially autoreactive T cells were demonstrated using 3D imaging and the “histocytometry” approach.^4^ In that work, segmentations were achieved for cells expressing a fluorescent marker—but not for unlabeled cells. Here, a simple extension to a 3-D U-Net architecture (Method S1) enabled the generation of probability maps from z stacked reflectance information that were easily segmentable into 3D cell objects using CellProfiler 4 (Figures 4A–4H). As before, cell segmentation accuracies in the xy, zy, and xz dimensions were assessed against manual annotations using the IOU approach (Figure S5). Encouragingly, use of a 3D network leveraging data across multiple z planes simultaneously improved the segmentation accuracies (median IOU scores xy = 0.84, zy = 0.74, xz = 0.78) achievable relative to the 2D network results (xy = 0.72) (Figures S5 and 2).

To test the ability of the approach to retrieve single-cell and nearest-neighbor relationships in 3D, immunofluorescence data for FOXP3 or isotype control were collected (Figure 4I). As in 2D, flow cytometry-type gating established the FOXP3^+^ cell population *in situ* (Figures 4J–4L). FOXP3^+^ cells were, indeed, isolatable as cell populations of independent events (Figure 4M), and harnessing the segmentation information of the unlabeled cells further allowed 3D identification and visualization of all nearest-cell neighbors with interactive potential (Figure 4N). In this way, individual cells, with or without “touching” nearest-cell neighbors, could be isolated and examined as independent populations in a manner similar to imaging flow cytometry,^16^^,^^17^ with the additional ability to rotate, cut away, and consider objects and their contents from any angle (Figure 4M). Moreover, with tissue-relevant localization retained, the full *in vivo* cell-cell environment incorporating all nearest neighbors in 3D was revealed (Figures 4L–4N). The approach was tested with tissue sections up to ∼100 μm in thickness. At this depth, the fluorescence from typical blue nuclear labels (e.g., Hoechst 33342 or DAPI) is attenuated by the tissue thickness and cannot serve as an input for accurate segmentation, whereas the label-free strategy still operated effectively (Figure S6).

## Discussion

There is a growing need for accessible means to obtain *in situ*, single-cell information from tissue images across the bioclinical sciences. A major barrier is the relatively few channels, for separate biomarkers, that conventional microscopy allows for experimentation (often ≤6 in practice). This becomes compounded when two of these channels are required to achieve accurate cell segmentation. Imaging mass spectrometry systems, which are capable of resolving many metal-conjugated antibodies, may partially obviate these issues, but instrumentation is not widely available. In practice, to move bioclinical research beyond “representative image” reporting, 2D and 3D cell-based quantitation of tissues with standard confocal microscopy equipment must become routine, and the workflow from image data to cell features must be disseminable.^2^^,^^4^^,^^5^

Previously, fluorescence image restoration^18^ and virtual *in vitro* cell labeling^7^^,^^8^ have been demonstrated as powerful applications of fluorescence image reconstruction by deep learning. In their seminal paper, using a custom-built multimodal reflectance microscope, Cheng et al. also showed that fluorescent stain predictions from reflected light information could be used to achieve 2D segmentation of monolayer cells in culture. Notably, however, *ex vivo* tissue microscopy offers a very different challenge to cell microscopy. Tissue is made up of multiple different cell types and extracellular features in dense, layer-upon-layer arrangements in a way that is not present *in vitro.* The image information is also fundamentally different due to the histological preparation steps (fixation, embedding, and sectioning) that are different or not required for cultured cells. Here, we recognized that during confocal imaging with entirely standard equipment, there is always freely available “byproduct” reflected light. Our work now shows that this carries sufficient information, with sufficient penetration, to establish accurate 2D and 3D segmentations of cells in tissues. To do this, we use classification and probability mapping—as opposed to regression-based fluorescent stain predictions—as intensity uniformity across the outputted probability maps is advantageous to the cell segmentation task. Moreover, we provide the software, data, and video tutorials necessary to remove the programming barrier to access, making this accessible for everyone. Because generalizability is essential to the practical utility of any method, we carefully demonstrate our approach using data from two different confocal microscopes across 40× and 63× objective lenses in three different tissues using reflectance from three different laser lines across four image resolutions (from 3.5 to 8.3 pixels per μm). We also demonstrate the compatibility of the approach with both frozen and FFPE tissue section types.

An important aspect of the presented method is that it allows the assembly of large amounts of sample-matched training data without the need of cell annotation. This enables conventional U-Net models to be trained with bespoke exemplifications of the task,^13^^,^^18^ maximizing performance while providing data at a scale sufficient to avoid memorization and enable rigorous cross-validation testing.^19^ These training data are prepared using antibody-independent affinity staining, enabling easy transfer across species^2^ while minimizing pixel labeling errors through avoidance of non-specific binding.^7^

In this way, the presented work enables a move beyond disaggregated flow cytometry measurements and qualitative microscopy reporting by harnessing label-free information from every cell in a tissue section such that cell content and *in situ* location can be reported together. Detailed cell-cell interactions (nearest-neighbor-type relationships) in complex tissue environments are achievable and will provide the bridge between deep immunological knowledge of single cell types and macroscopic tissue function. All data, code, and methodological steps are available for download alongside detailed video tutorials demonstrating deployment in Python, MATLAB, or provided standalone software for Windows.

### Limitations of the study

This technique relies on the existence of a relationship between the reflectance signal and the fluorescence information used to determine the ground truth.^18^^,^^20^ Moreover, this relationship must describe the cellular structure in a manner that enables accurate cell-object segmentations. Here, we demonstrate that label-free predictions of cytoskeletal or cell membrane structure enable this from frozen and paraffin-embedded tissue sections for diverse lymphoid tissues (spleen, Peyer’s patch, and mesenteric lymph node), where cell relationships are so important in establishing fundamental biology including responses to infection, vaccination, and carcinogenesis. However, this approach may not work in every tissue type as it is dependent upon the specific structural morphology of cell and tissue and the resultant optical scattering coefficients.

## STAR★Methods

### Key resources table

**Table:** 

REAGENT or RESOURCE	SOURCE	IDENTIFIER
**Antibodies**		

Rat anti-mouse CD3-EF450	Thermo Fisher	Cat #48-0032-82
Rat anti-mouse CD4-PE	Thermo Fisher	Cat #12-0041-83
Hamster anti-mouse CD11c-EF660	Thermo Fisher	Cat #50-0114-82
Rat anti-mouse FOXP3-EF660	Thermo Fisher	Cat #50-5773-82
Rabbit anti-mouse CD3	Abcam	Cat #AB5690
Hamster anti-mouse CD11c	Abcam	Cat #AB33483
Goat anti-Rabbit IgG (H + L) Alexa Fluor 568	Thermo Fisher	Cat #A-11011
Goat anti-Hamster IgG (H + L) Alexa Fluor 488	Thermo Fisher	Cat #A-11008

**Biological samples**		

Frozen C57BL/6J mouse spleen sections	This paper	N/A
Frozen C57BL/6J mouse Peyer’s patch sections	This paper	N/A
Frozen C57BL/6J mouse mesenteric lymph node sections	This paper	N/A
Formalin-fixed paraffin embedded C57BL/6J mouse Peyer’s patch sections	This paper	N/A

**Chemicals, peptides, and recombinant proteins**		

Hoescht 33,342	Thermo Fisher	Cat #H3570
Phalloidin-AlexaFluor 647	Thermo Fisher	Cat #A22287
Wheat germ agglutinin-AlexaFluor 555	Thermo Fisher	Cat #W32464

**Deposited data**		

Raw and analyzed microscopy data	This paper	https://www.ebi.ac.uk/biostudies/studies/S-BSST742

**Experimental models: Organisms/strains**		

Mouse: C57BL/6J	Charles River	Cat #027

**Software and algorithms**		

MATLAB code	This paper	Method S1 or https://www.ebi.ac.uk/biostudies/studies/S-BSST742
Python code	This paper	Method S1 or https://www.ebi.ac.uk/biostudies/studies/S-BSST742
Standalone label free prediction software	This paper	https://www.ebi.ac.uk/biostudies/studies/S-BSST742
MATLAB R2021a (or later)	MathWorks	https://uk.mathworks.com/products/new_products/release2021a.html
Deep Learning Toolbox 14.3	MathWorks	https://uk.mathworks.com/help/deeplearning/
Image Processing Toolbox 11.4	MathWorks	https://uk.mathworks.com/products/image.html
Computer Vision Toolbox 10.1	MathWorks	https://uk.mathworks.com/products/computer-vision.html
Python 3.6	python.org	https://www.python.org/downloads/release/python-360/
tensorflowgpu 1.9.0	tensorflow.org	https://pypi.org/project/tensorflow-gpu/1.9.0/
Keras 2.1.5	keras.io	https://pypi.org/project/keras/2.1.5/
numpy 1.18.1	numpy.org	https://pypi.org/project/numpy/1.18.1/
scipy 1.4.1	scipy.org	https://pypi.org/project/scipy/1.4.1/
pandas 1.0.3	pandas.pydata.org	https://pypi.org/project/pandas/1.0.3/
scikit-learn 0.22.1	scikit-learn.org	https://pypi.org/project/scikit-learn/0.21.1/
scikit-image 0.16.2	scikit-image.org	https://pypi.org/project/scikit-image/0.16.2/
pillow 8.4.0	pillow.readthedocs.io	https://pypi.org/project/Pillow/8.4.0/
ipython 7.13.0	ipython.org	https://pypi.org/project/ipython/7.13.0/
opencv 4.2.0.34	opencv.org	https://pypi.org/project/opencv-python/4.2.0.34/
javabridge 1.0.19	pythonhosted.org	https://pypi.org/project/javabridge/
bioformats 1.5.2	openmicroscopy.org	https://pypi.org/project/python-bioformats/1.5.2/
matplotlib 3.3.4	matplotlib.org	https://pypi.org/project/matplotlib/3.3.4/
H5py 2.10.0	h5py.org	https://pypi.org/project/h5py/2.10.0/
Imageio 2.11.0	imageio.readthedocs.io	https://pypi.org/project/imageio/2.11.0/
Java SE Development kit 11.0	oracle.com	https://www.oracle.com/uk/java/technologies/javase/jdk11-archive-downloads.html
CUDA Toolkit 9.0	developer.nvidia.com	https://developer.nvidia.com/cuda-90-download-archive
cuDNN 7.6.4	developer.nvidia.com	https://developer.nvidia.com/cudnn
Cell Profiler 4.1.3 (or later)	cellprofiler.org	https://cellprofiler.org/releases
Vaa3D	alleninstitute.org	https://github.com/Vaa3D/release/releases/

**Other**		

Zeiss LSM 780 confocal microscope	Zeiss	https://www.zeiss.com/microscopy/en/products/light-microscopes/confocal-microscopes.html
Leica SP8 confocal microscope	Leica Microsystems	https://www.leica-microsystems.com/products/confocal-microscopes/

### Resource availability

#### Lead contact

Further information and requests for resources and reagents should be directed to and will be fulfilled by the lead contact, John W. Wills (jw2020@cam.ac.uk).

#### Materials availability

This study did not generate new unique reagents.  

### Experimental model and subject details

#### Murine tissues

Spleen, ileum (containing Peyer’s patches) and mesenteric lymph node tissues were collected from healthy, male C57BL/6 mice (n = 4) (8-12 week-old) sacrificed by carbon dioxide asphyxiation. Tissues for cryosection analysis were snap-frozen in isopentane cooled on dry ice before storage in liquid nitrogen until use. Tissues for FFPE processing were fixed in neutral buffered formalin (4 h) prior to transfer to tissue cassettes. Samples were embedded in paraffin by dehydrating through an aqueous ethanol series (5 min each 20%, 50% 70% (100% x2) v/v) followed by three changes of 100% xylene (30°C) then three changes of paraffin wax (62°C). All animal work complied with the University of Cambridge Ethics Committee regulations and was performed under the Home Office Project License numbers 80/2572 and P48B8DA35.

### Method details

#### Tissue sectioning

Frozen tissues were transferred into the cryostat chamber (- 15°C) and acclimatised for 30 min. Samples were trimmed to remove any excess fat, and transferred to cryomolds containing pre-chilled optimal cutting temperature compound (OCT) (#00411243, VWR). Sections were cut at 25 or 100 micron thicknesses (for 2D or 3D imaging, respectively) and collected on Super-Frost Plus adhesion treated slides (#J1800AMNT, Thermo) before resting at room temperature for 2 h prior to immunofluorescence labeling. Formalin-fixed, paraffin embedded (FFPE) sections were cut at 5 μm thickness. FFPE sections were dewaxed by baking at 60°C for 1 h prior to changing twice through xylene. Prior to fluorescence counterstaining, FFPE sections were rehydrated using a reverse ethanol series (100%, 70%, 50%, 10%; 5 min each) followed by immersion in water (1 min).

#### Immunofluorescence labeling

Tissue sections were ringed with hydrophobic barrier pen (Vector, #H-4000). Frozen sections were fixed using fresh 4% paraformaldehyde in 0.1 M PBS (pH 7.4) at room temperature for 10 or 20 min (25 μm or 100 μm sections, respectively). All subsequent steps were carried out under gentle agitation on a rotating shaker. To facilitate antibody penetration, the frozen sections were permeabilised for 2 or 4 h using 0.3% (v/v) Triton X-100 in 0.1 M PBS (pH 7.4) (25 μm or 100 μm sections, respectively). Sections were then blocked using 25 mM TBS (pH 7.4) supplemented with 10% (v/v) goat serum (ThermoFisher, #16210064), 2% (w/v) BSA (BioSera, #PM-T1726) and 20 mM glycine for 2 h. Primary antibodies or isotype controls were prepared in block buffer and added at 150 μL per section for 18–24 h at 4°C. All subsequent steps took place at room temperature. Sections were washed (3 × 3 min, TBS) prior to incubation with secondary antibodies (when needed) diluted in block buffer for 4 or 8 h (see Table S1 for antibody concentrations, fluorophores conjugations and manufacturer information) (25 μm or 100 μm sections, respectively). After washing (3 × 3 min, TBS) frozen sections destined for the provision of training data underwent nuclear and f-actin counterstaining using 2 μg/mL Hoechst 33,342 (#H3570, Thermo) and 500 nM phalloidin-AlexaFluor 647 (#A22287, Thermo) in TBS for 1 h. The cellular structure of the FFPE sections was counterstained by labeling cell membranes using 20 μg/mL wheat-germ agglutinin (WGA) conjugated with Alexa Fluor 555 (#W32464, Thermo). After counterstaining, all sections were washed for a final time (1 × 3 min, TBS) before mounting with #1.5 coverslips in Prolong Glass mountant (#P36980, Thermo).

#### Antibody controls

Three antibody controls commonly used by the flow cytometry community were measured in tissue-matched serial sections. Secondary-only controls received just the secondary antibody in absence of any primary antibody. Any signal in the collection channel for this control thus represented endogenous tissue autofluorescence or contributions from the fluorophore-conjugated secondary antibody binding non-specifically in the tissue section. Fluorescence-minus-one (FMO) controls contained all of the fluorescent stains – bar the one under quantification. Here, any signal in the collection channel was typically from ‘spill over’ from the other fluorophores into this empty channel. Finally, ‘isotype controls’ switched out the primary antibody for an irrelevant antibody (*i.e.,* raised against an antigen not present in the sample) but otherwise identical (*i.e.* same isoclass) to the primary antibody. Here, non-specific binding of this irrelevant primary antibody or capture by, *e.g.,* Fc receptors led to signal in the collection channel, informing on the level of non-specific binding.

#### Confocal microscopy

The label-free strategy was developed using image data collected from two commonplace (Leica SP8/Zeiss LSM 780) laser scanning confocal microscopy platforms. The SP8 was inverted configuration, whilst LSM780 was upright. No modifications from standard were necessary to enable the presented approach. Detailed instructions for setting up reflected light collection are provided in Method S1. Image data for the presented 2D analyses were collected using the SP8 via 40X/1.3 or 63X/1.4 oil immersion objectives. Reflectance was collected from the 488 nm laser via detector placement +/− 3 nm either side of the excitation line (*i.e.,* detection in the range 485–491 nm). 3D data were collected using the LSM 780 via the 40X/1.3 oil immersion objective. Reflectance was collected from the 561 nm laser via detector placement +/− 9 nm either side of the excitation line (*i.e.,* 552-600 nm). Tilescans were conducted with 10% edge overlap to facilitate registration. Details of the tissue specimen, image dimensions and pixel/voxel densities are provided for all image data in Table S1.

### Quantification and statistical analysis

#### Label-free cell segmentation workflow

Neural network training data was collected from parallel tissue sections to those undergoing immunofluorescence labeling. Training data was achieved by collecting fluorescence images for nuclei and cytoskeletal f-actin (frozen sections) or nuclei and the cell membrane (FFPE sections) (fluorescence staining described above) alongside the ‘paired’ reflectance signal. Binary pixel classification labels were assembled by thresholding the fluorescence information to create pixel label classes representing ‘nuclei’, ‘cytoskeleton’ and ‘background’ classifications. Because of the paired nature of the training and test image-data (*i.e.,* collected using the same microscope settings) input reflectance data were rescaled in the zero-one interval with no contrast adjustment prior to inputting into the 2D or 3D U-Nets (architectures shown, Method S1). Using cross-entropy loss, the networks were then trained to output the probability that pixels in the reflectance image belonged to each of the classifications with the probability image from the loss function serving as the direct input for cell segmentation. Network training, optimisation and validation testing was conducted using MATLAB R2021a and the Deep Learning, Image Processing and Computer Vision toolboxes (described, Method S1). Scripts for running the 2D and 3D U-Nets were also written for Python 3 using keras/TensorFlow-gpu 1.9 (TensorFlow install guide and U-Net scripts described, Method S1). The probability map images outputted by the U-Net networks were segmented into 2D or 3D cell objects using marker-controlled watershed algorithms deployed in CellProfiler^12^ (version 4.1.3) (instructions for installing CellProfiler and running the 2D and 3D image analysis pipelines are provided in Method S1). In all instances, neural networks were trained on data from one tissue section before validation testing using data collected from an entirely different tissue section.

#### 2D U-NET

Reflectance data were passed to the network as patches with dimensions 256 × 256 × 1 (x, y, channels) with augmentation by x/y reflection and rotation. The three-class U-Net architecture used an encoder depth of 4 with 64 filters in the first layer (shown, Method S1). Complete up-convolutional expansion was used to provide probability maps of the same size as the input images. Training lasted for 50 epochs (frozen sections) or 150 epochs (FFPE sections) using a batch size of 12 with zero-center normalisation (demonstrated, Video S1). Training was optimised using stochastic gradient descent using cross-entropy loss. The initial learning rate was 0.05, dropping every 10 epochs by 0.1 under momentum 0.9 and L2 regularisation 1x10^−4^. Patches were shuffled every epoch.

#### 3D U-NET

Reflectance data were patched through the network with input dimensions 64 × 64 × 64x1 (x, y, z, channels) and augmentation by x/y reflection and rotation. The three-class U-NET architecture used an encoder depth of 4 with 64 filters in the first layer (shown, Method S1). Complete up-convolutional expansion was used to output probability maps of the same dimensions as the source microscopy data. Training lasted for 150 epochs using a batch size of 8 with zero-center normalisation. Training was optimised under ADAM using cross-entropy loss. The initial learning rate was 5x10^−4^, dropping every 5 epochs by 0.95 under L2 regularisation 1x10^−4^. Patches were shuffled every epoch.

#### 2D/3D U-NET: Standalone windows software

The label-free prediction software (described, Method S1) for Windows was built in MATLAB R2021a using the MATLAB App Designer and MATLAB Compiler. This enables 2D/3-D U-NET training and deployment via a simple graphical user interface removing the need for programming expertise.

#### Segmentation accuracy

Using the Jaccard index (intersection over union) approach, 2D and 3D label-free cell segmentation accuracies were assessed by comparing pixel positions within automatically segmented cell objects against those inside manually-drawn cell outline annotations. To assess the 3D segmentations, annotations of XY as well as ZY and XY dimensions were used to fully explore the validity of the segmented cell objects along all three dimensions. The Jaccard index was calculated as:

(Equation 1) $$J\left(A,M\right)=\frac{\left|A\cap M\right|}{\left|A\cup M\right|}=\frac{\left|A\cap M\right|}{\left|A\right|+\left|M\right|-\left|A\cap M\right|}$$

Where, J is the Jaccard distance for two sets containing pixel positions for the automated segmentation (A) and the manual annotation (M) respectively. A score of 0 represents no overlap (*i.e.,* false negative) whereas 1 represents exact pixel-for-pixel overlap. It is acknowledged that this approach is a relatively harsh success measure and that a score of ∼0.7 indicates a good segmentation result.^13^ This is due in-part to the inaccuracies that are inevitably present even in the human annotated data (*e.g.,* due to outline smoothing, ambiguity in determining the precise position of each cell’s boundary from the fluorescent staining information and available image resolution *etc.*).

#### Single-cell data extraction

After cell segmentation, subsequent CellProfiler modules enabled image preprocessing and cell feature extraction. 2D and 3D CellProfiler workflows are demonstrated in the Method S1. Immunofluorescence channels were thresholded at the level required to remove ∼95% of fluorescence in tissue-matched, secondary antibody-only control images.^2^ Fluorescence intensity values per cell, alongside cell size and shape features were then measured for all channels. Integration of binarized immunofluorescence images was used to measure the fluorescence area/volume.

#### Image analysis and data visulisation

Following recommended best practice,^21^ cell-objects lying outside of the fifth or 95^th^ percentiles by area (2D analyses) or volume (3D analyses) were discarded. Nearest-cell neighbors to gated startpoint cells were identified using a spherical structuring element to dilate the startpoint cell’s boundary by 3 pixels before identifying neighboring objects subsequently eroded. Immunofluorescence visualisations and gated cell-object surface overlays were created using the freely available Vaa3D software.^22^

### Additional resources

Image-data, code and screencast tutorials demonstrating deployment of the described label-free cell segmentation method using MATLAB (R2021a, using Deep Learning, Image Processing and Computer Vision toolboxes), Python 3 (using keras/Tensorflow-gpu 1.9) or via precompiled, standalone software for Windows are downloadable from the BioStudies database (https://www.ebi.ac.uk/biostudies/) under accession number S-BSST742.

## Data Availability

• Microscopy data are publically available as of the date of publication from the BioStudies database (https://www.ebi.ac.uk/biostudies/) under accession number S-BSST742. • All original code (in MATLAB and Python languages) is available in the Method S1 file. All code as well as the precompiled, Windows software has also been deposited at the BioStudies database (https://www.ebi.ac.uk/biostudies/) under accession number S-BSST742 alongside screencast tutorial videos demonstrating deployment. These files are publicly available as of the date of publication. • Any additional information required to reanalyse the data reported in this paper is available from the lead contact upon request.

## Supplemental information

Document S1. Figures S1–S6, Table S1, and Methods S1

Document S2. Article plus supplemental information
