# Supplementary material for: Label-free cell segmentation of diverse lymphoid tissues in 2D and 3D
Source: Cell Rep Methods. 2023 Feb 2;3(2):100398. doi: 10.1016/j.crmeth.2023.100398 (PMC10014308; doi:10.1016/j.crmeth.2023.100398)
Supplement: Document S1. Figures S1–S6, Table S1, and Methods S1 [file mmc1.pdf]

Cell Reports Methods, Volume *3*

Supplemental information

Label-free cell segmentation

# of diverse lymphoid tissues in 2D and 3D

John W. Wills, Jack Robertson, Pani Tourlomousis, Clare M.C. Gillis, Claire M. Barnes, Michelle Miniter, Rachel E. Hewitt, Clare E. Bryant, Huw D. Summers, Jonathan J. Powell, and Paul Rees

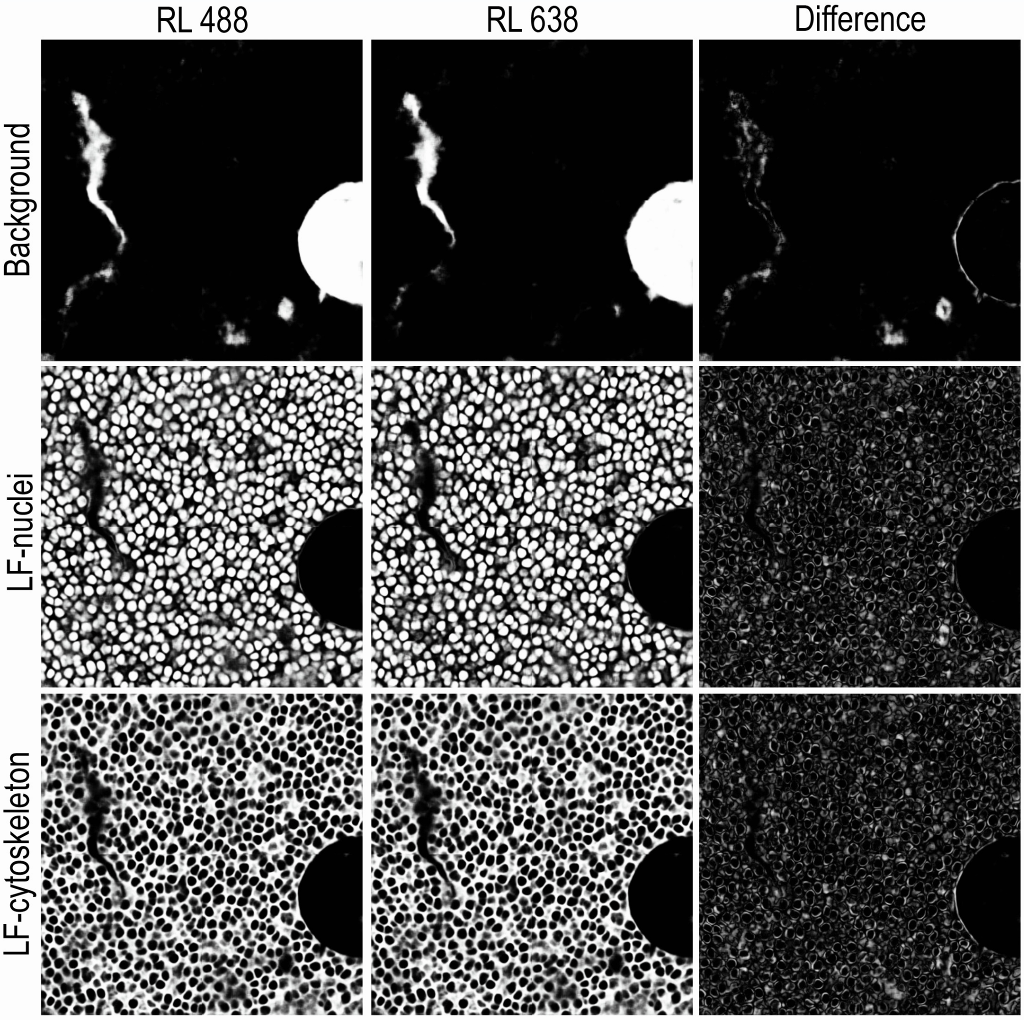

Figure S1 related to Figure 1 – Comparing label-free probability maps using reflectance data obtained using 488 nm or 638 nm laser excitation. The choice of excitation wavelength for generating the reflectance signal has minimal influence on the probability maps obtained from the network (left versus middle; difference shown right). The user can therefore reasonably use whatever is available on their individual microscope. For some applications, choosing a longer excitation wavelength may reduce fluorophore photobleaching / improve tissue penetrance and reflectance recovery - for instance, during 3-D Z-stack imaging in thicker tissue specimens.

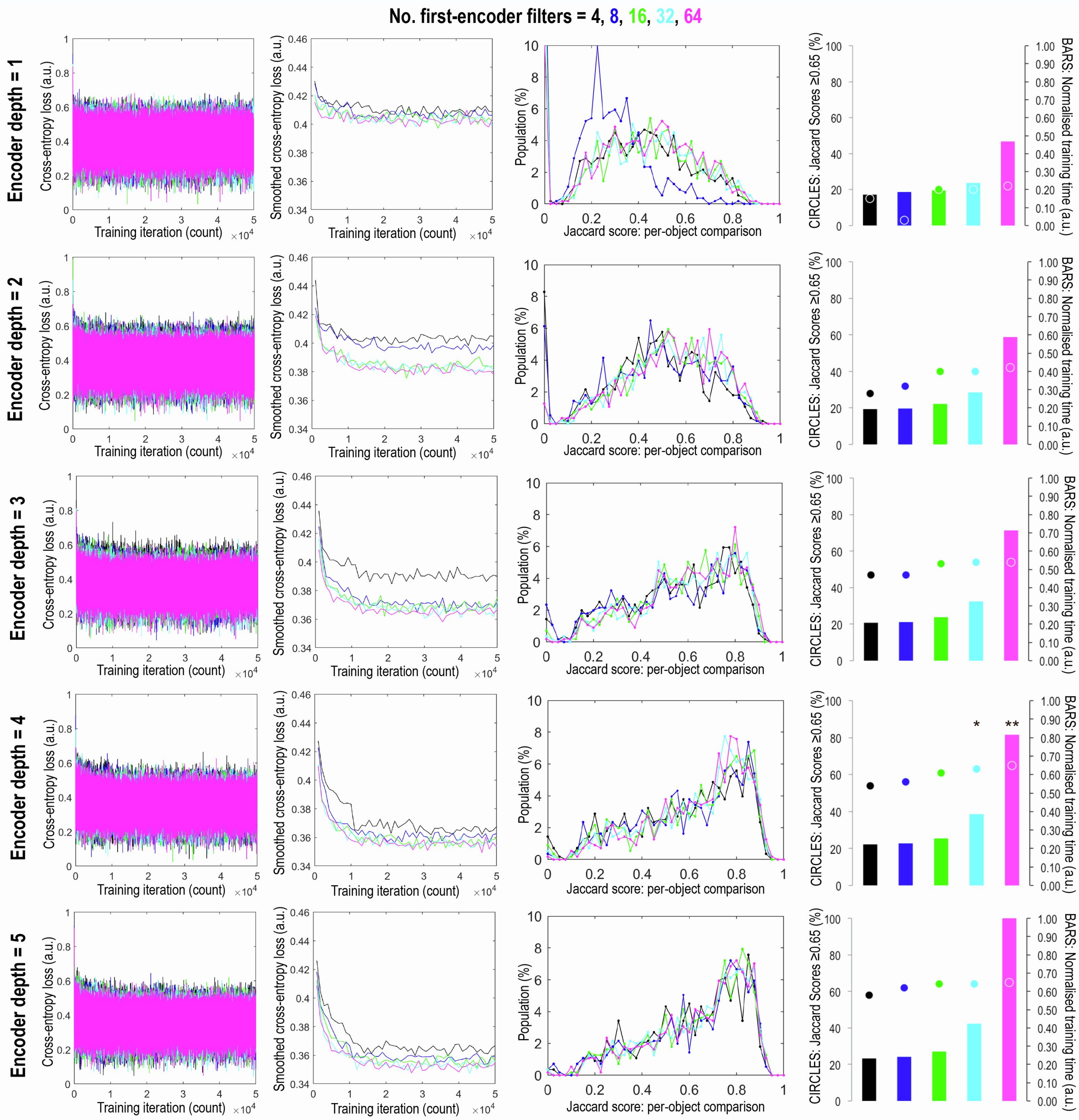

Figure S2 related to Figure 1 – Number of first-encoder filters and encoder depth optimisation to define the best performing 2-D Unet model. Bars represent normalised training time whilst circles indicate label-free cell segmentation accuracies (assessed by Jaccard index). The best performing model used an encoder depth of 4 with 64 filters at the level of the first encoder (indicated, **). Of note, using 32 filters instead of 64 can achieve a training speed up of ~50% for a negligible (~1%) decrease in segmentation accuracy (indicated, *). Increasing the encoder depth to 5 did not further improve cell segmentation accuracies (bottom row).

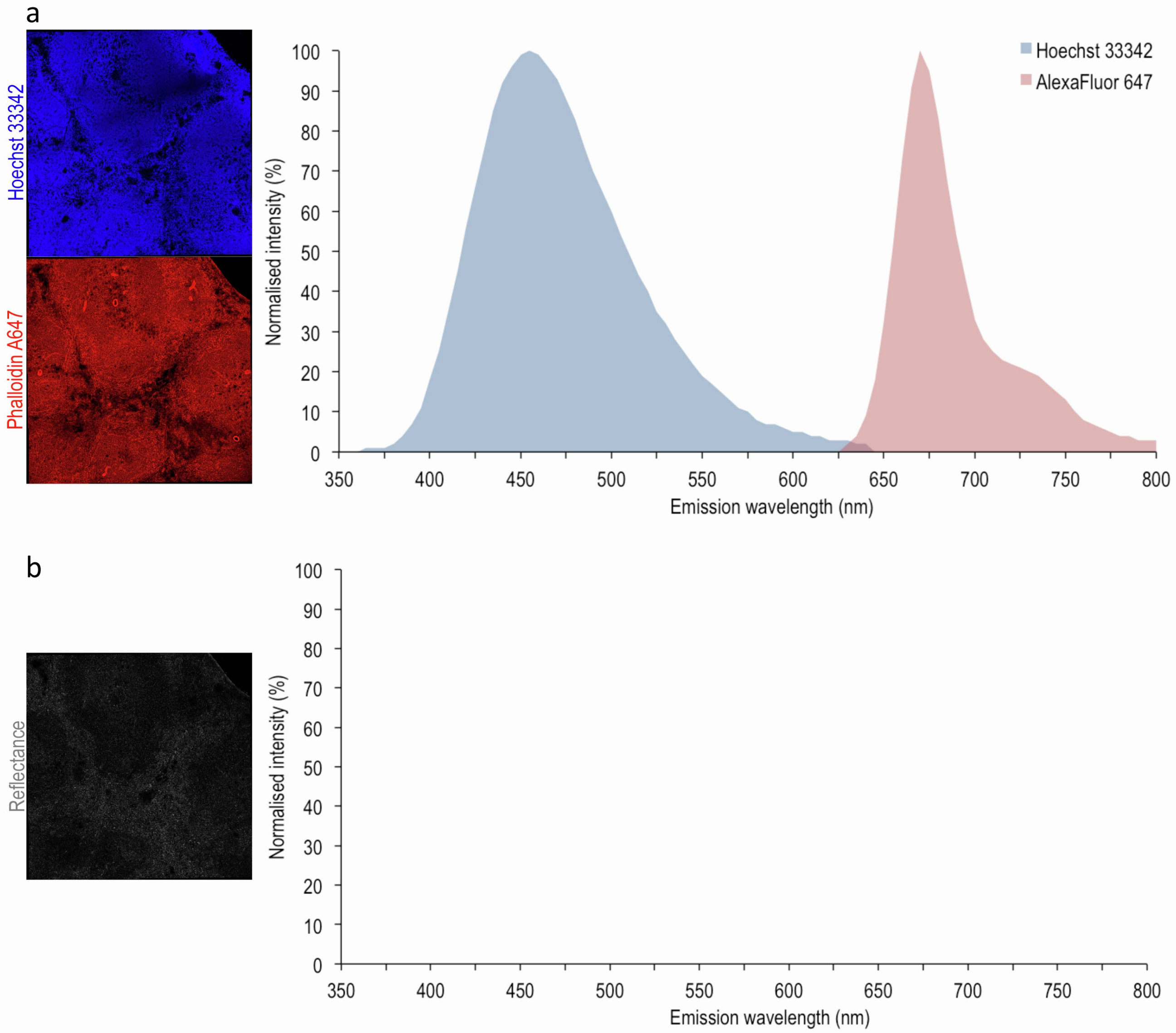

Figure S3 related to Figure 1 – Spectral bandwidth saving achieved by the label-free cell segmentation strategy. a, Emission spectra for Hoechst 33342 and AlexaFluor 647 as might typically be used to delineate cell nuclei and cell cytoskeletons when carrying out fluorescence-based cell segmentation. **b**, Harnessing reflectance information, the label-free cell segmentation method described here removes the need for these fluorescence stains leaving the spectrum entirely open for sensitive experimental measurements with single-cell quantification.

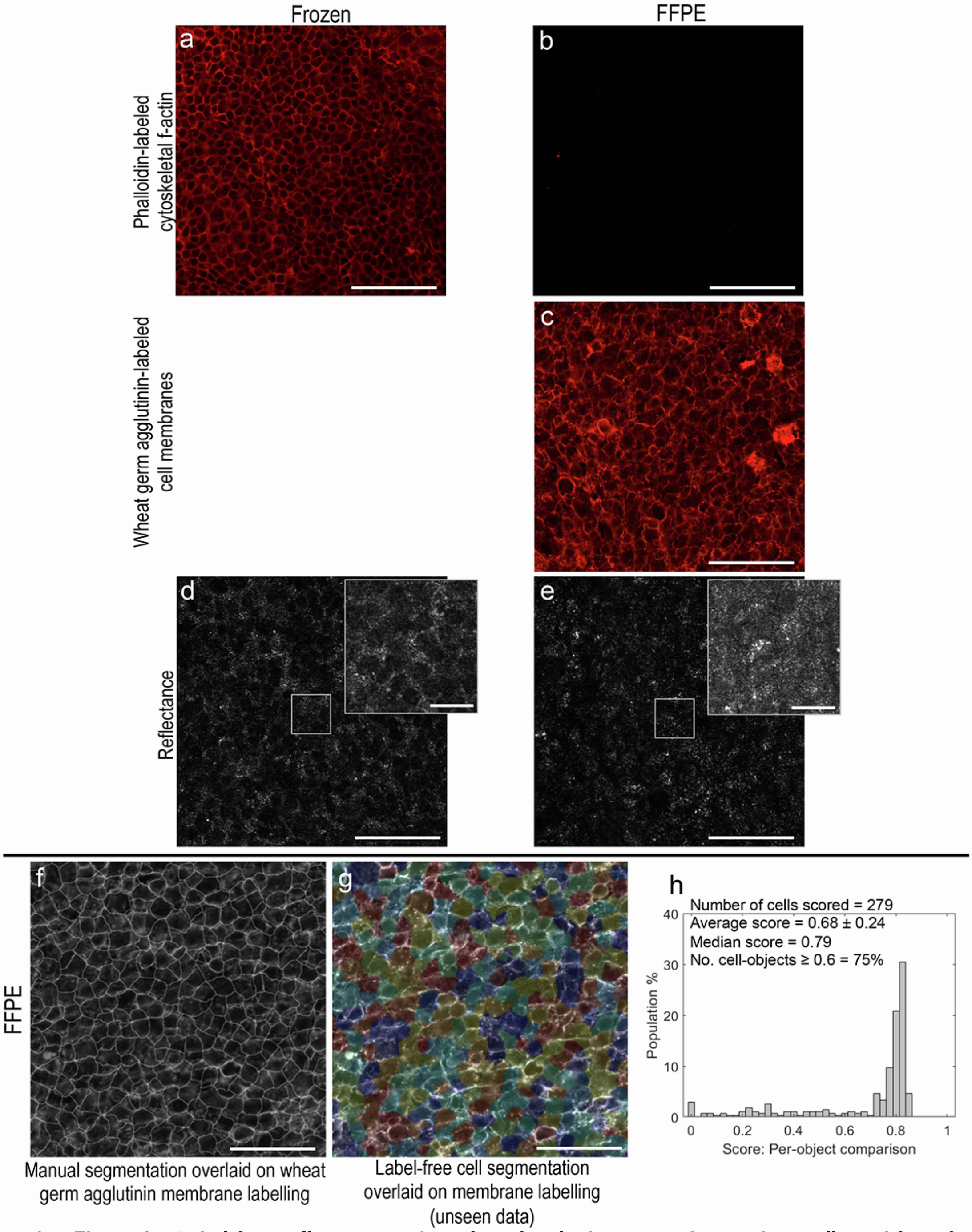

Figure S4 related to Figure 2 – Label-free cell segmentation of confocal microscopy image-data collected from formalin-fixed, paraffin embedded tissue sections. a, In frozen cryostat sections, f-actin staining using phalloidin conjugates clearly delineates cell outlines providing ground truth to enable the presented label-free cell segmentation approach. **b**, In contrast, in formalin-fixed paraffin embedded (FFPE) tissue sections, phalloidin staining fails because solvent exposure during the fixation and paraffin embedding process degrades the actin cytoskeleton. **c**, Demonstrated here using murine Peyer's patch tissue sections, successful ground truth labelling can be restored in the FFPE section-type by switching to cell membrane (*i.e.,* phospholipid) staining using wheat germ agglutinin (WGA) fluorescence conjugates. **d/e** Comparison of the reflectance signal from the frozen and FFPE section-types. Cytoskeletal degradation appears to change the reflectance images observed from the FFPE tissue: the faint trace of the cell outlines visible in the frozen sections is no longer apparent and instead the intracellular regions appear to exhibit the highest reflectance signal. **f-h** Despite this, a relationship between the reflectance signal and a WGA-delineated ground truth is still determinable by the neural network allowing (**g/h**) successful label-free cell segmentation direct from the reflectance signal. **h**, Intersection over union (IOU) score distribution comparing a (**f**) hand-drawn segmentation and the (**g**) automated, label-free cell segmentation outcome. An IOU score of 1 represents a perfect, per-pixel overlap between the hand-drawn and automated cell segmentations. Within the comparison presented here, scores ≥ 0.6 are seen to represent a good match, approaching the limits of hand-drawing accuracy. By harnessing ground truth from other fluorescence labels, the label-free strategy can operate in *both* FFPE and frozen tissue-types. Given that tissue archiving in FFPE format is commonplace worldwide, this finding dramatically increases the application domain of the presented label-free cell segmentation strategy. *Scale bars: a/b = 20 microns; c/d = 100 microns; e/f = 75 microns; g/h = 50 microns.*

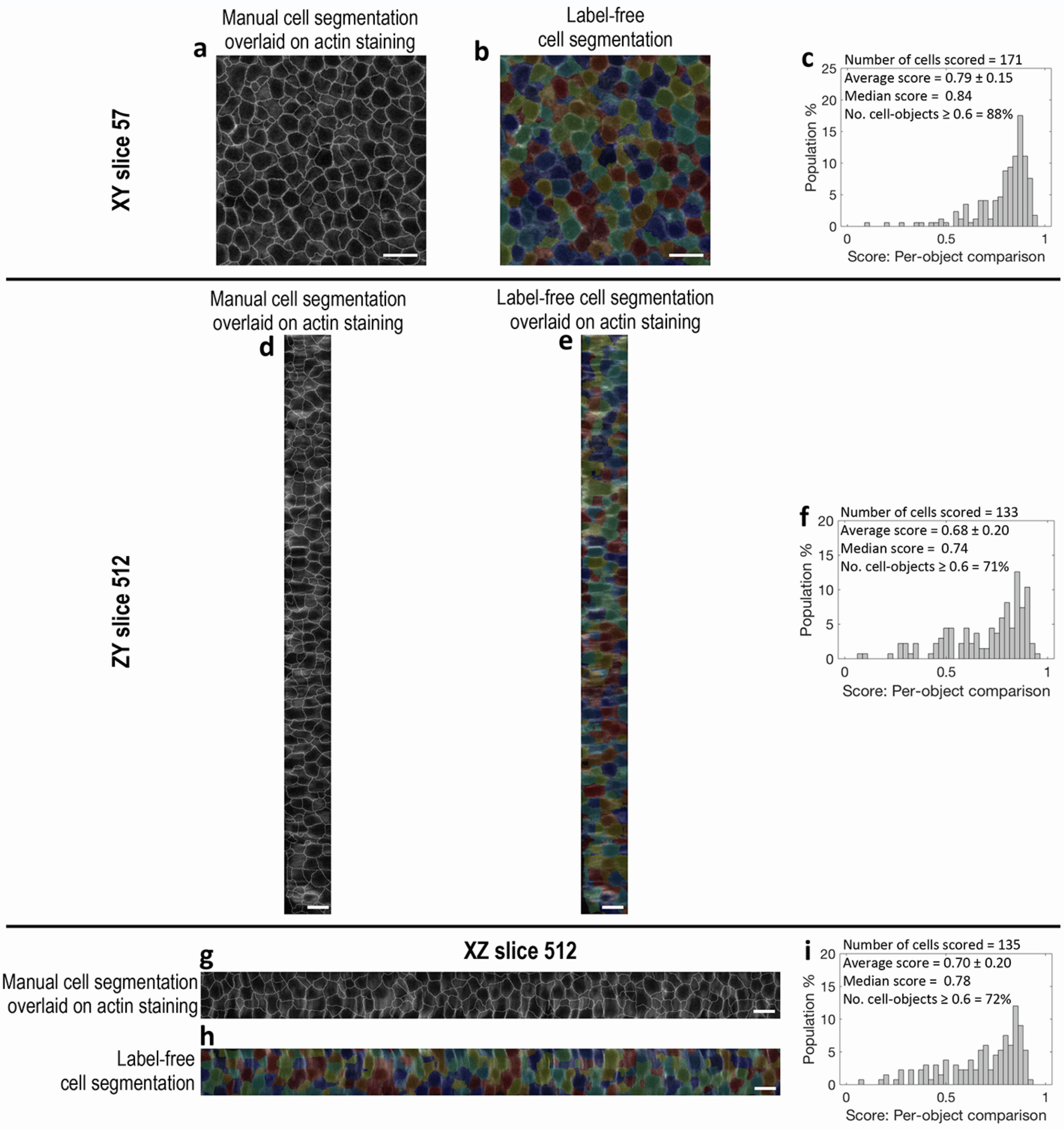

Figure S5 related to Figure 4 – Assessing 3-D label-free cell segmentation accuracies using mouse Peyer's patch tissue. a, Hand-drawn cell segmentations performed using the nuclei/actin fluorescence information for Z-planes (**a**) 57 in the XY dimension (**d**) 512 in ZY dimension and (**g**) 512 in the XZ dimension (unseen test image-data is 512x512x114 (X,Y,Z)). **b/e/h**, Automated cell segmentations for the same image-regions as (**a/d/g**) but achieved label-free direct from the reflectance signal. **c/f/i,** Cell-object intersection-over-union score distributions comparing – cell-object by cell-object – the (**a/d/g**) hand-drawn segmentations against the (**b/e/h**) automated, label-free cell segmentations. An IOU score of 1 represents perfect, per-pixel overlap between the hand-drawn and automated cell segmentations. Within the comparison presented here, scores ≥ 0.6 are seen to represent a good match, approaching the limits of hand-drawing accuracy. Encouragingly, the 3-D approach outperformed the segmentation accuracies achieved in 2-D (shown, **Figure 2**). *Scale bars equal 20 microns.*

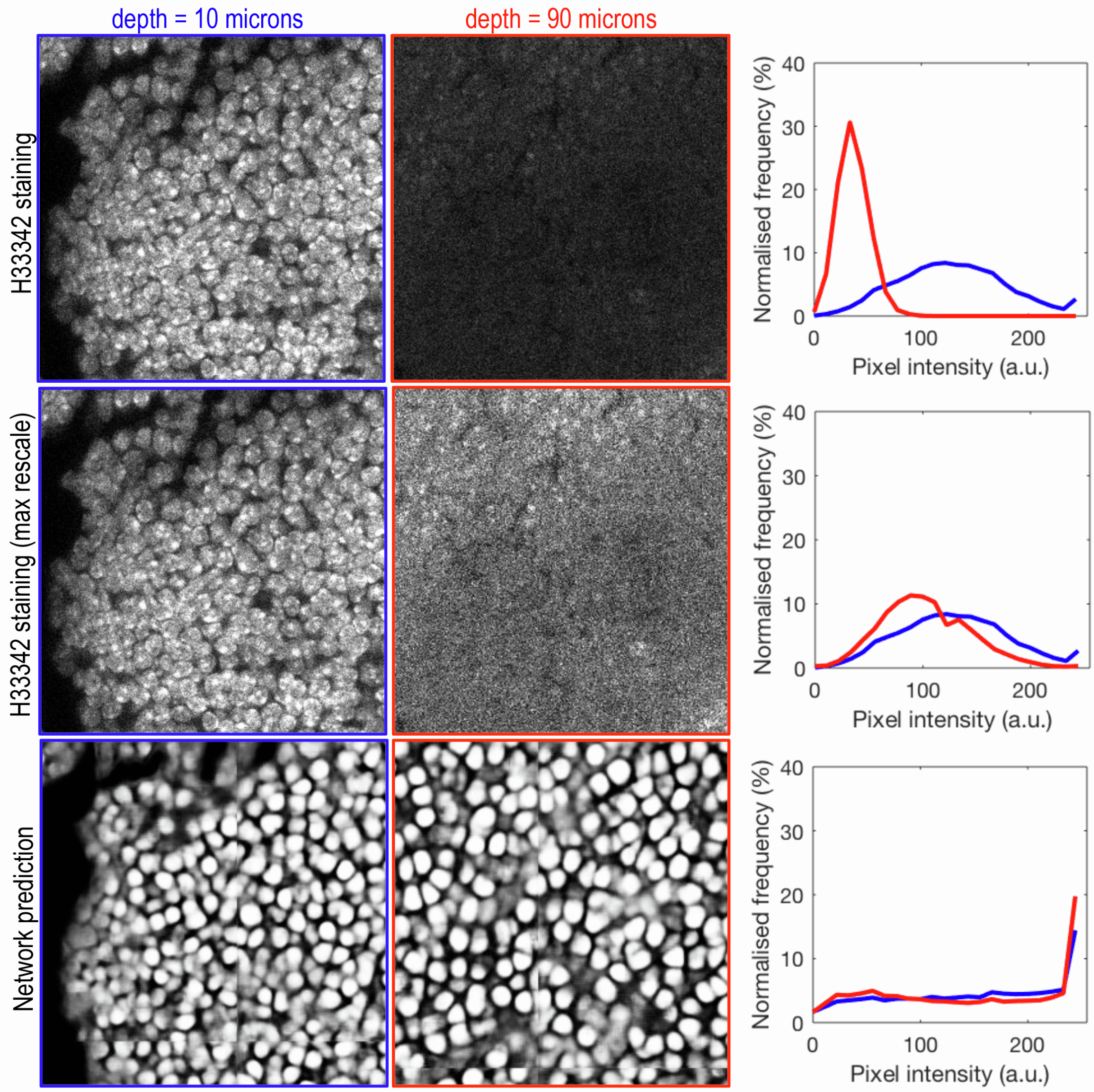

Figure S6 related to Figure 4 – Fluorescence versus label-free nuclei predictions at Z-depths of ~10 and ~90 microns. Using reflectance information from a 638 nm excitation laser, the 3-D network is able to consistently recover nuclear information long after the blue nuclear stain (Hoechst 33342) has decayed from multiple scattering effects (bottom right versus middle right). The resultant pixel intensity histograms from the probability map images are extremely stable (bottom right). This is advantageous for achieving consistent, depth-invariant 3-D cell segmentation in thick tissue specimens.

Table S1 related to Star Methods – Antibody and Image Information Table

PRIMARY ANTIBODIES	Product no	Supplier	Dilution primary	Stock concentration	Host	Secondary (detailed below)	Detection	Figure
Anti-mouse CD3-EF450	48-0032-82	Thermo Fisher	1:25	0.2 mg/mL	Rat	N/A	eFluor 450	Figure 1
Anti-mouse CD4-PE	12-0041-83	Thermo Fisher	1:25	0.2 mg/mL	Rat	N/A	R-phycoerythrin	Figure 1
Anti-mouse CD11c-EF660	50-0114-82	Thermo Fisher	1:25	0.2 mg/mL	Armenian Hamster	N/A	eFluor 660	Figure 1
Anti-mouse FOXP3-EF660	50-5773-82	Thermo Fisher	1:25	0.2 mg/mL	Rat	N/A	eFluor 660	Figure 2
Anti-mouse CD3	AB5690	Abcam	1:150	0.2 mg/mL	Rabbit	Anti-Rabbit AF568	AlexaFluor 568	Figure 3
Anti-mouse CD11c	AB33483	Abcam	1:400	0.5 mg/mL	Armenian Hamster	Anti-Hamster A488	AlexaFluor 488	Figure 3

SECONDARY ANTIBODIES	Product no	Supplier	Stock concentration	Host	Secondary dilution	Fluorophore
Goat anti-Rabbit IgG (H+L)	A-11011	Thermo Fisher	2 mg/mL	Goat	1:400	AlexaFluor568
Goat anti-Hamster IgG (H+L)	A-11008	Thermo Fisher	2 mg/mL	Goat	1:400	AlexaFluor488

**Figure ID**	**Section type**	**Tissue type** (Sectioning orientation)	**Objective lens** Magnification / Numerical aperture (Microscope)	**Pixel density** Pixels per micron	**Voxel size** x,y,z µm	**Reflectance** Excitation laser (Detector placement) Wavelength, nm	**Train / Test image(s)** (number) x/y/z dimensions Pixel dimensions	**Patch size / (patches per epoch)**	**Approx. Training time (single NVIDIA 1080 Ti GPU)**
Figure 1	Cryostat (frozen)	Mouse spleen (transverse)	40X/1.3 (Leica SP8 inverted)	5.2842	0.1892 x 0.1892	488 (485-491)	Train: (1) 7617 x 7661<br>Test: (1) 5766 x 5787	256x256 (1000)	268 min (50 epochs)
Figure 4	Cryostat (frozen)	Mouse mesenteric lymph node (transverse)	40X/1.3 (Zeiss LSM780 upright)	4.8177	0.2076 x 0.2076 x 0.1500	561 (558-564)	Train: (2) 1024 x 1024 x 107<br>Test: (1) 1024 x 1024 x 111	64x64x64 (1000)	750 min (150 epochs)
Figure S1 Figure S6	Cryostat (frozen)	Mouse ileal Peyer's patch	40X/1.3 (Leica SP8 inverted)	3.5200	0.2840 x 0.2840 x 0.346	488 (485-491)<br>638 (635-641)	Train: (3) 512 x 512 x 103<br>Test: (1) 512 x 512 x 103	64x64x64 (500)	320 min (150 epochs)
Figure 3 Video S1	Cryostat (frozen)	Mouse ileal Peyer's patch (transverse)	63X/1.4 (Leica SP8 inverted)	8.324	0.1201 x 0.1201	488 (485-491)	Train: (1) 7607 x 11253<br>Test: (1) 7610 x 11247	256x256 (1500)	360 min (50 epochs)
Figure S4	FFPE	Mouse ileal Peyer's patch (transverse)	40X/1.3 (Leica SP8 inverted)	7.0463	0.1419 x 0.1419	638 (635-641)	Train: (1) 11364 x 11421<br>Test: (1) 7641 x 7660	256x256 (2000)	502 min (50 epochs)

Methods S1 – Related to Star Methods

Table of contents –

Pages 2-5:	Setting up reflectance imaging on Leica and Zeiss confocal microscopes
Pages 6-12:	Label-free probability map generation using standalone Windows software
Pages 13-21:	Running the label-free cell segmentation deep learning scripts using MATLAB
Pages 22-29:	MATLAB code: 2-D pipeline
Pages 30-39:	MATLAB code: 3-D pipeline
Pages 40-58:	Running the label-free cell segmentation deep learning scripts using Python
Pages 59-68:	Python code: 2-D pipeline
Pages 69-83:	Python code: 3-D pipeline
Pages 84-89:	Extracting single-cell features using CellProfiler 4
Pages 90-119:	2-D CellProfiler pipeline
Pages 120-139:	3-D CellProfiler pipeline

Setting up sequential reflectance imaging using a standard Zeiss LSM780 confocal microscope.

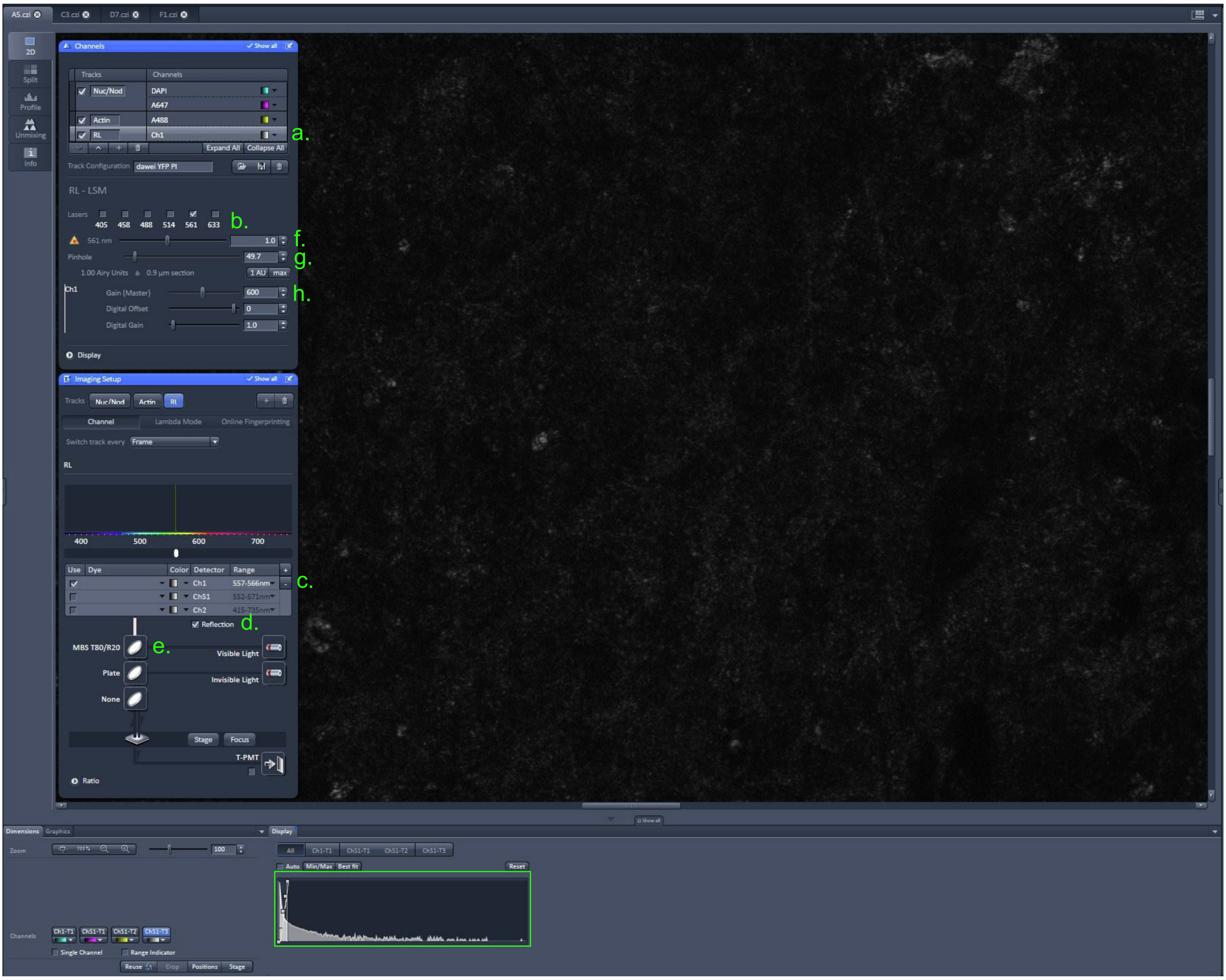

a, Once the fluorescence imaging sequences are set up, a new track for reflectance is added to the sequential scan. **b**, The desired excitation laser for reflectance imaging is selected (here, 561 nm). The choice of laser is not particularly important, but use of longer wavelengths may reduce fluorophore photobleaching and improve penetrance / recovery in thicker specimens (shown, **Figures S1/S6**). **c**, A photomultiplier detector (here Ch1) is turned on and the range set to approximately +/- 3 nm either side of the excitation wavelength (here 558 – 564 nm was entered, but the software rounds to display ~ 557 – 566 nm). **d**, The tick-box allowing reflected light to pass to the detector is turned on. **e**, The T80/R20 beam splitter is chosen (this indicates a transmission/reflection ratio of 80:20). **f**, A low laser excitation power (here, 1%) is entered. *N.B.,* use of a reflectance light path with high laser excitation power may damage the camera, so care should be taken here. The pinhole is set to ~ 1 airy unit, yielding an optical section of around ~ 1 micron with a high numerical aperture 40X or 63X objective. **g**, Running in ‘live mode’, the gain is slowly increased until the reflectance signal occupies approximately 80% of the range histogram (indicated, green box). Compressing the histogram in the range indicated yields a typical ‘view’ of the reflectance signal from a lymphoid tissue specimen (on display in the main image window).

Setting up sequential reflectance imaging using a standard Leica SP8 confocal microscope.

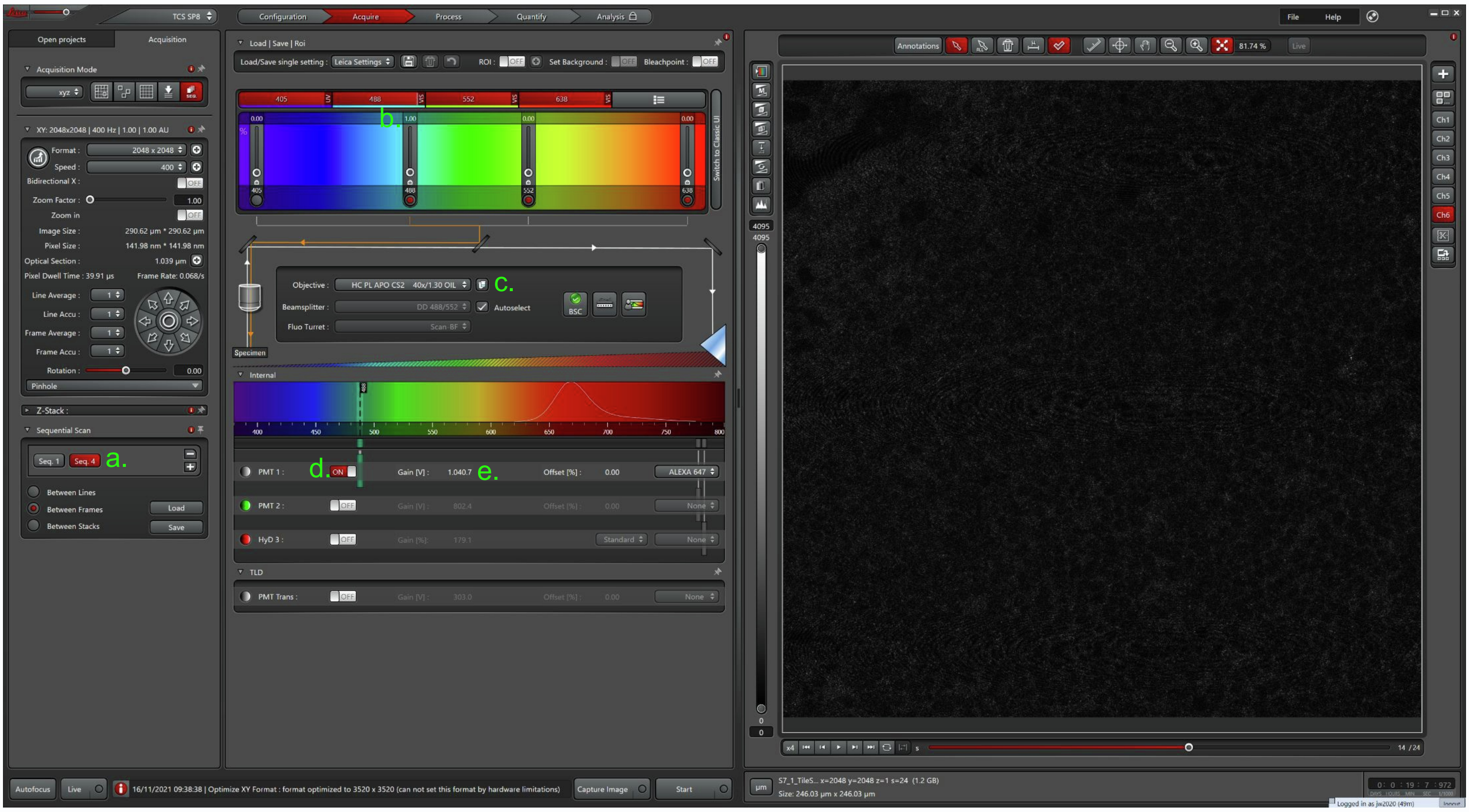

a, Once the fluorescence imaging sequence(s) are set up, a new track for reflectance is added to the sequential scan (here, 'Seq 4'). **b**, The desired excitation laser for reflectance imaging is turned on (here, 488 nm). The choice of laser is not particularly important, but use of longer wavelengths may reduce fluorophore photobleaching and improve penetrance / recovery in thicker specimens (shown, **Figures S1/S6**). A low laser excitation power (*e.g.,* 1%) is also specified at this step. *N.B.,* Use of a reflectance light path with high laser excitation powers could damage the camera, so care should be taken at this step. **c**, An appropriate beam splitter is chosen for the excitation line, or, the 'Autoselect' checkbox can be ticked to set this automatically. **d**, A photomultiplier detector (here PMT1) is turned on and the range set to approximately +/- 3 nm either side of the excitation wavelength (*i.e.,* here, 485-491 nm). **e**, Running in 'live mode', the gain is slowly increased until the reflectance signal occupies approximately 80% of the available intensity range. The main window shows a typical 'view' of the reflectance signal from a lymphoid tissue specimen under this setup.

Setting up simultaneous reflectance and fluorescence imaging using a standard Leica SP8 confocal microscope.

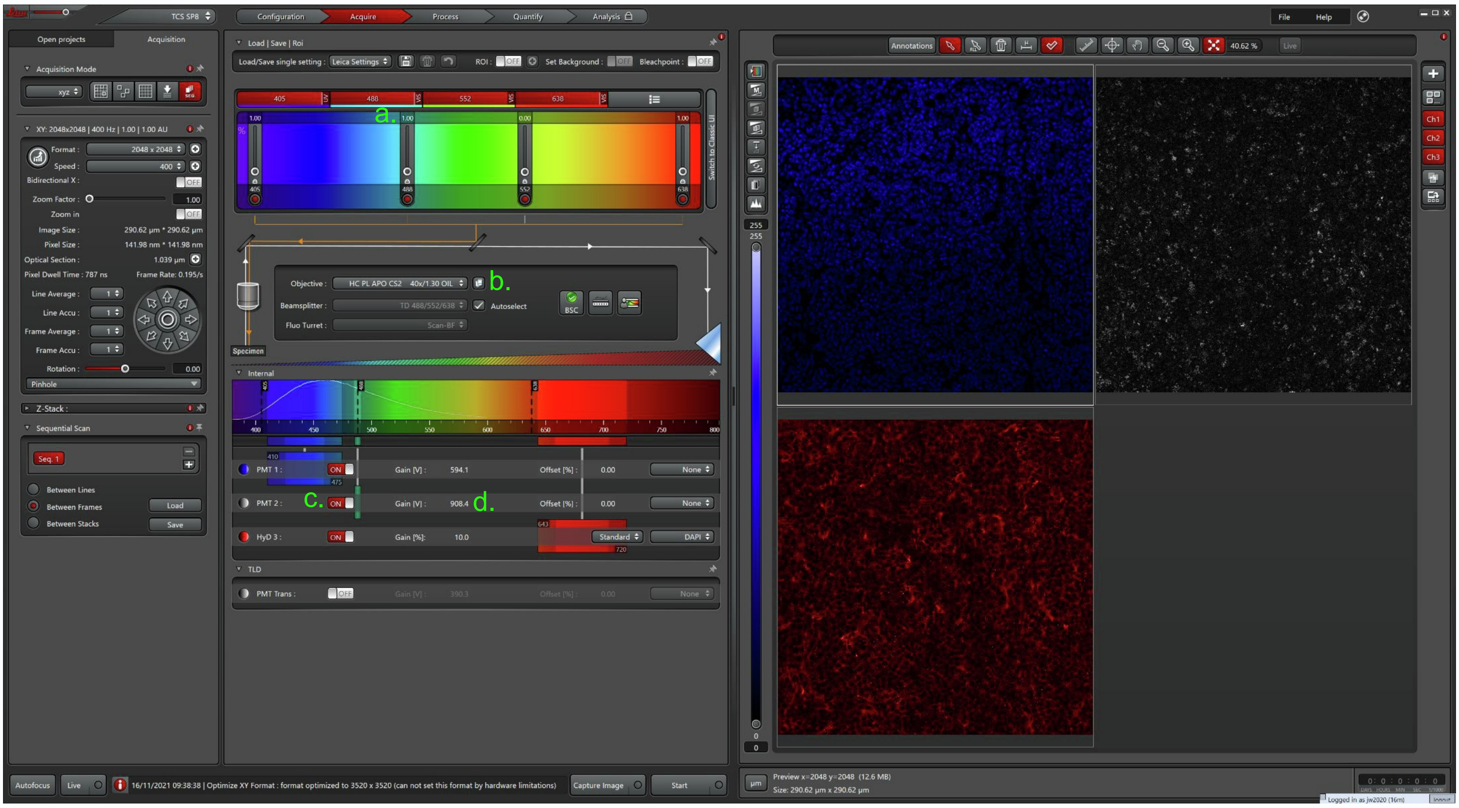

Once fluorescence excitation and collection are configured, (**a**) any remaining laser line can be used for reflectance imaging (*e.g.*, here, the 488 nm line is used). The choice of laser is not particularly important, but use of longer wavelengths may reduce fluorophore photobleaching and improve penetrance / recovery in thicker specimens (shown, **Figures S1/S6**). A low laser excitation power (*e.g.*, 1%) should also be specified here. Reflectance imaging with high laser excitation powers could damage the camera, so care should be taken at this step. **b**, An appropriate beam splitter for the excitation lasers is chosen, or the 'Autoselect' checkbox ticked to enable automatic setting. **c**, A free detector (here PMT2) is turned on and the range set to approximately +/- 3 nm either side of the excitation wavelength (*i.e.*, 485-491 nm). **d**, Running in 'live mode', the gain is slowly increased until the reflectance signal occupies approximately 80% of the available intensity range. The top-right image in the main window shows a typical 'view' of the reflectance signal from a lymphoid tissue specimen under this setup. This approach allows reflectance data to be concomitantly collected alongside fluorescence without adding the additional run-time of further sequences. *N.B.*, It is worth noting that in a similar way, a laser that is already being used for fluorescence excitation may also be used to obtain reflectance data (exemplified **page below**). For example, here, PMT2 could be moved up to collect reflectance from the 638 nm laser in the range 635-641 nm. Doing this has the advantage of reducing the photon budget for the sample. However, it also necessitates that enough excitation power is being used to obtain a good reflectance signal, and that a free detector can be moved within the necessary detection range. This is not always compatible with optimal fluorescence imaging – hence the setup shown here.

Setting up ‘free’ reflectance imaging alongside fluorescence collection using a standard Leica SP8 confocal microscope.

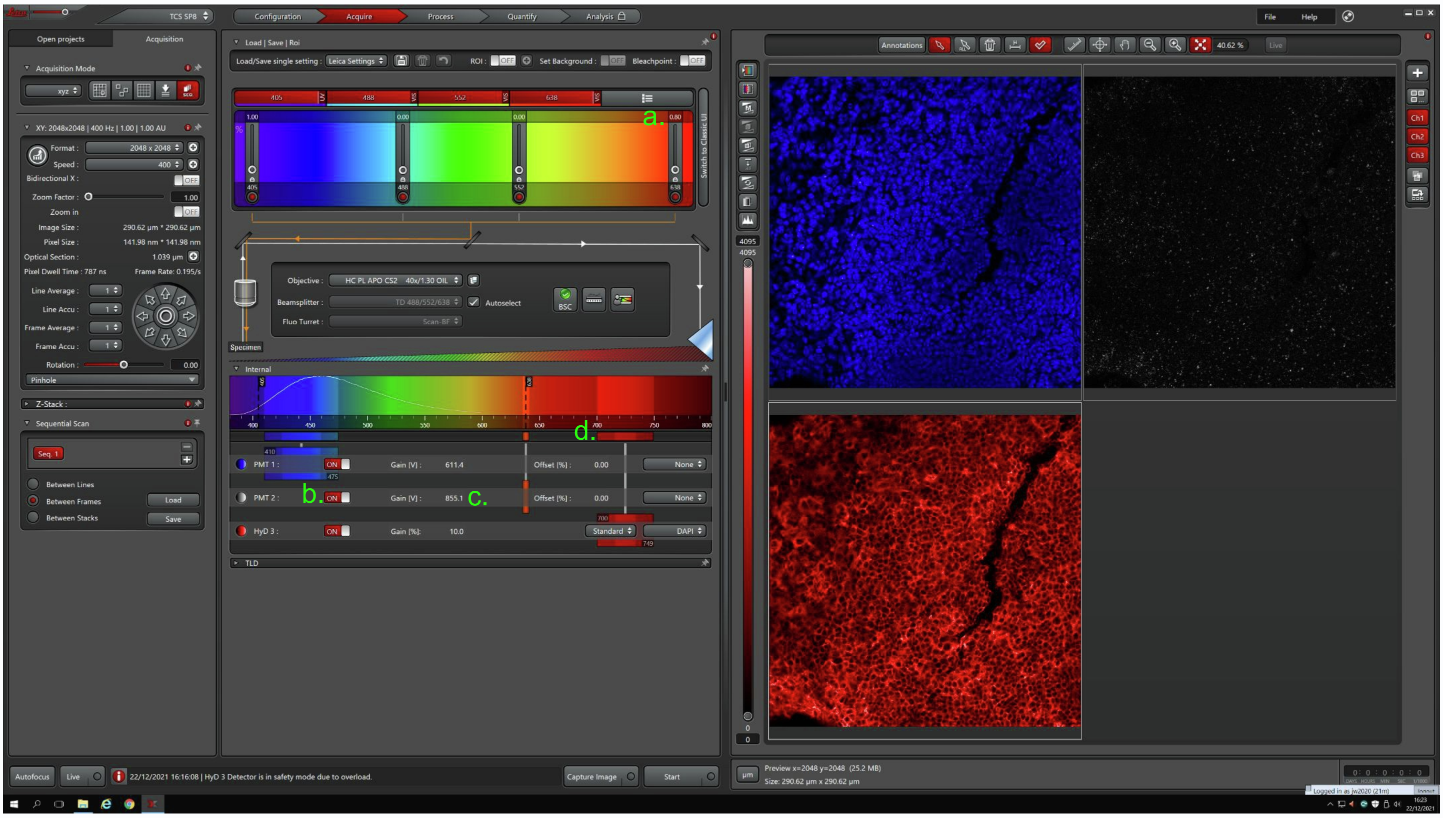

Once fluorescence excitation and collection are configured, (**a**) any remaining detector can be used to simultaneously collect the reflectance signal from one of the excitation lasers being used to stimulate fluorescence (*e.g.,* here, ‘PMT2’ is used to collect reflectance from the 638 nm laser line (**b**) – which is also being used to excite AlexaFluor 647). This is achieved by placing the detector approximately +/- 3 nm either side of the excitation wavelength (*i.e.,* 635-641 nm). **c**, Running in ‘live mode’, the gain is slowly increased until the reflectance signal occupies approximately 80% of the available intensity range. The top-right image in the main window shows a typical ‘view’ of the reflectance signal from a lymphoid tissue specimen under this setup. Simultaneous reflectance imaging has the advantage of reducing the photon budget for the sample, as the reflectance information is effectively recovered for ‘free’ by harnessing scatter from a laser that is already in use. However, this setup also necessitates that enough excitation power is available to obtain a good reflectance signal, and that a free detector can be moved into the necessary detection range. To achieve this here without saturating the AlexaFluor 647 signal (shown bottom-left in the main image window) the AlexaFluor647 detection range was narrowed (**d**) to ~ 700-750 nm. Where this setup cannot be accommodated one of the other options that instead use a dedicated laser for reflectance imaging should be utilised (shown, **three above pages).**

Windows 10: Running the label-free cell segmentation pipeline using standalone software

Running this software requires:

- Windows 10 machine (NVIDIA GPU desirable for model training)
- Label-free stain prediction standalone software (BioStudies download)
- MATLAB runtime 9.11 (installs automatically alongside software – see below).

Note: This software is free to install. No MATLAB license is required to run this standalone software.

Installation Steps:

1. **Install the Label Free Stain Prediction software** by running the Windows 10 installer from the BioStudies download

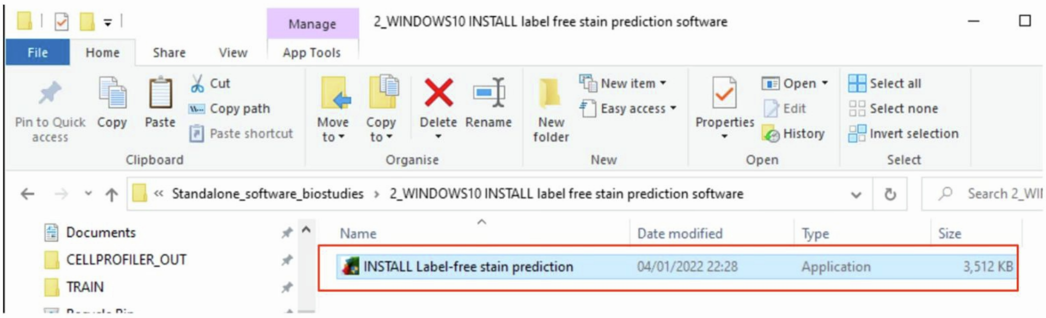

2. Follow through the setup procedure to install the software:

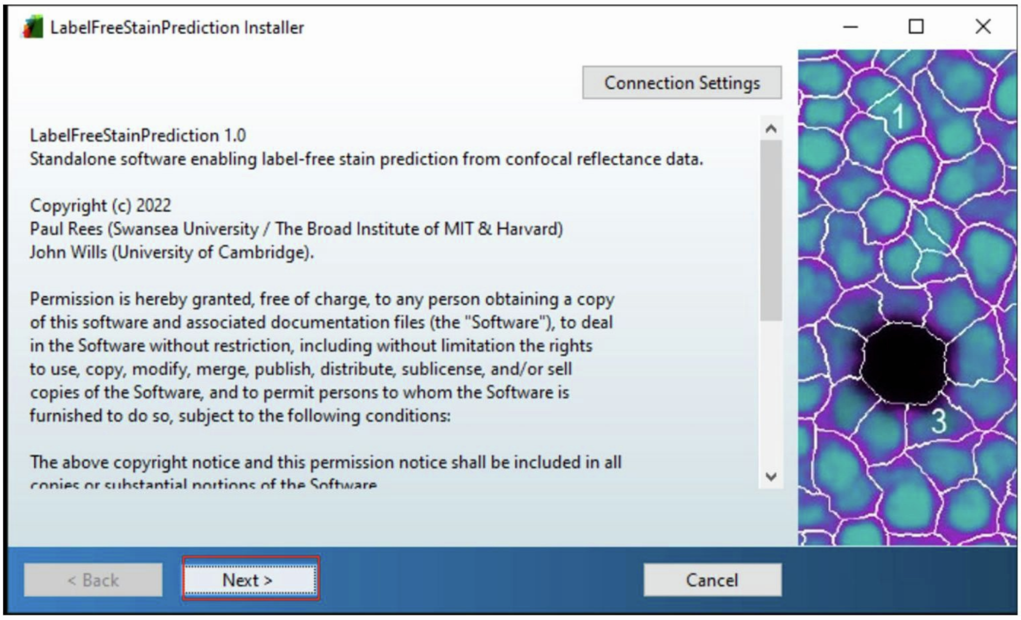

- Check 'add shortcut to the desktop':

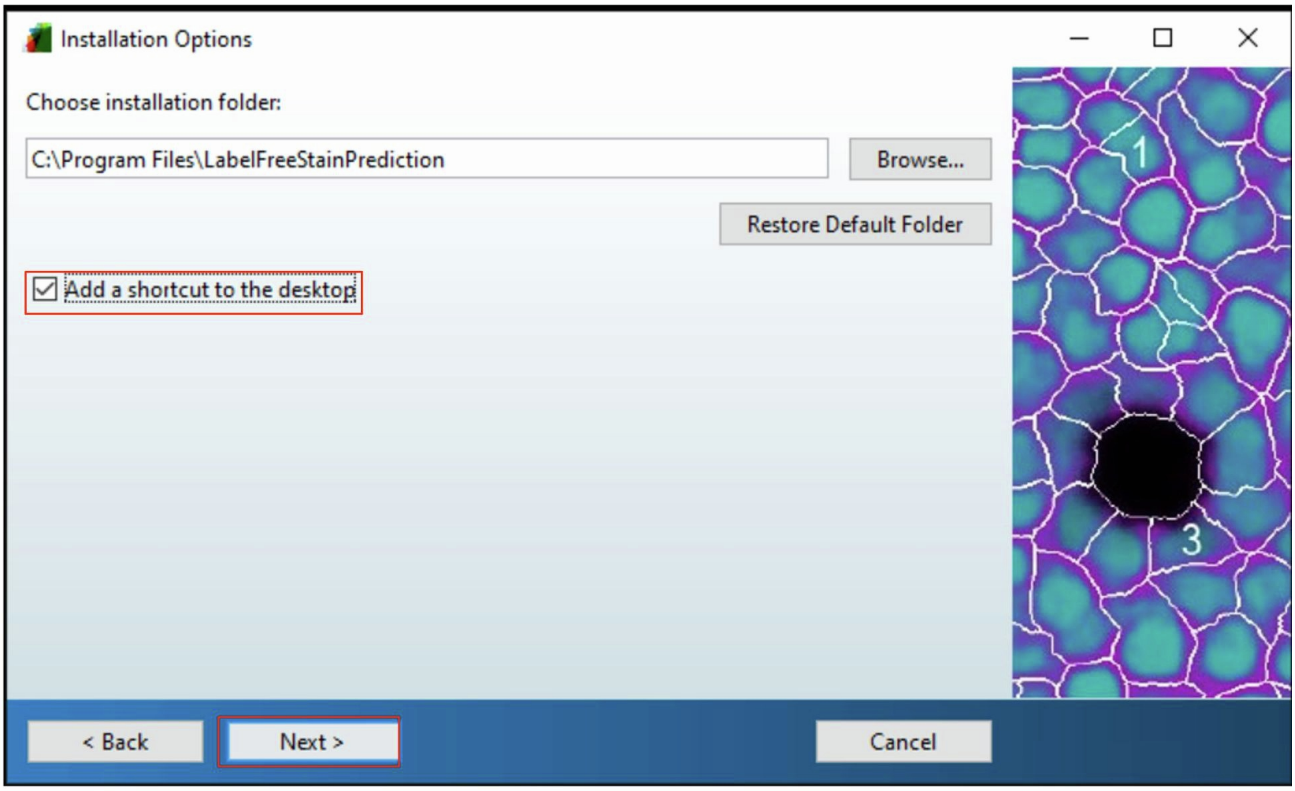

- When prompted, install MATLAB runtime (free; no license is required):

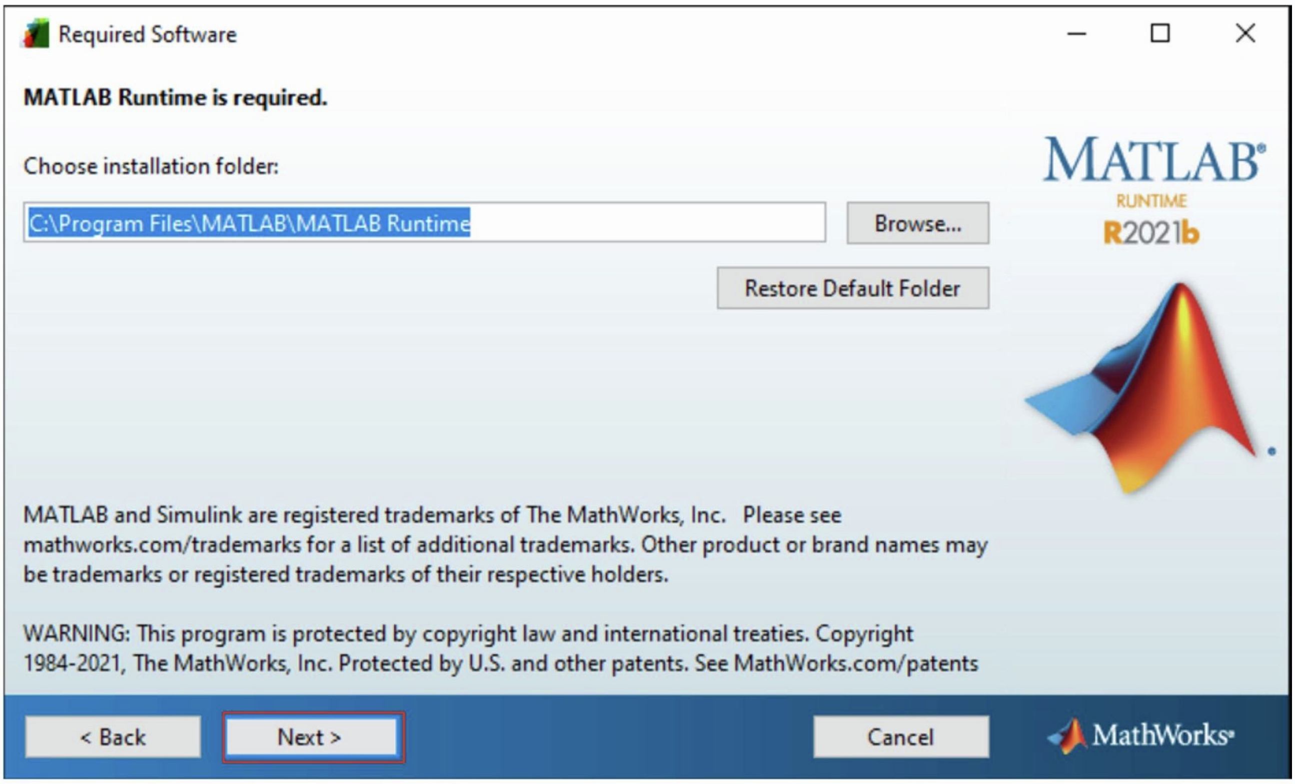

- click ‘install’ to install the label free stain prediction software and MATLAB runtime 9.11

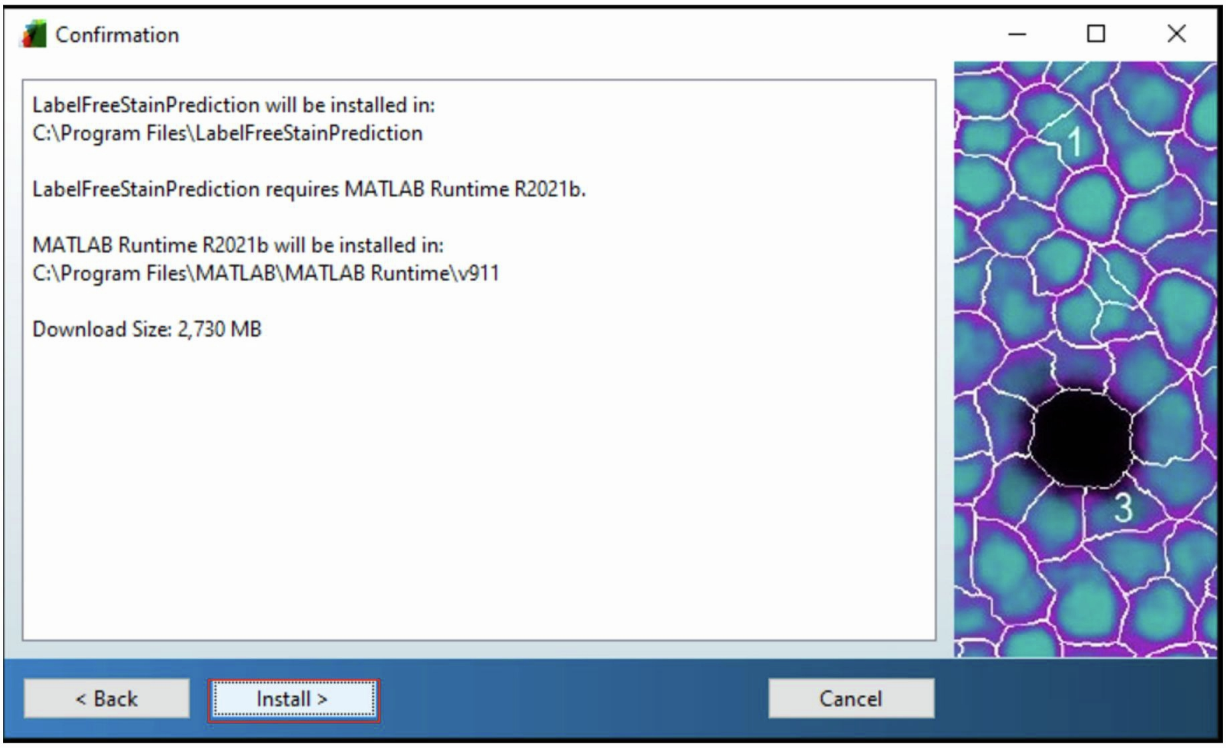

3. Training 2-D or 3-D UNET model using the standalone software

-A screencast video is included with the BioStudies archive.

-Launch the Label Free Stain Prediction software from the desktop icon

-Example 2-D and 3-D image data and training labels are included in the BioStudies download.

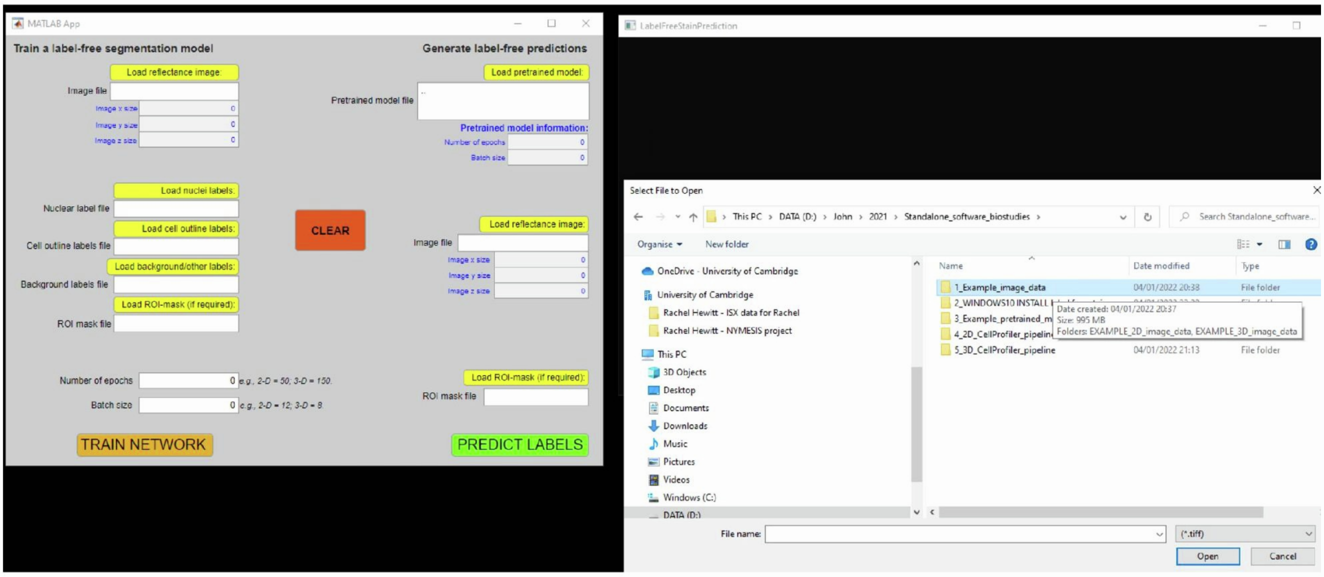

- To Train a 2-D or 3-D UNET model to predict the probability images needed to enable the label-free segmentation, load reflectance data and matching labels for 'Nuclei', 'Cell Outlines' (e.g. actin) and 'background/other' classes.
-The label images should be in .tiff format with the stains represented as foreground (i.e., white).
-2-D or 3-D data will be automatically detected.

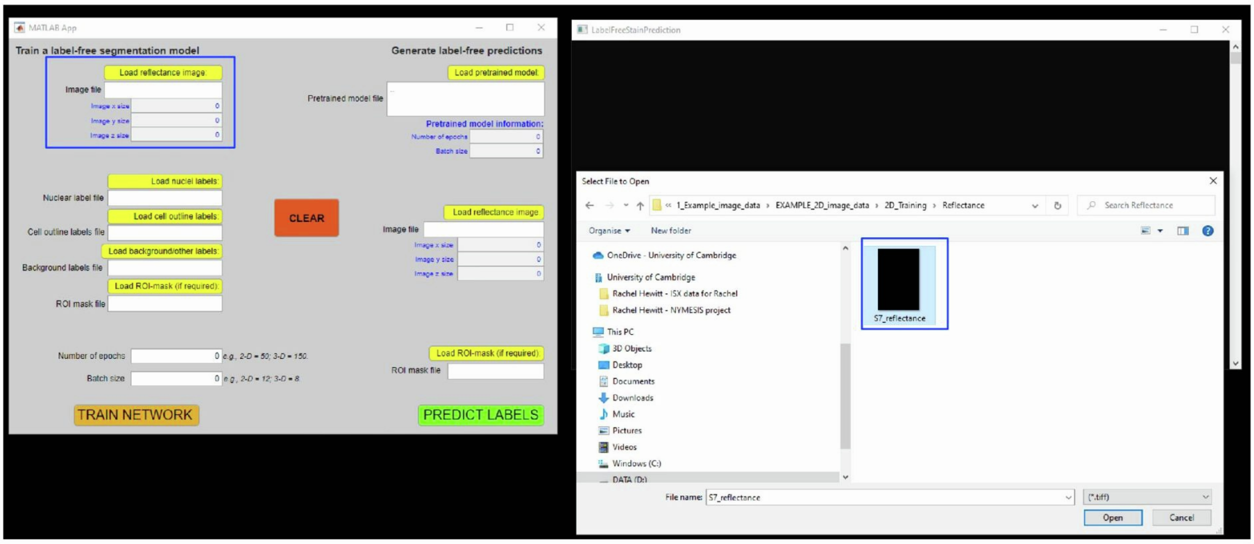

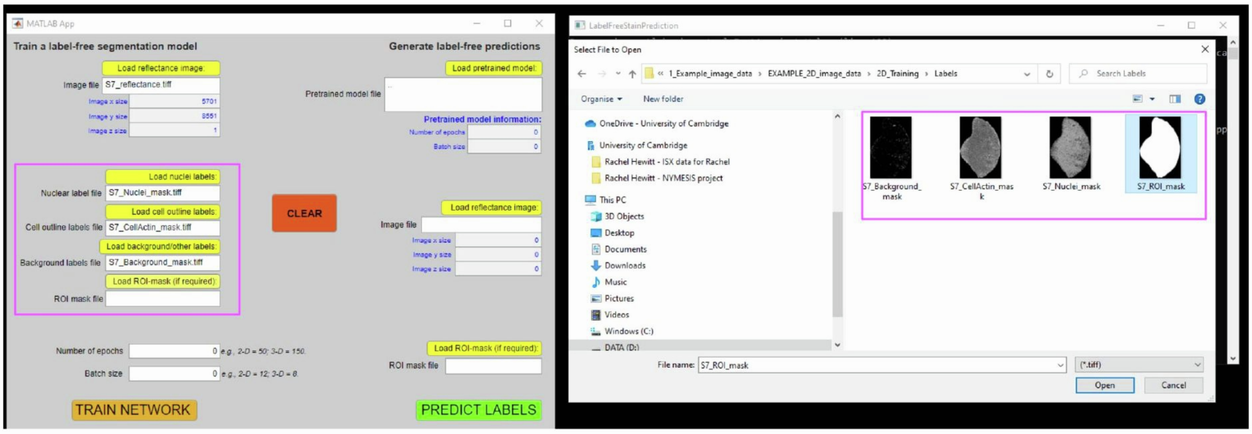

- If desired, a ROI-mask can also be loaded to mask the outputted probability maps to the desired tissue region.
- If this isn't needed – just leave this box blank.

- Set the desired batch-size and number of training epochs and click 'Train Network'.
- After a few seconds, the loaded reflectance data and labels will be indicated in "Figure 1"
- Progress can be tracked in the Command Prompt console
- MAT formatted data and the trained model will be saved to folders on the desktop once training completes:
- Training with the example data takes ~6h on an NVIDIA 1080 GTX GPU. (Previously trained models are provided).

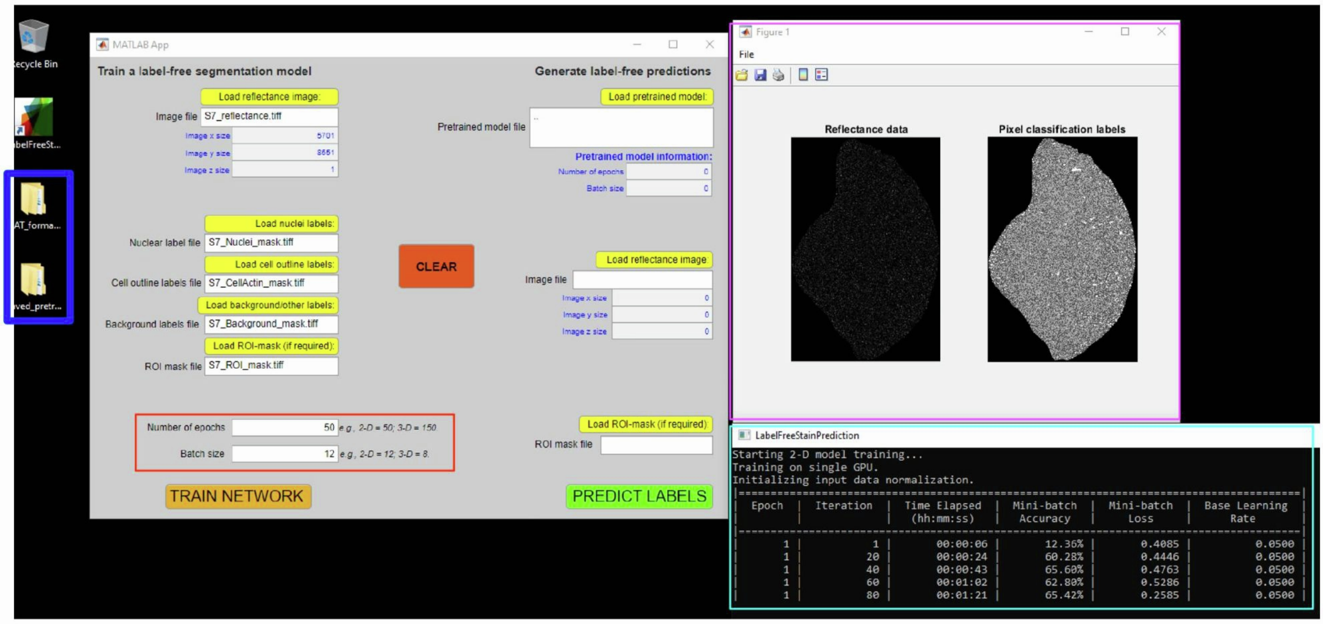

4. Using a pretrained 2-D or 3-D UNET model to generate probability images for label-free segmentation using unseen reflectance data.

-On the right-side of the software, load a pretrained model.

-This can be from Step 3 (above), or, example pretrained networks are provided in 'Folder 3' of the BioStudies download. Example unseen 'test' image-data is also provided in 'Folder 1' in 2-D and 3-D.

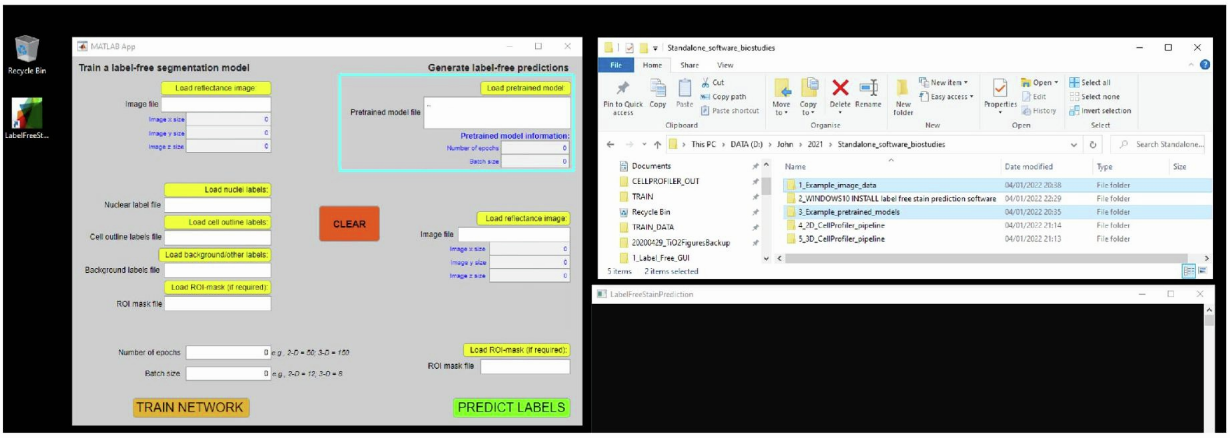

- Load 2-D or 3-D reflectance data:

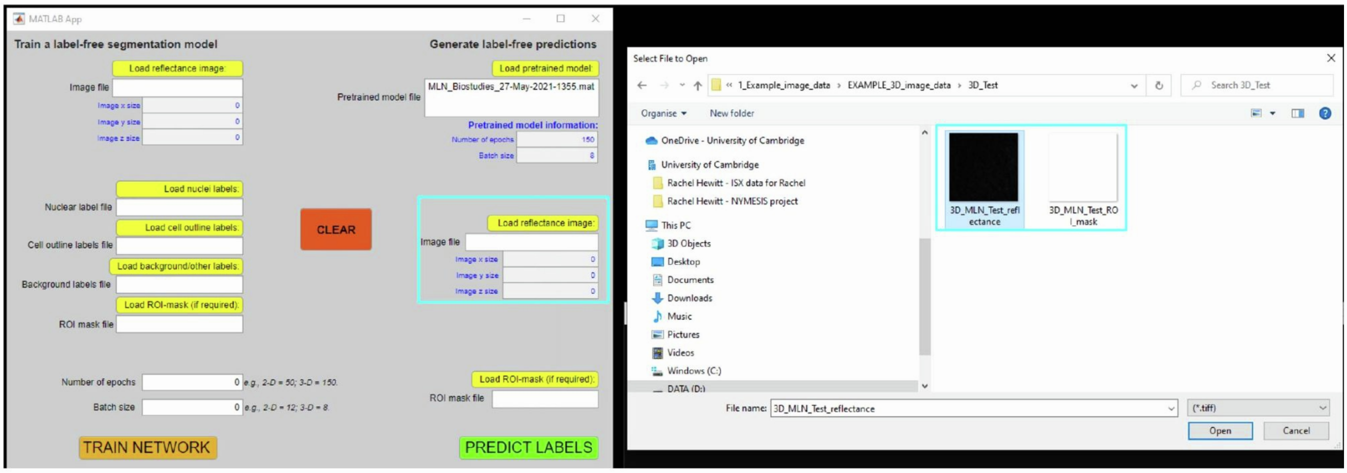

- If desired, load a ROI-mask to limit the probability maps to the desired tissue region:
- (Leave blank if not required).

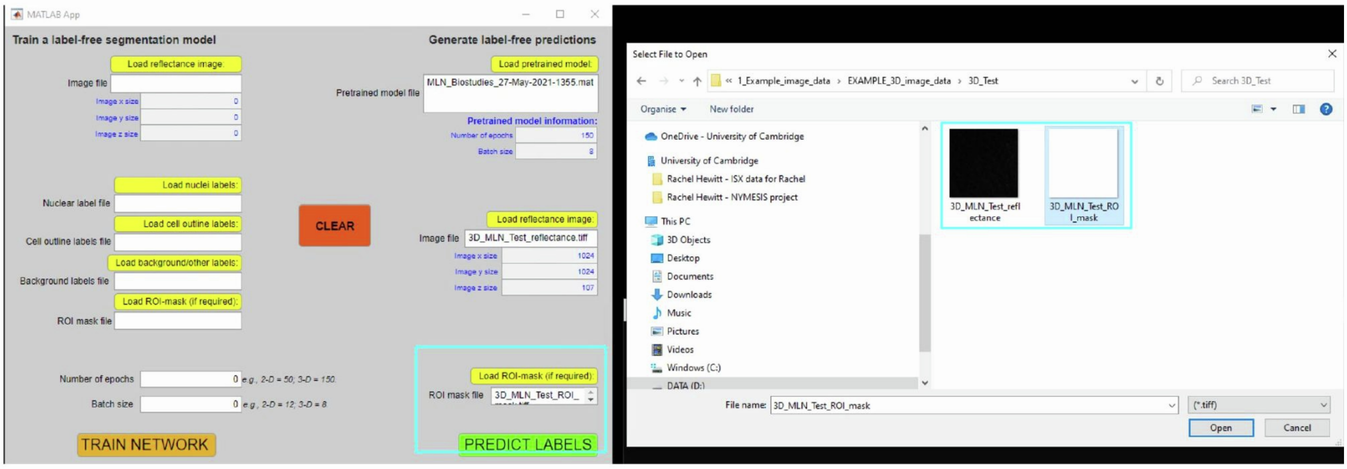

- Click 'Predict Labels' to generate the label-free probability images
- Progress can be tracked in the Command Prompt console
- Figure 1 demonstrates probability images for each class (central z-plane for 3-D data)
- Outputs are saved to the Desktop ready for loading into the CellProfiler pipelines enabling cell feature extraction (described below).

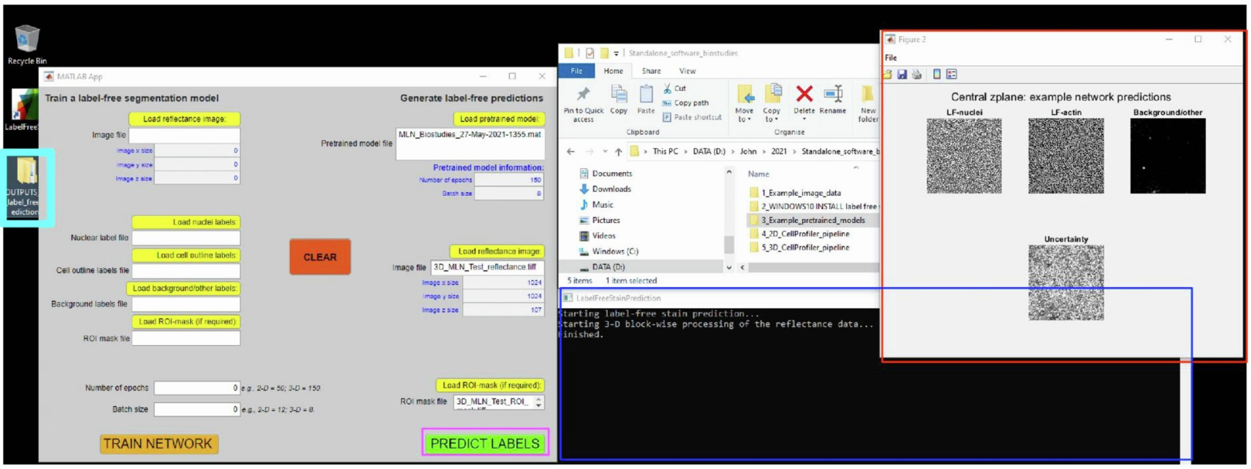

Windows 10: Running the label-free cell segmentation deep learning scripts on an NVIDIA GPU using MATLAB R2021b and the Deep Learning Toolbox

Running these deep learning scripts in MATLAB requires:

- MATLAB (version R2019a or later)
- Deep Learning Toolbox
- Image Processing Toolbox
- Computer Vision Toolbox

Installation Steps:
1. Install MATLAB
https://ch.mathworks.com/downloads/web_downloads
- Login to your MathWorks account to access the downloads page at the link above.
- Download and run the installer for MATLAB R2021b.

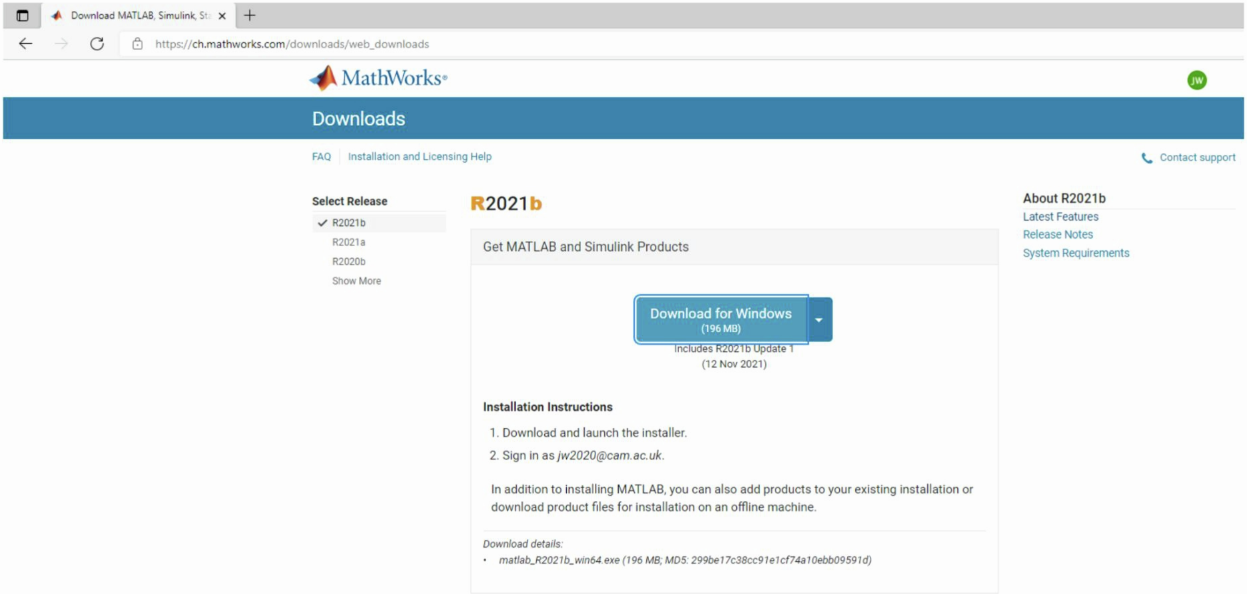

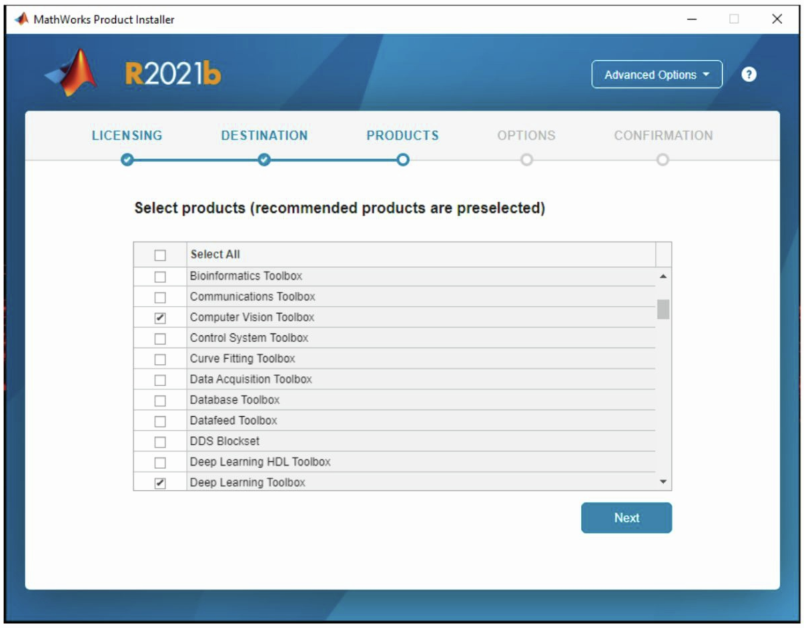

- During installation, install the Computer Vision, Deep Learning and Image Processing Toolboxes by selecting them at the 'Products' step of the installation.

- Once the installation is complete, you can proceed to running the scripts.

2. Training a 2-D UNET Model
-A screencast video is included with the BioStudies archive.
- Download and unzip the MATLAB BioStudies project archive at a suitable location on your computer.

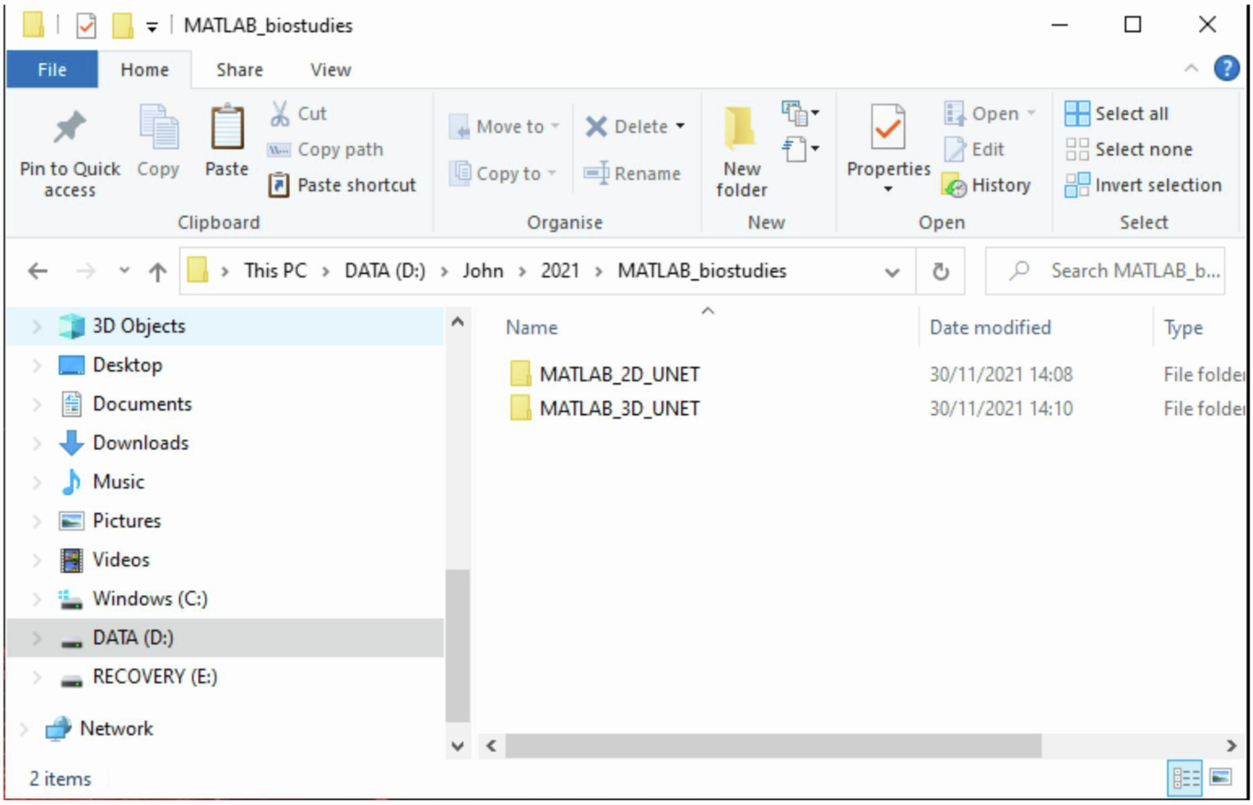

- Inside the MATLAB_2D_UNET folder, there is a sequentially-organised workflow starting with raw data and ending with a CellProfiler pipeline that obtains cell features from the label-free cell segmentation:

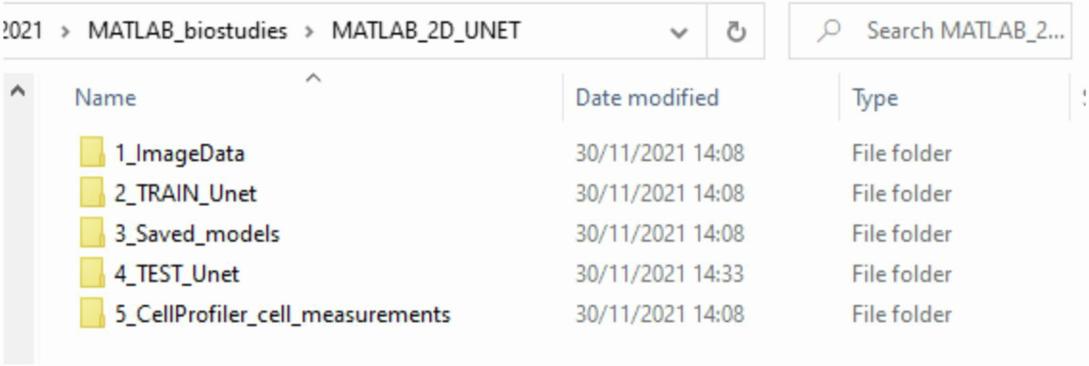

- To train a 2-D UNET model, open MATLAB and set the working directory to the “2_TRAIN_Unet” directory. Open the “A_TRAIN_UNET” script.

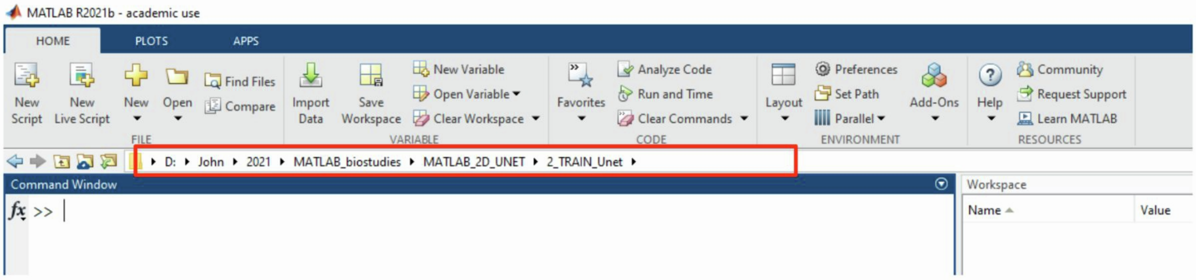

- Update the path to the training data (located in the “1_ImageData” folder).
- Update the path to tissue ROI mask which isolates the lymphoid follicle from the surrounding tissue (located in the “1_ImageData” folder).
- Update the path to the pixel classification labels (located in the “1_ImageData” folder)

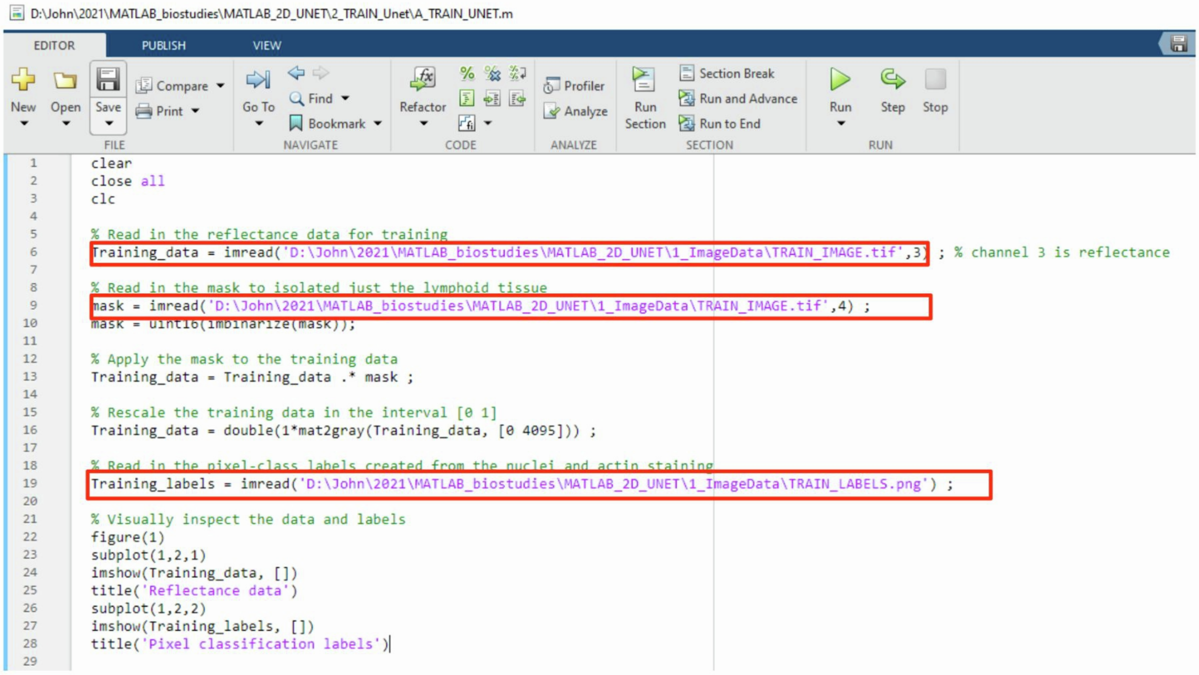

- On Line 46, update the path to the pixel classification labels (located in the “1_ImageData” folder) used by the “pixelLabelDatastore” function.

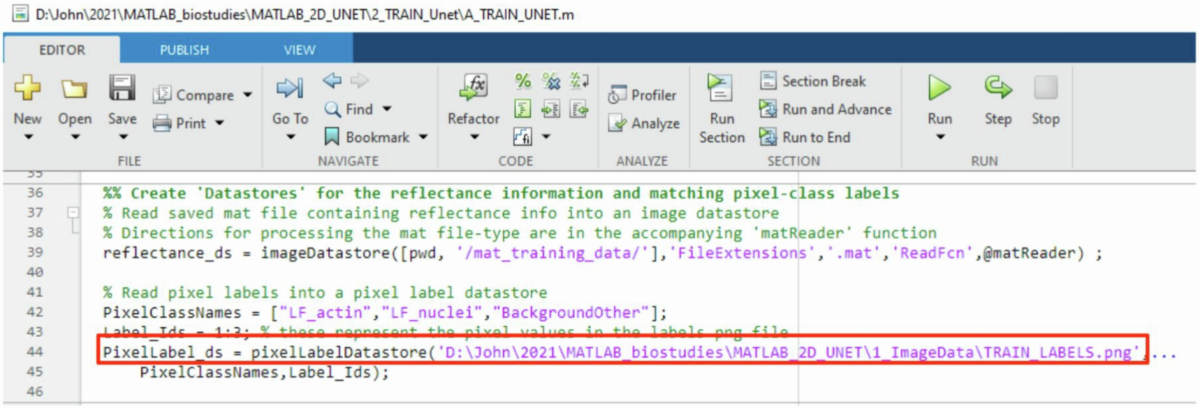

- At the bottom of the script on Line 195, update the path to the saved_network_directory.
- This should be folder 3 of the workflow ("3_Saved_models").

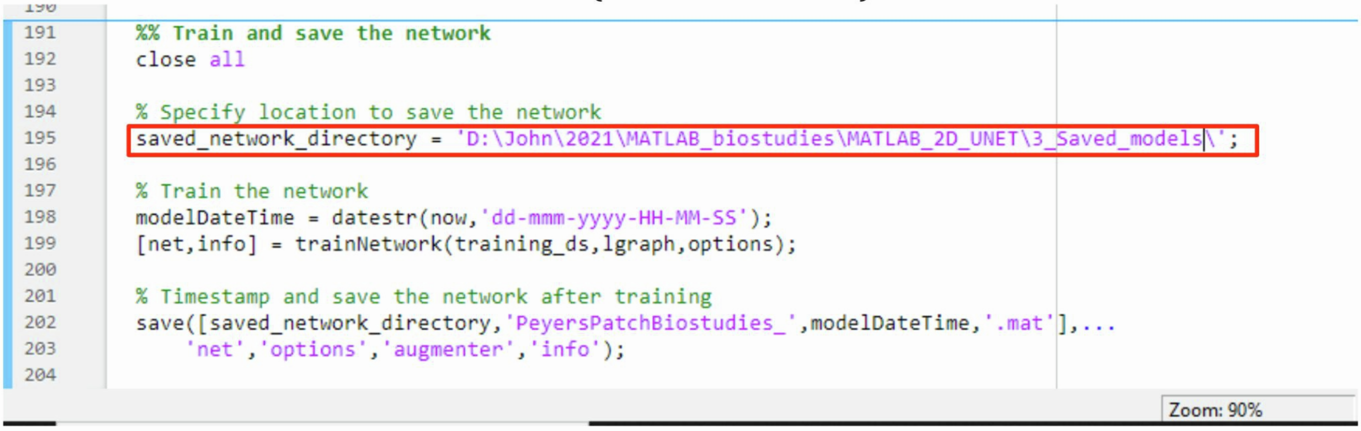

- Run the script by clicking the green arrow at the top.
- Model training takes several hours (~ 4h on a NVIDIA 1080 Ti GPU)
- The newly-trained model will be saved in the "3_Saved_models" directory with a new time/date stamp.

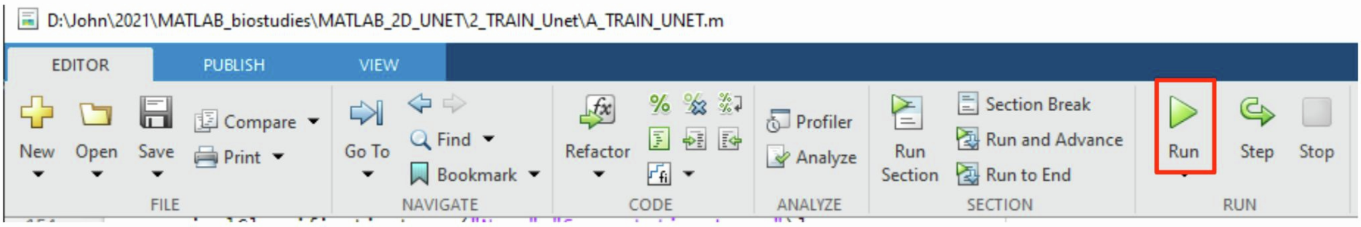

3. Testing a pretrained 2-D model using unseen data
-A screencast video is included with the BioStudies archive.
- Change the MATLAB working directory to the "4_Test_Unet" directory. Open the "A_TEST_UNET" script.

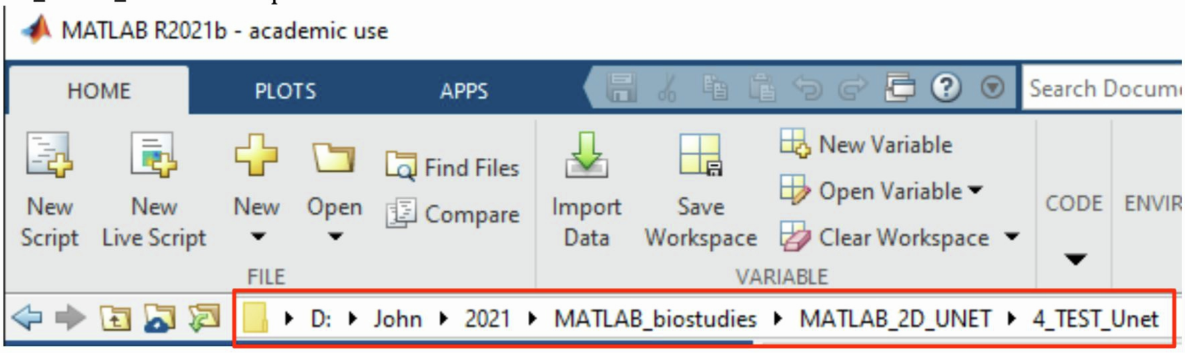

- Change the path to the unseen test image (located in the "1_ImageData" folder).
- Specify the ROI mask which identifies the lymphoid tissue (located in the "1_ImageData" folder).
- Specify which pretrained network to use (pretrained networks are located in the "3_Saved_models" folder.

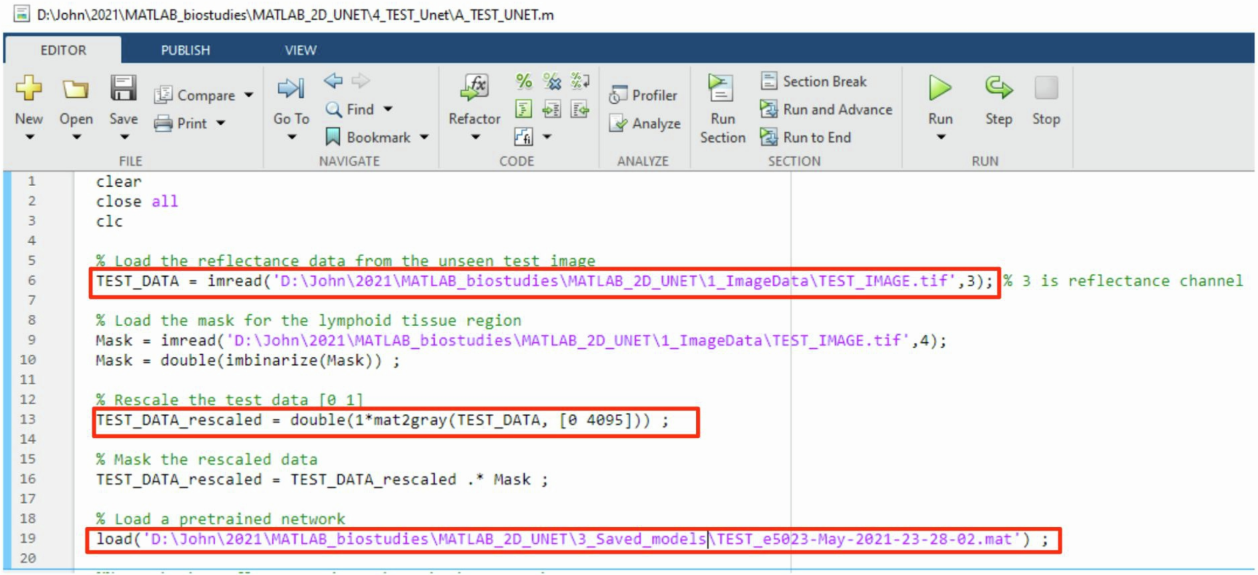

- Click the "Run" button to process the unseen reflectance data with the selected pretrained network:

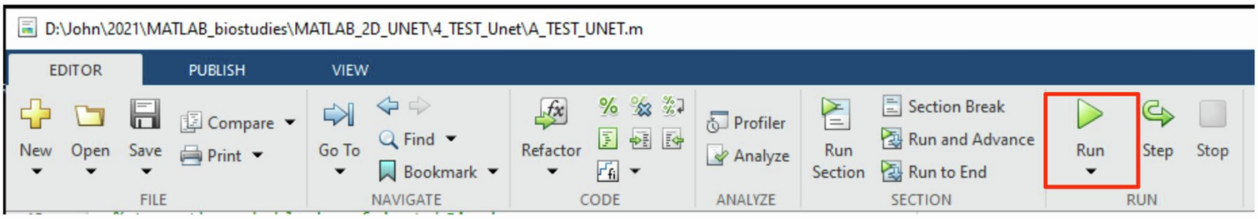

- Probability maps for the LF-nuclei, LF-actin and Background/Other classes will be saved in the working directory named ready for input into the CellProfiler pipeline (filenames Ch_5_lm_001, Ch_6_lm_001, Ch_7_lm_001, respectively).

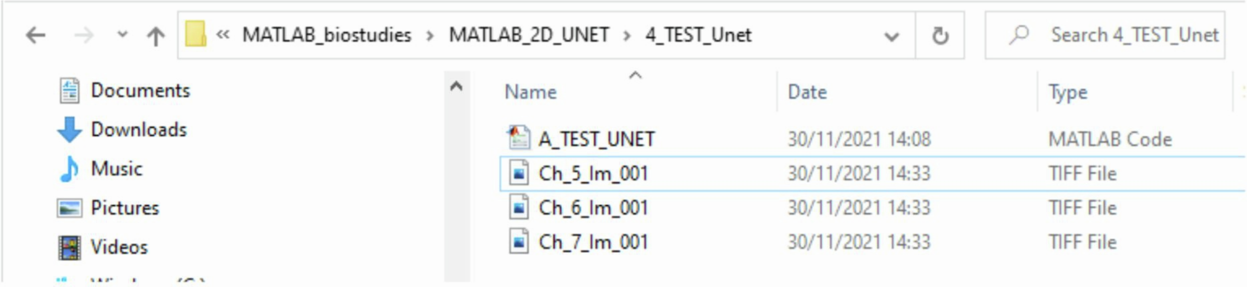

4. Training a 3-D UNET Model

-A screencast video is included with the BioStudies archive.

- Inside the MATLAB_3D_UNET folder, there is a sequentially-organised workflow starting with raw data and ending with a CellProfiler pipeline that obtains 3-D cell features from the label-free cell segmentation:

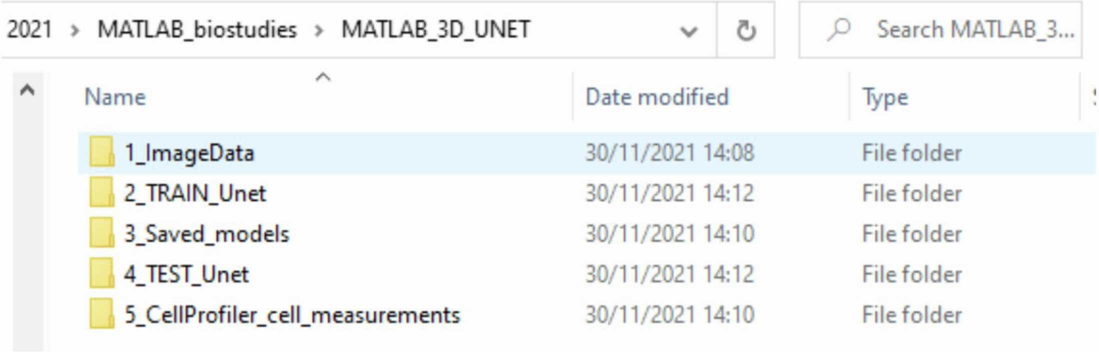

- To train a 3-D UNET model, open MATLAB and set the working directory to the "2_TRAIN_Unet" directory. Open the "A_TRAIN_3D_UNET" script.

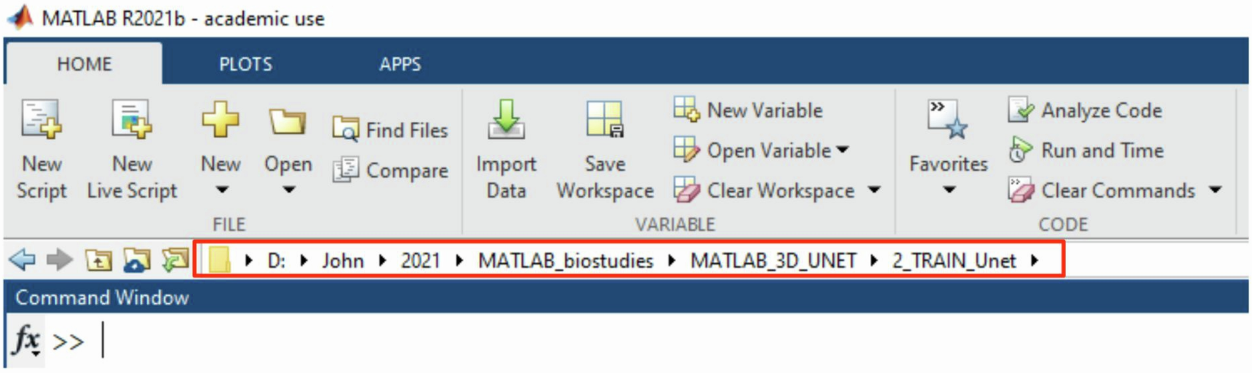

- Specify the path to the bioformats library. This is included inside the “2_TRAIN_Unet” folder (“bfmatlab”).
- Update the path to the training data directory (located in the “1_ImageData/TRAIN/DATA” folder).
- Specify the channel number in the training data that contains the reflectance information (here, ‘3’).
- Specify the location of the pixel classification training labels (located at “1_ImageData/TRAIN/LABELS” folder.

```
clear
close all
clc

% Specify path to bioformats library
addpath('D:\John\2021\MATLAB_biostudies\MATLAB_3D_UNET\2_TRAIN_Unet\bfmatlab\')

% Specify directory containing training data
Training_data_directory = 'D:\John\2021\MATLAB_biostudies\MATLAB_3D_UNET\1_ImageData\TRAIN\DATA\' ;

% Specify channel number in training data that contains reflectance information
RL_training_directory = 3;

% Specify directory containing training labels
Training_labels_path = 'D:\John\2021\MATLAB_biostudies\MATLAB_3D_UNET\1_ImageData\TRAIN\LABELS\' ;

```

- Specify the location to save the model when training completes. This should be the “3_Saved_models” directory.

```
%% Train and save the network
close all

% Specify location to save the network
saved_network_directory = 'D:\John\2021\MATLAB_biostudies\MATLAB_3D_UNET\3_Saved_models\';

% Train the network
modelDateTime = datestr(now,'dd-mmm-yyyy-HHMM');
[net,info] = trainNetwork(Training_ds,lgraph,options);

% Timestamp and save the network after training
save([saved_network_directory,'MLN_Biostudies_',modelDateTime,'.mat'],'net','options','info');

```

- Click “Run” to commence model training.
- This takes considerable time (approximately 6h on a NVIDIA 1080Ti GPU card).

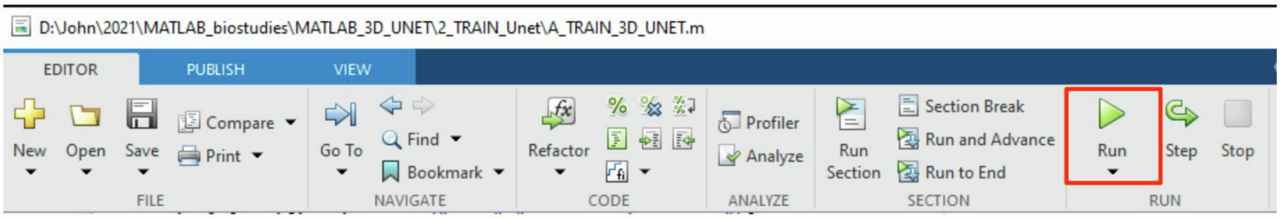

6. Testing a pretrained 3-D model using unseen data

-A screencast video is included with the BioStudies archive.

- Change the MATLAB working directory to the "4_Test_Unet" directory. Open the "A_TEST_3D_UNET" script.

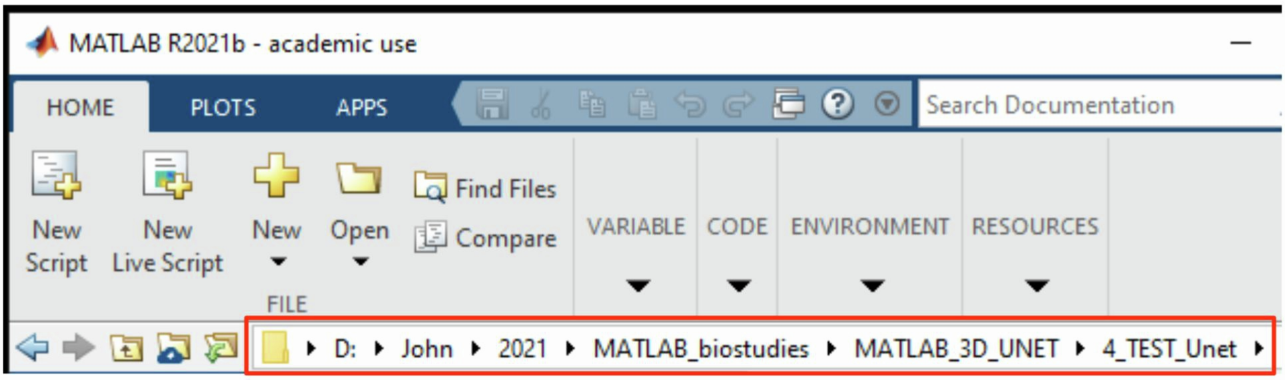

- Specify the path to the Bioformats library. (This is included inside the "4_TEST_Unet" folder ("bfmatlab").
- Specify the location of the unseen test image-data. This is located inside the "1_ImageData" folder at "1_ImageData/TEST/".

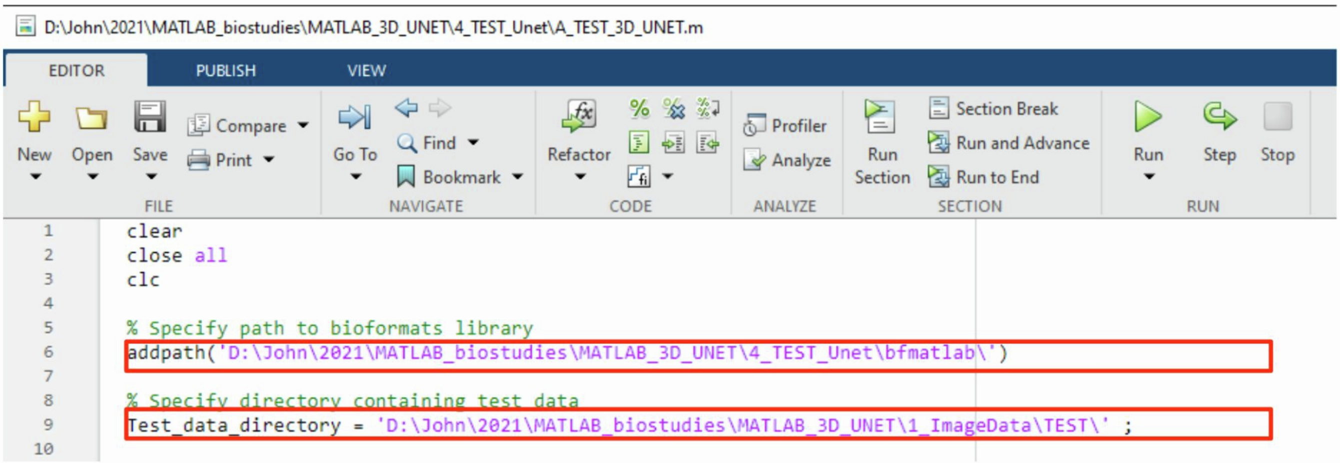

- Specify a pretrained 3-D Unet model from the "3_Saved_models" directory.

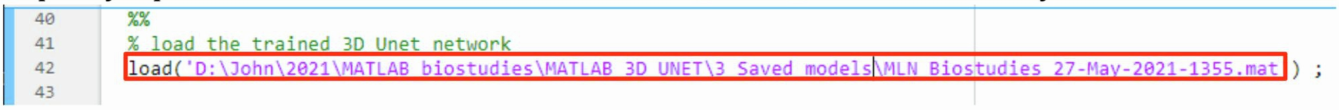

- Click "Run" to process the unseen reflectance data with the 3-D Unet model.

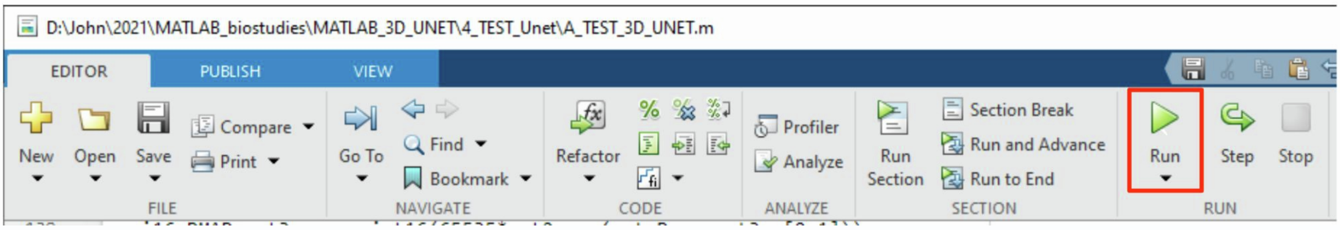

- Probability maps for the LF-nuclei, LF-actin and Background/Other classes will be saved as multipage .TIFF files in the working directory named ready for input into the CellProfiler pipeline (filenames Ch_3_lm_001, Ch_4_lm_001, Ch_5_lm_001, respectively).

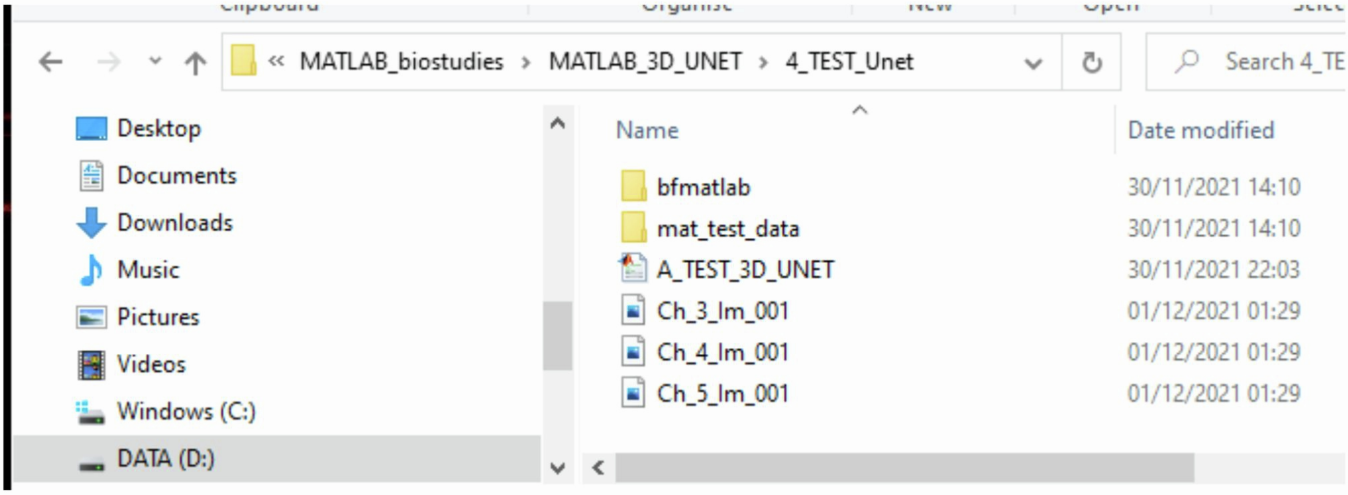

```
% MATLAB SCRIPT: TRAIN 2D UNET
% All code, image-data and screen-cast tutorial videos available for download at
% the Biostudies database under accession number S-BSST742

clear
close all
clc

% Read in the reflectance data for training
Training_data =
imread('D:\John\2021\MATLAB_biostudies\MATLAB_2D_UNET\1_ImageData\TRAIN_IMAGE.ti
f',3) ; % channel 3 is reflectance

% Read in the mask to isolated just the lymphoid tissue
mask =
imread('D:\John\2021\MATLAB_biostudies\MATLAB_2D_UNET\1_ImageData\TRAIN_IMAGE.ti
f',4) ;
mask = uint16(imbinarize(mask));

% Apply the mask to the training data
Training_data = Training_data .* mask ;

% Rescale the training data in the interval [0 1]
Training_data = double(1*mat2gray(Training_data, [0 4095])) ;

% Read in the pixel-class labels created from the nuclei and actin staining
Training_labels =
imread('D:\John\2021\MATLAB_biostudies\MATLAB_2D_UNET\1_ImageData\TRAIN_LABELS.p
ng') ;

% Visually inspect the data and labels
figure(1)
subplot(1,2,1)
imshow(Training_data, [])
title('Reflectance data')
subplot(1,2,2)
imshow(Training_labels, [])
title('Pixel classification labels')

%% Once happy with labels and data, commit the reflectance information data to
sub-directory in .mat format
if ~exist('mat_training_data', 'dir')
    mkdir('mat_training_data');
end
save([pwd,'/mat_training_data/','Training_data.mat'],'Training_data');

%% Create 'Datastores' for the reflectance information and matching pixel-class
labels
% Read saved mat file containing reflectance info into an image datastore
% Directions for processing the mat file-type are in the accompanying
'matReader' function
reflectance_ds = imageDatastore([pwd,
'/mat_training_data/'],'FileExtensions','.mat','ReadFcn',@matReader) ;

% Read pixel labels into a pixel label datastore
PixelClassNames = ["LF_actin","LF_nuclei","BackgroundOther"];
Label_Ids = 1:3; % these represent the pixel values in the labels png file
PixelLabel_ds =
pixelLabelDatastore('D:\John\2021\MATLAB_biostudies\MATLAB_2D_UNET\1_ImageData\T
RAIN_LABELS.png',...
```

```
    PixelClassNames,Label_Ids);

%% Define augmentation options
augmenter = imageDataAugmenter(...       %%% description and defaults %%%
    'FillValue',0,...                   % define out-of-bounds points when resampling
0
    'RandXReflection',true,...          % Random reflection in the left-right
direction false
    'RandYReflection',true,...          % Random reflection in the top-bottom
direction false
    'RandRotation',[0 360],...          % Range of rotation, in degrees [0 0]
    'RandScale',[1 1],...               % Range of uniform (isotropic) scaling [1 1]
    'RandXScale',[1 1],...              % Range of horizontal scaling [1 1]
    'RandYScale',[1 1],...              % Range of vertical scaling [1 1]
    'RandXShear',[0 0],...              % Range of horizontal shear [0 0]
    'RandYShear',[0 0],...              % Range of vertical shear [0 0]
    'RandXTranslation',[0 0],...        % Range of horizontal translation [0 0]
    'RandYTranslation',[0 0]...         % Range of vertical translation [0 0]
    ) ;

%% Create a training datastore comprising matching patches (256x256 pixels) of
image-data and training labels
training_ds =
randomPatchExtractionDatastore(reflectance_ds,PixelLabel_ds,[256,256],...
    'PatchesPerImage',12000,'DataAugmentation',augmenter); % 743 draws == 1
epoch

%% CREATE THE UNET
% input layer 256x256x1
% encoder depth 4
% 64 filters at the level of the first encoder

lgraph = layerGraph();
tempLayers = [
    imageInputLayer([256 256 1],"Name","ImageInputLayer")
    convolution2dLayer([3 3],64,"Name","Encoder-Stage-1-Conv-
1","Padding","same","WeightsInitializer","he")
    reluLayer("Name","Encoder-Stage-1-ReLU-1")
    convolution2dLayer([3 3],64,"Name","Encoder-Stage-1-Conv-
2","Padding","same","WeightsInitializer","he")
    reluLayer("Name","Encoder-Stage-1-ReLU-2")];
lgraph = addLayers(lgraph,tempLayers);

tempLayers = [
    maxPooling2dLayer([2 2],"Name","Encoder-Stage-1-MaxPool","Stride",[2 2])
    convolution2dLayer([3 3],128,"Name","Encoder-Stage-2-Conv-
1","Padding","same","WeightsInitializer","he")
    reluLayer("Name","Encoder-Stage-2-ReLU-1")
    convolution2dLayer([3 3],128,"Name","Encoder-Stage-2-Conv-
2","Padding","same","WeightsInitializer","he")
    reluLayer("Name","Encoder-Stage-2-ReLU-2")];
lgraph = addLayers(lgraph,tempLayers);

tempLayers = [
    maxPooling2dLayer([2 2],"Name","Encoder-Stage-2-MaxPool","Stride",[2 2])
    convolution2dLayer([3 3],256,"Name","Encoder-Stage-3-Conv-
1","Padding","same","WeightsInitializer","he")
    reluLayer("Name","Encoder-Stage-3-ReLU-1")
    convolution2dLayer([3 3],256,"Name","Encoder-Stage-3-Conv-
2","Padding","same","WeightsInitializer","he")
```

```
    reluLayer("Name","Encoder-Stage-3-ReLU-2")];
lgraph = addLayers(lgraph,tempLayers);

tempLayers = [
    maxPooling2dLayer([2 2],"Name","Encoder-Stage-3-MaxPool","Stride",[2 2])
    convolution2dLayer([3 3],512,"Name","Encoder-Stage-4-Conv-
1","Padding","same","WeightsInitializer","he")
    reluLayer("Name","Encoder-Stage-4-ReLU-1")
    convolution2dLayer([3 3],512,"Name","Encoder-Stage-4-Conv-
2","Padding","same","WeightsInitializer","he")
    reluLayer("Name","Encoder-Stage-4-ReLU-2")];
lgraph = addLayers(lgraph,tempLayers);

tempLayers = [
    dropoutLayer(0.5,"Name","Encoder-Stage-4-DropOut")
    maxPooling2dLayer([2 2],"Name","Encoder-Stage-4-MaxPool","Stride",[2 2])
    convolution2dLayer([3 3],1024,"Name","Bridge-Conv-
1","Padding","same","WeightsInitializer","he")
    reluLayer("Name","Bridge-ReLU-1")
    convolution2dLayer([3 3],1024,"Name","Bridge-Conv-
2","Padding","same","WeightsInitializer","he")
    reluLayer("Name","Bridge-ReLU-2")
    dropoutLayer(0.5,"Name","Bridge-DropOut")
    transposedConv2dLayer([2 2],512,"Name","Decoder-Stage-1-
UpConv","BiasLearnRateFactor",2,"Stride",[2 2],"WeightsInitializer","he")
    reluLayer("Name","Decoder-Stage-1-UpReLU")];
lgraph = addLayers(lgraph,tempLayers);

tempLayers = [
    depthConcatenationLayer(2,"Name","Decoder-Stage-1-DepthConcatenation")
    convolution2dLayer([3 3],512,"Name","Decoder-Stage-1-Conv-
1","Padding","same","WeightsInitializer","he")
    reluLayer("Name","Decoder-Stage-1-ReLU-1")
    convolution2dLayer([3 3],512,"Name","Decoder-Stage-1-Conv-
2","Padding","same","WeightsInitializer","he")
    reluLayer("Name","Decoder-Stage-1-ReLU-2")
    transposedConv2dLayer([2 2],256,"Name","Decoder-Stage-2-
UpConv","BiasLearnRateFactor",2,"Stride",[2 2],"WeightsInitializer","he")
    reluLayer("Name","Decoder-Stage-2-UpReLU")];
lgraph = addLayers(lgraph,tempLayers);

tempLayers = [
    depthConcatenationLayer(2,"Name","Decoder-Stage-2-DepthConcatenation")
    convolution2dLayer([3 3],256,"Name","Decoder-Stage-2-Conv-
1","Padding","same","WeightsInitializer","he")
    reluLayer("Name","Decoder-Stage-2-ReLU-1")
    convolution2dLayer([3 3],256,"Name","Decoder-Stage-2-Conv-
2","Padding","same","WeightsInitializer","he")
    reluLayer("Name","Decoder-Stage-2-ReLU-2")
    transposedConv2dLayer([2 2],128,"Name","Decoder-Stage-3-
UpConv","BiasLearnRateFactor",2,"Stride",[2 2],"WeightsInitializer","he")
    reluLayer("Name","Decoder-Stage-3-UpReLU")];
lgraph = addLayers(lgraph,tempLayers);

tempLayers = [
    depthConcatenationLayer(2,"Name","Decoder-Stage-3-DepthConcatenation")
    convolution2dLayer([3 3],128,"Name","Decoder-Stage-3-Conv-
1","Padding","same","WeightsInitializer","he")
    reluLayer("Name","Decoder-Stage-3-ReLU-1")
```

```
    convolution2dLayer([3 3],128,"Name","Decoder-Stage-3-Conv-
2","Padding","same","WeightsInitializer","he")
    reluLayer("Name","Decoder-Stage-3-ReLU-2")
    transposedConv2dLayer([2 2],64,"Name","Decoder-Stage-4-
UpConv","BiasLearnRateFactor",2,"Stride",[2 2],"WeightsInitializer","he")
    reluLayer("Name","Decoder-Stage-4-UpReLU")];
lgraph = addLayers(lgraph,tempLayers);

tempLayers = [
    depthConcatenationLayer(2,"Name","Decoder-Stage-4-DepthConcatenation")
    convolution2dLayer([3 3],64,"Name","Decoder-Stage-4-Conv-
1","Padding","same","WeightsInitializer","he")
    reluLayer("Name","Decoder-Stage-4-ReLU-1")
    convolution2dLayer([3 3],64,"Name","Decoder-Stage-4-Conv-
2","Padding","same","WeightsInitializer","he")
    reluLayer("Name","Decoder-Stage-4-ReLU-2")
    convolution2dLayer([1 1],3,"Name","Final-
ConvolutionLayer","Padding","same","WeightsInitializer","he")
    softmaxLayer("Name","Softmax-Layer")
    pixelClassificationLayer("Name","Segmentation-Layer")];
lgraph = addLayers(lgraph,tempLayers);

clear tempLayers;

% encoder / decoder connections
lgraph = connectLayers(lgraph,"Encoder-Stage-1-ReLU-2","Encoder-Stage-1-
MaxPool");
lgraph = connectLayers(lgraph,"Encoder-Stage-1-ReLU-2","Decoder-Stage-4-
DepthConcatenation/in2");
lgraph = connectLayers(lgraph,"Encoder-Stage-2-ReLU-2","Encoder-Stage-2-
MaxPool");
lgraph = connectLayers(lgraph,"Encoder-Stage-2-ReLU-2","Decoder-Stage-3-
DepthConcatenation/in2");
lgraph = connectLayers(lgraph,"Encoder-Stage-3-ReLU-2","Encoder-Stage-3-
MaxPool");
lgraph = connectLayers(lgraph,"Encoder-Stage-3-ReLU-2","Decoder-Stage-2-
DepthConcatenation/in2");
lgraph = connectLayers(lgraph,"Encoder-Stage-4-ReLU-2","Encoder-Stage-4-
DropOut");
lgraph = connectLayers(lgraph,"Encoder-Stage-4-ReLU-2","Decoder-Stage-1-
DepthConcatenation/in2");
lgraph = connectLayers(lgraph,"Decoder-Stage-1-UpReLU","Decoder-Stage-1-
DepthConcatenation/in1");
lgraph = connectLayers(lgraph,"Decoder-Stage-2-UpReLU","Decoder-Stage-2-
DepthConcatenation/in1");
lgraph = connectLayers(lgraph,"Decoder-Stage-3-UpReLU","Decoder-Stage-3-
DepthConcatenation/in1");
lgraph = connectLayers(lgraph,"Decoder-Stage-4-UpReLU","Decoder-Stage-4-
DepthConcatenation/in1");

% analyzeNetwork(lgraph) % comment in to check structure and errors
plot(lgraph) % comment in to show network structure

%% Specify training options
options = trainingOptions('sgdm',...
    'InitialLearnRate',0.05, ...
    'Momentum',0.9,...
    'L2Regularization',0.0001,...
    'MaxEpochs',50,...
    'MiniBatchSize',12,...
```

```
    'LearnRateSchedule','piecewise',...
    'Shuffle','every-epoch',...
    'GradientThresholdMethod','l2norm',...
    'GradientThreshold',0.05, ...
    'Plots','training-progress', ...
    'VerboseFrequency',20,...
    'ExecutionEnvironment','auto');

%% Train and save the network
close all

% Specify location to save the network
saved_network_directory =
'D:\John\2021\MATLAB_biostudies\MATLAB_2D_UNET\3_Saved_models\';

% Train the network
modelDateTime = datestr(now,'dd-mmm-yyyy-HH-MM-SS');
[net,info] = trainNetwork(training_ds,lgraph,options);

% Timestamp and save the network after training
save([saved_network_directory,'PeyersPatchBiostudies_',modelDateTime,'.mat'],...
    'net','options','augmenter','info');
```

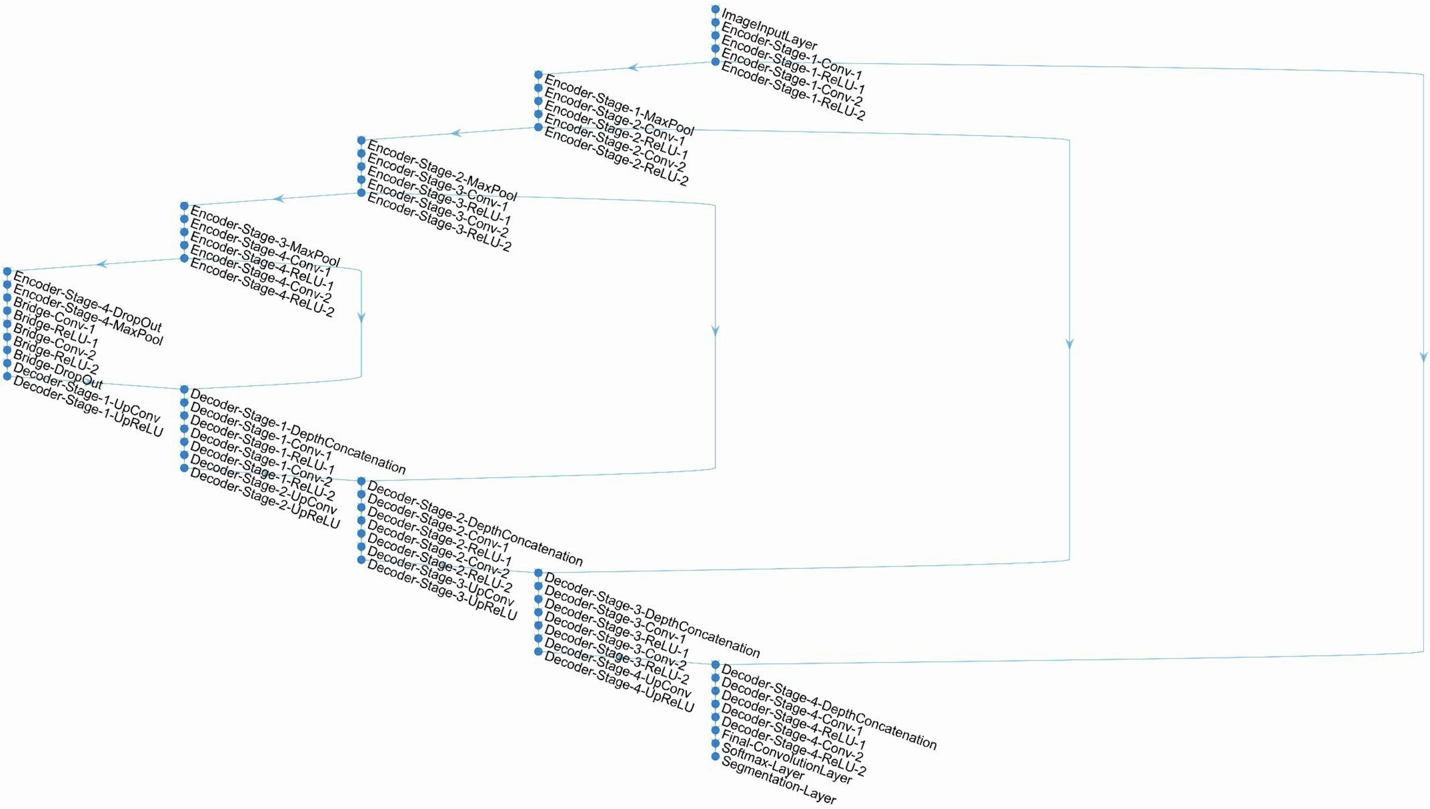

2-D Unet architecture schematic. The network uses an input layer for the reflectance data of 256x256x1 (x, y, channels). The best performing three-class Unet architecture uses an encoder depth of 4 with 64 filters at the level of the first encoder (shown, **Figure S2**). The network uses complete up-convolutional expansion to yield outputted probability maps that are identically sized to the input layer.

```
% MATLAB SCRIPT: TEST 2D UNET
% All code, image-data and screen-cast tutorial videos available for download at
% the Biostudies database under accession number S-BSST742

clear
close all
clc

% Load the reflectance data from the unseen test image
TEST_DATA =
imread('D:\John\2021\MATLAB_biostudies\MATLAB_2D_UNET\1_ImageData\TEST_IMAGE.tif
',3); % 3 is reflectance channel

% Load the mask for the lymphoid tissue region
Mask =
imread('D:\John\2021\MATLAB_biostudies\MATLAB_2D_UNET\1_ImageData\TEST_IMAGE.tif
',4);
Mask = double(imbinarize(Mask)) ;

% Rescale the test data [0 1]
TEST_DATA_rescaled = double(1*mat2gray(TEST_DATA, [0 4095])) ;

% Mask the rescaled data
TEST_DATA_rescaled = TEST_DATA_rescaled .* Mask ;

% Load a pretrained network
load('D:\John\2021\MATLAB_biostudies\MATLAB_2D_UNET\3_Saved_models\Rescaled0-
1_2DUNET_S7-RL-SA_enc-4_filt-64_e50.mat') ;

%% Patch the reflectance data through the network
%%%%%%%%%%%%%%%%%%%%%%%%%%%%%%%%%%%%%%%%%%%%%%%%%
% Set the patch sizes to be passed to the net
patchSize = [2048 2048]; % decrease if insufficient GPU memory eg., [1024 1024]

% Segment blockwise then reassemble in full
% Define image dimensions
[height, width, nChannel] = size(TEST_DATA_rescaled);
patch = zeros([patchSize, nChannel], 'like', TEST_DATA_rescaled);

% Pad image to have dimensions as multiples of patchSize
padSize(1) = patchSize(1) - mod(height, patchSize(1));
padSize(2) = patchSize(2) - mod(width, patchSize(2));
im_pad = padarray (TEST_DATA_rescaled, padSize, 0, 'post');
[height_pad, width_pad, nChannel_pad] = size(im_pad);

% Preallocate some matrices to receive the network outputs
out_Uncertainty_Scores = zeros([size(im_pad,1), size(im_pad,2)], 'double');
out_Pmap_cat1 = out_Uncertainty_Scores;
out_Pmap_cat2 = out_Uncertainty_Scores;
out_Pmap_cat3 = out_Uncertainty_Scores;

% Loop through blocks of 'patchSize'
    for loop = 1:patchSize(1):height_pad
        for j =1:patchSize(2):width_pad
            for p = 1:nChannel
                patch(:,:,p) = squeeze( im_pad( loop:loop+patchSize(1)-1,...
                                                j:j+patchSize(2)-1,p));
            end
            % deploy net
```

```
            [patch_seg, Scores, allScores] = semanticseg(patch, net,
'OutputType', 'double',...
                'ExecutionEnvironment','auto');
            % catch what comes out
            out_Uncertainty_Scores(loop:loop+patchSize(1)-1, j:j+patchSize(2)-1)
= Scores;
            out_Pmap_cat1(loop:loop+patchSize(1)-1, j:j+patchSize(2)-1) =
allScores(:,:,1);
            out_Pmap_cat2(loop:loop+patchSize(1)-1, j:j+patchSize(2)-1) =
allScores(:,:,2);
            out_Pmap_cat3(loop:loop+patchSize(1)-1, j:j+patchSize(2)-1) =
allScores(:,:,3);
        end
    end

% Remove padding from the network outputs
out_Uncertainty_Scores = out_Uncertainty_Scores(1:height, 1:width);
out_Pmap_cat1 = out_Pmap_cat1(1:height, 1:width);
out_Pmap_cat2 = out_Pmap_cat2(1:height, 1:width);
out_Pmap_cat3 = out_Pmap_cat3(1:height, 1:width);

%% visualise network outputs
figure(2)
clf(figure(2))
ax1 = subplot(2,3,1);
imshow(out_Pmap_cat1,[])
title('LF-actin')
ax2 = subplot(2,3,2) ;
imshow(out_Pmap_cat2,[])
title('LF-nuclei')
ax3 = subplot(2,3,3);
imshow(out_Pmap_cat3,[])
title('Background/other')
ax4 = subplot(2,3,5);
imshow(out_Uncertainty_Scores,[])
title('Uncertainty')
linkaxes([ax1 ax2 ax3 ax4], 'xy')

%% Map to 16-bit and save for loading into CellProfiler
ui16_PMAP_cat1_xy = uint16(65535*mat2gray(out_Pmap_cat1, [0 1])) ;
imwrite(ui16_PMAP_cat1_xy, ['Ch_6_Im_001','.tiff']) ;

ui16_PMAP_cat2_xy = uint16(65535*mat2gray(out_Pmap_cat2, [0 1])) ;
imwrite(ui16_PMAP_cat2_xy, ['Ch_5_Im_001','.tiff']) ;

ui16_PMAP_cat3_xy = uint16(65535*mat2gray(out_Pmap_cat3, [0 1])) ;
imwrite(ui16_PMAP_cat3_xy, ['Ch_7_Im_001','.tiff']) ;
```

```
% MATLAB SCRIPT: Train 3D UNET
% All code, image-data and screen-cast tutorial videos available for download at
% the Biostudies database under accession number S-BSST742

clear
close all
clc

% Specify path to bioformats library
addpath('D:\John\2021\MATLAB_biostudies\MATLAB_3D_UNET\2_TRAIN_Unet\bfmatlab\')

% Specify directory containing training data
Training_data_directory =
'D:\John\2021\MATLAB_biostudies\MATLAB_3D_UNET\1_ImageData\TRAIN\DATA\' ;

% Specify channel number in training data that contains reflectance information
RL_training_directory = 3;

% Specify directory containing training labels
Training_labels_path =
'D:\John\2021\MATLAB_biostudies\MATLAB_3D_UNET\1_ImageData\TRAIN\LABELS\' ;

%% COMMIT TRAINING DATA TO DIRECTORY rescaled [0 1] IN MAT FORMAT
if ~exist('mat_training_data', 'dir')
    mkdir('mat_training_data');
end

for loop = 1:2
    counter = sprintf('%03d',loop) ;

    % find metadata describing file
    reader = bfGetReader([Training_data_directory,'TRAIN_',counter,'.tif']);
    omeMeta = reader.getMetadataStore();
    number_of_channels = omeMeta.getChannelCount(0);
    stackSizeZ = omeMeta.getPixelsSizeZ(0).getValue(); % number of Z slices

    % Load reflectance information
    for zplane = 1:stackSizeZ
        iPlane = reader.getIndex(zplane -1, RL_training_directory -1, 0) + 1; %
because zplanes and channels are numbered from zero
        channel_zimage{zplane} = bfGetPlane(reader, iPlane);
    end

    IM_DATA = cat(3,channel_zimage{:}) ;
    IM_DATA = double(1*mat2gray(IM_DATA, [0 65535])) ;
    save([pwd,'/mat_training_data/','TRAIN_DATA_',counter,'.mat'], 'IM_DATA') ;
end

%% COMMIT TRAINING LABELS TO DIRECTORY IN MAT FORMAT
if ~exist('mat_training_labels', 'dir')
    mkdir('mat_training_labels');
end

for loop = 1:2
    counter = sprintf('%03d',loop) ;

        % load label information
        for zplane = 1:stackSizeZ
            iplane =
imread([Training_labels_path,'LABELS_',counter,'.tif'],zplane);
```

```
            label_zimage{zplane} = iplane ;
        end

        IM_LABELS = cat(3,label_zimage{:});
        IM_LABELS = double(IM_LABELS) ;
        save([pwd,'/mat_training_labels/','TRAIN_LABELS_',counter,'.mat'],
'IM_LABELS') ;
end

% Once data is prepared for training, clear workspace
clear ;

%% Create 'Datastores' for the reflectance information and matching pixel-class
labels
% Read saved mat file containing reflectance info into an image datastore
% Directions for processing the mat file-type are in the accompanying 'matRead'
function
data_location = [pwd, '\mat_training_data\'] ;
volReader = @(x) JWmatRead(x) ;
reflectance_ds =
imageDatastore(data_location,'FileExtensions','.mat','ReadFcn',volReader);

% Read pixel labels into a pixel label datastore
label_location = [pwd, '\mat_training_labels\'] ;
labelReader = @(x) JWLabelRead(x);
classNames = ["LFNuclei","LFActin","BackgroundOther"];
pixelLabelID = 1:3; % these represent the pixel values in the labels file
PixelLabel_ds = pixelLabelDatastore(label_location,classNames,pixelLabelID,...
    'FileExtensions','.mat','ReadFcn',labelReader);

%% Set up patch extraction from reflectance datastore
patchSize = [64 64 32];
patchPerImage = 375;
MiniBatchSize = 8; % set the batch size
reflectance_patches_ds =
randomPatchExtractionDatastore(reflectance_ds,PixelLabel_ds,patchSize,'PatchesPe
rImage',patchPerImage);
reflectance_patches_ds.MiniBatchSize = MiniBatchSize;

% Augment the patches using 'augment3dPatch' function
Training_ds = transform(reflectance_patches_ds,@JWaugment3dPatch);

%% CREATE THE 3D UNET
% input layer 64x64x32x1
% encoder depth 4
% 32 filters at the level of the first encoder

lgraph = layerGraph();

tempLayers = [
    image3dInputLayer([64 64 32 1],"Name","ImageInputLayer")
    convolution3dLayer([3 3 3],32,"Name","Encoder-Stage-1-Conv-
1","Padding","same","WeightsInitializer","he")
    batchNormalizationLayer("Name","Encoder-Stage-1-BN-1")
    reluLayer("Name","Encoder-Stage-1-ReLU-1")
    convolution3dLayer([3 3 3],64,"Name","Encoder-Stage-1-Conv-
2","Padding","same","WeightsInitializer","he")
    batchNormalizationLayer("Name","Encoder-Stage-1-BN-2")
    reluLayer("Name","Encoder-Stage-1-ReLU-2")];
lgraph = addLayers(lgraph,tempLayers);
```

```
tempLayers = [
    maxPooling3dLayer([2 2 2],"Name","Encoder-Stage-1-MaxPool","Stride",[2 2 2])
    convolution3dLayer([3 3 3],64,"Name","Encoder-Stage-2-Conv-
1","Padding","same","WeightsInitializer","he")
    batchNormalizationLayer("Name","Encoder-Stage-2-BN-1")
    reluLayer("Name","Encoder-Stage-2-ReLU-1")
    convolution3dLayer([3 3 3],128,"Name","Encoder-Stage-2-Conv-
2","Padding","same","WeightsInitializer","he")
    batchNormalizationLayer("Name","Encoder-Stage-2-BN-2")
    reluLayer("Name","Encoder-Stage-2-ReLU-2")];
lgraph = addLayers(lgraph,tempLayers);

tempLayers = [
    maxPooling3dLayer([2 2 2],"Name","Encoder-Stage-2-MaxPool","Stride",[2 2 2])
    convolution3dLayer([3 3 3],128,"Name","Encoder-Stage-3-Conv-
1","Padding","same","WeightsInitializer","he")
    batchNormalizationLayer("Name","Encoder-Stage-3-BN-1")
    reluLayer("Name","Encoder-Stage-3-ReLU-1")
    convolution3dLayer([3 3 3],256,"Name","Encoder-Stage-3-Conv-
2","Padding","same","WeightsInitializer","he")
    batchNormalizationLayer("Name","Encoder-Stage-3-BN-2")
    reluLayer("Name","Encoder-Stage-3-ReLU-2")];
lgraph = addLayers(lgraph,tempLayers);

tempLayers = [
    maxPooling3dLayer([2 2 2],"Name","Encoder-Stage-3-MaxPool","Stride",[2 2 2])
    convolution3dLayer([3 3 3],256,"Name","Encoder-Stage-4-Conv-
1","Padding","same","WeightsInitializer","he")
    batchNormalizationLayer("Name","Encoder-Stage-4-BN-1")
    reluLayer("Name","Encoder-Stage-4-ReLU-1")
    convolution3dLayer([3 3 3],512,"Name","Encoder-Stage-4-Conv-
2","Padding","same","WeightsInitializer","he")
    batchNormalizationLayer("Name","Encoder-Stage-4-BN-2")
    reluLayer("Name","Encoder-Stage-4-ReLU-2")];
lgraph = addLayers(lgraph,tempLayers);

tempLayers = [
    maxPooling3dLayer([2 2 2],"Name","Encoder-Stage-4-MaxPool","Stride",[2 2 2])
    convolution3dLayer([3 3 3],512,"Name","Bridge-Conv-
1","Padding","same","WeightsInitializer","he")
    batchNormalizationLayer("Name","Bridge-BN-1")
    reluLayer("Name","Bridge-ReLU-1")
    convolution3dLayer([3 3 3],1024,"Name","Bridge-Conv-
2","Padding","same","WeightsInitializer","he")
    batchNormalizationLayer("Name","Bridge-BN-2")
    reluLayer("Name","Bridge-ReLU-2")
    transposedConv3dLayer([2 2 2],1024,"Name","Decoder-Stage-1-
UpConv","BiasLearnRateFactor",2,"Stride",[2 2 2],"WeightsInitializer","he")];
lgraph = addLayers(lgraph,tempLayers);

tempLayers = [
    concatenationLayer(4,2,"Name","Decoder-Stage-1-Concatenation")
    convolution3dLayer([3 3 3],512,"Name","Decoder-Stage-1-Conv-
1","Padding","same","WeightsInitializer","he")
    batchNormalizationLayer("Name","Decoder-Stage-1-BN-1")
    reluLayer("Name","Decoder-Stage-1-ReLU-1")
    convolution3dLayer([3 3 3],512,"Name","Decoder-Stage-1-Conv-
2","Padding","same","WeightsInitializer","he")
    batchNormalizationLayer("Name","Decoder-Stage-1-BN-2")
```

```
    reluLayer("Name","Decoder-Stage-1-ReLU-2")
    transposedConv3dLayer([2 2 2],512,"Name","Decoder-Stage-2-
UpConv","BiasLearnRateFactor",2,"Stride",[2 2 2],"WeightsInitializer","he")];
lgraph = addLayers(lgraph,tempLayers);

tempLayers = [
    concatenationLayer(4,2,"Name","Decoder-Stage-2-Concatenation")
    convolution3dLayer([3 3 3],256,"Name","Decoder-Stage-2-Conv-
1","Padding","same","WeightsInitializer","he")
    batchNormalizationLayer("Name","Decoder-Stage-2-BN-1")
    reluLayer("Name","Decoder-Stage-2-ReLU-1")
    convolution3dLayer([3 3 3],256,"Name","Decoder-Stage-2-Conv-
2","Padding","same","WeightsInitializer","he")
    batchNormalizationLayer("Name","Decoder-Stage-2-BN-2")
    reluLayer("Name","Decoder-Stage-2-ReLU-2")
    transposedConv3dLayer([2 2 2],256,"Name","Decoder-Stage-3-
UpConv","BiasLearnRateFactor",2,"Stride",[2 2 2],"WeightsInitializer","he")];
lgraph = addLayers(lgraph,tempLayers);

tempLayers = [
    concatenationLayer(4,2,"Name","Decoder-Stage-3-Concatenation")
    convolution3dLayer([3 3 3],128,"Name","Decoder-Stage-3-Conv-
1","Padding","same","WeightsInitializer","he")
    batchNormalizationLayer("Name","Decoder-Stage-3-BN-1")
    reluLayer("Name","Decoder-Stage-3-ReLU-1")
    convolution3dLayer([3 3 3],128,"Name","Decoder-Stage-3-Conv-
2","Padding","same","WeightsInitializer","he")
    batchNormalizationLayer("Name","Decoder-Stage-3-BN-2")
    reluLayer("Name","Decoder-Stage-3-ReLU-2")
    transposedConv3dLayer([2 2 2],128,"Name","Decoder-Stage-4-
UpConv","BiasLearnRateFactor",2,"Stride",[2 2 2],"WeightsInitializer","he")];
lgraph = addLayers(lgraph,tempLayers);

tempLayers = [
    concatenationLayer(4,2,"Name","Decoder-Stage-4-Concatenation")
    convolution3dLayer([3 3 3],64,"Name","Decoder-Stage-4-Conv-
1","Padding","same","WeightsInitializer","he")
    batchNormalizationLayer("Name","Decoder-Stage-4-BN-1")
    reluLayer("Name","Decoder-Stage-4-ReLU-1")
    convolution3dLayer([3 3 3],64,"Name","Decoder-Stage-4-Conv-
2","Padding","same","WeightsInitializer","he")
    batchNormalizationLayer("Name","Decoder-Stage-4-BN-2")
    reluLayer("Name","Decoder-Stage-4-ReLU-2")
    convolution3dLayer([1 1 1],3,"Name","Final-
ConvolutionLayer","Padding","same","WeightsInitializer","he")
    softmaxLayer("Name","Softmax-Layer")
    pixelClassificationLayer("Name","Segmentation-Layer")];
lgraph = addLayers(lgraph,tempLayers);

clear tempLayers;

% encoder / decoder connections
lgraph = connectLayers(lgraph,"Encoder-Stage-1-ReLU-2","Encoder-Stage-1-
MaxPool");
lgraph = connectLayers(lgraph,"Encoder-Stage-1-ReLU-2","Decoder-Stage-4-
Concatenation/in2");
lgraph = connectLayers(lgraph,"Encoder-Stage-2-ReLU-2","Encoder-Stage-2-
MaxPool");
lgraph = connectLayers(lgraph,"Encoder-Stage-2-ReLU-2","Decoder-Stage-3-
Concatenation/in2");
```

```
lgraph = connectLayers(lgraph,"Encoder-Stage-3-ReLU-2","Encoder-Stage-3-
MaxPool");
lgraph = connectLayers(lgraph,"Encoder-Stage-3-ReLU-2","Decoder-Stage-2-
Concatenation/in2");
lgraph = connectLayers(lgraph,"Encoder-Stage-4-ReLU-2","Encoder-Stage-4-
MaxPool");
lgraph = connectLayers(lgraph,"Encoder-Stage-4-ReLU-2","Decoder-Stage-1-
Concatenation/in2");
lgraph = connectLayers(lgraph,"Decoder-Stage-1-UpConv","Decoder-Stage-1-
Concatenation/in1");
lgraph = connectLayers(lgraph,"Decoder-Stage-2-UpConv","Decoder-Stage-2-
Concatenation/in1");
lgraph = connectLayers(lgraph,"Decoder-Stage-3-UpConv","Decoder-Stage-3-
Concatenation/in1");
lgraph = connectLayers(lgraph,"Decoder-Stage-4-UpConv","Decoder-Stage-4-
Concatenation/in1");

% analyzeNetwork(lgraph) % comment in to check structure and errors
plot(lgraph) % comment in to show network structure

%% Specify training options
options = trainingOptions('adam', ...
    'MaxEpochs',150, ...
    'InitialLearnRate',5e-4, ...
    'L2Regularization',1e-4,...
    'LearnRateSchedule','piecewise', ...
    'LearnRateDropPeriod',5,...
    'LearnRateDropFactor',0.95, ...
    'Plots','training-progress', ...
    'Verbose',true, ...
    'VerboseFrequency',20,...
    'Shuffle','every-epoch',...%
    'ExecutionEnvironment','auto',...
    'MiniBatchSize',MiniBatchSize);

%% Train and save the network
close all

% Specify location to save the network
saved_network_directory =
'D:\John\2021\MATLAB_biostudies\MATLAB_3D_UNET\3_Saved_models\';

% Train the network
modelDateTime = datestr(now,'dd-mmm-yyyy-HHMM');
[net,info] = trainNetwork(Training_ds,lgraph,options);

% Timestamp and save the network after training
save([saved_network_directory,'MLN_Biostudies_',modelDateTime,'.mat'],'net','opt
ions','info');
```

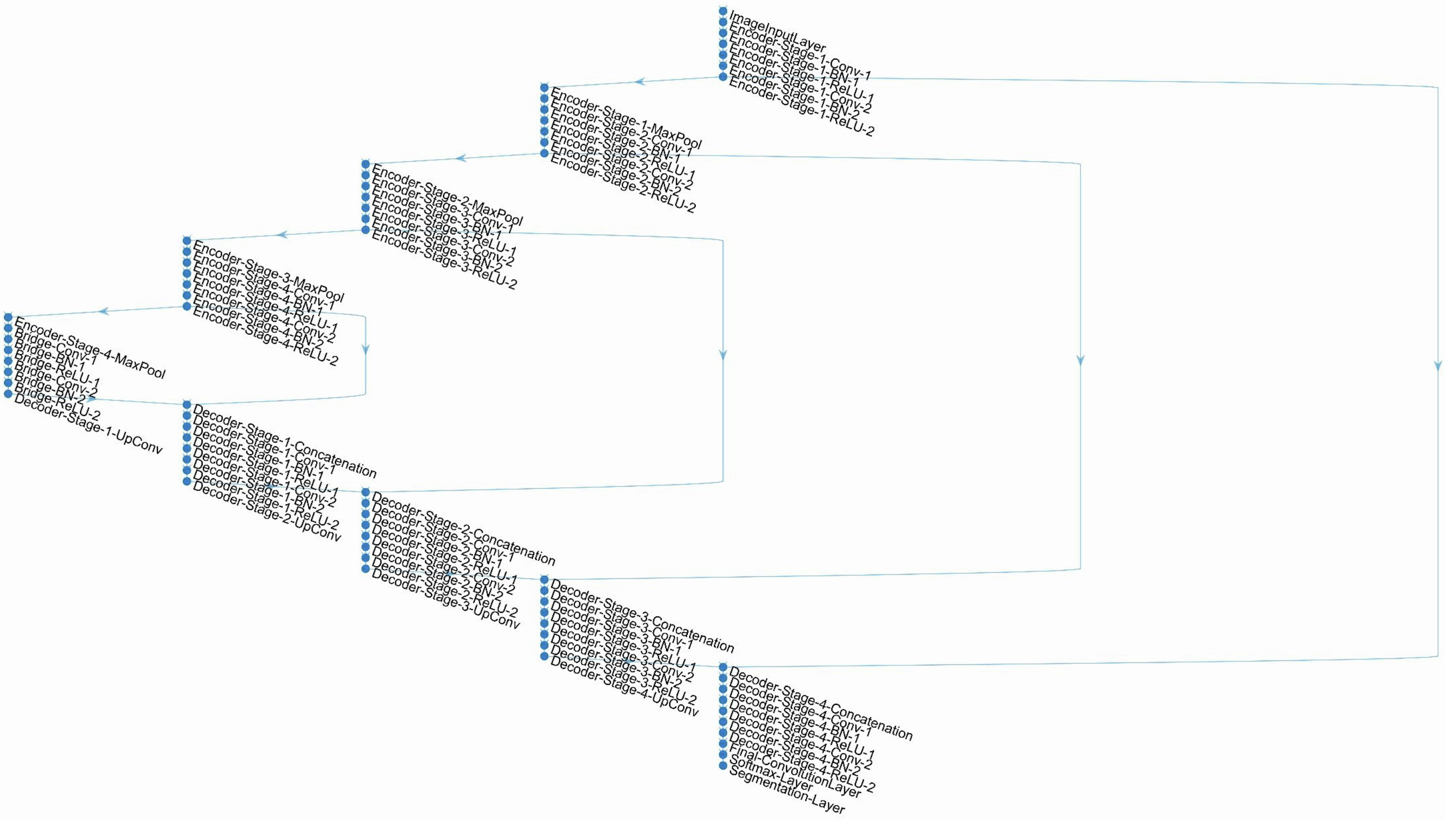

3-D Unet architecture schematic. The network uses an input layer for the reflectance data of 64x64x64x1 (x, y, z, channels). The three-class Unet architecture uses an encoder depth of 4 with 64 filters at the level of the first encoder. The network uses complete up-convolutional expansion to yield outputted probability maps that are identically sized to the input layer.

```
% MATLAB SCRIPT: TEST 3D UNET
% All code, image-data and screen-cast tutorial videos available for download at
% the Biostudies database under accession number S-BSST742

clear
close all
clc

% Specify path to bioformats library
addpath('D:\John\2021\MATLAB_biostudies\MATLAB_3D_UNET\4_TEST_Unet\bfmatlab\')

% Specify directory containing test data
Test_data_directory =
'D:\John\2021\MATLAB_biostudies\MATLAB_3D_UNET\1_ImageData\TEST\' ;

% Specify channel number in test data that contains reflectance information
RL_channel_number = 2;

% COMMIT REFLECTANCE TEST DATA TO DIRECTORY RESCALED [0 1] IN MAT FORMAT
if ~exist('mat_test_data', 'dir')
    mkdir('mat_test_data');
end

for loop = 1:1
    counter = sprintf('%03d',loop) ;

    % find metadata describing file
    reader = bfGetReader([Test_data_directory,'TEST_',counter,'.tif']);
    omeMeta = reader.getMetadataStore();
    number_of_channels = omeMeta.getChannelCount(0);
    stackSizeZ = omeMeta.getPixelsSizeZ(0).getValue(); % number of Z slices

    % Load reflectance information
    for zplane = 1:stackSizeZ
        iPlane = reader.getIndex(zplane -1, RL_channel_number -1, 0) + 1; %
because zplanes and channels are numbered from zero
        channel_zimage{zplane} = bfGetPlane(reader, iPlane);
    end

    IM_DATA = cat(3,channel_zimage{:}) ;
    IM_DATA = double(1*mat2gray(IM_DATA, [0 65535])) ; % rescale zero-one
    save([pwd,'/mat_test_data/','TEST_DATA_',counter,'.mat'], 'IM_DATA') ;
end
clear

%%
% load the trained 3D Unet network
load('D:\John\2021\MATLAB_biostudies\MATLAB_3D_UNET\3_Saved_models\MLN_Biostudie
s_27-May-2021-1355.mat') ;

% load the rescaled reflectance test data
load([pwd,'/mat_test_data/','TEST_DATA_001.mat']);

%% Patch the reflectance data through the network
%%%%%%%%%%%%%%%%%%%%%%%%%%%%%%%%%%%%%%%%%%%%%%%%%
% Set the patch sizes to be passed to the net
patchSize = [256 256 64];

% Segment blockwise then reassemble in full
% Define image dimensions
```

```
[height, width, depth, nChannel] = size(IM_DATA);
patch = zeros([patchSize, nChannel], 'like', IM_DATA);
number_of_height_patches =ceil(height/patchSize(1));
number_of_width_patches  =ceil(width/patchSize(2));
number_of_depth_patches  =ceil(depth/patchSize(3));

% Pad image to have dimensions as multiples of patchSize
height_pad = number_of_height_patches*patchSize(1);
width_pad  = number_of_width_patches*patchSize(2);
depth_pad  = number_of_depth_patches*patchSize(3);

% Amount to pad different dimensions of image
padSize(1) = height_pad - height;
padSize(2) = width_pad - width;
padSize(3) = depth_pad - depth;

% Pad the image by correct amounts
im_pad = padarray (IM_DATA, padSize, 0, 'post');

% Preallocate some matrices to catch the probability maps
out_Uncertainty_Scores = zeros([size(im_pad,1), size(im_pad,2), size(im_pad,3)],
'double'); % needs to match OutputType in semanticseg below
out_scores_from_network_all_classes=zeros([size(im_pad,1), size(im_pad,2),
size(im_pad,3), 3], 'double');

% Loop through blocks of 'patchSize'
for loop_height = 1:number_of_height_patches
    for loop_width = 1:number_of_width_patches
        for loop_depth = 1:number_of_depth_patches

            start_height_position=(loop_height-1)*patchSize(1)+1;
            end_height_position=loop_height*patchSize(1);

            start_width_position=(loop_width-1)*patchSize(2)+1;
            end_width_position=loop_width*patchSize(2);

            start_depth_position=(loop_depth-1)*patchSize(3)+1;
            end_depth_position=loop_depth*patchSize(3);

            patch_to_deploy=...

im_pad(start_height_position:end_height_position,start_width_position:end_width_
position,start_depth_position:end_depth_position,:);

            % deploy net
            [patch_seg, Scores, allScores] = semanticseg(patch_to_deploy, net,
'OutputType', 'double',...
             'ExecutionEnvironment','auto');


out_Uncertainty_Scores(start_height_position:end_height_position,start_width_pos
ition:end_width_position,start_depth_position:end_depth_position)...
            =Scores;

out_scores_from_network_all_classes(start_height_position:end_height_position,st
art_width_position:end_width_position,start_depth_position:end_depth_position,:)
...
            =allScores;

        end
```

```
    end
end

% Script is general for n classes to this point
out_Pmap_cat1 = out_scores_from_network_all_classes(:,:,:,1);
out_Pmap_cat2 = out_scores_from_network_all_classes(:,:,:,2);
out_Pmap_cat3 = out_scores_from_network_all_classes(:,:,:,3);

% Remove padding from probability maps
out_Uncertainty_Scores = out_Uncertainty_Scores(1:height, 1:width, 1:depth);
out_Pmap_cat1 = out_Pmap_cat1(1:height, 1:width, 1:depth);
out_Pmap_cat2 = out_Pmap_cat2(1:height, 1:width, 1:depth);
out_Pmap_cat3 = out_Pmap_cat3(1:height, 1:width, 1:depth);

%% visualise network outputs
figure(2)
clf(figure(2))
ax1 = subplot(2,3,1);
imshow(out_Pmap_cat1(:,:,60),[])
title('LF-nuclei')
ax2 = subplot(2,3,2) ;
imshow(out_Pmap_cat2(:,:,60),[])
title('LF-actin')
ax3 = subplot(2,3,3);
imshow(out_Pmap_cat3(:,:,60),[])
title('Background/other')
ax4 = subplot(2,3,5);
imshow(out_Uncertainty_Scores(:,:,60),[])
title('Uncertainty')
linkaxes([ax1 ax2 ax3 ax4], 'xy')

%% Map to 16-bit and save for loading into CellProfiler
ui16_PMAP_cat1_xy = uint16(65535*mat2gray(out_Pmap_cat1, [0 1])) ;
ui16_PMAP_cat2_xy = uint16(65535*mat2gray(out_Pmap_cat2, [0 1])) ;
ui16_PMAP_cat3_xy = uint16(65535*mat2gray(out_Pmap_cat3, [0 1])) ;
data_to_save = cat(4,ui16_PMAP_cat1_xy,ui16_PMAP_cat2_xy,ui16_PMAP_cat3_xy);

% Map to 16-bit and save for loading into CellProfiler
for loop = 1:3
    CP_channel_number = num2str(loop +2) ;

    MultiChImgTile = data_to_save(:,:,:,loop) ;

    % spec a fiji description
    fiji_descr = ['ImageJ=1.52p' newline ...
                'images=' num2str(size(MultiChImgTile,3)*...
                                  size(MultiChImgTile,4)*...
                                  size(MultiChImgTile,5)) newline...
                'channels=' num2str(size(MultiChImgTile,4)) newline...
                'slices=' num2str(size(MultiChImgTile,3)) newline...
                'frames=' num2str(size(MultiChImgTile,5)) newline...
                'hyperstack=true' newline...
                'mode=grayscale' newline...
                'loop=false' newline...
                'min=0.0' newline...
                'max=65535.0'];  % bitdepth spec

    %%%%%%%%%%%%%%%%%%%%%%%%%%%%%%%%%%%%%%%%%%%%%%%%%%%%%%%%%%%%%%%%%%%%%%%%%%%%
    t = Tiff(['Ch_',CP_channel_number,'_Im_','001','.tif'],'w') ;
    %%%%%%%%%%%%%%%%%%%%%%%%%%%%%%%%%%%%%%%%%%%%%%%%%%%%%%%%%%%%%%%%%%%%%%%%%%%%
```

```
    tagstruct.ImageLength = size(MultiChImgTile,1);
    tagstruct.ImageWidth = size(MultiChImgTile,2);
    tagstruct.Photometric = Tiff.Photometric.MinIsBlack;
    tagstruct.BitsPerSample = 16;
    tagstruct.SamplesPerPixel = 1;
    tagstruct.Compression = Tiff.Compression.None; %% lzw is not compatible w
CP4 out-of-box
    tagstruct.PlanarConfiguration = Tiff.PlanarConfiguration.Chunky;
    tagstruct.SampleFormat = Tiff.SampleFormat.UInt;
    tagstruct.ImageDescription = fiji_descr;

        for frame = 1:size(MultiChImgTile,5)
            for slice = 1:size(MultiChImgTile,3)
                for channel = 1:size(MultiChImgTile,4)
                    t.setTag(tagstruct)
                    t.write(im2uint16(MultiChImgTile(:,:,slice,channel,frame)));
                    t.writeDirectory(); % saves a new page in the tiff file
                end
            end
        end
        t.close()
end
```

Windows 10: Running the label-free cell segmentation deep learning scripts on an NVIDIA GPU using Python 3.6 and Tensorflow-gpu 1.9.0

In brief, it is recommended to use these deep learning files with:

- Python 3.6
- Tensorflow-gpu 1.9.0
- Keras 2.1.5
- Numpy 1.18.1
- Scipy 1.4.1
- Java SE Development Kit 11.0
- Python-bioformats 1.5.2
- CUDA Toolkit 9.0 / cuDNN v7.6.4

Installation Steps:

1. Install Visual Studio Express Community 2017

https://visualstudio.microsoft.com/dev-essentials/#software

- This is necessary to enable the install of the CUDA toolkit.
- At the link above, join Visual Studio Development Essentials (free sign-up).
- Use the search tool to find, download and install Visual Studio Express Community 2017.
- Using the recommended configurations at every step of the installation works fine.

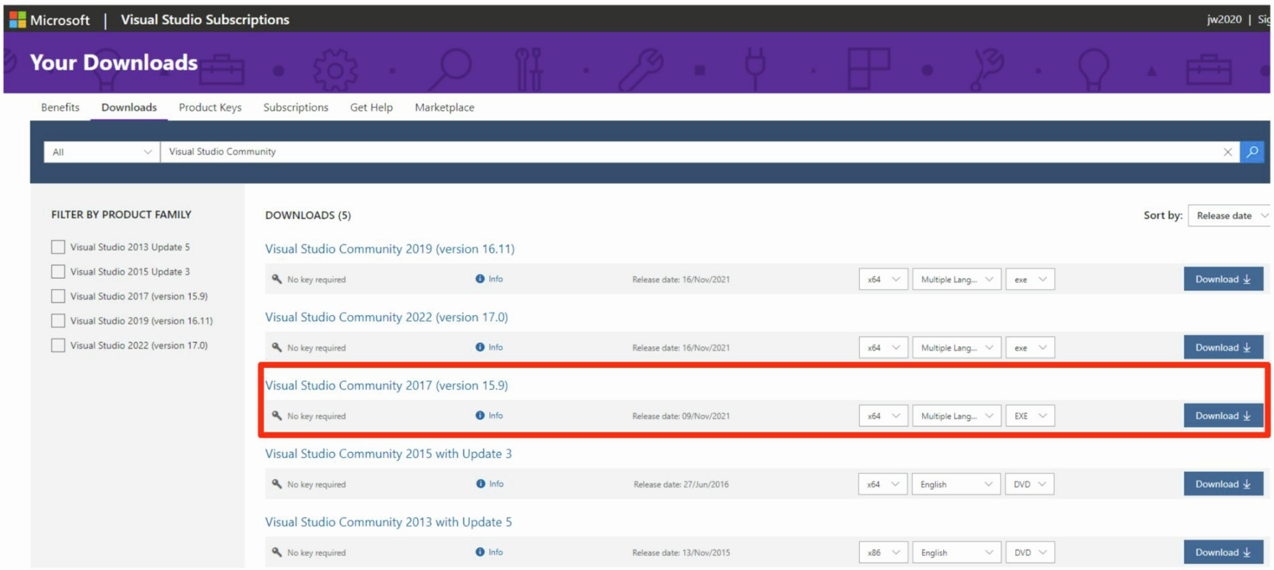

- Restarting your PC after installing Visual Studio is a probably a good idea.

2. Install CUDA Toolkit 9.0 and accompanying patches for Windows 10.

- Tensorflow-GPU 1.9.0 requires CUDA 9.0 - not whatever the latest version of the toolkit is.
- This is available at the Nvidia website "CUDA Toolkit Archive" --> select CUDA Toolkit 9.0

https://developer.nvidia.com/cuda-toolkit-archive

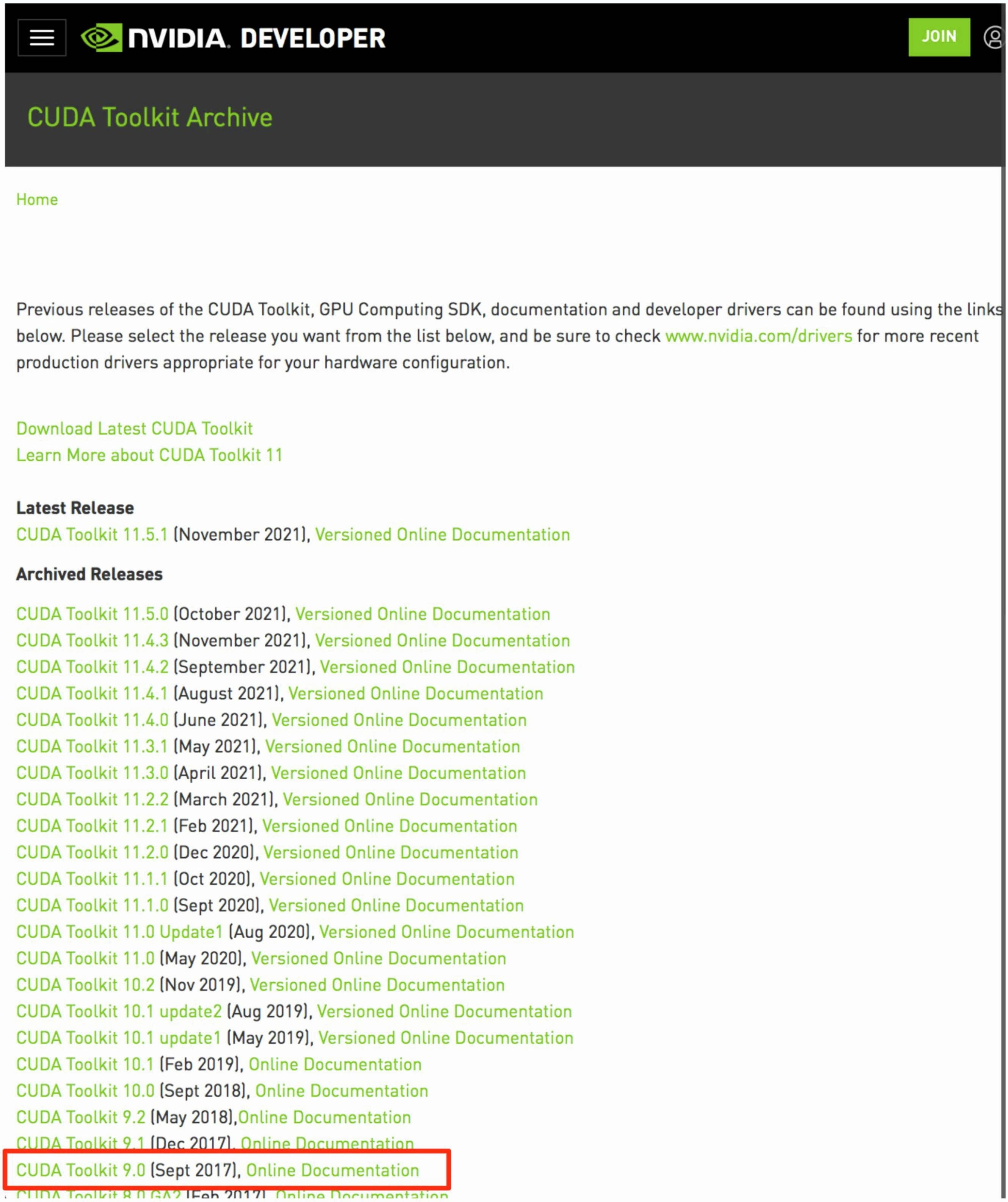

- Select the target platform as Windows, X86_64, version 10 and the installer type as exe(local)

Select Target Platform

Click on the green buttons that describe your target platform. Only supported platforms will be shown.

Operating System

Windows | Linux | Mac OSX

Architecture

x86_64

Version

10 | 8.1 | 7 | Server 2016 | Server 2012 R2

Installer Type

exe (network) | exe (local)

Download Installers for Windows 10 x86_64

The base installer is available for download below.
There are 4 patches available. These patches require the base installer to be installed first.

> Base Installer — Download (1.4 GB)

Installation Instructions:

1. Double click cuda_9.0.176_win10.exe
2. Follow on-screen prompts

> Patch 1 (Released Jan 25, 2018) — Download (54.1 MB)

cuBLAS Patch Update: This update to CUDA 9.0 includes new GEMM kernels optimized for the Volta architecture and improved heuristics to select GEMM kernels for given input sizes.

> Patch 2 (Released Mar 5, 2018) — Download (54.7 MB)

cuBLAS Patch Update: This update to CUDA 9 includes GEMM heuristics improvements to selects the most optimized algorithms for input sizes commonly used in Deep Learning RNNs. The update also includes other bug-fixes and performance enhancements.

> Patch 3 (Released Jun 7, 2018) — Download (82.3 MB)

cuBLAS Patch Update: This update to cuBLAS addresses issues with Convolutional Seq2Seq and RNN inference performance.

> Patch 4 (Released Aug 6, 2018) — Download (56.2 MB)

cuBLAS Patch Update: This update to cuBLAS includes optimized implementations of GEMV operations for mixed precision input and output types and important fixes to address performance issues.

The checksums for the installer and patches can be found in Installer Checksums.
For further information, see the Installation Guide for Microsoft Windows and the CUDA Quick Start Guide.

- First install the "base installer" following the standard, "express configurations" options.

- Then install Patches 1-4 sequentially in order by just following the on-screen prompts after each download.

3. Install cuDNN v7.6.4 for CUDA 9.0

- To download and install cuDNN, first join the NVIDIA Developer Program – which is free. https://developer.nvidia.com/cudnn-download-survey

- Once signed in, proceed the cuDNN download page and click archived cuDNN releases: https://developer.nvidia.com/rdp/cudnn-archive

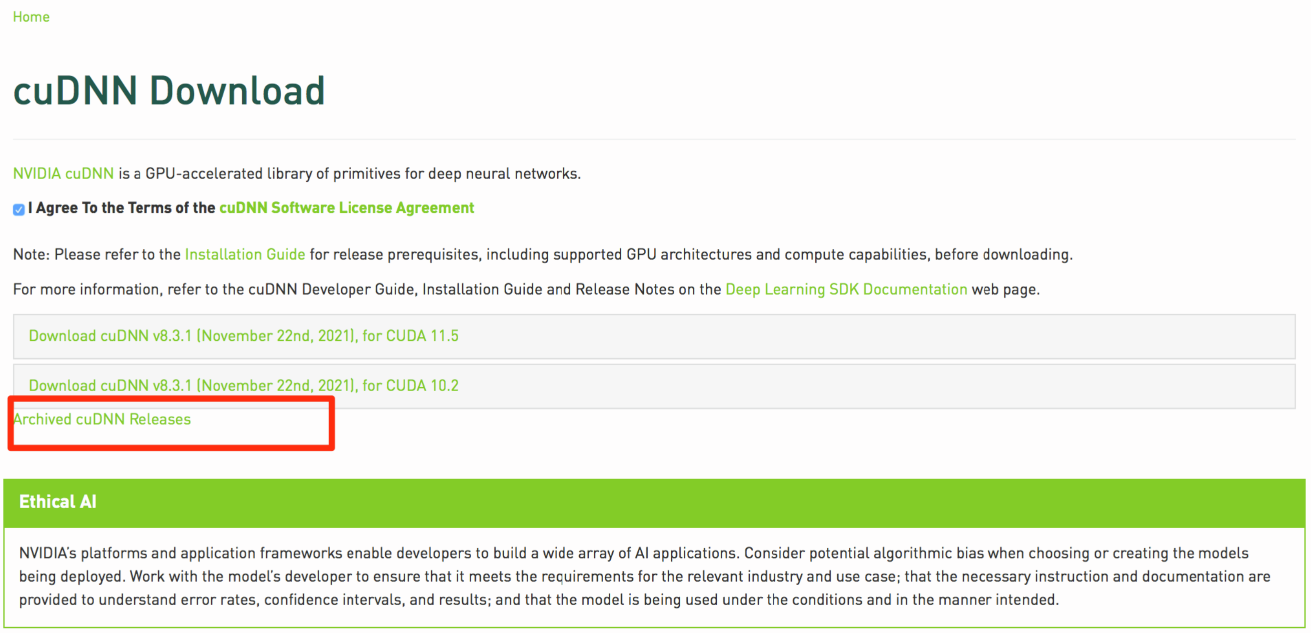

- Scroll down to the option to download cuDNN v7.6.4 for CUDA 9.0 and download the library for Windows 10.

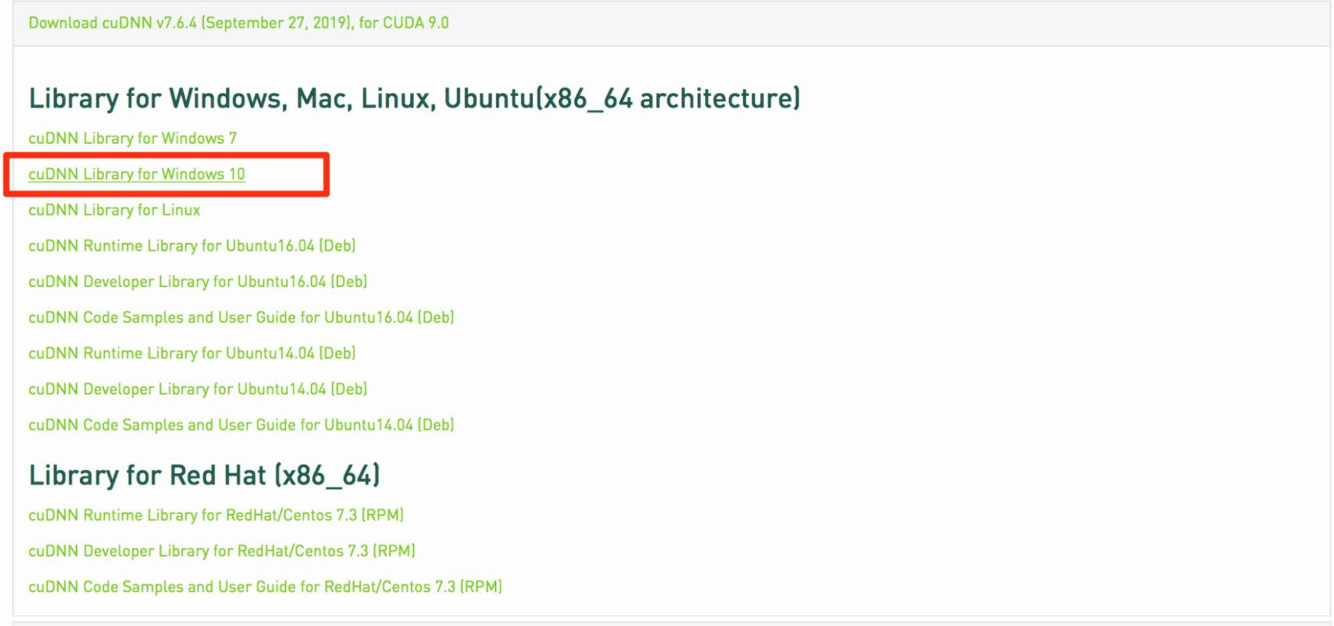

- Unzip the downloaded cuDNN .zip file.

- Inside are three files which need to be copied to the correct folder subdirectories of your Window 10 installation of the CUDA 9.0 toolkit:

- These files are **cudnn64_7.dll, cudnn.h** and **cudnn.lib**:

1. cudnn64_7.dll
Copy the file from the unzipped cuDNN download folder at e.g.,
<unzipped_download>/cudnn-9.0-windows10-x64-v7.6.2.24\cuda\bin\cudnn64_7.dll

and paste it into the NVIDIA GPU computing toolkit v9.0 subdirectory 'bin' located at e.g.,
C:\Program Files\NVIDIA GPU Computing Toolkit\CUDA\v9.0\bin\

2. cudnn.h
Copy the file from the unzipped cuDNN download folder at e.g.,
<unzipped_download>/cudnn-9.0-windows10-x64-v7.6.2.24\cuda\ include\cudnn.h

and paste it into the NVIDIA GPU computing toolkit v9.0 subdirectory 'include' located at e.g.,
C:\Program Files\NVIDIA GPU Computing Toolkit\CUDA\v9.0\include\

3. cudnn.lib
Copy the file from the unzipped cuDNN download folder at e.g.,
<unzipped_download>/cudnn-9.0-windows10-x64-v7.6.2.24\cuda\lib\x64\cudnn.lib

and paste it into the NVIDIA GPU computing toolkit v9.0 subdirectory 'include' located at e.g.,
C:\Program Files\NVIDIA GPU Computing Toolkit\CUDA\v9.0\lib\x64\

4. Check that the CUDA environment variables are set in Windows 10

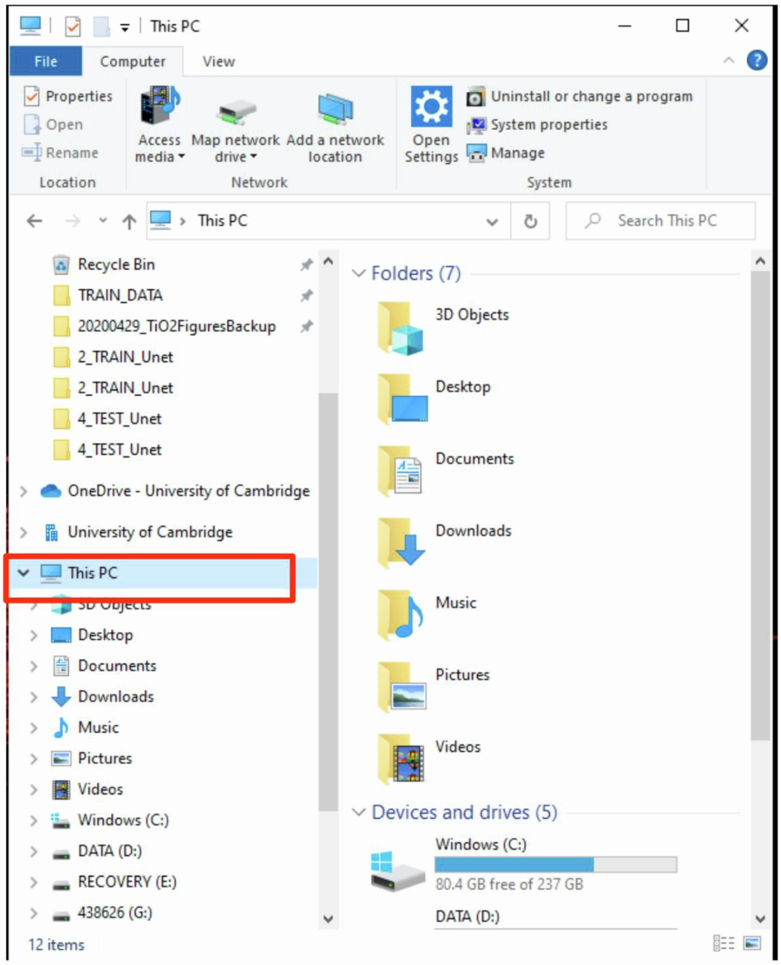

- From File Explorer, right-click on “This PC” on the left-side and select “Properties”

- On the right-side, of the dialogue that opens, click “Advanced system settings”

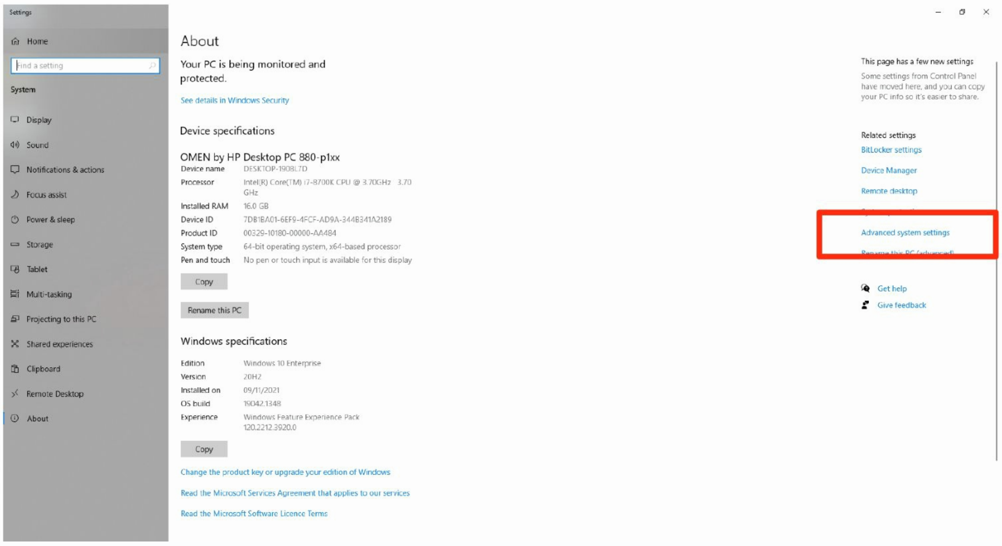

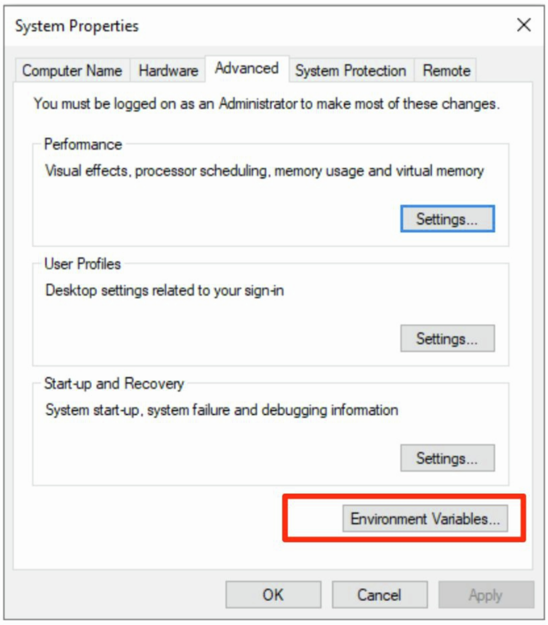

- In the “System Properties” dialogue box, click “Environment Variables” at the bottom

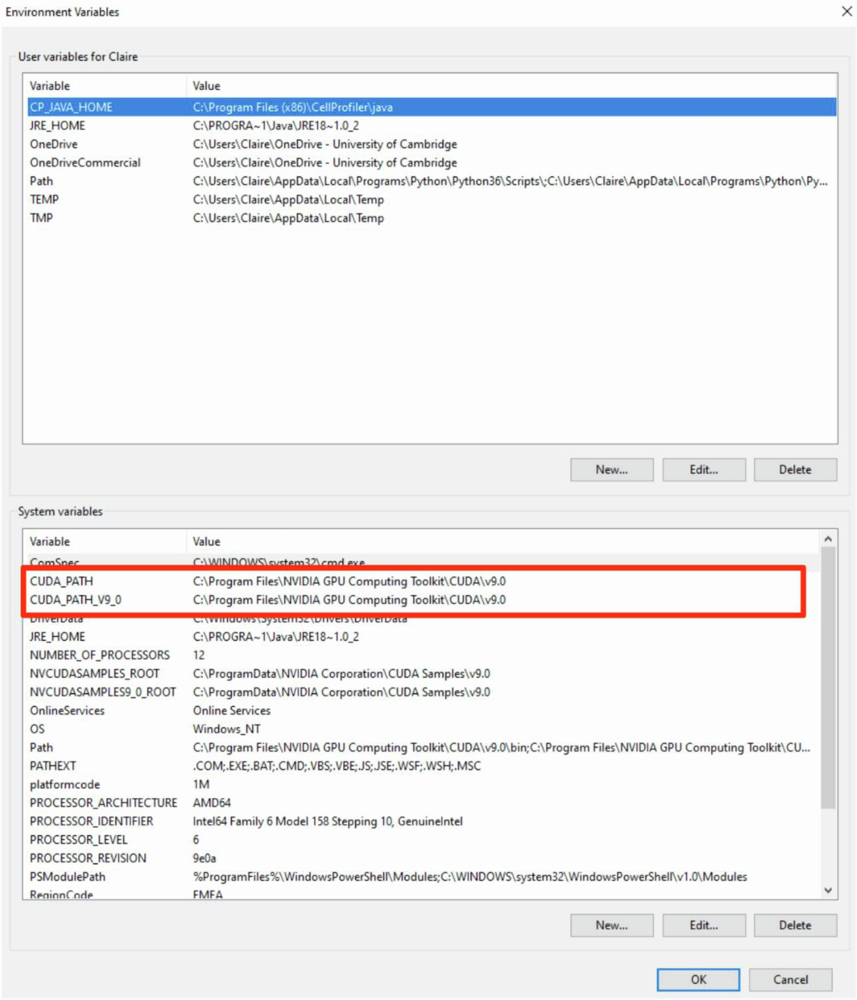

- Make sure in the bottom window (labelled "System Variables") that variables named CUDA_PATH and CUDA PATH V9.0 exist and point to the correct locations e.g.,

C:\Program Files\NVIDIA GPU Computing Toolkit\CUDA\v9.0\bin

and
C:\Program Files\NVIDIA GPU Computing Toolkit\CUDA\v9.0\libnvvp

respectively.

- These paths should be auto-installed. If they are missing on your system, they can be added by clicking the "New" button below the system variables bottom panel and entering the name and path into the dialogue box for your system.

5. Install Python 3.6.8

- To install Python version 3.6.8 navigate to the previous release at:
https://www.python.org/downloads/release/python-368/

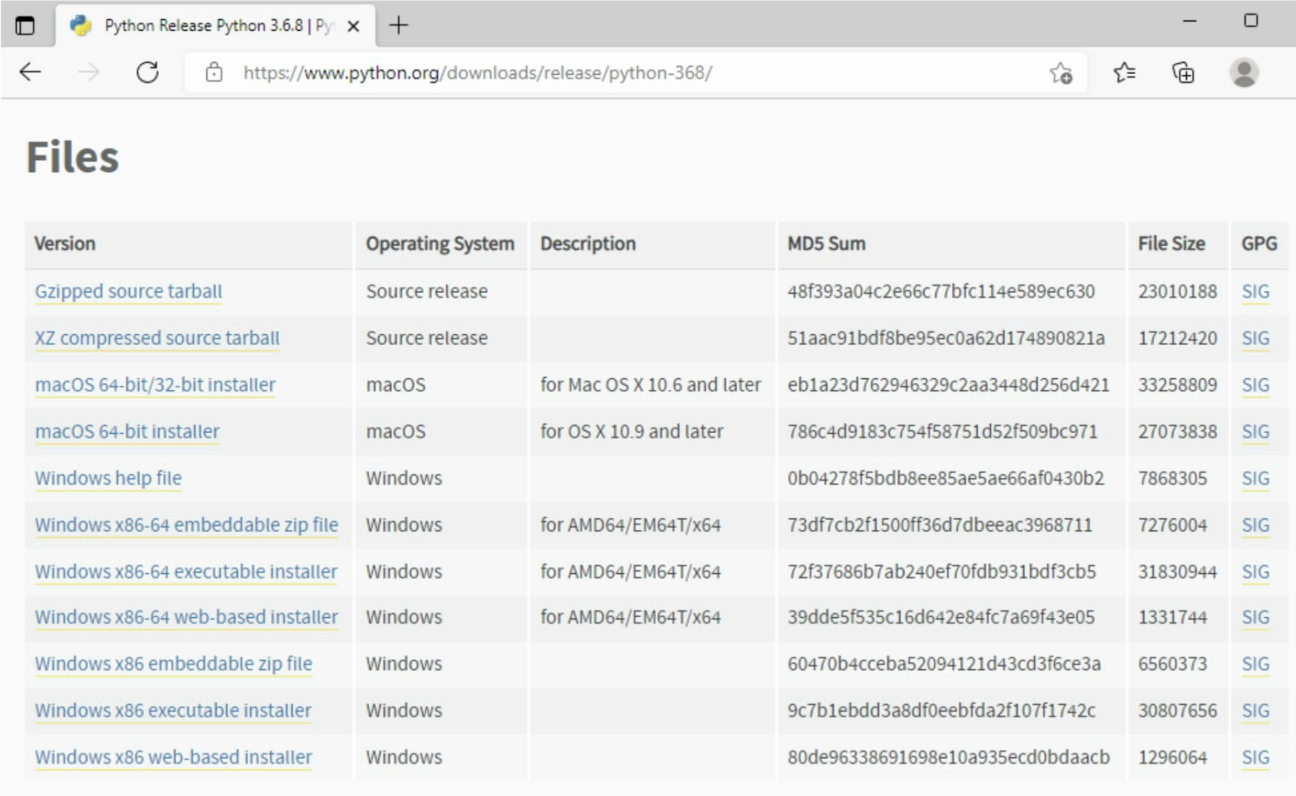

Python Release Python 3.6.8 | Py
https://www.python.org/downloads/release/python-368/

Files

Version	Operating System	Description	MD5 Sum	File Size	GPG
Gzipped source tarball	Source release		48f393a04c2e66c77bfc114e589ec630	23010188	SIG
XZ compressed source tarball	Source release		51aac91bdf8be95ec0a62d174890821a	17212420	SIG
macOS 64-bit/32-bit installer	macOS	for Mac OS X 10.6 and later	eb1a23d762946329c2aa3448d256d421	33258809	SIG
macOS 64-bit installer	macOS	for OS X 10.9 and later	786c4d9183c754f58751d52f509bc971	27073838	SIG
Windows help file	Windows		0b04278f5bdb8ee85ae5ae66af0430b2	7868305	SIG
Windows x86-64 embeddable zip file	Windows	for AMD64/EM64T/x64	73df7cb2f1500ff36d7dbeeac3968711	7276004	SIG
Windows x86-64 executable installer	Windows	for AMD64/EM64T/x64	72f37686b7ab240ef70fdb931bdf3cb5	31830944	SIG
Windows x86-64 web-based installer	Windows	for AMD64/EM64T/x64	39dde5f535c16d642e84fc7a69f43e05	1331744	SIG
Windows x86 embeddable zip file	Windows		60470b4cceba52094121d43cd3f6ce3a	6560373	SIG
Windows x86 executable installer	Windows		9c7b1ebdd3a8df0eebfda2f107f1742c	30807656	SIG
Windows x86 web-based installer	Windows		80de96338691698e10a935ecd0bdaacb	1296064	SIG

- Scroll to the bottom of the page and download the "Windows x86-64 executable installer" with description "AMD64/EM64T/x64".

- Once downloaded, follow the on-screen prompts to install Python 3.6.8.

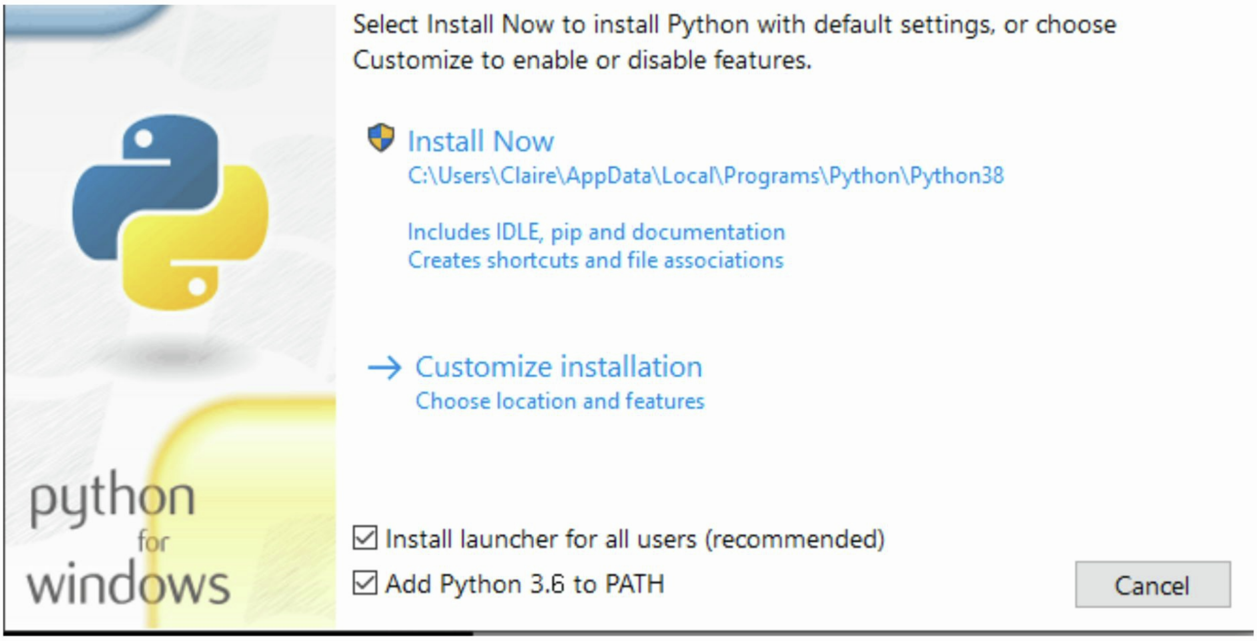

- Check the box to add Python to the Windows path.

6. Install Java Development Kit 11
- This is used by Python-bioformats to enable read/write of image-data.
- Download and install Java Development kit 11
https://www.oracle.com/java/technologies/downloads/#java11

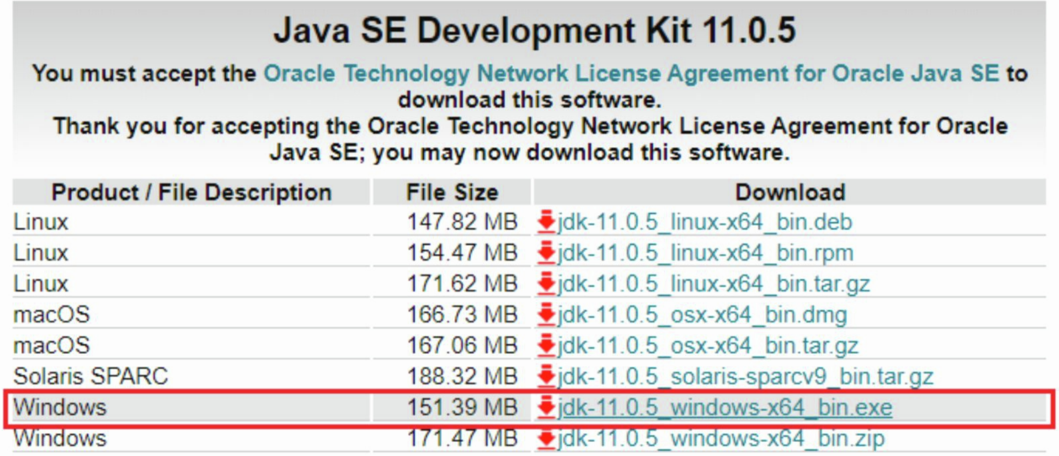

Java SE Development Kit 11.0.5

You must accept the Oracle Technology Network License Agreement for Oracle Java SE to download this software.
Thank you for accepting the Oracle Technology Network License Agreement for Oracle Java SE; you may now download this software.

Product / File Description	File Size	Download
Linux	147.82 MB	jdk-11.0.5_linux-x64_bin.deb
Linux	154.47 MB	jdk-11.0.5_linux-x64_bin.rpm
Linux	171.62 MB	jdk-11.0.5_linux-x64_bin.tar.gz
macOS	166.73 MB	jdk-11.0.5_osx-x64_bin.dmg
macOS	167.06 MB	jdk-11.0.5_osx-x64_bin.tar.gz
Solaris SPARC	188.32 MB	jdk-11.0.5_solaris-sparcv9_bin.tar.gz
Windows	151.39 MB	jdk-11.0.5_windows-x64_bin.exe
Windows	171.47 MB	jdk-11.0.5_windows-x64_bin.zip

- Once Java SE Development Kit 11 is installed, set the **JAVA_HOME** environment variable and add the Java development kit to the Windows path. To do this, as above, open a File Explorer window, right-click on the '**This PC**' option and select '**Properties**' from the drop-down menu.

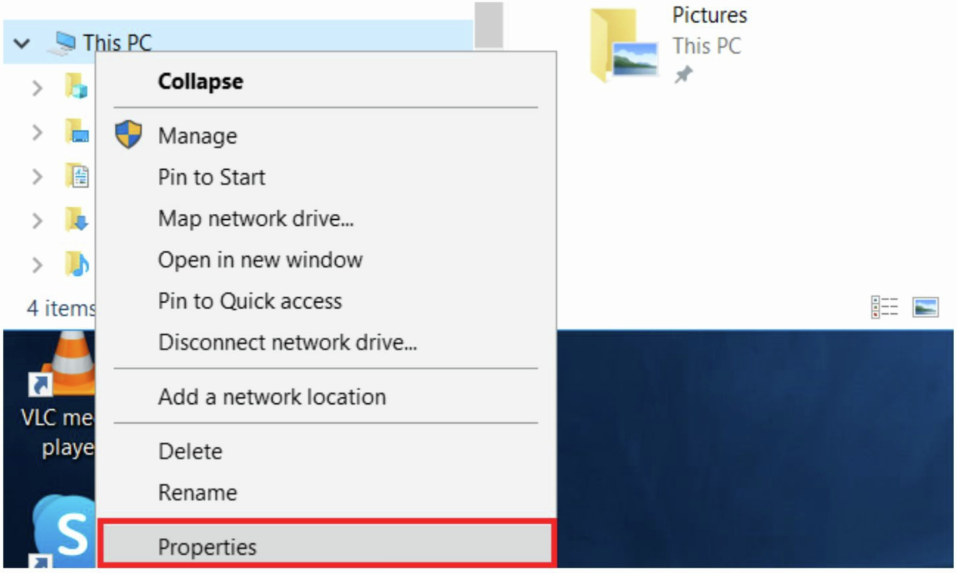

The control panel will pop up as a separate window. Select '**Advanced system settings'** from the list appearing at the right of the window.

In the 'system properties' dialogue that opens, select '**Environment Variables**'. This will cause the environment variables window to appear. To set the 'JAVA_HOME' variable, Click the '**New**' button option at the bottom of this window in the '<u>System variables'</u> section.

Name the new variable 'JAVA_HOME' and use the '**Browse Directory**' option to specify the path to JDK 11. Select '**OK**' to create this new variable:

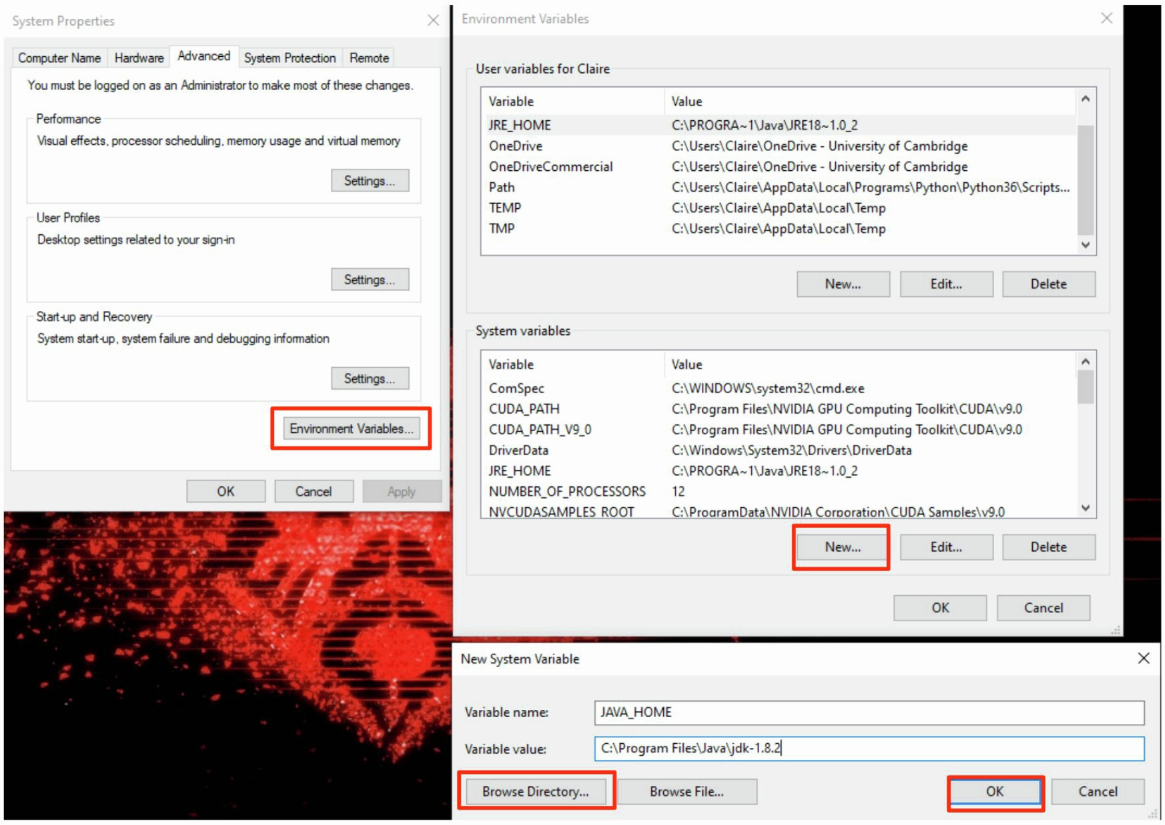

To add JDK11 to the Windows path, Click on the exisiting ‘Path’ system variable and click the ‘**Edit**’ button. A new window will appear. Click new and type ‘**%JAVA_HOME%\bin**’.

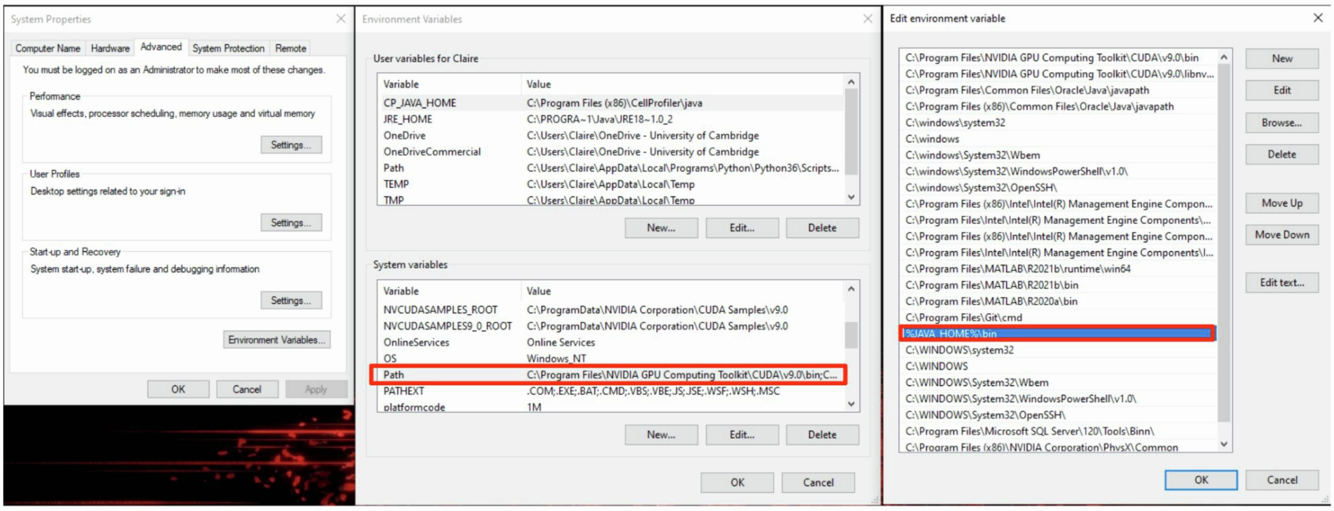

- Click ‘**OK**’ to apply changes and close all opened windows.

7. Setup a virtual environment and install the dependencies necessary to run the code

- Download and unzip the "Python_biostudies" folder from the BioStudies project archive to a suitable location on your computer.

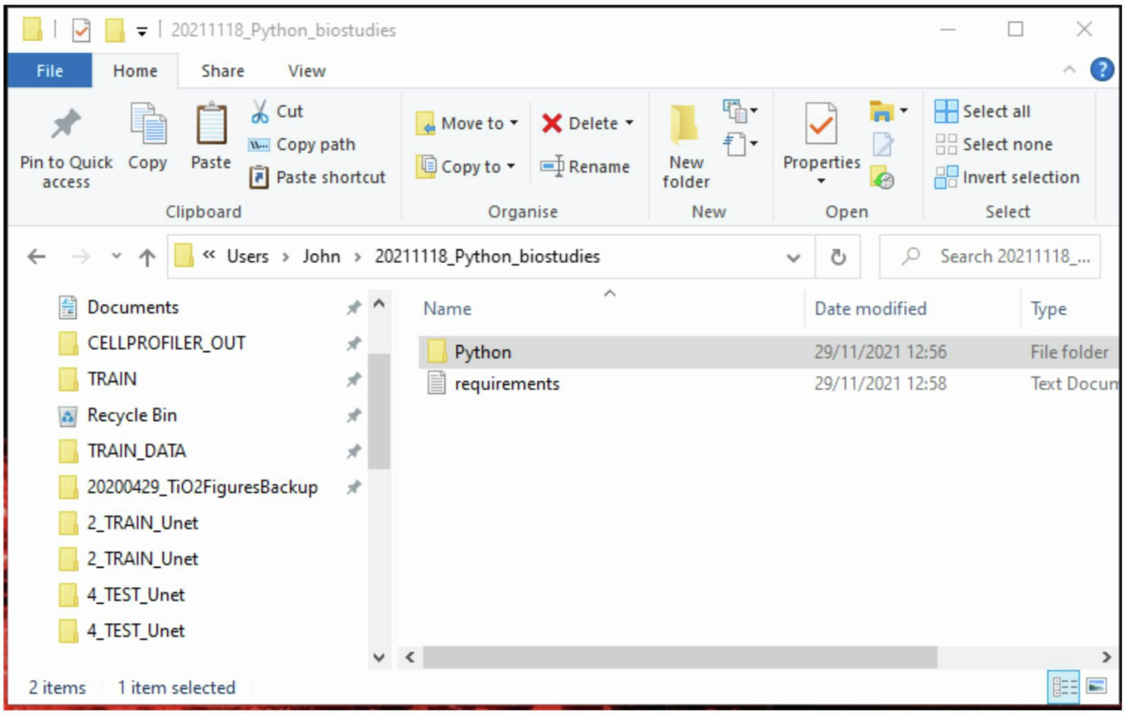

- Run command prompt as administrator:
- To do this, Search "CMD", right-click on "Command Prompt" from the search results dialogue and click "Run as administrator":

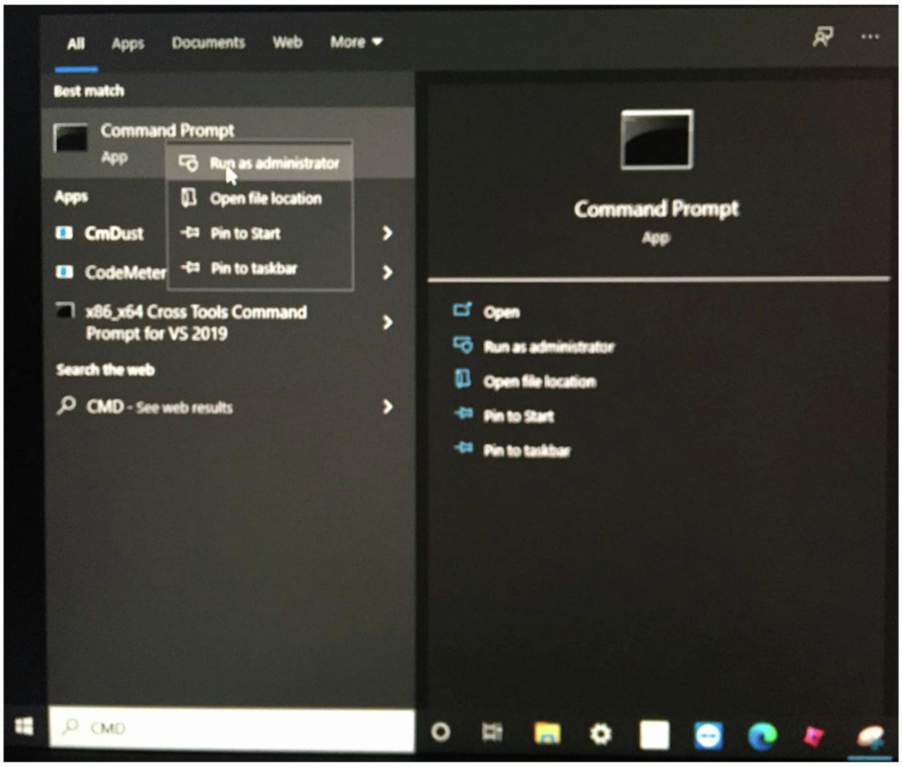

- Using the ‘cd’ command, change the current directory to the unzipped python scripts folder downloaded from Biostudies e.g.,

```
Administrator: Command Prompt
Microsoft Windows [Version 10.0.19042.1348]
(c) Microsoft Corporation. All rights reserved.

C:\WINDOWS\system32>cd C:\Users\John\20211118_Python_biostudies
```

- Make a new subdirectory ‘venv’ and initialise an instance of Python inside it e.g.,

```
C:\Users\John\20211118_Python_biostudies>python -m venv ./venv
```

- Activate the newly-created virtual environment e.g.,

```
C:\Users\John\20211118_Python_biostudies>venv\Scripts\activate.bat
```

- Update pip to the latest version (N.B., this is critical to the successful install of javabridge with Python 3.6)

```
(venv) C:\Users\John\20211118_Python_biostudies>python -m pip install --upgrade pip
```

- Using the “requirements.txt” file included in the BioStudies download, install the required dependencies and the Spyder 4.0 Integrated Development Environment (IDE) to the newly-created virtual environment e.g.,

```
(venv) C:\Users\John\20211118_Python_biostudies>pip install -r requirements.txt
```

8. Training a 2-D UNET model

- A screencast video is included with the BioStudies project archive.

- Download and unzip the Python BioStudies project archive at a suitable location on your computer.

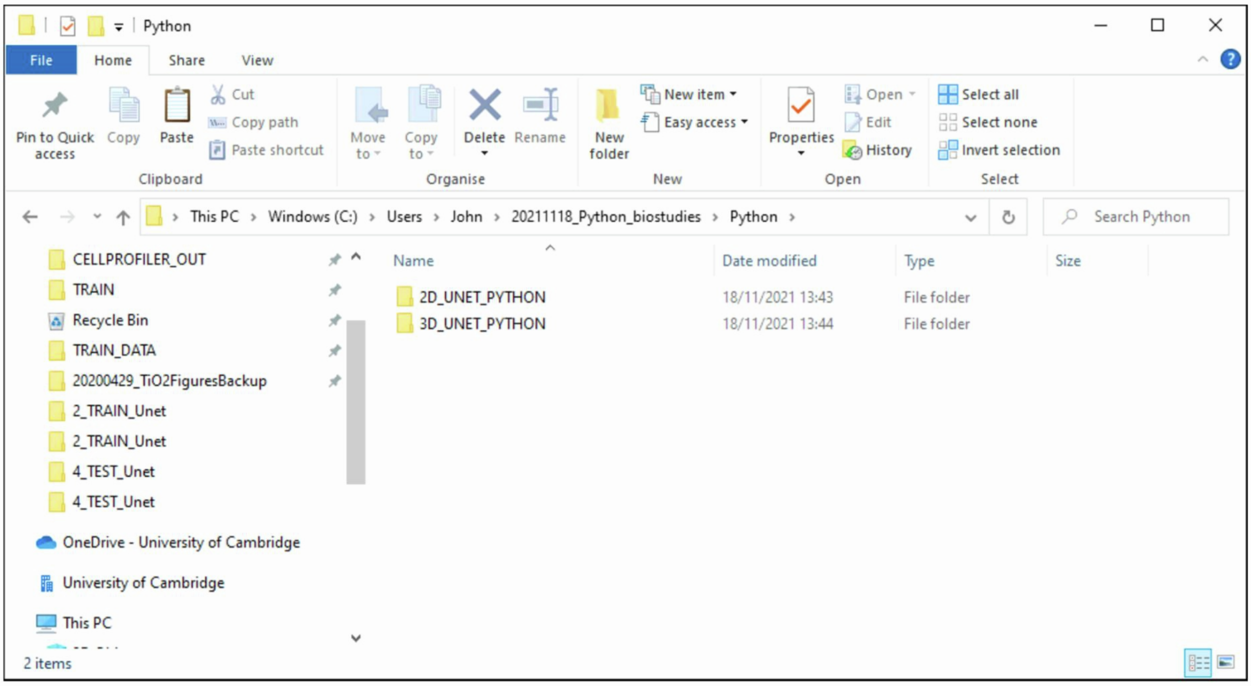

- Inside the 2D_UNET_Python folder, there is a sequentially-organised workflow starting with raw data and ending with a CellProfiler pipeline that obtains cell features from the label-free cell segmentation:

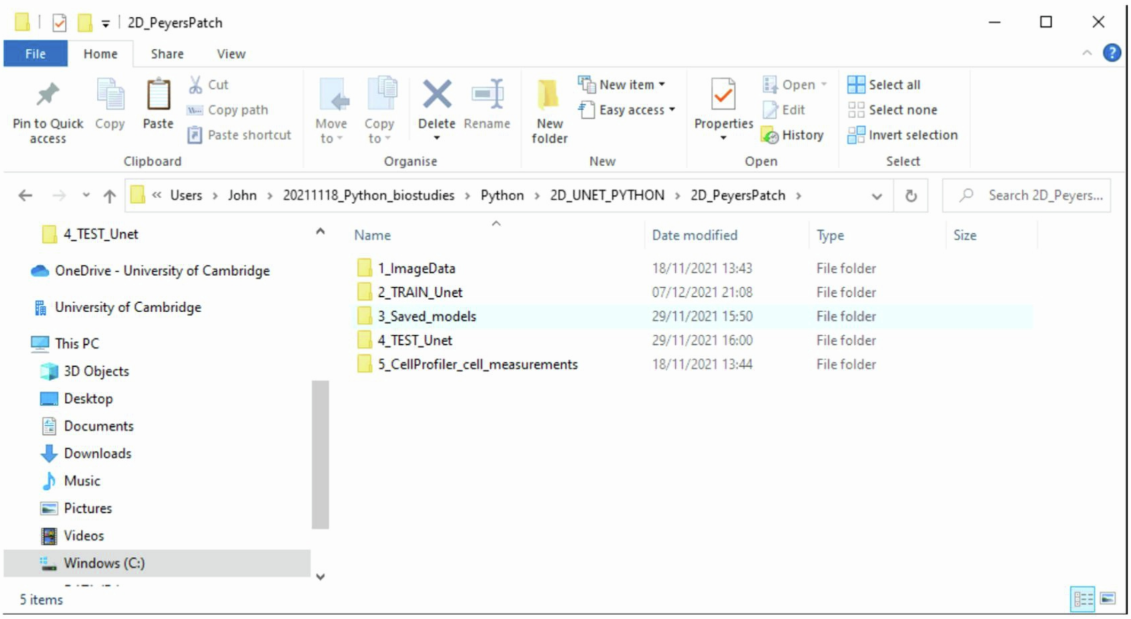

- In command prompt with the virtual environment active, type 'Spyder' to start the Spyder4 IDE.

```
(venv) C:\Users\John\20211118_Python_biostudies>spyder
```

- To train a 2-D UNET model, set the Spyder working directory to the “2_TRAIN_Unet” directory. Open the “A_TRAIN_UNET” script.

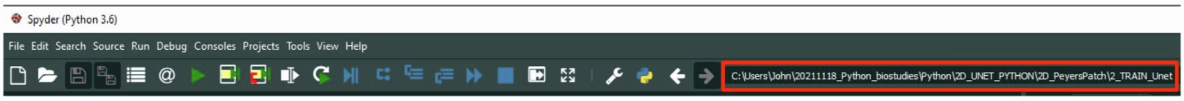

- Update the path to the training data (located in the “1_ImageData” folder).
- Update the path to the pixel classification labels (located in the “1_ImageData” folder)

```
# Read in the reflectance data for training
Training_data_holder = skimage.io.imread('C:/Users/John/20211118_Python_biostudies/Python/2D_UNET_PYTHON/2D_PeyersPatch/1_ImageData/TRAIN_IMAGE.tif')
Training_data = Training_data_holder[0,0,:,:,2]

#Read in the mask to isolated just the lymphoid tissue
mask = Training_data_holder[0,0,:,:,3]
thresh = threshold_otsu(Training_data)
mask = mask > thresh
mask = mask*1
mask = np.uint16(mask)

# Apply the mask to the training data
Training_data = np.multiply(Training_data,mask);
Training_data = np.double(Training_data);

# Rescale the training data in the interval [0 1]
Training_data_norm_holder = np.zeros((8551, 5701),np.double)
Training_data_norm = cv2.normalize(Training_data, Training_data_norm_holder , 1.0, 0.0, cv2.NORM_MINMAX)

#Read in the pixel-class labels created from the nuclei and actin staining
Training_labels_holder = skimage.io.imread('C:/Users/John/20211118_Python_biostudies/Python/2D_UNET_PYTHON/2D_PeyersPatch/1_ImageData/TRAIN_LABELS.png');
Training_labels = np.where(Training_labels_holder == 3, 0, Training_labels_holder)
```

- Update the path saving the training data and pixel classification labels in numpy format.
- This should be inside the”2_TRAIN_Unet” directory.

```
#%% Once happy with labels and data, commit the reflectance information data to sub-directory in .npy format

MYDIR = 'C:/Users/John/20211118_Python_biostudies/Python/2D_UNET_PYTHON/2D_PeyersPatch/2_TRAIN_Unet/npy_training_data/'
CHECK_FOLDER = os.path.isdir(MYDIR)

# If folder doesn't exist, then create it.
if not CHECK_FOLDER:
    os.makedirs(MYDIR)

np.save(os.path.join(MYDIR, 'Training_data'), Training_data_norm)
```

- The script will save the model along with checkpoint values taken during training. You should specify where these files are to be saved. This should be folder 3 of the workflow: (“3_Saved_models”).

```
#%% Define path to safe network, time stamp network
checkpoint_filepath = 'C:/Users/John/20211118_Python_biostudies/Python/2D_UNET_PYTHON/2D_PeyersPatch/3_Saved_models/'
now = datetime.now()
dt_string = now.strftime("%d/%m/%Y%H:%M:%S")
NAME =dt_string
Name_file = 'PeyersPatchBiostudies_'
checkpoint_filepath_name = checkpoint_filepath + Name_file+dt_string


checkpoint_dir =  os.path.dirname(checkpoint_filepath_name)

#Specify that weights are to be saved
model_checkpoint_callback = tensorflow.keras.callbacks.ModelCheckpoint(
    filepath=checkpoint_dir,
    save_weights_only=True,
    monitor='val_accuracy',
    mode='max',
    save_best_only=True)

# Specify training options
BATCH_SIZE = 12
NUM_EPOCHS = 50

#Augment training data
train_generator = get_train_augmented(trainX=trainX, trainY=trainY, BATCH_SIZE=BATCH_SIZE)

#Train and save the network
history = model.fit_generator(train_generator, steps_per_epoch=len(trainX)/(BATCH_SIZE*2), epochs=NUM_EPOCHS, callbacks=[model_checkpoint_callback])

#Plot progress # this plots over the view of the data
plt.figure(2)
plt.plot(history.history['sparse_categorical_accuracy'])
plt.title('Model accuracy')
plt.ylabel('accuracy')
plt.xlabel('epoch')
plt.legend(['train', 'test'], loc='upper left')
plt.savefig('Training_plot.png')
plt.show()

#Save final fully trained model as a .h5 file
model.save("C:/Users/John/20211118_Python_biostudies/Python/2D_UNET_PYTHON/2D_PeyersPatch/3_Saved_models/my_h5_model.h5")
checkpoint_filepath_name_h5 = checkpoint_filepath + Name_file+dt_string+'.h5'
model.save(checkpoint_filepath_name_h5)
```

- To start model training, click the green arrow button at the top of the Spyder dialogue or type the script name at the command line.

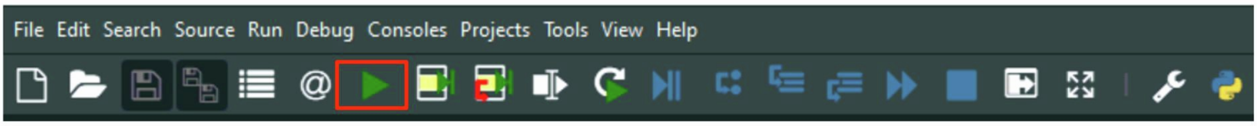

- Model training takes several hours (~ 4h on a NVIDIA GTX 1080 Ti GPU)
- The newly-trained model will be saved in the "3_Saved_models" directory.

9. Testing a pretrained 2-D model using unseen data
- A screencast video is included with the BioStudies project archive.
- Change the Spyder working directory to the "4_Test_Unet" directory. Open the "A_TEST_UNET" script.

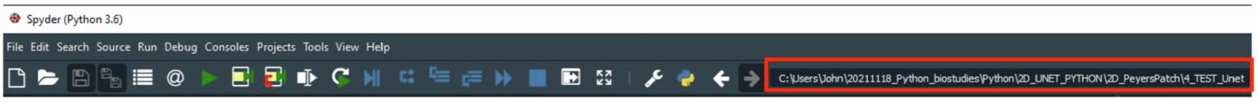

- Change the path to the unseen test image (located in the "1_ImageData" folder).

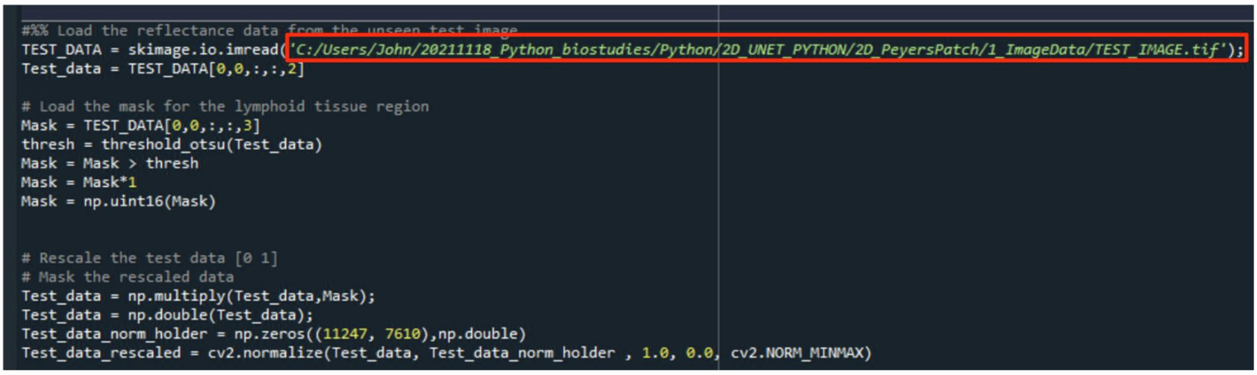

- Specify which pretrained network to use (pretrained networks are located in the "3_Saved_models" folder

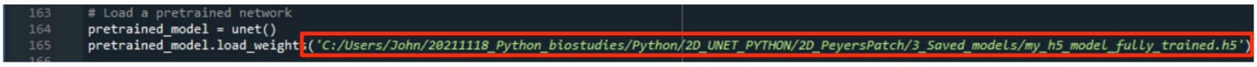

- Clicking the green arrow button or type the file name at the command line to process the unseen reflectance data with the selected pretrained network:

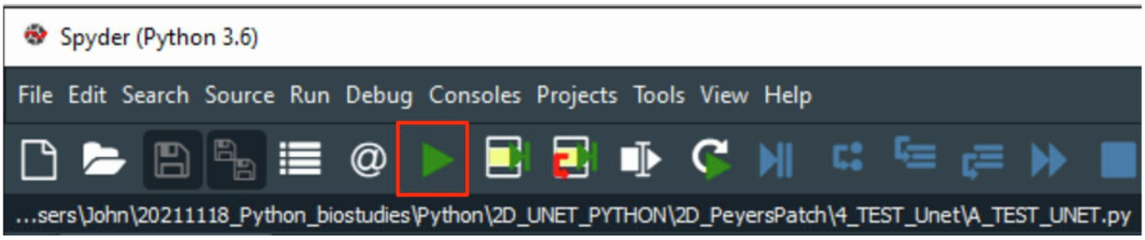

- Probability maps for the LF-nuclei, LF-actin and Background/Other classes will be saved in the working directory named ready for input into the CellProfiler pipeline (filenames Ch_5_lm_001, Ch_6_lm_001, Ch_7_lm_001, respectively).

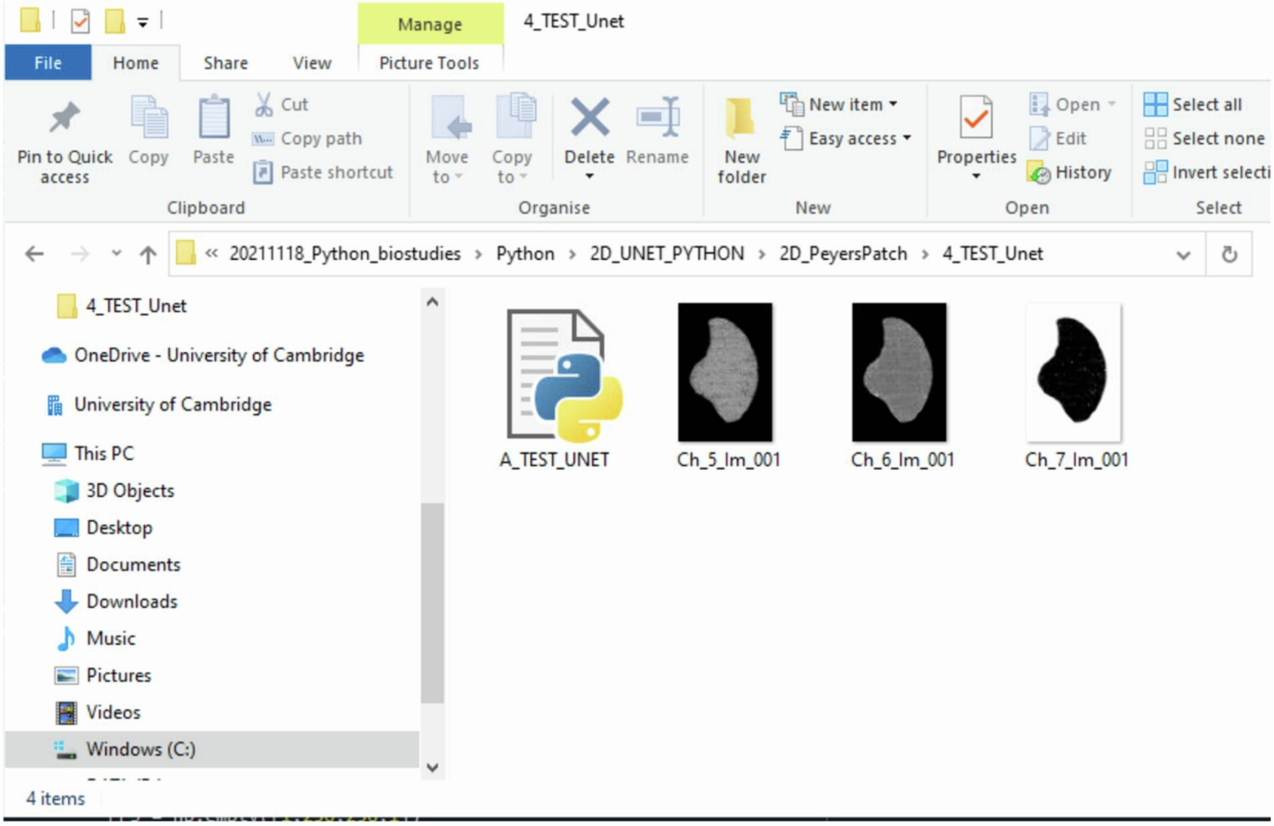

10. Training a 3-D UNET Model

- A screencast video is included with the BioStudies project archive.

- Inside the 3D_UNET_Python folder, there is a sequentially-organised workflow starting with raw data and ending with a CellProfiler pipeline that obtains 3-D cell features from the label-free cell segmentation:

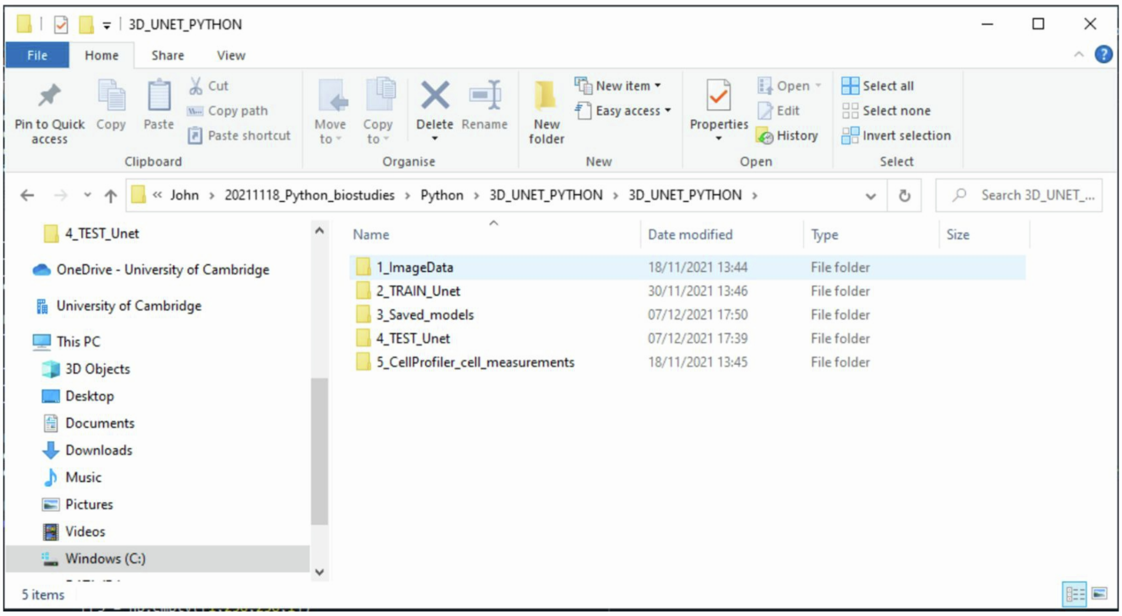

- To train a 3-D UNET model, open Spyder and set the working directory to the “2_TRAIN_Unet” directory. Open the “A_TRAIN_UNET_3D” script.

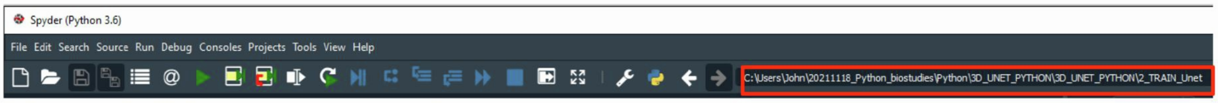

- Specify the path to the training data (located in the “1_ImageData/TRAIN/DATA” folder).
- Specify the channel number in the training data that contains the reflectance information (here ‘3’).
- Specify the location of the pixel-classification training labels (located at “1_ImageData/TRAIN/LABELS” folder).
- Specify the directory where the training data should be saved in Numpy format.

```
#%% Specify directory containing training data
Training_data_directory = 'C:/Users/John/20211118_Python_biostudies/Python/3D_UNET_PYTHON/3D_UNET_PYTHON/1_ImageData/TRAIN/DATA/'

# Specify channel number in training data that contains reflectance information
RL_training_directory = 3;

# Specify directory containing training labels
Training_labels_path = 'C:/Users/John/20211118_Python_biostudies/Python/3D_UNET_PYTHON/3D_UNET_PYTHON/1_ImageData/TRAIN/LABELS/' ;


# COMMIT TRAINING DATA TO DIRECTORY rescaled [0 1] IN MAT FORMAT
MYDIR ='C:/Users/John/20211118_Python_biostudies/Python/3D_UNET_PYTHON/3D_UNET_PYTHON/1_ImageData/TRAIN/LABELS/mat_training_data/'
CHECK_FOLDER = os.path.isdir(MYDIR)

# If folder doesn't exist, then create it.
if not CHECK_FOLDER:
    os.makedirs(MYDIR)
```

- Specify the checkpoint path during model training:

```
#%% Specify location to save the network
checkpoint_filepath = 'C:/Users/John/20211118_Python_biostudies/Python/3D_UNET_PYTHON/3D_UNET_PYTHON/3_Saved_models/'
now = datetime.now()
dt_string = now.strftime("%d/%m/%Y%H:%M:%S")
NAME =dt_string
Name_file = 'PeyersPatchBiostudies_'
checkpoint_filepath_name = checkpoint_filepath + Name_file+dt_string
```

- Specify the location to save the model when training completes. This should be the “3_Saved_models” directory.

```
#%% Train the model
history = model.fit(
    storeX,
    storeY,
    batch_size = BATCH_SIZE,
    epochs = NUM_EPOCHS,callbacks=[model_checkpoint_callback])

plt.plot(history.history['sparse_categorical_accuracy'])
plt.title('Model accuracy')
plt.ylabel('accuracy')
plt.xlabel('epoch')
plt.legend(['train', 'test'], loc='upper left')
plt.savefig('Training_plot.png')
plt.show()

model.save("C:/Users/John/20211118_Python_biostudies/Python/3D_UNET_PYTHON/3D_UNET_PYTHON/3_Saved_models/my_h53D_model.h5"
```

- Click “Run” to commence model training.
- This takes considerable time (approximately 6h on a NVIDIA GTX 1080 Ti GPU card).

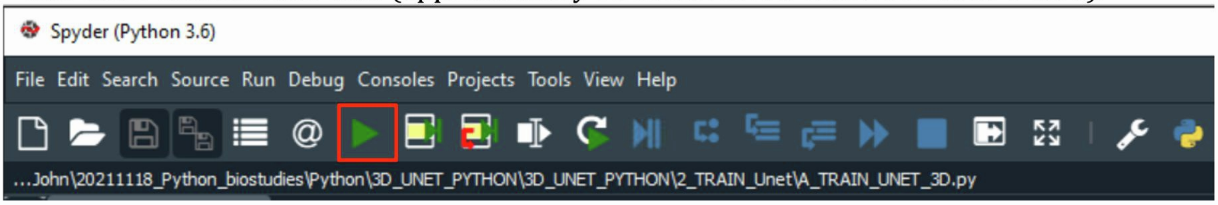

11. Testing a pretrained 3-D UNET Model using unseen data

- A screencast video is included with the BioStudies project archive.

- Change the Python working directory to the “4_Test_Unet” directory. Open the “A_TEST_UNET_3D” script.

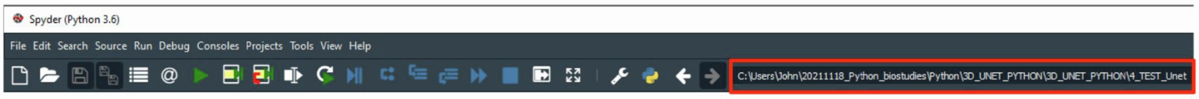

- Specify the location of the unseen test image-data. This is located inside the “1_ImageData” folder at “1_ImageData/TEST/”.
- Specify the channel containing the reflectance information (here, ‘2’).
- Specify where the unseen data should be stored upon conversion to Numpy format. This should be inside the “4_Test_Unet” folder.

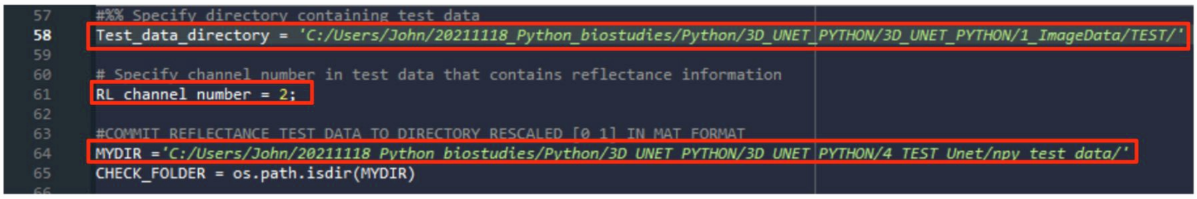

- Specify a pretrained 3-D Unet model from the “3_Saved_models” directory.

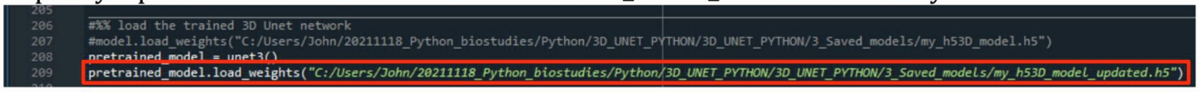

- Click the green arrow to process the unseen reflectance data with the 3-D Unet model.

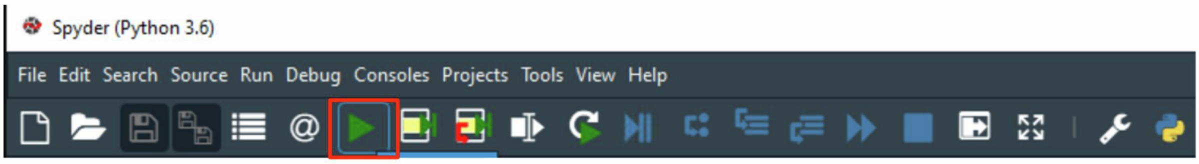

- Probability maps for the LF-nuclei, LF-actin and Background/Other classes will be saved as multipage .TIFF files in the working directory named ready for input into the CellProfiler pipeline (filenames Ch_3_lm_001, Ch_4_lm_001, Ch_5_lm_001, respectively).

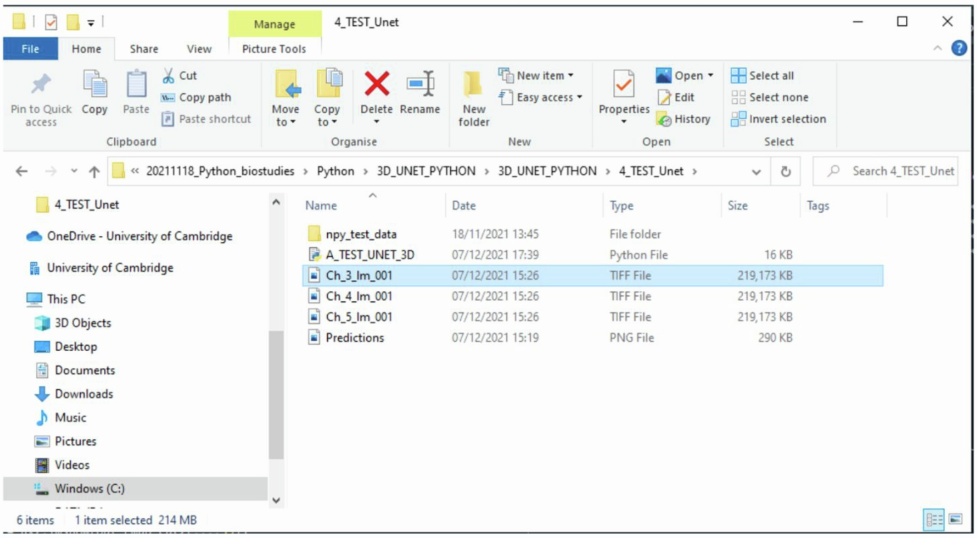

```
% PYTHON SCRIPT: TRAIN 2D UNET
% All code, image-data and screen-cast tutorial videos available for download at
% the Biostudies database under accession number S-BSST742


# -*- coding: utf-8 -*-
"""
Created on Mon Sep 20 23:58:04 2021

@author: Paul
"""
################################################################################
#######################################################
#Import all required modules
################################################################################
#######################################################

from PIL import Image
import cv2
import matplotlib.pyplot as plt
import matplotlib.pyplot as plt
import skimage
from skimage import data
from skimage.filters import threshold_otsu
import cv2
import os
import numpy as np
import skimage.transform as trans
import tensorflow
from tensorflow.keras.models import *
from tensorflow.keras.layers import *
from tensorflow.keras.optimizers import *
from tensorflow.keras.callbacks import ModelCheckpoint, LearningRateScheduler
from tensorflow.keras import backend as keras
from tensorflow.keras.preprocessing.image import ImageDataGenerator
import sklearn.feature_extraction
from sklearn.datasets import load_sample_image
from sklearn.feature_extraction import image
from datetime import datetime
from keras.utils import to_categorical
from tensorflow.python.keras.optimizers import *
import skimage.io
import h5py # this was missing

#%%

# Read in the reflectance data for training
Training_data_holder =
skimage.io.imread('C:/Users/John/20211118_Python_biostudies/Python/2D_UNET_PYTHO
N/2D_PeyersPatch/1_ImageData/TRAIN_IMAGE.tif')
Training_data = Training_data_holder[0,0,:,:,2]

#Read in the mask to isolated just the lymphoid tissue
mask = Training_data_holder[0,0,:,:,3]
thresh = threshold_otsu(Training_data)
mask = mask > thresh
mask = mask*1
mask = np.uint16(mask)

# Apply the mask to the training data
```

```
Training_data = np.multiply(Training_data,mask);
Training_data = np.double(Training_data);

# Rescale the training data in the interval [0 1]
Training_data_norm_holder = np.zeros((8551, 5701),np.double)
Training_data_norm = cv2.normalize(Training_data, Training_data_norm_holder ,
1.0, 0.0, cv2.NORM_MINMAX)

#Read in the pixel-class labels created from the nuclei and actin staining
Training_labels_holder =
skimage.io.imread('C:/Users/John/20211118_Python_biostudies/Python/2D_UNET_PYTHO
N/2D_PeyersPatch/1_ImageData/TRAIN_LABELS.png') ;
Training_labels = np.where(Training_labels_holder == 3, 0,
Training_labels_holder)


#% Visually inspect the data and labels
fig1, (ax1, ax2) = plt.subplots(1, 2) # figure1
ax1.imshow(Training_data_norm)
ax1.set_title('Reflectance data')
ax2.imshow(Training_labels)
ax2.set_title('Pixel classification labels')


#%% Once happy with labels and data, commit the reflectance information data to
sub-directory in .npy format

MYDIR =
'C:/Users/John/20211118_Python_biostudies/Python/2D_UNET_PYTHON/2D_PeyersPatch/2
_TRAIN_Unet/npy_training_data/'
CHECK_FOLDER = os.path.isdir(MYDIR)

# If folder doesn't exist, then create it.
if not CHECK_FOLDER:
    os.makedirs(MYDIR)

np.save(os.path.join(MYDIR, 'Training_data'), Training_data_norm)

# Create matrices to store training data
Patching_data = np.zeros((8551, 5701,2))
Patching_data[:,:,0] =  Training_data_norm
Patching_data[:,:,1] = Training_labels

#Create a training data comprising matching patches (256x256 pixels) of image-
data and training labels
Patched_images =
sklearn.feature_extraction.image.extract_patches_2d(Patching_data, patch_size =
[256, 256], max_patches=12000, random_state=None)

#%%
################################################################################
#######################################################
# CREATE THE UNET
# input layer 256x256x1
# encoder depth 4
# 64 filters at the level of the first encoder
################################################################################
#######################################################

def unet(pretrained_weights = None,input_size=(256,256,1), n_class=3):
```

```
    inputs = tensorflow.keras.Input(shape=input_size)
    conv1 = Conv2D(64, 3, activation = 'relu', dilation_rate=2,padding = 'same',
kernel_initializer = 'he_normal')(inputs)
    conv1 = BatchNormalization()(conv1)
    conv1 = Conv2D(64, 3, activation = 'relu', dilation_rate=2,padding = 'same',
kernel_initializer = 'he_normal')(conv1)
    conv1 = BatchNormalization()(conv1)
    pool1 = MaxPooling2D(pool_size=(2, 2))(conv1)
    conv2 = Conv2D(128, 3, activation = 'relu', dilation_rate=2,padding =
'same', kernel_initializer = 'he_normal')(pool1)
    conv2 = BatchNormalization()(conv2)
    conv2 = Conv2D(128, 3, activation = 'relu', dilation_rate=2, padding =
'same', kernel_initializer = 'he_normal')(conv2)
    conv2 = BatchNormalization()(conv2)
    pool2 = MaxPooling2D(pool_size=(2, 2))(conv2)
    conv3 = Conv2D(256, 3, activation = 'relu', padding = 'same',
kernel_initializer = 'he_normal')(pool2)
    conv3 = BatchNormalization()(conv3)
    conv3 = Conv2D(256, 3, activation = 'relu', padding = 'same',
kernel_initializer = 'he_normal')(conv3)
    conv3 = BatchNormalization()(conv3)
    pool3 = MaxPooling2D(pool_size=(2, 2))(conv3)
    conv4 = Conv2D(512, 3, activation = 'relu', padding = 'same',
kernel_initializer = 'he_normal')(pool3)
    conv4 = BatchNormalization()(conv4)
    conv4 = Conv2D(512, 3, activation = 'relu', padding = 'same',
kernel_initializer = 'he_normal')(conv4)
    conv4 = BatchNormalization()(conv4)
    drop4 = Dropout(0.5)(conv4, training=True)
    pool4 = MaxPooling2D(pool_size=(2, 2))(drop4)

    conv5 = Conv2D(1024, 3, activation = 'relu', padding = 'same',
kernel_initializer = 'he_normal')(pool4)
    conv5 = BatchNormalization()(conv5)
    conv5 = Conv2D(1024, 3, activation = 'relu', padding = 'same',
kernel_initializer = 'he_normal')(conv5)
    conv5 = BatchNormalization()(conv5)
    drop5 = Dropout(0.5)(conv5, training=True)

    up6 = Conv2D(512, 2, activation = 'relu', padding = 'same',
kernel_initializer = 'he_normal')(UpSampling2D(size = (2,2))(drop5))
    merge6 = concatenate([drop4,up6], axis = 3)
    conv6 = Conv2D(512, 3, activation = 'relu', padding = 'same',
kernel_initializer = 'he_normal')(merge6)
    conv6 = Conv2D(512, 3, activation = 'relu', padding = 'same',
kernel_initializer = 'he_normal')(conv6)


    up7 = Conv2D(256, 2, activation = 'relu', padding = 'same',
kernel_initializer = 'he_normal')(UpSampling2D(size = (2,2))(conv6))
    merge7 = concatenate([conv3,up7], axis = 3)
    conv7 = Conv2D(256, 3, activation = 'relu', padding = 'same',
kernel_initializer = 'he_normal')(merge7)
    conv7 = Conv2D(256, 3, activation = 'relu', padding = 'same',
kernel_initializer = 'he_normal')(conv7)


    up8 = Conv2D(128, 2, activation = 'relu', padding = 'same',
kernel_initializer = 'he_normal')(UpSampling2D(size = (2,2))(conv7))
```

```
    merge8 = concatenate([conv2,up8], axis = 3)
    conv8 = Conv2D(128, 3, activation = 'relu', padding = 'same',
kernel_initializer = 'he_normal')(merge8)
    conv8 = Conv2D(128, 3, activation = 'relu', padding = 'same',
kernel_initializer = 'he_normal')(conv8)


    up9 = Conv2D(64, 2, activation = 'relu', padding = 'same',
kernel_initializer = 'he_normal')(UpSampling2D(size = (2,2))(conv8))
    merge9 = concatenate([conv1,up9], axis = 3)
    conv9 = Conv2D(64, 3, activation = 'relu', padding = 'same',
kernel_initializer = 'he_normal')(merge9)
    conv9 = Conv2D(64, 3, activation = 'relu', padding = 'same',
kernel_initializer = 'he_normal')(conv9)


    conv10 = Conv2D(n_class, 1, activation = 'softmax')(conv9)

    model = tensorflow.keras.Model(inputs = inputs, outputs = conv10)

    model.compile(optimizer = Adam(lr = 0.0001),loss =
'sparse_categorical_crossentropy', metrics = ['sparse_categorical_accuracy'])


    if(pretrained_weights):
      model=keras.models.load_model(pretrained_weights)

    return model



#%% Split data into image and label data and reshape ready for training
trainX = Patched_images[:,:,:,0]
trainX = np.asarray(trainX).reshape((12000, 256, 256,1))
trainY = Patched_images[:,:,:,1]
trainY = np.asarray(trainY).reshape((12000, 256, 256, 1))



#Define model for data
model = unet(pretrained_weights = None,input_size = (256,256,1),n_class=3)


# Define augmentation options
def get_train_augmented(trainX=trainX, trainY=trainY, BATCH_SIZE=12):



    aug_X = ImageDataGenerator(rotation_range=360, zoom_range=[1,1],
width_shift_range=[0,0], height_shift_range=[0,0], horizontal_flip=True,
vertical_flip = True, shear_range = 0,fill_mode = "constant",cval=0.0)
    aug_Y = ImageDataGenerator(rotation_range=360, zoom_range=[1,1],
width_shift_range=[0,0], height_shift_range=[0,0], horizontal_flip=True,
vertical_flip = True, shear_range = 0,fill_mode = "constant",cval=0.0)

    aug_X.fit(trainX, augment=True, seed=1)
    aug_Y.fit(trainY, augment=True, seed=1)

    X_train_augmented = aug_X.flow(trainX, batch_size=BATCH_SIZE,shuffle=True,
seed=1)
```

```
    Y_train_augmented = aug_Y.flow(trainY, batch_size=BATCH_SIZE, shuffle=True,
seed=1)

    train_generator = zip(X_train_augmented, Y_train_augmented)

    for (X_train_augmented,Y_train_augmented) in train_generator:
        yield (X_train_augmented,Y_train_augmented)




#%% Define path to safe network, time stamp network
checkpoint_filepath =
'C:/Users/John/20211118_Python_biostudies/Python/2D_UNET_PYTHON/2D_PeyersPatch/3
_Saved_models/'
now = datetime.now()
dt_string = now.strftime("%d/%m/%Y%H:%M:%S")
NAME =dt_string
Name_file = 'PeyersPatchBiostudies_'
checkpoint_filepath_name = checkpoint_filepath + Name_file+dt_string


checkpoint_dir =  os.path.dirname(checkpoint_filepath_name)

#Specify that weights are to be saved
model_checkpoint_callback = tensorflow.keras.callbacks.ModelCheckpoint(
    filepath=checkpoint_dir,
    save_weights_only=True,
    monitor='val_accuracy',
    mode='max',
    save_best_only=True)

# Specify training options
BATCH_SIZE = 12
NUM_EPOCHS = 50

#Augment training data
train_generator = get_train_augmented(trainX=trainX, trainY=trainY,
BATCH_SIZE=BATCH_SIZE)

#Train and save the network
history = model.fit_generator(train_generator,
steps_per_epoch=len(trainX)/(BATCH_SIZE*2), epochs=NUM_EPOCHS,
callbacks=[model_checkpoint_callback])

#Plot progress # this plots over the view of the data
plt.figure(2)
plt.plot(history.history['sparse_categorical_accuracy'])
plt.title('Model accuracy')
plt.ylabel('accuracy')
plt.xlabel('epoch')
plt.legend(['train', 'test'], loc='upper left')
plt.savefig('Training_plot.png')
plt.show()

#Save final fully trained model as a .h5 file
model.save("C:/Users/John/20211118_Python_biostudies/Python/2D_UNET_PYTHON/2D_Pe
yersPatch/3_Saved_models/my_h5_model.h5")
checkpoint_filepath_name_h5 = checkpoint_filepath + Name_file+dt_string+'.h5'
model.save(checkpoint_filepath_name_h5)
```

```
% PYTHON SCRIPT: TEST 2D UNET
% All code, image-data and screen-cast tutorial videos available for download at
% the Biostudies database under accession number S-BSST742


# -*- coding: utf-8 -*-
"""
Created on Tue Sep 21 02:46:52 2021

@author: Paul
"""
#Import all required modules
import skimage
from skimage import data
from skimage.filters import threshold_otsu
from tensorflow.keras.preprocessing.image import ImageDataGenerator
import sklearn.feature_extraction
from sklearn.datasets import load_sample_image
from sklearn.feature_extraction import image
import matplotlib.cm as cm
import matplotlib.pyplot as plt
import matplotlib.cbook as cbook
from matplotlib.path import Path
from matplotlib.patches import PathPatch
import PIL
import numpy as np
import cv2
import tensorflow
from tensorflow.keras.models import *
from tensorflow.keras.layers import *
from tensorflow.keras.optimizers import *
from tensorflow.keras.callbacks import ModelCheckpoint, LearningRateScheduler
from tensorflow.keras import backend as keras
from tensorflow.keras.preprocessing.image import ImageDataGenerator
import sklearn.feature_extraction
from sklearn.datasets import load_sample_image
from sklearn.feature_extraction import image
from datetime import datetime
from keras.utils import to_categorical
from tensorflow.python.keras.optimizers import *
import skimage.io

#%% Load the reflectance data from the unseen test image
TEST_DATA =
skimage.io.imread('C:/Users/John/20211118_Python_biostudies/Python/2D_UNET_PYTHO
N/2D_PeyersPatch/1_ImageData/TEST_IMAGE.tif');
Test_data = TEST_DATA[0,0,:,:,2]

# Load the mask for the lymphoid tissue region
Mask = TEST_DATA[0,0,:,:,3]
thresh = threshold_otsu(Test_data)
Mask = Mask > thresh
Mask = Mask*1
Mask = np.uint16(Mask)


# Rescale the test data [0 1]
# Mask the rescaled data
Test_data = np.multiply(Test_data,Mask);
Test_data = np.double(Test_data);
```

```
Test_data_norm_holder = np.zeros((11247, 7610),np.double)
Test_data_rescaled = cv2.normalize(Test_data, Test_data_norm_holder , 1.0, 0.0,
cv2.NORM_MINMAX)

#%%
################################################################################
####################################################
# Define model for testing
################################################################################
#####################################################
def unet(pretrained_weights = None,input_size=(256,256,1), n_class=3):

    inputs = tensorflow.keras.Input(shape=input_size)
    conv1 = Conv2D(64, 3, activation = 'relu', dilation_rate=2,padding = 'same',
kernel_initializer = 'he_normal')(inputs)
    conv1 = BatchNormalization()(conv1)
    conv1 = Conv2D(64, 3, activation = 'relu', dilation_rate=2,padding = 'same',
kernel_initializer = 'he_normal')(conv1)
    conv1 = BatchNormalization()(conv1)
    pool1 = MaxPooling2D(pool_size=(2, 2))(conv1)
    conv2 = Conv2D(128, 3, activation = 'relu', dilation_rate=2,padding =
'same', kernel_initializer = 'he_normal')(pool1)
    conv2 = BatchNormalization()(conv2)
    conv2 = Conv2D(128, 3, activation = 'relu', dilation_rate=2, padding =
'same', kernel_initializer = 'he_normal')(conv2)
    conv2 = BatchNormalization()(conv2)
    pool2 = MaxPooling2D(pool_size=(2, 2))(conv2)
    conv3 = Conv2D(256, 3, activation = 'relu', padding = 'same',
kernel_initializer = 'he_normal')(pool2)
    conv3 = BatchNormalization()(conv3)
    conv3 = Conv2D(256, 3, activation = 'relu', padding = 'same',
kernel_initializer = 'he_normal')(conv3)
    conv3 = BatchNormalization()(conv3)
    pool3 = MaxPooling2D(pool_size=(2, 2))(conv3)
    conv4 = Conv2D(512, 3, activation = 'relu', padding = 'same',
kernel_initializer = 'he_normal')(pool3)
    conv4 = BatchNormalization()(conv4)
    conv4 = Conv2D(512, 3, activation = 'relu', padding = 'same',
kernel_initializer = 'he_normal')(conv4)
    conv4 = BatchNormalization()(conv4)
    drop4 = Dropout(0.5)(conv4, training=True)
    pool4 = MaxPooling2D(pool_size=(2, 2))(drop4)

    conv5 = Conv2D(1024, 3, activation = 'relu', padding = 'same',
kernel_initializer = 'he_normal')(pool4)
    conv5 = BatchNormalization()(conv5)
    conv5 = Conv2D(1024, 3, activation = 'relu', padding = 'same',
kernel_initializer = 'he_normal')(conv5)
    conv5 = BatchNormalization()(conv5)
    drop5 = Dropout(0.5)(conv5, training=True)

    up6 = Conv2D(512, 2, activation = 'relu', padding = 'same',
kernel_initializer = 'he_normal')(UpSampling2D(size = (2,2))(drop5))
    merge6 = concatenate([drop4,up6], axis = 3)
    conv6 = Conv2D(512, 3, activation = 'relu', padding = 'same',
kernel_initializer = 'he_normal')(merge6)
    conv6 = Conv2D(512, 3, activation = 'relu', padding = 'same',
kernel_initializer = 'he_normal')(conv6)
```

```
    up7 = Conv2D(256, 2, activation = 'relu', padding = 'same',
kernel_initializer = 'he_normal')(UpSampling2D(size = (2,2))(conv6))
    merge7 = concatenate([conv3,up7], axis = 3)
    conv7 = Conv2D(256, 3, activation = 'relu', padding = 'same',
kernel_initializer = 'he_normal')(merge7)
    conv7 = Conv2D(256, 3, activation = 'relu', padding = 'same',
kernel_initializer = 'he_normal')(conv7)


    up8 = Conv2D(128, 2, activation = 'relu', padding = 'same',
kernel_initializer = 'he_normal')(UpSampling2D(size = (2,2))(conv7))
    merge8 = concatenate([conv2,up8], axis = 3)
    conv8 = Conv2D(128, 3, activation = 'relu', padding = 'same',
kernel_initializer = 'he_normal')(merge8)
    conv8 = Conv2D(128, 3, activation = 'relu', padding = 'same',
kernel_initializer = 'he_normal')(conv8)


    up9 = Conv2D(64, 2, activation = 'relu', padding = 'same',
kernel_initializer = 'he_normal')(UpSampling2D(size = (2,2))(conv8))
    merge9 = concatenate([conv1,up9], axis = 3)
    conv9 = Conv2D(64, 3, activation = 'relu', padding = 'same',
kernel_initializer = 'he_normal')(merge9)
    conv9 = Conv2D(64, 3, activation = 'relu', padding = 'same',
kernel_initializer = 'he_normal')(conv9)


    conv10 = Conv2D(n_class, 1, activation = 'softmax')(conv9)


    model = tensorflow.keras.Model(inputs = inputs, outputs = conv10)


    model.compile(optimizer = Adam(lr = 0.0001),loss =
'sparse_categorical_crossentropy', metrics = ['sparse_categorical_accuracy'])



    if(pretrained_weights):
      model=keras.models.load_model(pretrained_weights)

    return model


#%%
################################################################################
####################################################
# Patch the reflectance data through the network
################################################################################
#####################################################
# Set the patch sizes to be passed to the net
patchSize = [256,256]
Test_data_rescaled =
np.asarray(Test_data_rescaled).reshape((Test_data_rescaled.shape[0],
Test_data_rescaled.shape[1],1))

# Segment blockwise then reassemble in full
# Define image dimensions
[height,width,nChannel] = np.shape(Test_data_rescaled)
```

```
patch = np.zeros([patchSize[0], patchSize[1],
nChannel],dtype=Test_data_rescaled.dtype);

# Pad image to have dimensions as multiples of patchSize
padSize = np.empty([1, 2])
padSize[0,0] = patchSize[0] - np.remainder(height, patchSize[0]);
padSize[0,1] = patchSize[1] - np.remainder(width, patchSize[1]);
Starting_shape = np.shape(Test_data_rescaled)
a1 = Starting_shape[0]+padSize[0,0]
a2 = Starting_shape[1]+padSize[0,1]

im_pad = np.zeros([int(a1),int(a2)])
im_pad[:Test_data_rescaled.shape[0],:Test_data_rescaled.shape[1]] =
Test_data_rescaled[:,:,0]
im_pad = np.asarray(im_pad).reshape((im_pad.shape[0], im_pad.shape[1],1))
[height_pad, width_pad, nChannel_pad] = np.shape(im_pad);

# Preallocate some matrices to receive the network outputs
out_Uncertainty_Scores = np.zeros([np.shape(im_pad)[0], np.shape(im_pad)[1]],
'double');

out_Pmap_cat1 = np.zeros([np.shape(im_pad)[0], np.shape(im_pad)[1]], 'double');
out_Pmap_cat2 = np.zeros([np.shape(im_pad)[0], np.shape(im_pad)[1]], 'double');
out_Pmap_cat3 = np.zeros([np.shape(im_pad)[0], np.shape(im_pad)[1]], 'double');

# Load a pretrained network
pretrained_model = unet()
pretrained_model.load_weights('C:/Users/John/20211118_Python_biostudies/Python/2
D_UNET_PYTHON/2D_PeyersPatch/3_Saved_models/my_h5_model_fully_trained.h5')

# Loop through blocks of 'patchSize'
for loop in range(1, int(height_pad),int(patchSize[0])):
    print(loop)
    for j in range(1,int(width_pad),int(patchSize[1])):

        for p in range(1,(nChannel+1)):
            PP3 = np.empty([1,256,256,1])

            im_pad_touse =im_pad[(loop-1):((loop-1)+patchSize[0]),(j-1):((j-
1)+(patchSize[1]))]
            patch[:,:,0] = np.squeeze(im_pad_touse,axis=None)

            PP3[0,:,:,:]=patch
            # deploy net
            predictions = pretrained_model.predict(PP3)

            scores = np.max(predictions,axis=3)
            predictions.argmax(axis=3)

            out_Uncertainty_Scores[(loop-1):(loop+patchSize[0]-1), (j-
1):(j+patchSize[1]-1)] = scores;
            out_Pmap_cat1[(loop-1):(loop+patchSize[0]-1), (j-1):(j+patchSize[1]-
1)] = predictions[0,:,:,0];
            out_Pmap_cat2[(loop-1):(loop+patchSize[0]-1), (j-1):(j+patchSize[1]-
1)] = predictions[0,:,:,1];
            out_Pmap_cat3[(loop-1):(loop+patchSize[0]-1), (j-1):(j+patchSize[1]-
1)] = predictions[0,:,:,2];


# Remove padding from the network outputs
```

```
out_Uncertainty_Scores = out_Uncertainty_Scores[0:height, 0:width];
out_Pmap_cat1 = out_Pmap_cat1[0:height, 0:width];
out_Pmap_cat2 = out_Pmap_cat2[0:height, 0:width];
out_Pmap_cat3 = out_Pmap_cat3[0:height, 0:width];

image_to_plot = np.zeros((11247, 7610,3))
image_to_plot[:,:,0] = out_Pmap_cat1
image_to_plot[:,:,1] = out_Pmap_cat2
image_to_plot[:,:,2] = out_Pmap_cat3

#%% visualise network outputs
fig, (ax1, ax2, ax3, ax4) = plt.subplots(1, 4, sharey=True)
fig.set_size_inches(14, 12)

im = ax1.imshow(out_Pmap_cat1)
ax1.title.set_text('Background/Other')

im = ax2.imshow(out_Pmap_cat2)
ax2.title.set_text('LF-Actin')

im = ax3.imshow(out_Pmap_cat3)
ax3.title.set_text('LF-Nuclei')

im = ax4.imshow(out_Uncertainty_Scores)
ax4.title.set_text('Uncertainty')

plt.savefig('Predictions.png')

#%% Map to 16-bit and save for loading into CellProfiler
# Background/other
data_norm_holder = np.zeros((11247, 7610),np.uint16)
ui16_PMAP_cat1_xy = cv2.normalize(out_Pmap_cat1, data_norm_holder , 65535.0,
0.0, cv2.NORM_MINMAX)
img_data2 = (ui16_PMAP_cat1_xy).astype(dtype=np.uint16)
rawtiff=PIL.Image.fromarray(img_data2)
rawtiff.save('Ch_7_Im_001'+'.tiff')

# LF-Actin
data_norm_holder = np.zeros((11247, 7610),np.uint16)
ui16_PMAP_cat2_xy = cv2.normalize(out_Pmap_cat2, data_norm_holder , 65535.0,
0.0, cv2.NORM_MINMAX)
img_data2 = (ui16_PMAP_cat2_xy).astype(dtype=np.uint16)
rawtiff=PIL.Image.fromarray(img_data2)
rawtiff.save('Ch_6_Im_001'+'.tiff')

# LF-Nuclei
data_norm_holder = np.zeros((11247, 7610),np.uint16)
ui16_PMAP_cat3_xy = cv2.normalize(out_Pmap_cat3, data_norm_holder , 65535.0,
0.0, cv2.NORM_MINMAX)
img_data2 = (ui16_PMAP_cat3_xy).astype(dtype=np.uint16)
rawtiff=PIL.Image.fromarray(img_data2)
rawtiff.save('Ch_5_Im_001'+'.tiff')
```

```
% PYTHON SCRIPT: TRAIN 3D UNET
% All code, image-data and screen-cast tutorial videos available for download at
% the Biostudies database under accession number S-BSST742


# -*- coding: utf-8 -*-
"""
Created on Mon Jun 14 15:21:44 2021

@author: Paul
"""

from PIL import Image
import javabridge
import bioformats
javabridge.start_vm(class_path=bioformats.JARS)
import cv2
import matplotlib.pyplot as plt
import matplotlib.pyplot as plt
import skimage
from skimage import data
from skimage.filters import threshold_otsu
import os
import numpy as np
import skimage.transform as trans
import numpy as np
import tensorflow
from tensorflow.keras.models import *
from tensorflow.keras.layers import *
from tensorflow.keras.optimizers import *
from tensorflow.keras.callbacks import ModelCheckpoint, LearningRateScheduler
from tensorflow.keras import backend as keras
from tensorflow.keras.preprocessing.image import ImageDataGenerator
import sklearn.feature_extraction
from sklearn.datasets import load_sample_image
from sklearn.feature_extraction import image
from datetime import datetime
from keras.utils import to_categorical
from tensorflow.python.keras.optimizers import *
import random
import matplotlib.pyplot as plt
from mpl_toolkits.mplot3d.art3d import Poly3DCollection
import numpy as np

from skimage import exposure, io, util


#%% Specify directory containing training data
Training_data_directory =
'C:/Users/John/20211118_Python_biostudies/Python/3D_UNET_PYTHON/3D_UNET_PYTHON/1
_ImageData/TRAIN/DATA/'

# Specify channel number in training data that contains reflectance information
RL_training_directory = 3;

# Specify directory containing training labels
Training_labels_path =
'C:/Users/John/20211118_Python_biostudies/Python/3D_UNET_PYTHON/3D_UNET_PYTHON/1
_ImageData/TRAIN/LABELS/' ;
```

```
# COMMIT TRAINING DATA TO DIRECTORY rescaled [0 1] IN MAT FORMAT
MYDIR
='C:/Users/John/20211118_Python_biostudies/Python/3D_UNET_PYTHON/3D_UNET_PYTHON/
1_ImageData/TRAIN/LABELS/mat_training_data/'
CHECK_FOLDER = os.path.isdir(MYDIR)

# If folder doesn't exist, then create it.
if not CHECK_FOLDER:
    os.makedirs(MYDIR)


Training_labels_holder =
skimage.io.imread('C:/Users/John/20211118_Python_biostudies/Python/3D_UNET_PYTHO
N/3D_UNET_PYTHON/1_ImageData/TRAIN/LABELS/LABELS_001.tif') ;
Training_labels = np.where(Training_labels_holder == 3, 0,
Training_labels_holder)


#%%
for i in range(0,2):

    loop = i+1

    counter = '00'+str(loop)

    # find metadata describing file
    TOTAL_path = Training_data_directory+'TRAIN_'+counter+'.tif'

    reader = bioformats.ImageReader(TOTAL_path)
    NUMBERIMAGES = reader.rdr.getSeriesCount(TOTAL_path)
    number_of_channels = reader.rdr.getSizeC(TOTAL_path)
    Xlength = reader.rdr.getSizeX(TOTAL_path)
    Ylength = reader.rdr.getSizeY(TOTAL_path)

    stackSizeZ  = reader.rdr.getSizeZ(TOTAL_path)# number of Z slices
    channel_zimage = np.zeros((Xlength,Ylength,stackSizeZ))


    # Load reflectance information
    for zplane in range(1,stackSizeZ+1):

        channel_zimage[:,:,(zplane-1)] = bioformats.load_image(TOTAL_path,z =
(zplane-1), c= (RL_training_directory-1))
    IM_DATA = channel_zimage


    IM_DATA = np.double(IM_DATA);
    DIM = IM_DATA.shape
    IM_DATA_norm_holder = np.zeros((DIM[0],DIM[1],DIM[2]),np.double)
    IM_DATA_norm = cv2.normalize(IM_DATA, IM_DATA_norm_holder, 1.0, 0.0,
cv2.NORM_MINMAX)
    pw = os.getcwd()
    FILENAME = pw+'\\npy_training_data\\'+'TRAIN_DATA_'+counter
    FOLDER_CHECK = pw+'\\npy_training_data\\'
    CHECK_FOLDER = os.path.isdir(FOLDER_CHECK)
    if not CHECK_FOLDER:
        os.makedirs(FOLDER_CHECK)

    np.save(FILENAME,IM_DATA_norm)
```

```
pw = os.getcwd()

#%% COMMIT TRAINING LABELS TO DIRECTORY IN MAT FORMAT
FOLDER_NAME = pw+'\\npy_training_labels\\'
CHECK_FOLDER2 = os.path.isdir(FOLDER_NAME)
if not CHECK_FOLDER2:
    os.makedirs(FOLDER_NAME)

for i in range(0,2):
    loop = i +1
    counter = '00'+str(loop)
    label_zimage = [];
    label_zimage = np.zeros((Xlength,Ylength,stackSizeZ))
    # load label information
    for zplane in range(1,stackSizeZ+1):

        TOTAL_path = Training_labels_path+'LABELS_'+counter+'.tif'
        iplane = bioformats.load_image(TOTAL_path,z=(zplane-1), c=0,rescale =
False )

        label_zimage[:,:,(zplane-1)] = iplane

    IM_LABELS = np.double(label_zimage);
    DIM = IM_LABELS.shape
    IM_LABELS_norm_holder = np.zeros((DIM[0],DIM[1],DIM[2]),np.double)
    IM_LABELS_norm_holder[:,:,:] = label_zimage[:,:,:]
    pw = os.getcwd()
    FILENAME = pw+'\\npy_training_labels\\'+'TRAIN_LABELS_'+counter
    FOLDER_CHECK = pw+'\\npy_training_labels\\'
    CHECK_FOLDER = os.path.isdir(FOLDER_CHECK)
    if not CHECK_FOLDER:
        os.makedirs(FOLDER_CHECK)

    np.save(FILENAME, IM_LABELS_norm_holder)

#%%
NUMBER_batch = 1

del  channel_zimage,  IM_LABELS, IM_DATA,   label_zimage

# Prepare to extract patches
patchPerImage = 375;
xrand = random.sample(range(0,959), patchPerImage)
yrand = random.sample(range(0,959), patchPerImage)
zrand = np.random.choice(78,patchPerImage, replace=True)


# Define random patch extraction and augmentarion of patches
def get_train_patched(xrand, yrand, zrand,BATCH_SIZE,batch_number =
NUMBER_batch, trainXm=IM_DATA_norm, trainYm=IM_LABELS_norm_holder):

    patched_data_image = np.zeros((BATCH_SIZE,64,64,32))
    patched_data_labels = np.zeros((BATCH_SIZE,64,64,32))
    for i in range(0,BATCH_SIZE):
        val = (i + batch_number)-1;
        patched_data_image[i,:,:,:] =
trainXm[(xrand[val]):(xrand[val]+64),(yrand[val]):(yrand[val]+64),(zrand[val]):(
zrand[val]+32)];
```

```
        patched_data_labels[i,:,:,:] =
trainYm[(xrand[val]):(xrand[val]+64),(yrand[val]):(yrand[val]+64),(zrand[val]):(
zrand[val]+32)];
    return patched_data_image, patched_data_labels


def get_train_augmented(trainXm,trainYm ,BATCH_SIZE,batch_number =
NUMBER_batch):
    for i in range(0,BATCH_SIZE):
        rand_number = random.sample(range(BATCH_SIZE), 1)
        my_arr = [np.rot90, np.flipud, np.fliplr]
        if int(rand_number[0])<3:
            trainXm[i,:,:,:] = my_arr[int(rand_number[0])](trainXm[i,:,:,:])
            trainYm[i,:,:,:] = my_arr[int(rand_number[0])](trainYm[i,:,:,:])


        elif int(rand_number[0]) ==3:

            trainXm[i,:,:,:] = np.fliplr(trainXm[i,:,:,:])
            trainYm[i,:,:,:] = np.fliplr(trainYm[i,:,:,:])
            trainXm[i,:,:,:] = np.rot90(trainXm[i,:,:,:])
            trainYm[i,:,:,:] = np.rot90(trainYm[i,:,:,:])

        else:
            trainXm[i,:,:,:] = trainXm[i,:,:,:]
            trainYm[i,:,:,:] = trainYm[i,:,:,:]
        return trainXm, trainYm

#%%
################################################################################
####################################################
# CREATE THE 3D UNET
# input layer 64x64x32x1
# encoder depth 4
# 32 filters at the level of the first encoder
################################################################################
#####################################################

def unet3(pretrained_weights = None,input_size= (64,64,32,1), n_class=4):

    inputs = tensorflow.keras.Input(shape=input_size)
    conv1 = Conv3D(32, kernel_size=(3, 3, 3),dilation_rate=2,padding = 'same',
kernel_initializer = 'he_normal')(inputs)
    conv1 = BatchNormalization()(conv1)
    conv1 = Activation('relu')(conv1)
    conv1 = Conv3D(64, kernel_size=(3, 3, 3), dilation_rate=2,padding = 'same',
kernel_initializer = 'he_normal')(conv1)
    conv1 = BatchNormalization()(conv1)
    up1 = Activation('relu')(conv1)
    pool1 = MaxPooling3D(pool_size=(2,2,2),strides = (2,2,2))(up1)
    conv2 = Conv3D(64, kernel_size=(3, 3, 3), dilation_rate=2,padding = 'same',
kernel_initializer = 'he_normal')(pool1)
    conv2 = BatchNormalization()(conv2)
    conv2 = Activation('relu')(conv2)
    conv2 = Conv3D(128, kernel_size=(3, 3, 3), dilation_rate=2,padding = 'same',
kernel_initializer = 'he_normal')(up1)
    conv2 = BatchNormalization()(conv2)
    up2 = Activation('relu')(conv2)
    pool2 = MaxPooling3D(pool_size=(2,2,2),strides = (2,2,2))(up2)
```

```
    conv3 = Conv3D(128, kernel_size=(3, 3, 3), dilation_rate=2,padding = 'same', 
kernel_initializer = 'he_normal')(pool2)
    conv3 = BatchNormalization()(conv3)
    conv3 = Activation('relu')(conv3)
    conv3 = Conv3D(256, kernel_size=(3, 3, 3), dilation_rate=2,padding = 'same', 
kernel_initializer = 'he_normal')(conv3)
    conv3 = BatchNormalization()(conv3)
    up3 = Activation('relu')(conv3)
    pool3 = MaxPooling3D(pool_size=(2,2,2),strides = (2,2,2))(up3)
    conv4 = Conv3D(256, kernel_size=(3, 3, 3),  dilation_rate=2,padding = 
'same', kernel_initializer = 'he_normal')(pool3)
    conv4 = BatchNormalization()(conv4)
    conv4 = Activation('relu')(conv4)
    conv4 = Conv3D(512, kernel_size=(3, 3, 3),  dilation_rate=2,padding = 
'same', kernel_initializer = 'he_normal')(conv4)
    conv4 = BatchNormalization()(conv4)
    up4 = Activation('relu')(conv4)
    pool4 = MaxPooling3D(pool_size=(2,2,2),strides = (2,2,2))(up4)
    conv5 = Conv3D(512, kernel_size=(3, 3, 3),  dilation_rate=2,padding = 
'same', kernel_initializer = 'he_normal')(pool4)
    conv5 = BatchNormalization()(conv5)
    conv5 = Activation('relu')(conv5)
    conv5 = Conv3D(1024, kernel_size=(3, 3, 3),  dilation_rate=2,padding = 
'same', kernel_initializer = 'he_normal')(conv5)
    conv5 = BatchNormalization()(conv5)
    conv5 = Activation('relu')(conv5)
    drop5 = Conv3DTranspose(1024, kernel_size=(2, 2, 2),strides=(2, 2, 2), 
activation = 'relu', dilation_rate=2,padding = 'same', kernel_initializer = 
'he_normal')(conv5)

    merge6 = concatenate([drop5,up4], axis = 4)
    conv6 = Conv3D(512, kernel_size=(3, 3, 3),  dilation_rate=2,padding = 
'same', kernel_initializer = 'he_normal')(merge6)
    conv6 = BatchNormalization()(conv6)
    conv6 = Activation('relu')(conv6)
    conv6 = Conv3D(512, kernel_size=(3, 3, 3),  dilation_rate=2,padding = 
'same', kernel_initializer = 'he_normal')(conv6)
    conv6 = BatchNormalization()(conv6)
    conv6 = Activation('relu')(conv6)
    drop6 = Conv3DTranspose(512, kernel_size=(2, 2, 2),strides=(2, 2, 2), 
activation = 'relu', dilation_rate=2,padding = 'same', kernel_initializer = 
'he_normal')(conv6)
    merge7 = concatenate([drop6,up3], axis = 4)

    conv7 = Conv3D(256, kernel_size=(3, 3, 3), dilation_rate=2,padding = 'same', 
kernel_initializer = 'he_normal')(merge7)
    conv7 = BatchNormalization()(conv7)
    conv7 = Activation('relu')(conv7)
    conv7 = Conv3D(256, kernel_size=(3, 3, 3), dilation_rate=2,padding = 'same', 
kernel_initializer = 'he_normal')(conv7)
    conv7 = BatchNormalization()(conv7)
    conv7 = Activation('relu')(conv7)
    drop7 = Conv3DTranspose(256, kernel_size=(2, 2, 2),strides=(2, 2, 2), 
activation = 'relu', dilation_rate=2,padding = 'same', kernel_initializer = 
'he_normal')(conv7)
    merge8 = concatenate([drop7,up2], axis = 4)


    conv8 = Conv3D(128, kernel_size=(3, 3, 3),  dilation_rate=2,padding = 
'same', kernel_initializer = 'he_normal')(merge8)
```

```
    conv8 = BatchNormalization()(conv8)
    conv8 = Activation('relu')(conv8)
    conv8 = Conv3D(128, kernel_size=(3, 3, 3),  dilation_rate=2,padding =
'same', kernel_initializer = 'he_normal')(conv8)
    conv8 = BatchNormalization()(conv8)
    conv8 = Activation('relu')(conv8)
    drop8 = Conv3DTranspose(128, kernel_size=(2, 2, 2),strides=(2, 2, 2),
activation = 'relu', dilation_rate=2,padding = 'same', kernel_initializer =
'he_normal')(conv8)
    conv8 = BatchNormalization()(drop8)
    conv8 = Activation('relu')(conv8)
    drop8 = Conv3DTranspose(128, kernel_size=(2, 2, 2),strides=(2, 2, 2),
activation = 'relu', dilation_rate=2,padding = 'same', kernel_initializer =
'he_normal')(conv7)


    merge9 = concatenate([drop8,up1], axis = 4)
    conv9 = Conv3D(64, kernel_size=(3, 3, 3),  dilation_rate=2,padding = 'same',
kernel_initializer = 'he_normal')(merge9)
    conv9 = BatchNormalization()(conv9)
    conv9 = Activation('relu')(conv9)
    conv9 = Conv3D(64, kernel_size=(3, 3, 3),  dilation_rate=2,padding = 'same',
kernel_initializer = 'he_normal')(conv9)
    conv9 = BatchNormalization()(conv9)
    conv9 = Activation('relu')(conv9)

    conv9 = Conv3D(n_class, kernel_size=(1, 1, 1),activation = 'softmax')(conv9)


    model = tensorflow.keras.Model(inputs = inputs, outputs = conv9)
    model.compile(optimizer = Adam(lr = 0.0005),loss =
'sparse_categorical_crossentropy', metrics = ['sparse_categorical_accuracy'])

    if(pretrained_weights):
      model=keras.models.load_model(pretrained_weights)

    return model


#%% Specify location to save the network
checkpoint_filepath =
'C:/Users/John/20211118_Python_biostudies/Python/3D_UNET_PYTHON/3D_UNET_PYTHON/3
_Saved_models/'
now = datetime.now()
dt_string = now.strftime("%d/%m/%Y%H:%M:%S")
NAME =dt_string
Name_file = 'PeyersPatchBiostudies_'
checkpoint_filepath_name = checkpoint_filepath + Name_file+dt_string

checkpoint_dir =  os.path.dirname(checkpoint_filepath_name)


model_checkpoint_callback = tensorflow.keras.callbacks.ModelCheckpoint(
    filepath=checkpoint_dir,
    save_weights_only=True,
    monitor='val_accuracy',
    mode='max',
    save_best_only=True)

#Specify training options
```

```
BATCH_SIZE = 8
NUM_EPOCHS = 50
patchPerImage = 375;
steps = np.floor(patchPerImage/8);

NUMBER_batch = 1;



start = 0;
finish = 8;
storeX = np.zeros((375,64,64,32,1))
storeY = np.zeros((375,64,64,32,1))

#Extract patches
for i in range(1,int(steps)):


    [patched_data_image, patched_data_labels]=get_train_patched(xrand, yrand,
zrand, BATCH_SIZE, batch_number = NUMBER_batch, trainXm=IM_DATA_norm,
trainYm=IM_LABELS_norm_holder)



    [trainXmgg, trainYmgg] =
get_train_augmented(BATCH_SIZE=8,trainXm=patched_data_image,
trainYm=patched_data_labels, batch_number = NUMBER_batch)
    trainYmgg = trainYmgg.reshape((8,64,64,32,1))
    trainXmgg = trainXmgg.reshape((8,64,64,32,1))

    trainXmgg = trainXmgg.astype('float32')
    trainYmgg = trainYmgg.astype('float32')

    storeX[start:finish,:,:,:,:] = trainXmgg
    storeY[start:finish,:,:,:,:] = trainYmgg

    start = finish
    finish = start+8
    NUMBER_batch = NUMBER_batch+1


[patched_data_image, patched_data_labels]=get_train_patched(xrand, yrand, zrand,
BATCH_SIZE=(patchPerImage-finish), batch_number = NUMBER_batch,
trainXm=IM_DATA_norm, trainYm=IM_LABELS_norm_holder)


[trainXmgg, trainYmgg] = get_train_augmented(BATCH_SIZE=(patchPerImage-
finish),trainXm=patched_data_image, trainYm=patched_data_labels, batch_number =
NUMBER_batch)
trainYmgg = trainYmgg.reshape((patchPerImage-finish,64,64,32,1))
trainXmgg = trainXmgg.reshape((patchPerImage-finish,64,64,32,1))

trainXmgg = trainXmgg.astype('float32')
trainYmgg = trainYmgg.astype('float32')

storeX[finish:patchPerImage,:,:,:,:] = trainXmgg
storeY[finish:patchPerImage,:,:,:,:] = trainYmgg

model = unet3(pretrained_weights = None,input_size =
(64,64,32,1),n_class=4)#change to 3
```

```
#%% Train the model
history = model.fit(
    storeX,
    storeY,
    batch_size = BATCH_SIZE,
    epochs = NUM_EPOCHS,callbacks=[model_checkpoint_callback])

plt.plot(history.history['sparse_categorical_accuracy'])
plt.title('Model accuracy')
plt.ylabel('accuracy')
plt.xlabel('epoch')
plt.legend(['train', 'test'], loc='upper left')
plt.savefig('Training_plot.png')
plt.show()

model.save("C:/Users/John/20211118_Python_biostudies/Python/3D_UNET_PYTHON/3D_UN
ET_PYTHON/3_Saved_models/my_h53D_model.h5")
```

```
% PYTHON SCRIPT: TEST 3D UNET
% All code, image-data and screen-cast tutorial videos available for download at
% the Biostudies database under accession number S-BSST742


# -*- coding: utf-8 -*-
"""
Created on Mon Sep 20 16:37:00 2021

@author: Paul
"""
from PIL import Image
import javabridge
import bioformats
javabridge.start_vm(class_path=bioformats.JARS)
import cv2
import matplotlib.pyplot as plt
import matplotlib.pyplot as plt
import skimage
from skimage import data
from skimage.filters import threshold_otsu
import os
import numpy as np
import skimage.transform as trans
import numpy as np
import tensorflow
from tensorflow.keras.models import *
from tensorflow.keras.layers import *
from tensorflow.keras.optimizers import *
from tensorflow.keras.callbacks import ModelCheckpoint, LearningRateScheduler
from tensorflow.keras import backend as keras
from tensorflow.keras.preprocessing.image import ImageDataGenerator
import sklearn.feature_extraction
from sklearn.datasets import load_sample_image
from sklearn.feature_extraction import image
from datetime import datetime
from keras.utils import to_categorical
from tensorflow.python.keras.optimizers import *
import random
import matplotlib.pyplot as plt
from mpl_toolkits.mplot3d.art3d import Poly3DCollection
import numpy as np

from skimage import exposure, io, util
from skimage import data
from tensorflow.keras.preprocessing.image import ImageDataGenerator
import sklearn.feature_extraction
from sklearn.datasets import load_sample_image
from sklearn.feature_extraction import image
import matplotlib.cm as cm

import matplotlib.cbook as cbook
from matplotlib.path import Path
from matplotlib.patches import PathPatch
import math
import json
import numpy as np
import PIL

import imageio
```

```
#%% Specify directory containing test data
Test_data_directory =
'C:/Users/John/20211118_Python_biostudies/Python/3D_UNET_PYTHON/3D_UNET_PYTHON/1
_ImageData/TEST/'

# Specify channel number in test data that contains reflectance information
RL_channel_number = 2;

#COMMIT REFLECTANCE TEST DATA TO DIRECTORY RESCALED [0 1] IN MAT FORMAT
MYDIR
='C:/Users/John/20211118_Python_biostudies/Python/3D_UNET_PYTHON/3D_UNET_PYTHON/
4_TEST_Unet/npy_test_data/'
CHECK_FOLDER = os.path.isdir(MYDIR)


# If folder doesn't exist, then create it.
if not CHECK_FOLDER:
    os.makedirs(MYDIR)


for i in range(0,1):

    loop = i+1

    counter = '00'+str(loop)

    TOTAL_path = Test_data_directory+'TEST_'+counter+'.tif'
    # find metadata describing file
    reader = bioformats.ImageReader(TOTAL_path)

    NUMBERIMAGES = reader.rdr.getSeriesCount(TOTAL_path)
    number_of_channels = reader.rdr.getSizeC(TOTAL_path)
    Xlength = reader.rdr.getSizeX(TOTAL_path)
    Ylength = reader.rdr.getSizeY(TOTAL_path)
    stackSizeZ  = reader.rdr.getSizeZ(TOTAL_path)

    channel_zimage = np.zeros((Xlength,Ylength,stackSizeZ))
    # Load reflectance information
    for zplane in range(1,stackSizeZ+1):

        channel_zimage[:,:,(zplane-1)] = bioformats.load_image(TOTAL_path,z =
(zplane-1), c= (RL_channel_number-1))
    IM_DATA = channel_zimage


    IM_DATA = np.double(IM_DATA);
    DIM = IM_DATA.shape
    IM_DATA_norm_holder = np.zeros((DIM[0],DIM[1],DIM[2]),np.double)
    IM_DATA_norm = cv2.normalize(IM_DATA, IM_DATA_norm_holder, 1.0, 0.0,
cv2.NORM_MINMAX)
    pw = os.getcwd()
    FILENAME = pw+'\\npy_test_data\\'+'TEST_DATA_'+counter
    FOLDER_CHECK = pw+'\\npy_test_data\\'
    CHECK_FOLDER = os.path.isdir(FOLDER_CHECK)
    if not CHECK_FOLDER:
        os.makedirs(FOLDER_CHECK)

    np.save(FILENAME,IM_DATA_norm)
```

```
#%%
#Redefine network
def unet3(pretrained_weights = None,input_size= (64,64,32,1), n_class=4):

    inputs = tensorflow.keras.Input(shape=input_size)
    conv1 = Conv3D(32, kernel_size=(3, 3, 3),dilation_rate=2,padding = 'same',
kernel_initializer = 'he_normal')(inputs)
    conv1 = BatchNormalization()(conv1)
    conv1 = Activation('relu')(conv1)
    conv1 = Conv3D(64, kernel_size=(3, 3, 3), dilation_rate=2,padding = 'same',
kernel_initializer = 'he_normal')(conv1)
    conv1 = BatchNormalization()(conv1)
    up1 = Activation('relu')(conv1)
    pool1 = MaxPooling3D(pool_size=(2,2,2),strides = (2,2,2))(up1)
    conv2 = Conv3D(64, kernel_size=(3, 3, 3), dilation_rate=2,padding = 'same',
kernel_initializer = 'he_normal')(pool1)
    conv2 = BatchNormalization()(conv2)
    conv2 = Activation('relu')(conv2)
    conv2 = Conv3D(128, kernel_size=(3, 3, 3), dilation_rate=2,padding = 'same',
kernel_initializer = 'he_normal')(up1)
    conv2 = BatchNormalization()(conv2)
    up2 = Activation('relu')(conv2)
    pool2 = MaxPooling3D(pool_size=(2,2,2),strides = (2,2,2))(up2)
    conv3 = Conv3D(128, kernel_size=(3, 3, 3), dilation_rate=2,padding = 'same',
kernel_initializer = 'he_normal')(pool2)
    conv3 = BatchNormalization()(conv3)
    conv3 = Activation('relu')(conv3)
    conv3 = Conv3D(256, kernel_size=(3, 3, 3), dilation_rate=2,padding = 'same',
kernel_initializer = 'he_normal')(conv3)
    conv3 = BatchNormalization()(conv3)
    up3 = Activation('relu')(conv3)
    pool3 = MaxPooling3D(pool_size=(2,2,2),strides = (2,2,2))(up3)
    conv4 = Conv3D(256, kernel_size=(3, 3, 3),  dilation_rate=2,padding =
'same', kernel_initializer = 'he_normal')(pool3)
    conv4 = BatchNormalization()(conv4)
    conv4 = Activation('relu')(conv4)
    conv4 = Conv3D(512, kernel_size=(3, 3, 3),  dilation_rate=2,padding =
'same', kernel_initializer = 'he_normal')(conv4)
    conv4 = BatchNormalization()(conv4)
    up4 = Activation('relu')(conv4)
    pool4 = MaxPooling3D(pool_size=(2,2,2),strides = (2,2,2))(up4)
    conv5 = Conv3D(512, kernel_size=(3, 3, 3),  dilation_rate=2,padding =
'same', kernel_initializer = 'he_normal')(pool4)
    conv5 = BatchNormalization()(conv5)
    conv5 = Activation('relu')(conv5)
    conv5 = Conv3D(1024, kernel_size=(3, 3, 3),  dilation_rate=2,padding =
'same', kernel_initializer = 'he_normal')(conv5)
    conv5 = BatchNormalization()(conv5)
    conv5 = Activation('relu')(conv5)
    drop5 = Conv3DTranspose(1024, kernel_size=(2, 2, 2),strides=(2, 2, 2),
activation = 'relu', dilation_rate=2,padding = 'same', kernel_initializer =
'he_normal')(conv5)

    merge6 = concatenate([drop5,up4], axis = 4)
    conv6 = Conv3D(512, kernel_size=(3, 3, 3),  dilation_rate=2,padding =
'same', kernel_initializer = 'he_normal')(merge6)
    conv6 = BatchNormalization()(conv6)
    conv6 = Activation('relu')(conv6)
    conv6 = Conv3D(512, kernel_size=(3, 3, 3),  dilation_rate=2,padding =
'same', kernel_initializer = 'he_normal')(conv6)
```

```
    conv6 = BatchNormalization()(conv6)
    conv6 = Activation('relu')(conv6)
    drop6 = Conv3DTranspose(512, kernel_size=(2, 2, 2),strides=(2, 2, 2),
activation = 'relu', dilation_rate=2,padding = 'same', kernel_initializer =
'he_normal')(conv6)
    merge7 = concatenate([drop6,up3], axis = 4)

    conv7 = Conv3D(256, kernel_size=(3, 3, 3), dilation_rate=2,padding = 'same',
kernel_initializer = 'he_normal')(merge7)
    conv7 = BatchNormalization()(conv7)
    conv7 = Activation('relu')(conv7)
    conv7 = Conv3D(256, kernel_size=(3, 3, 3), dilation_rate=2,padding = 'same',
kernel_initializer = 'he_normal')(conv7)
    conv7 = BatchNormalization()(conv7)
    conv7 = Activation('relu')(conv7)
    drop7 = Conv3DTranspose(256, kernel_size=(2, 2, 2),strides=(2, 2, 2),
activation = 'relu', dilation_rate=2,padding = 'same', kernel_initializer =
'he_normal')(conv7)
    merge8 = concatenate([drop7,up2], axis = 4)


    conv8 = Conv3D(128, kernel_size=(3, 3, 3),  dilation_rate=2,padding =
'same', kernel_initializer = 'he_normal')(merge8)
    conv8 = BatchNormalization()(conv8)
    conv8 = Activation('relu')(conv8)
    conv8 = Conv3D(128, kernel_size=(3, 3, 3),  dilation_rate=2,padding =
'same', kernel_initializer = 'he_normal')(conv8)
    conv8 = BatchNormalization()(conv8)
    conv8 = Activation('relu')(conv8)
    drop8 = Conv3DTranspose(128, kernel_size=(2, 2, 2),strides=(2, 2, 2),
activation = 'relu', dilation_rate=2,padding = 'same', kernel_initializer =
'he_normal')(conv8)
    conv8 = BatchNormalization()(drop8)
    conv8 = Activation('relu')(conv8)
    drop8 = Conv3DTranspose(128, kernel_size=(2, 2, 2),strides=(2, 2, 2),
activation = 'relu', dilation_rate=2,padding = 'same', kernel_initializer =
'he_normal')(conv7)


    merge9 = concatenate([drop8,up1], axis = 4)
    conv9 = Conv3D(64, kernel_size=(3, 3, 3),  dilation_rate=2,padding = 'same',
kernel_initializer = 'he_normal')(merge9)
    conv9 = BatchNormalization()(conv9)
    conv9 = Activation('relu')(conv9)
    conv9 = Conv3D(64, kernel_size=(3, 3, 3),  dilation_rate=2,padding = 'same',
kernel_initializer = 'he_normal')(conv9)
    conv9 = BatchNormalization()(conv9)
    conv9 = Activation('relu')(conv9)

    conv9 = Conv3D(n_class, kernel_size=(1, 1, 1),activation = 'softmax')(conv9)




    model = tensorflow.keras.Model(inputs = inputs, outputs = conv9)
    model.compile(optimizer = Adam(lr = 0.0005),loss =
'sparse_categorical_crossentropy', metrics = ['sparse_categorical_accuracy'])
```

```
    if(pretrained_weights):
      model=keras.models.load_model(pretrained_weights)

    return model

#%% load the trained 3D Unet network
#model.load_weights("C:/Users/John/20211118_Python_biostudies/Python/3D_UNET_PYT
HON/3D_UNET_PYTHON/3_Saved_models/my_h53D_model.h5")
pretrained_model = unet3()
pretrained_model.load_weights("C:/Users/John/20211118_Python_biostudies/Python/3
D_UNET_PYTHON/3D_UNET_PYTHON/3_Saved_models/my_h53D_model_updated.h5")


#%% Patch the reflectance data through the network
################################################################################
#################################################
# Set the patch sizes to be passed to the net

patchSize = [64, 64, 32]
# Segment blockwise then reassemble in full
# Define image dimensions
Shape_overall = np.shape(IM_DATA_norm)
height = Shape_overall[0]
width = Shape_overall[1]
depth = Shape_overall[2]

if len(Shape_overall) == 3:
    nChannel = 1
else:
    nChannel = Shape_overall[3]


patch = np.zeros([patchSize[0],patchSize[1],patchSize[2],
nChannel],dtype=IM_DATA_norm.dtype);
number_of_height_patches = math.ceil(height/patchSize[0]);
number_of_width_patches = math.ceil(width/patchSize[1]);
number_of_depth_patches = math.ceil(depth/patchSize[2]);

#Pad image to have dimensions as multiples of patchSize
height_pad = number_of_height_patches*patchSize[0];
width_pad  = number_of_width_patches*patchSize[1];
depth_pad  = number_of_depth_patches*patchSize[2];

# Amount to pad different dimensions of image
padSize = np.empty([1, 3])

# Pad the image by correct amounts
padSize[0,0] = height_pad;
padSize[0,1] = width_pad;
padSize[0,2] = depth_pad;

Starting_shape = np.shape(IM_DATA_norm)
a1 = padSize[0,0]
a2 = padSize[0,1]
a3 = padSize[0,2]
im_pad = np.zeros([int(a1),int(a2),int(a3)])

im_pad[:IM_DATA_norm.shape[0],:IM_DATA_norm.shape[1],:IM_DATA_norm.shape[2]] =
IM_DATA_norm[:,:,:]
```

```
im_pad = np.asarray(im_pad).reshape((im_pad.shape[0],
im_pad.shape[1],im_pad.shape[2],1))
[height_pad, width_pad, depth_pad, nChannel_pad] = np.shape(im_pad);

#Preallocate some matrices to catch the probability maps
out_Uncertainty_Scores = np.zeros([height_pad, width_pad, depth_pad],'double');
out_scores_from_network_all_classes = np.zeros([height_pad, width_pad,
depth_pad, 4], 'double');


#Loop through blocks of 'patchSize'
for loop_height in range(1,number_of_height_patches+1):

    for loop_width in range(1,number_of_width_patches+1):

        for loop_depth in range(1,number_of_depth_patches+1):
            PP3 = np.empty([1,64,64,32,1])
            start_height_position=(loop_height-1)*patchSize[0];
            end_height_position=loop_height*patchSize[0];

            start_width_position=(loop_width-1)*patchSize[1];
            end_width_position=loop_width*patchSize[1];

            start_depth_position=(loop_depth-1)*patchSize[2];
            end_depth_position=loop_depth*patchSize[2];


patch_to_deploy=im_pad[start_height_position:end_height_position,start_width_pos
ition:end_width_position,start_depth_position:end_depth_position,:];
            PP3[0,:,:,:]=patch_to_deploy
            # deploy net
            predictions = pretrained_model.predict(PP3)

            scores = np.max(predictions,axis=4)
            predictions.argmax(axis=3)




out_Uncertainty_Scores[start_height_position:end_height_position,start_width_pos
ition:end_width_position,start_depth_position:end_depth_position]=scores


out_scores_from_network_all_classes[start_height_position:end_height_position,st
art_width_position:end_width_position,start_depth_position:end_depth_position,0]
=predictions[0,:,:,:,0];

out_scores_from_network_all_classes[start_height_position:end_height_position,st
art_width_position:end_width_position,start_depth_position:end_depth_position,1]
=predictions[0,:,:,:,1];

out_scores_from_network_all_classes[start_height_position:end_height_position,st
art_width_position:end_width_position,start_depth_position:end_depth_position,2]
=predictions[0,:,:,:,2];

out_scores_from_network_all_classes[start_height_position:end_height_position,st
art_width_position:end_width_position,start_depth_position:end_depth_position,3]
=predictions[0,:,:,:,3];
```

```
#Script is general for n classes to this point
out_Pmap_cat1 = out_scores_from_network_all_classes[:,:,:,0];
out_Pmap_cat2 = out_scores_from_network_all_classes[:,:,:,1];
out_Pmap_cat3 = out_scores_from_network_all_classes[:,:,:,2];

out_Uncertainty_Scores = out_Uncertainty_Scores[0:height, 0:width, 0:depth];

#Remove padding from probability maps
out_Pmap_cat1 = out_Pmap_cat1[0:height, 0:width, 0:depth];
out_Pmap_cat2 = out_Pmap_cat2[0:height, 0:width, 0:depth];
out_Pmap_cat3 = out_Pmap_cat3[0:height, 0:width, 0:depth];

#%%visualise network outputs
fig, (ax1, ax2, ax3, ax4) = plt.subplots(1, 4, sharey=True)
fig.set_size_inches(14, 12)
im = ax1.imshow(out_Pmap_cat1[:,:,59])
ax1.title.set_text('Background/Other')
im = ax2.imshow(out_Pmap_cat2[:,:,59])
ax2.title.set_text('LF-nuclei')
im = ax3.imshow(out_Pmap_cat3[:,:,59])
ax3.title.set_text('LF-actin')
im = ax4.imshow(out_Uncertainty_Scores[:,:,59])
ax4.title.set_text('Uncertainty')
plt.savefig('Predictions.png')

#%%Map to 16-bit and save for loading into CellProfiler
data_norm_holder = np.zeros((1024, 1024, 107),np.uint16)
ui16_PMAP_cat1_xy = cv2.normalize(out_Pmap_cat1, data_norm_holder , 65535.0,
0.0, cv2.NORM_MINMAX)
data_norm_holder = np.zeros((1024, 1024, 107),np.uint16)
ui16_PMAP_cat2_xy = cv2.normalize(out_Pmap_cat2, data_norm_holder , 65535.0,
0.0, cv2.NORM_MINMAX)
data_norm_holder = np.zeros((1024, 1024, 107),np.uint16)
ui16_PMAP_cat3_xy = cv2.normalize(out_Pmap_cat3, data_norm_holder , 65535.0,
0.0, cv2.NORM_MINMAX)
data_to_save = np.zeros((1024,1024,107,3))
data_to_save[:,:,:,0] = ui16_PMAP_cat1_xy
data_to_save[:,:,:,1] = ui16_PMAP_cat2_xy
data_to_save[:,:,:,2] = ui16_PMAP_cat3_xy

#%% write files out as multipage tiff
for channel in range(0,3):
    image_to_save = (data_to_save[:,:,:,channel]).astype(dtype=np.uint16)
    rearranged_image_to_save = np.transpose(image_to_save, axes=[2,0,1])

imageio.mimwrite('Ch_'+str(channel+3)+'_lm_001'+'.tiff',rearranged_image_to_sav
e)
```

Extracting single-cell features using label-free cell segmentation and CellProfiler 4

- Download and install CellProfiler4 from the CellProfiler website.
https://cellprofiler.org

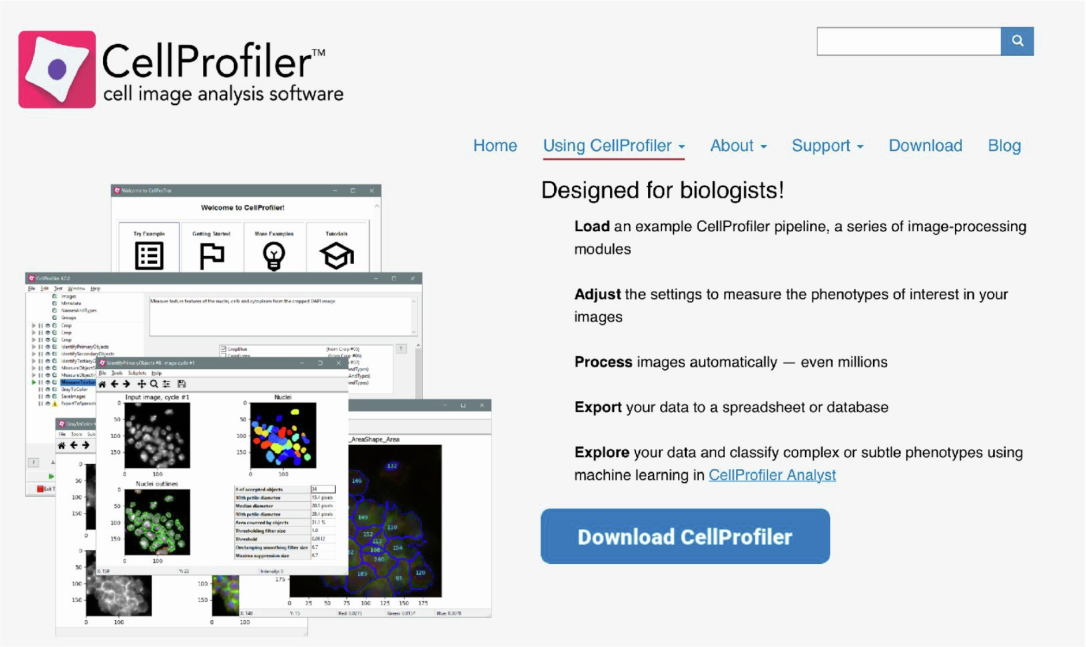

1. 2-D CellProfiler Pipeline
-A screencast video is included with the BioStudies archive.
- Folder 5 “5_CellProfiler_cell_measurements” in the 2-D BioStudies project archive contains example data, a CellProfiler pipeline and the subsequent CellProfiler single-cell outputs:

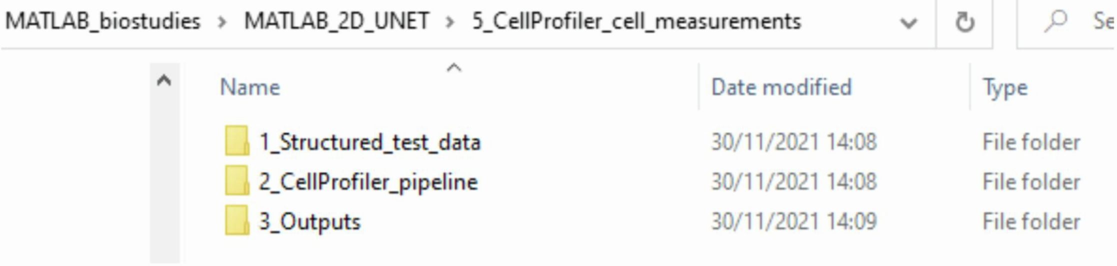

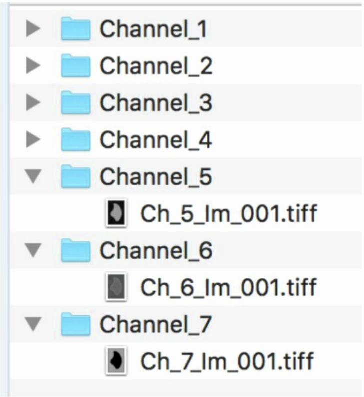

- The image-data (inside 1_Structured_test_data) contains four immunofluorescence channels followed by the probability maps obtained for the label-free nuclei, actin and background/other classes from either the Python or MATLAB deep learning scripts (channels 5, 6 and 7, respectively).

- Open CellProfiler 4 and load the image analysis pipeline from the "2_CellProfiler_pipeline" folder.
- Drag-and-drop the "1_structured_test_data" folder into the CellProfiler image-window to load the 2-D image-data:

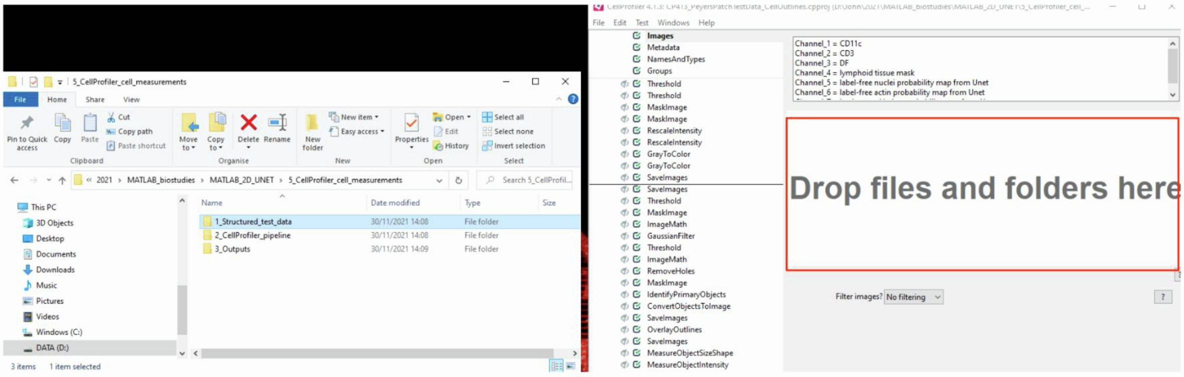

- Choose where to save the outputted cell features by clicking on the "Output Settings" tab at the bottom-left of the CellProfiler dialogue.

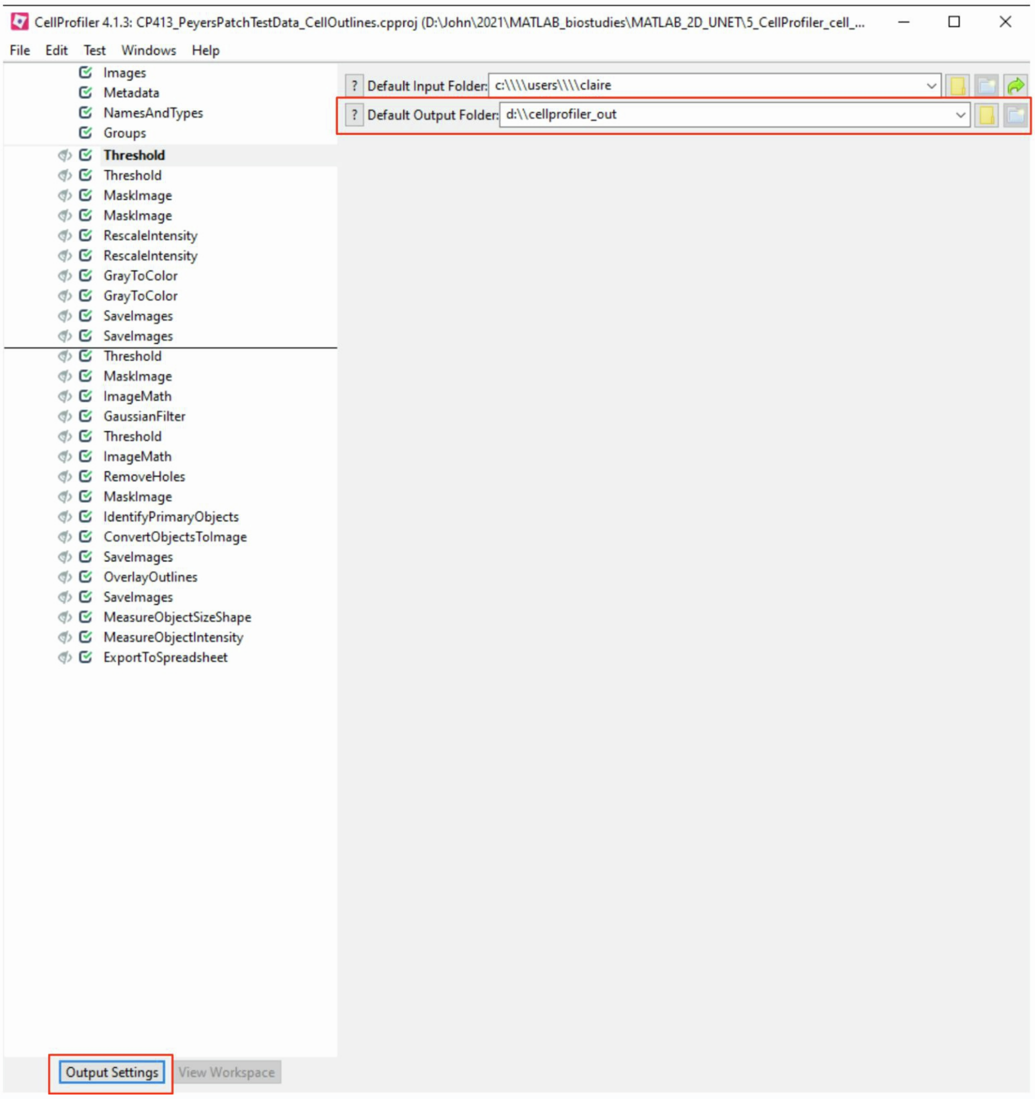

- To run the CellProfiler pipeline and save the cell features and other pipeline outputs at the specified location, click “Analyse Images” at the bottom-left of the CellProfiler screen.

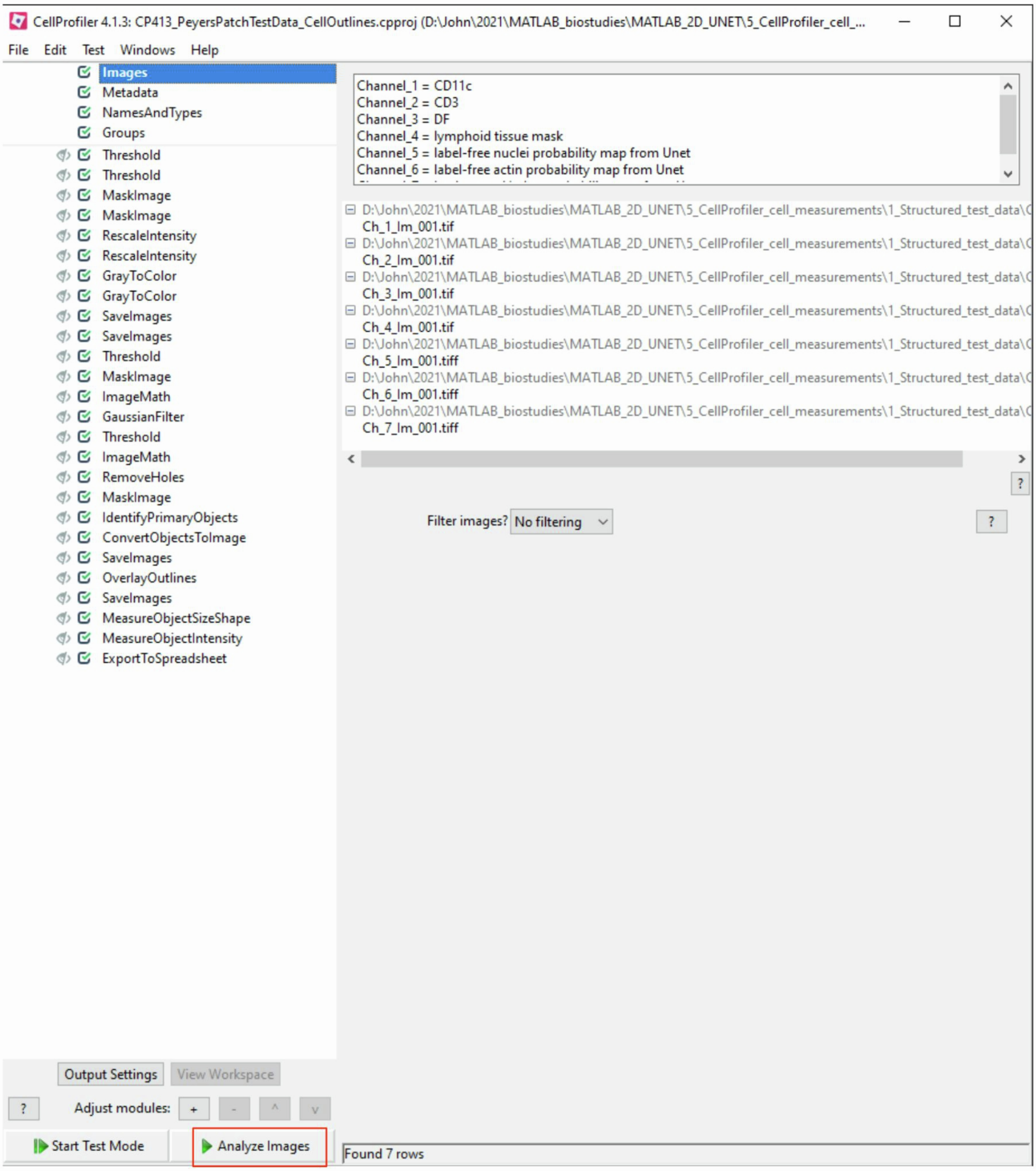

- Previously-saved CellProfiler outputs are also available inside the “3_Outputs” folder:

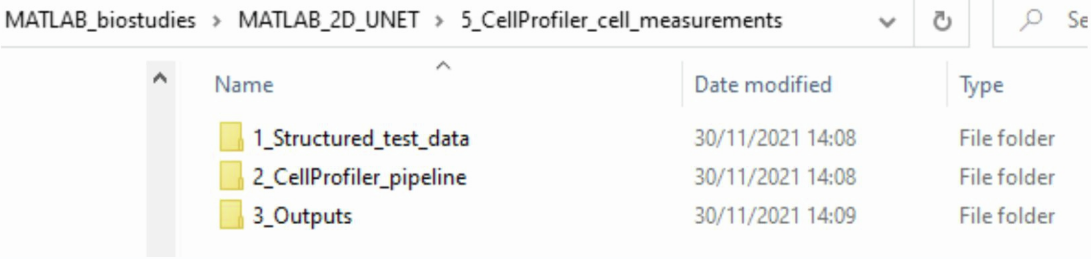

2. 3-D CellProfiler Pipeline
-A screencast video is included with the BioStudies archive.
- Folder 5 “5_CellProfiler_cell_measurements” in the 3-D BioStudies project archive contains example data, a 3-D CellProfiler pipeline and the subsequent CellProfiler single-cell outputs:

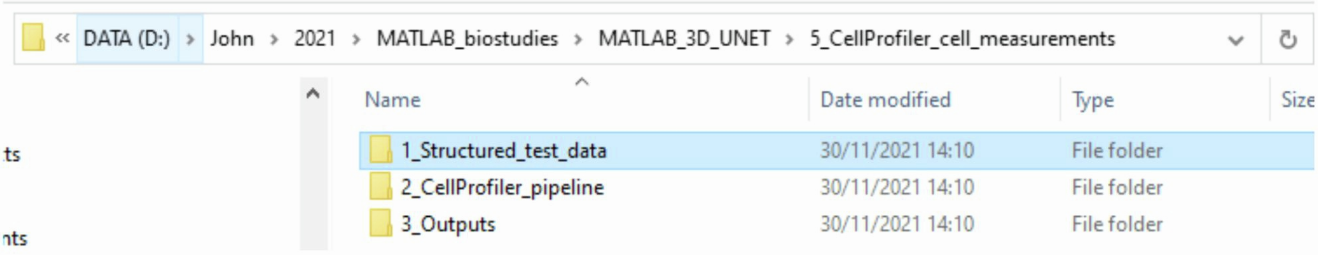

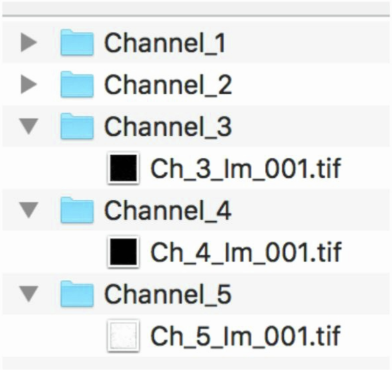

- The image-data (inside 1_Structured_test_data) contains two immunofluorescence channels followed by the 3-D probability maps obtained for the label-free nuclei, actin and background/other classes from either the Python or MATLAB deep learning scripts (channels 3, 4 and 5, respectively).

- Open CellProfiler 4 and load the image analysis pipeline from the “2_CellProfiler_pipeline” folder.
- Drag-and-drop the “1_structured_test_data” folder into the CellProfiler image-window to load the 2-D image-data:

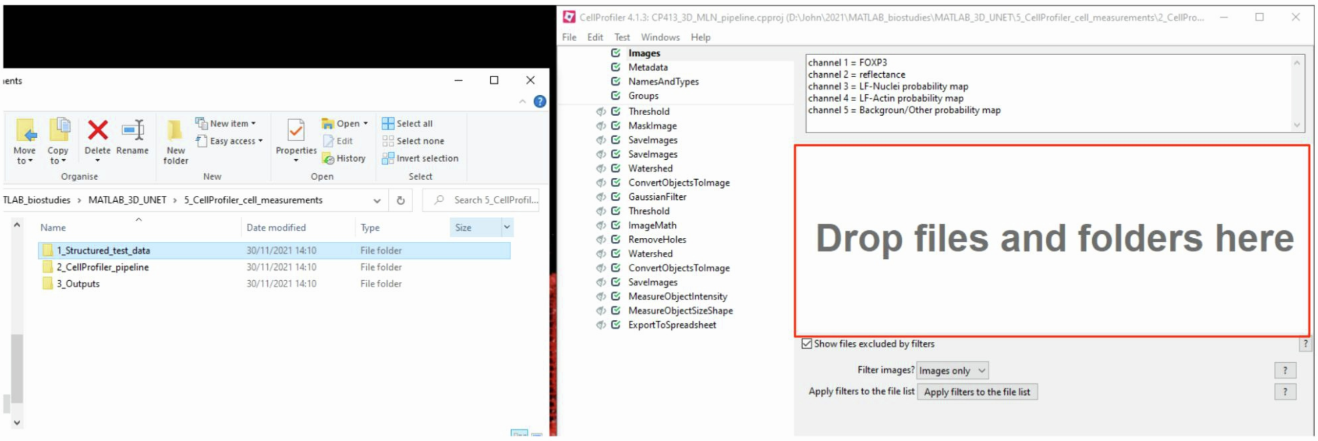

- Choose where to save the outputted cell features by clicking on the "Output Settings" tab at the bottom-left of the CellProfiler dialogue.

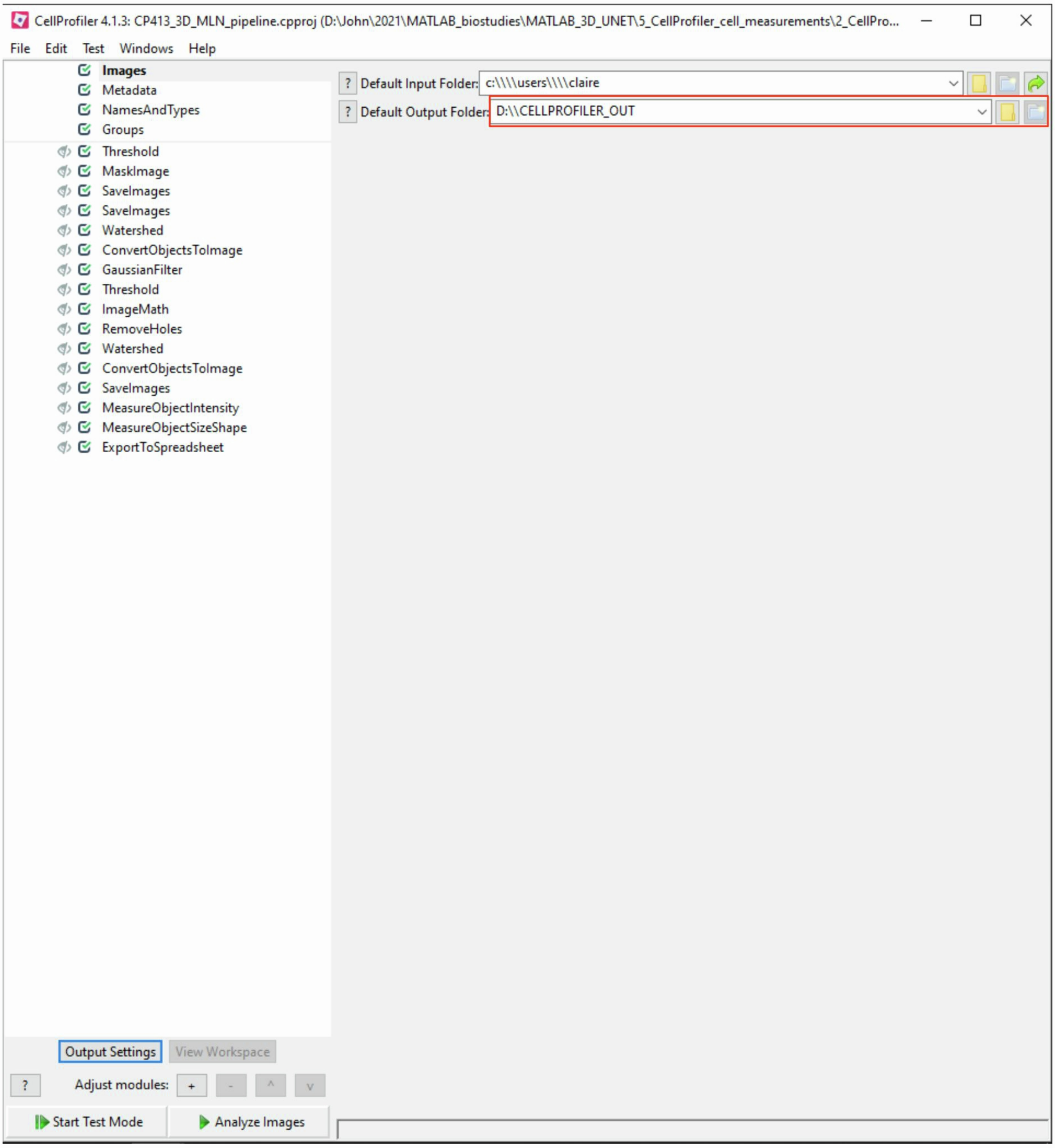

- To run the CellProfiler pipeline and save the cell features and other pipeline outputs at the specified location, click "Analyse Images" at the bottom-left of the CellProfiler screen.

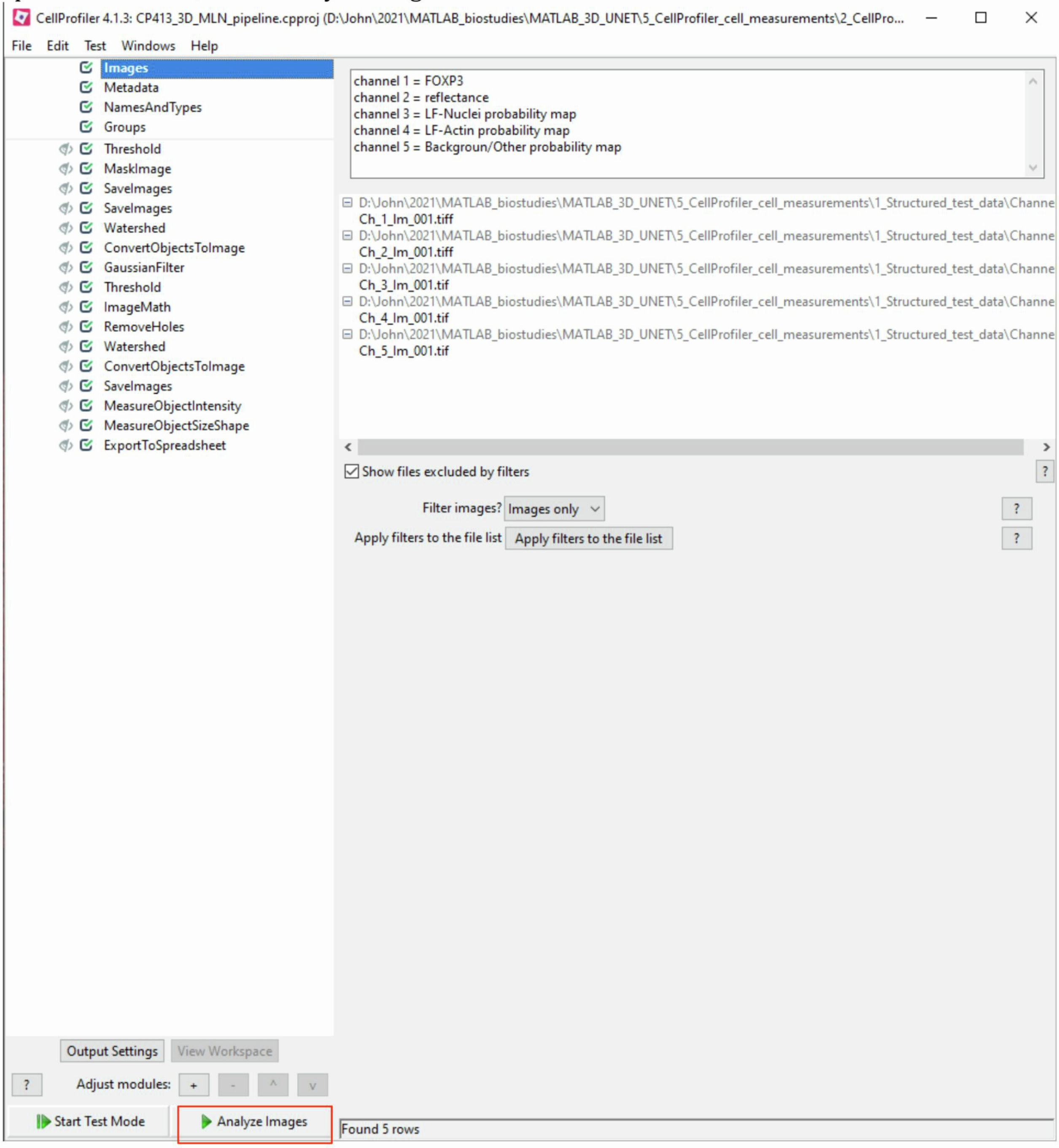

- Previously-saved CellProfiler outputs are also available inside the "3_Outputs" folder:

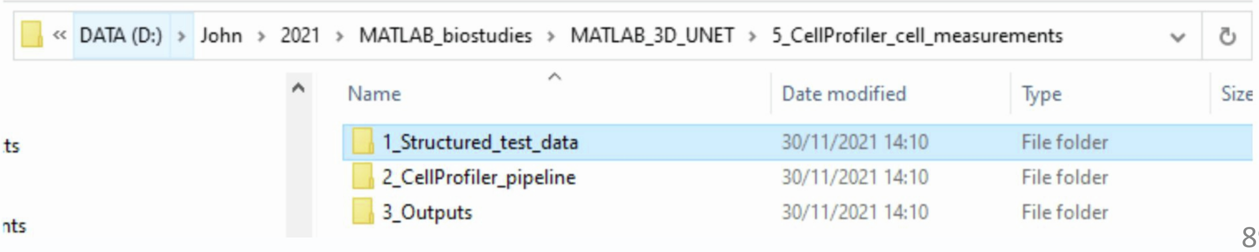

2-D Cell Profiler image analysis pipelines. This section presents screenshots of a CellProfiler image analysis pipeline used to achieve label-free cell segmentation in 2-D from the Unet network outputs, and to measure the intensity and size/shape features of identified cell-objects. To use the image analysis pipeline with new image data, the 'IdentifyPrimaryObjects' module simply needs adjusting so that the 'typical diameter of objects' size-range matches the pixel scaling of the new images. For newcomers to CellProfiler, we recommend downloading the image-data and pipeline from BioStudies database https://www.ebi.ac.uk/biostudies/ under accession number S-BSST742. This enables the pipeline to be run with the data described in the manuscript and allows the user to see how each module works.

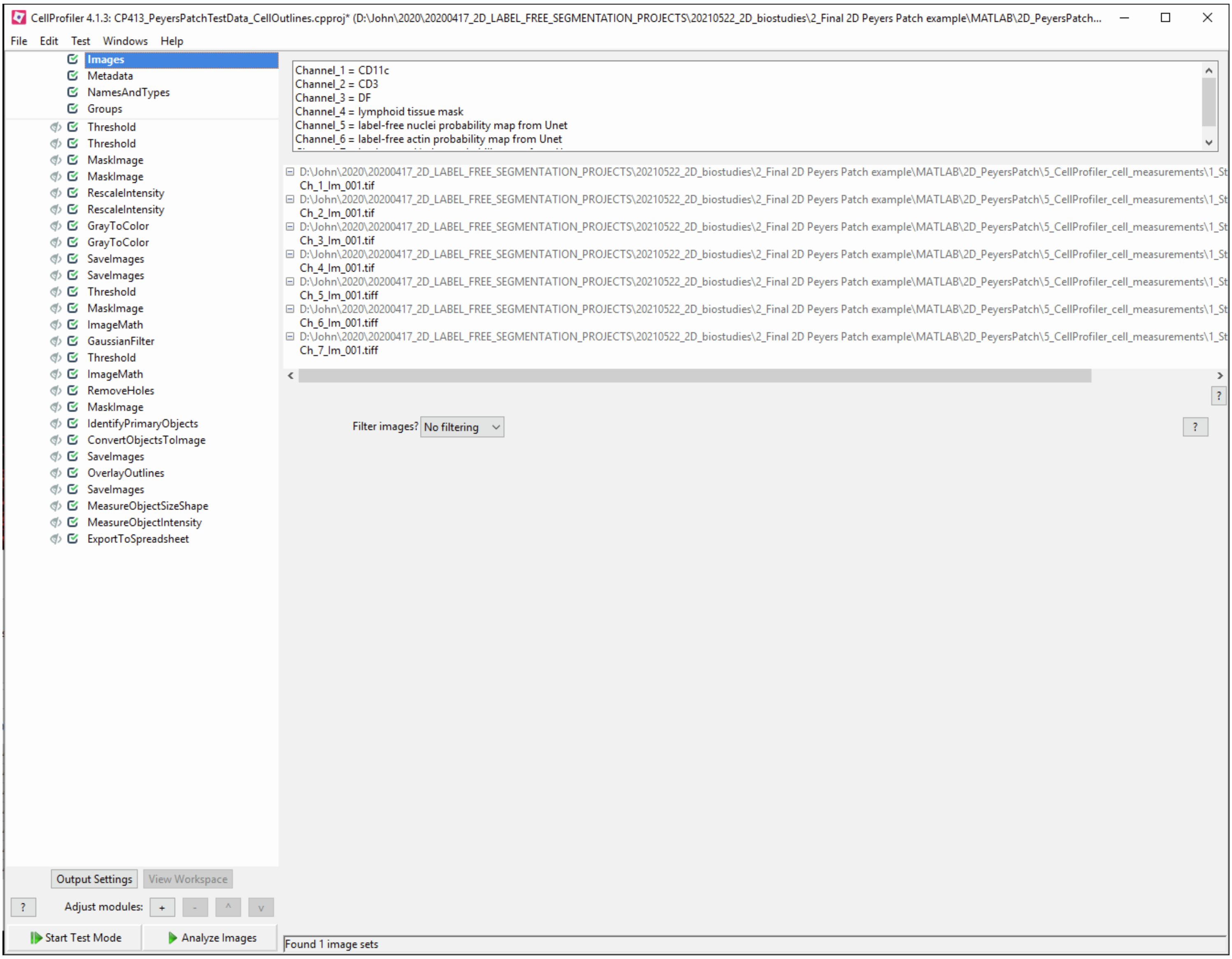

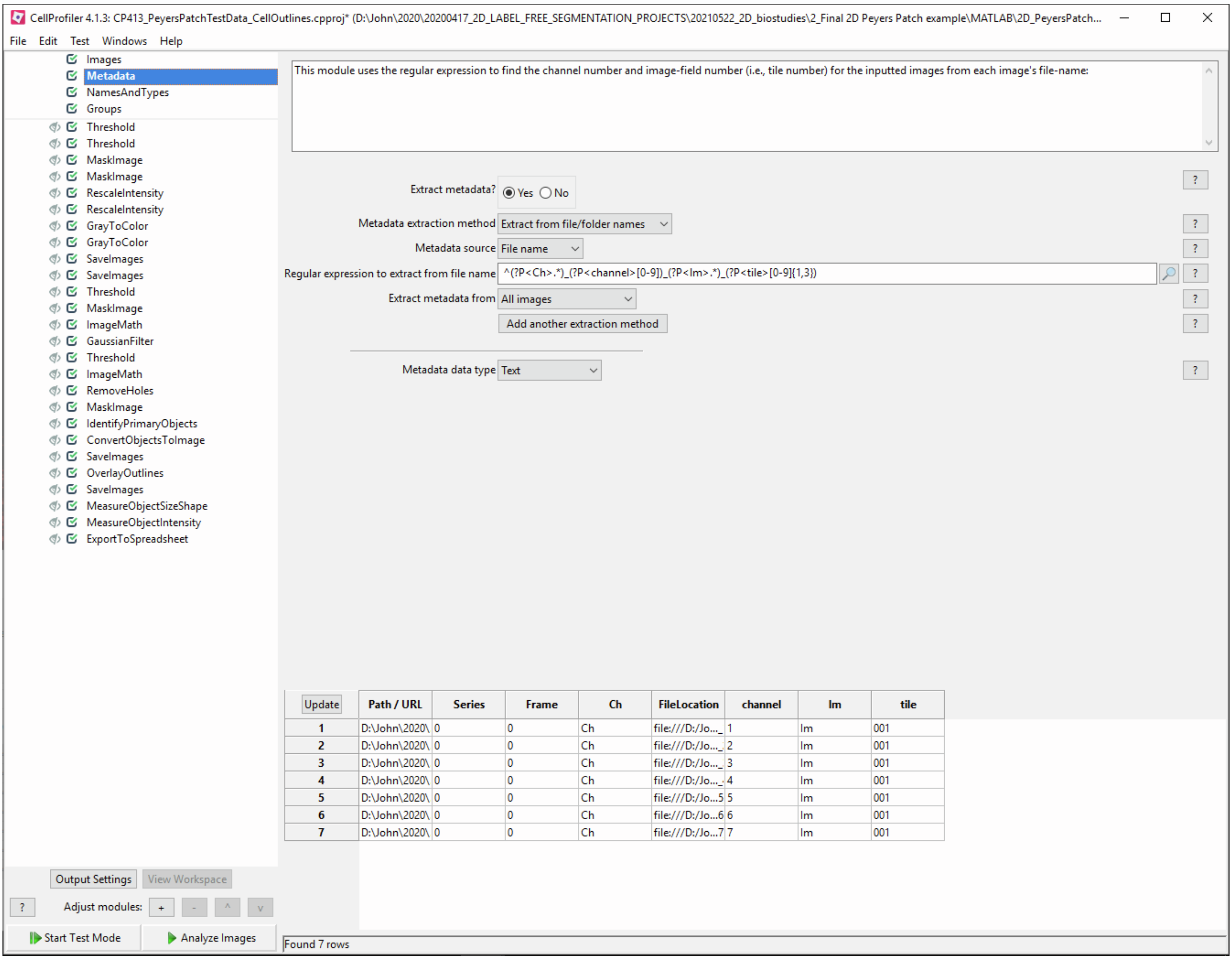
CellProfiler 4.1.3: CP413_PeyersPatchTestData_CellOutlines.cpproj* (D:\John\2020\20200417_2D_LABEL_FREE_SEGMENTATION_PROJECTS\20210522_2D_biostudies\2_Final 2D Peyers Patch example\MATLAB\2D_PeyersPatch...
File Edit Test Windows Help
Images
Metadata
NamesAndTypes
Groups
Threshold
Threshold
MaskImage
MaskImage
RescaleIntensity
RescaleIntensity
GrayToColor
GrayToColor
SaveImages
SaveImages
Threshold
MaskImage
ImageMath
GaussianFilter
Threshold
ImageMath
RemoveHoles
MaskImage
IdentifyPrimaryObjects
ConvertObjectsToImage
SaveImages
OverlayOutlines
SaveImages
MeasureObjectSizeShape
MeasureObjectIntensity
ExportToSpreadsheet
This module uses the regular expression to find the channel number and image-field number (i.e., tile number) for the inputted images from each image's file-name:
Extract metadata? Yes No
Metadata extraction method Extract from file/folder names
Metadata source File name
Regular expression to extract from file name ^(?P<Ch>.*)_(?P<channel>[0-9])_(?P<Im>.*)_(?P<tile>[0-9]{1,3})
Extract metadata from All images
Add another extraction method
Metadata data type Text
Update Path / URL Series Frame Ch FileLocation channel Im tile
1 D:\John\2020\ 0 0 Ch file:///D:/Jo..._ 1 Im 001
2 D:\John\2020\ 0 0 Ch file:///D:/Jo..._ 2 Im 001
3 D:\John\2020\ 0 0 Ch file:///D:/Jo..._ 3 Im 001
4 D:\John\2020\ 0 0 Ch file:///D:/Jo..._ 4 Im 001
5 D:\John\2020\ 0 0 Ch file:///D:/Jo...5 5 Im 001
6 D:\John\2020\ 0 0 Ch file:///D:/Jo...6 6 Im 001
7 D:\John\2020\ 0 0 Ch file:///D:/Jo...7 7 Im 001
Output Settings View Workspace
Adjust modules: + - ^ v
Start Test Mode Analyze Images
Found 7 rows

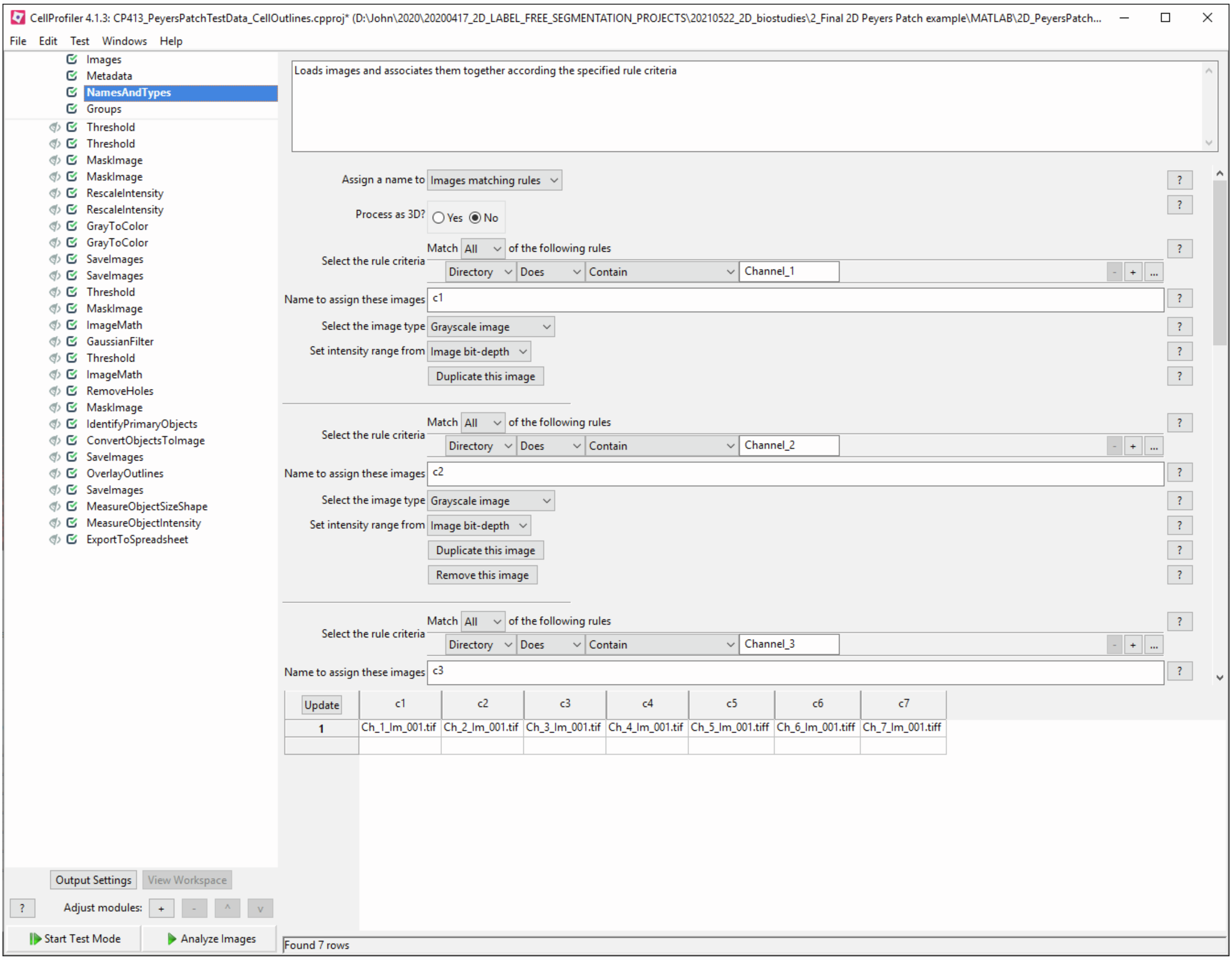
CellProfiler 4.1.3: CP413_PeyersPatchTestData_CellOutlines.cpproj* (D:\John\2020\20200417_2D_LABEL_FREE_SEGMENTATION_PROJECTS\20210522_2D_biostudies\2_Final 2D Peyers Patch example\MATLAB\2D_PeyersPatch...
File Edit Test Windows Help
Images
Metadata
NamesAndTypes
Groups
Threshold
Threshold
MaskImage
MaskImage
RescaleIntensity
RescaleIntensity
GrayToColor
GrayToColor
SaveImages
SaveImages
Threshold
MaskImage
ImageMath
GaussianFilter
Threshold
ImageMath
RemoveHoles
MaskImage
IdentifyPrimaryObjects
ConvertObjectsToImage
SaveImages
OverlayOutlines
SaveImages
MeasureObjectSizeShape
MeasureObjectIntensity
ExportToSpreadsheet
Loads images and associates them together according the specified rule criteria
Assign a name to Images matching rules
Process as 3D? Yes No
Match All of the following rules
Select the rule criteria Directory Does Contain Channel_1
Name to assign these images c1
Select the image type Grayscale image
Set intensity range from Image bit-depth
Duplicate this image
Match All of the following rules
Select the rule criteria Directory Does Contain Channel_2
Name to assign these images c2
Select the image type Grayscale image
Set intensity range from Image bit-depth
Duplicate this image
Remove this image
Match All of the following rules
Select the rule criteria Directory Does Contain Channel_3
Name to assign these images c3
Update c1 c2 c3 c4 c5 c6 c7
1 Ch_1_Im_001.tif Ch_2_Im_001.tif Ch_3_Im_001.tif Ch_4_Im_001.tif Ch_5_Im_001.tiff Ch_6_Im_001.tiff Ch_7_Im_001.tiff
Output Settings View Workspace
Adjust modules:
Start Test Mode Analyze Images
Found 7 rows

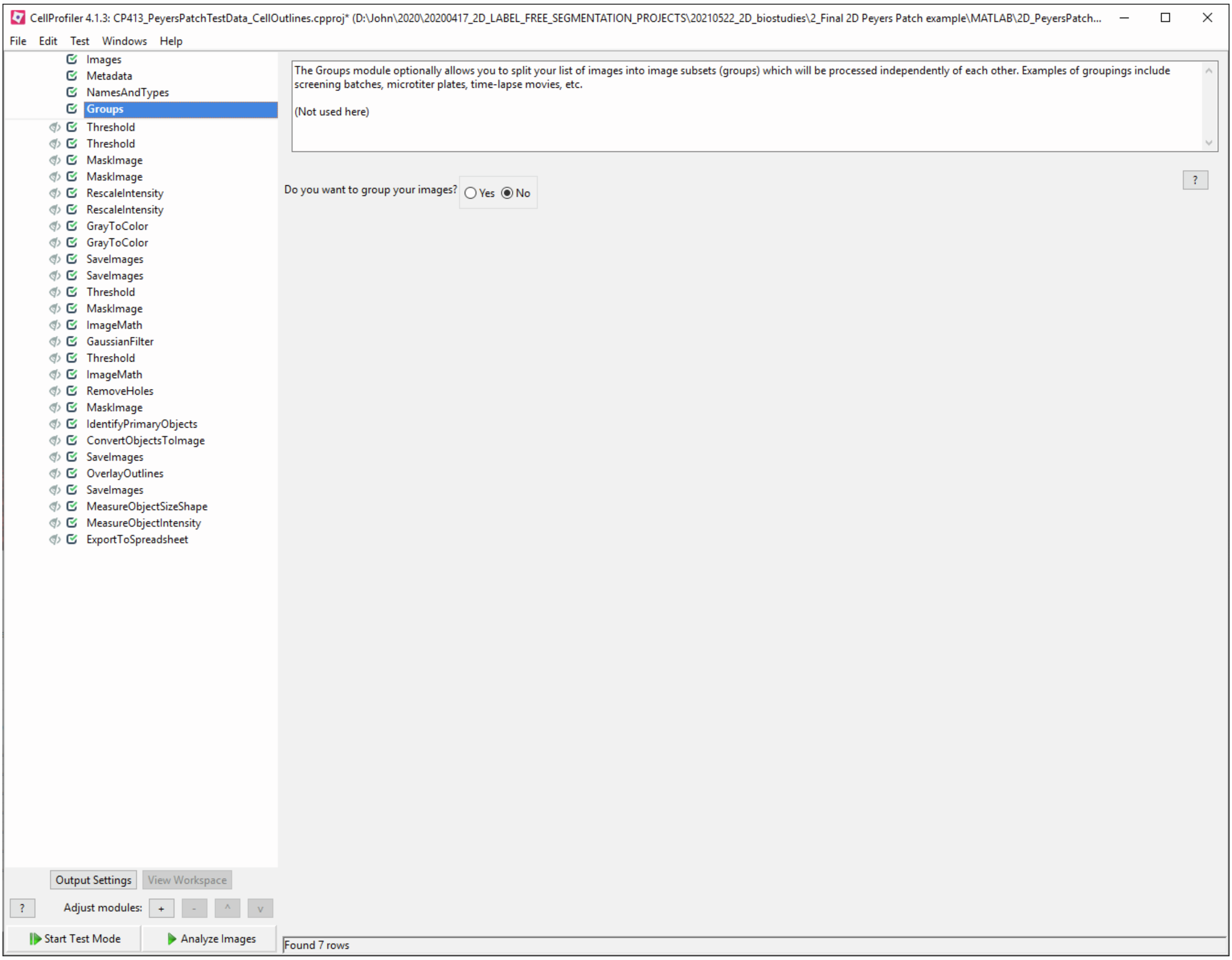
CellProfiler 4.1.3: CP413_PeyersPatchTestData_CellOutlines.cpproj* (D:\John\2020\20200417_2D_LABEL_FREE_SEGMENTATION_PROJECTS\20210522_2D_biostudies\2_Final 2D Peyers Patch example\MATLAB\2D_PeyersPatch...
File Edit Test Windows Help
Images
Metadata
NamesAndTypes
Groups
Threshold
Threshold
MaskImage
MaskImage
RescaleIntensity
RescaleIntensity
GrayToColor
GrayToColor
SaveImages
SaveImages
Threshold
MaskImage
ImageMath
GaussianFilter
Threshold
ImageMath
RemoveHoles
MaskImage
IdentifyPrimaryObjects
ConvertObjectsToImage
SaveImages
OverlayOutlines
SaveImages
MeasureObjectSizeShape
MeasureObjectIntensity
ExportToSpreadsheet
The Groups module optionally allows you to split your list of images into image subsets (groups) which will be processed independently of each other. Examples of groupings include screening batches, microtiter plates, time-lapse movies, etc.
(Not used here)
Do you want to group your images? Yes No
Output Settings
View Workspace
Adjust modules:
Start Test Mode
Analyze Images
Found 7 rows

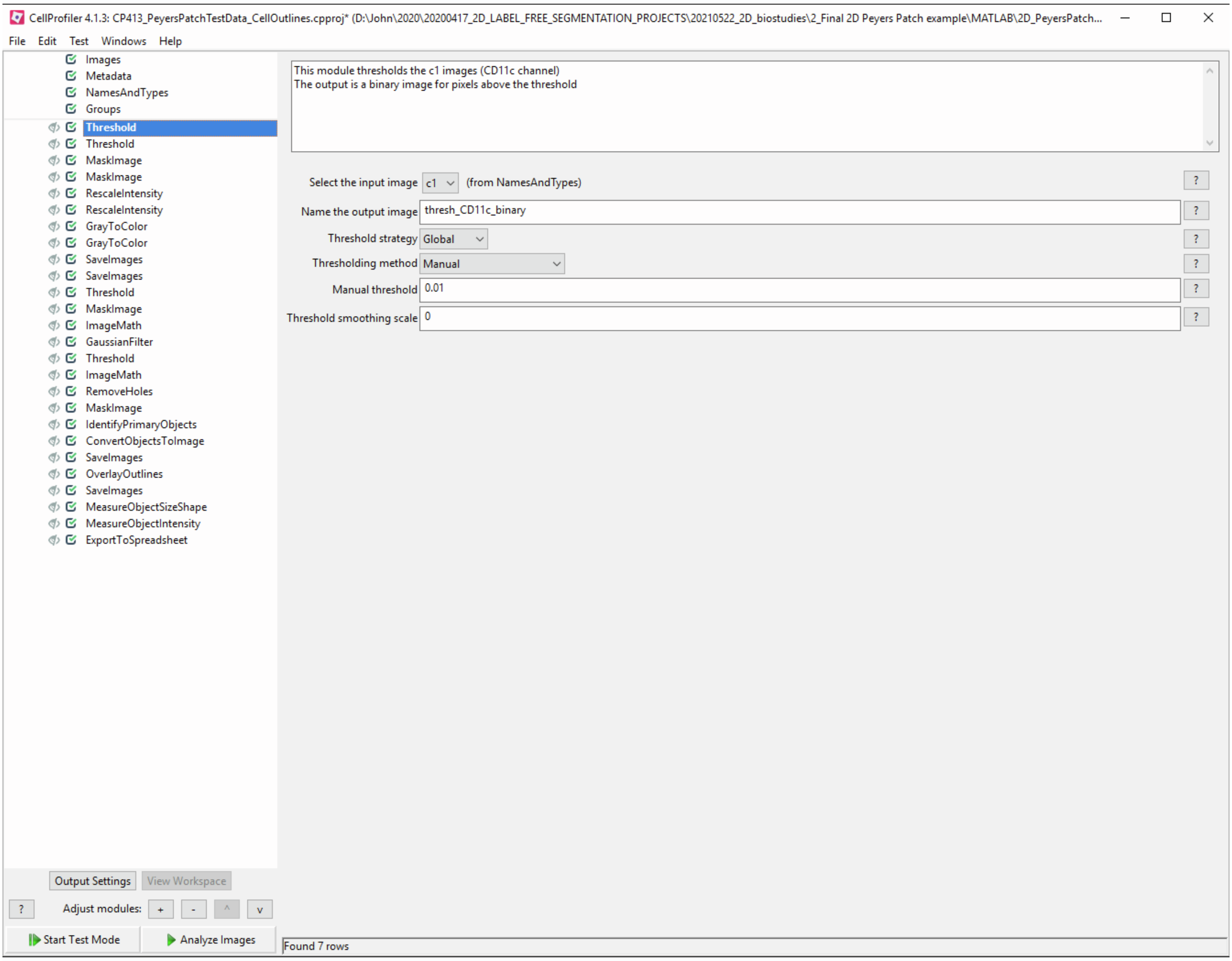
CellProfiler 4.1.3: CP413_PeyersPatchTestData_CellOutlines.cpproj* (D:\John\2020\20200417_2D_LABEL_FREE_SEGMENTATION_PROJECTS\20210522_2D_biostudies\2_Final 2D Peyers Patch example\MATLAB\2D_PeyersPatch...
File Edit Test Windows Help
Images
Metadata
NamesAndTypes
Groups
Threshold
Threshold
MaskImage
MaskImage
RescaleIntensity
RescaleIntensity
GrayToColor
GrayToColor
SaveImages
SaveImages
Threshold
MaskImage
ImageMath
GaussianFilter
Threshold
ImageMath
RemoveHoles
MaskImage
IdentifyPrimaryObjects
ConvertObjectsToImage
SaveImages
OverlayOutlines
SaveImages
MeasureObjectSizeShape
MeasureObjectIntensity
ExportToSpreadsheet
This module thresholds the c1 images (CD11c channel)
The output is a binary image for pixels above the threshold
Select the input image c1 (from NamesAndTypes)
Name the output image thresh_CD11c_binary
Threshold strategy Global
Thresholding method Manual
Manual threshold 0.01
Threshold smoothing scale 0
Output Settings
View Workspace
Adjust modules: + - ^ v
Start Test Mode
Analyze Images
Found 7 rows

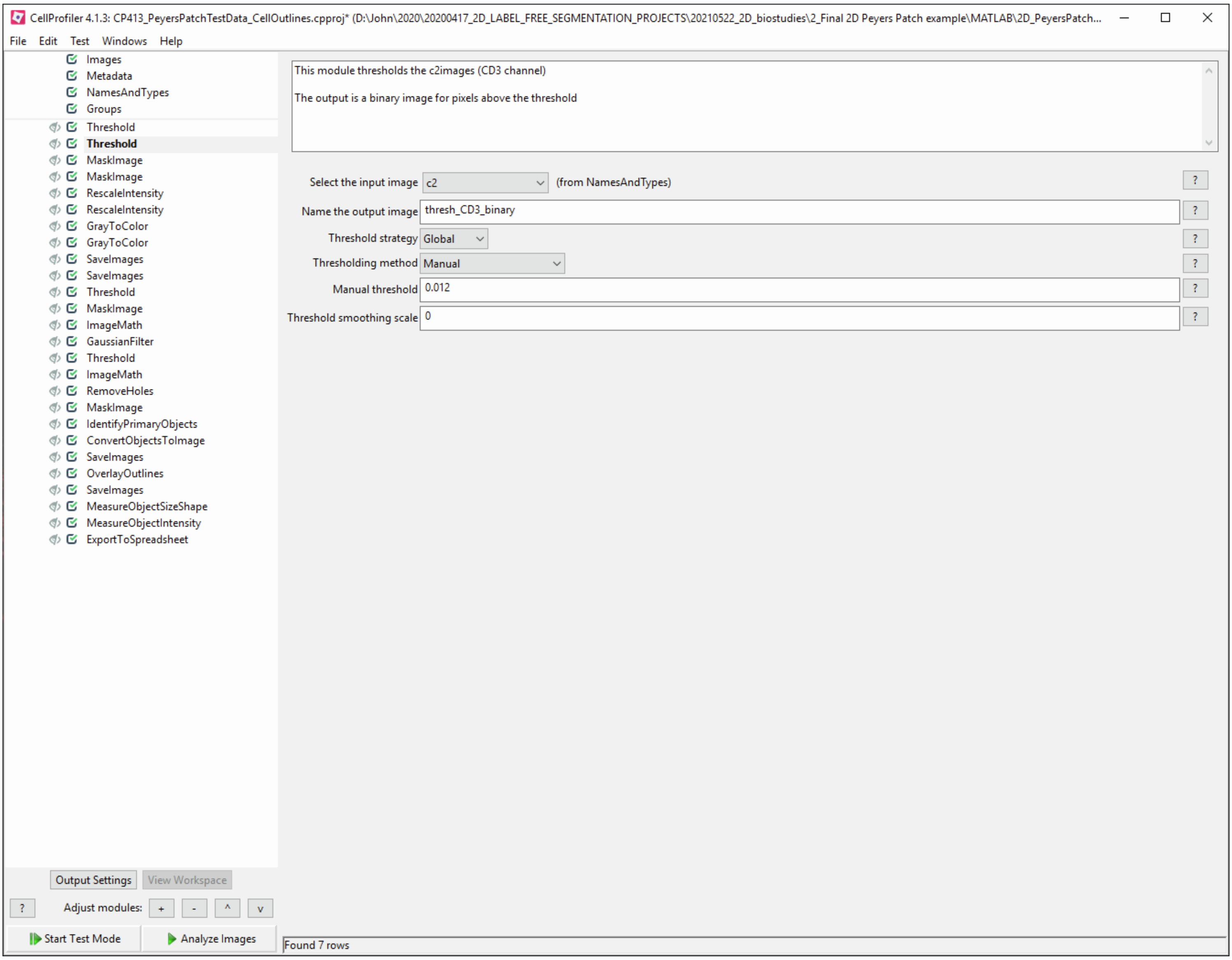
CellProfiler 4.1.3: CP413_PeyersPatchTestData_CellOutlines.cpproj* (D:\John\2020\20200417_2D_LABEL_FREE_SEGMENTATION_PROJECTS\20210522_2D_biostudies\2_Final 2D Peyers Patch example\MATLAB\2D_PeyersPatch...
File Edit Test Windows Help
Images
Metadata
NamesAndTypes
Groups
Threshold
Threshold
MaskImage
MaskImage
RescaleIntensity
RescaleIntensity
GrayToColor
GrayToColor
SaveImages
SaveImages
Threshold
MaskImage
ImageMath
GaussianFilter
Threshold
ImageMath
RemoveHoles
MaskImage
IdentifyPrimaryObjects
ConvertObjectsToImage
SaveImages
OverlayOutlines
SaveImages
MeasureObjectSizeShape
MeasureObjectIntensity
ExportToSpreadsheet
This module thresholds the c2images (CD3 channel)
The output is a binary image for pixels above the threshold
Select the input image c2 (from NamesAndTypes)
Name the output image thresh_CD3_binary
Threshold strategy Global
Thresholding method Manual
Manual threshold 0.012
Threshold smoothing scale 0
Output Settings
View Workspace
Adjust modules: + - ^ v
Start Test Mode
Analyze Images
Found 7 rows

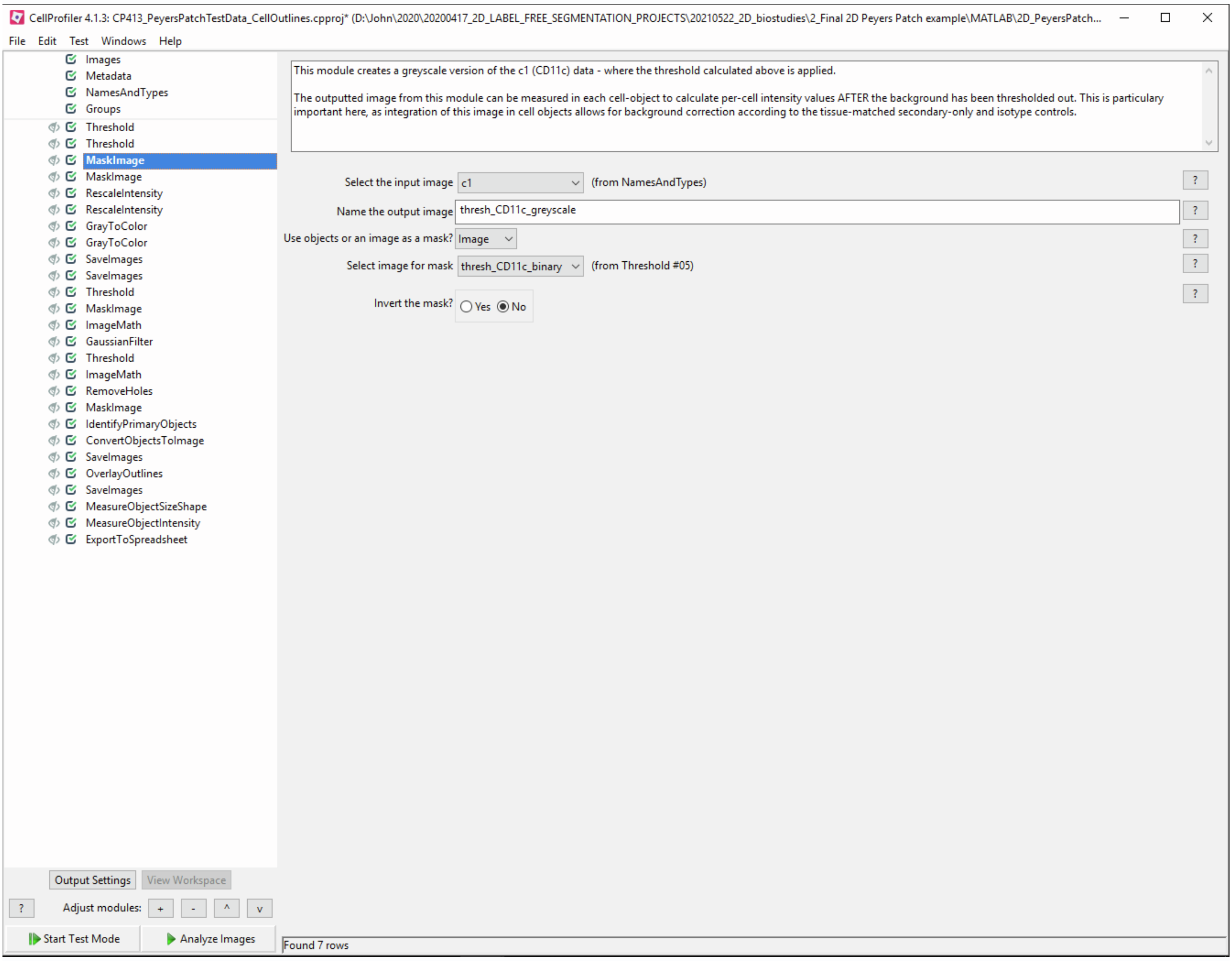
CellProfiler 4.1.3: CP413_PeyersPatchTestData_CellOutlines.cpproj* (D:\John\2020\20200417_2D_LABEL_FREE_SEGMENTATION_PROJECTS\20210522_2D_biostudies\2_Final 2D Peyers Patch example\MATLAB\2D_PeyersPatch...
File Edit Test Windows Help
Images
Metadata
NamesAndTypes
Groups
Threshold
Threshold
MaskImage
MaskImage
RescaleIntensity
RescaleIntensity
GrayToColor
GrayToColor
SaveImages
SaveImages
Threshold
MaskImage
ImageMath
GaussianFilter
Threshold
ImageMath
RemoveHoles
MaskImage
IdentifyPrimaryObjects
ConvertObjectsToImage
SaveImages
OverlayOutlines
SaveImages
MeasureObjectSizeShape
MeasureObjectIntensity
ExportToSpreadsheet
This module creates a greyscale version of the c1 (CD11c) data - where the threshold calculated above is applied.
The outputted image from this module can be measured in each cell-object to calculate per-cell intensity values AFTER the background has been thresholded out. This is particulary important here, as integration of this image in cell objects allows for background correction according to the tissue-matched secondary-only and isotype controls.
Select the input image c1 (from NamesAndTypes)
Name the output image thresh_CD11c_greyscale
Use objects or an image as a mask? Image
Select image for mask thresh_CD11c_binary (from Threshold #05)
Invert the mask? Yes No
Output Settings
View Workspace
Adjust modules: + - ^ v
Start Test Mode
Analyze Images
Found 7 rows

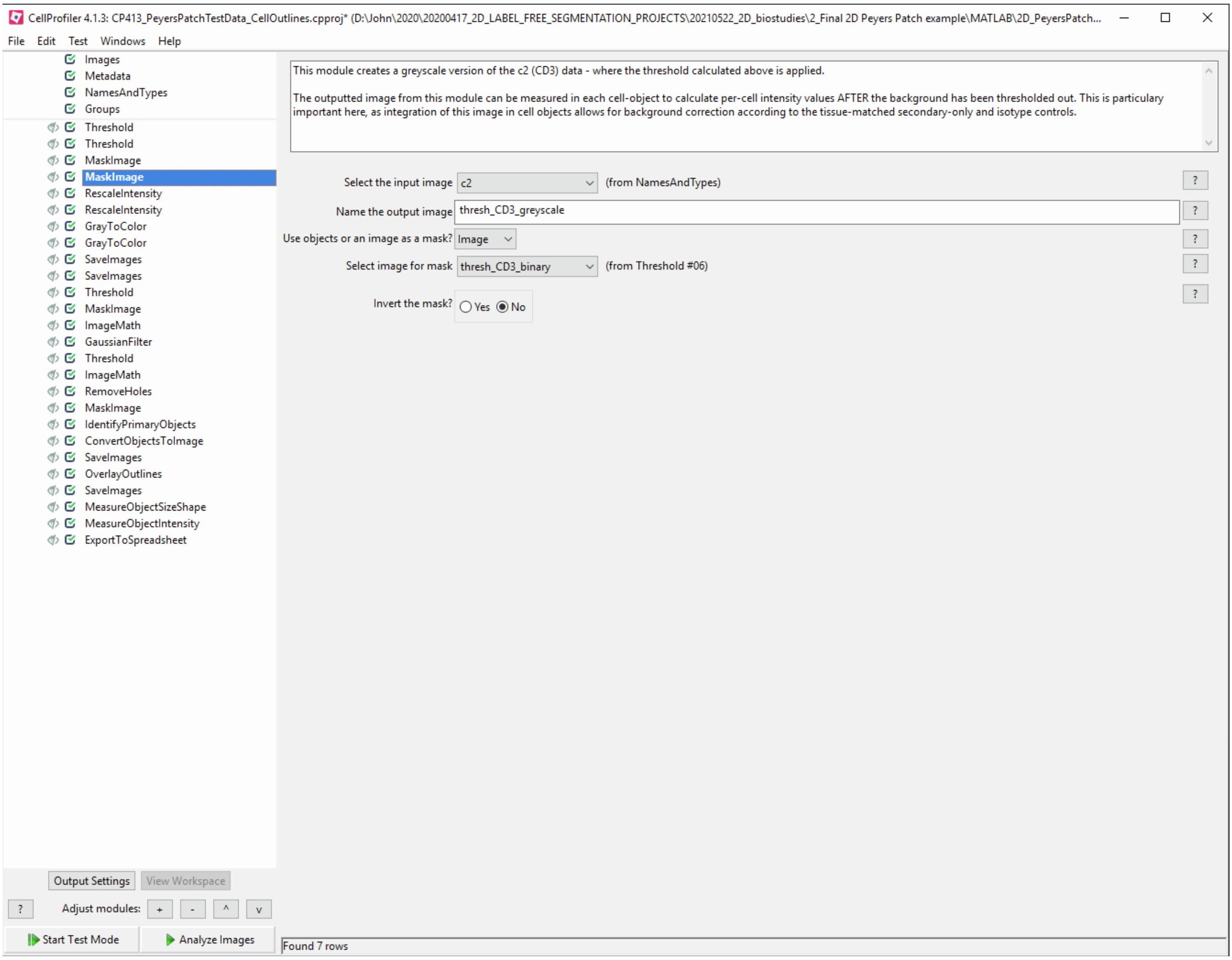
CellProfiler 4.1.3: CP413_PeyersPatchTestData_CellOutlines.cpproj* (D:\John\2020\20200417_2D_LABEL_FREE_SEGMENTATION_PROJECTS\20210522_2D_biostudies\2_Final 2D Peyers Patch example\MATLAB\2D_PeyersPatch...
File Edit Test Windows Help
Images
Metadata
NamesAndTypes
Groups
Threshold
Threshold
MaskImage
MaskImage
RescaleIntensity
RescaleIntensity
GrayToColor
GrayToColor
SaveImages
SaveImages
Threshold
MaskImage
ImageMath
GaussianFilter
Threshold
ImageMath
RemoveHoles
MaskImage
IdentifyPrimaryObjects
ConvertObjectsToImage
SaveImages
OverlayOutlines
SaveImages
MeasureObjectSizeShape
MeasureObjectIntensity
ExportToSpreadsheet
This module creates a greyscale version of the c2 (CD3) data - where the threshold calculated above is applied.
The outputted image from this module can be measured in each cell-object to calculate per-cell intensity values AFTER the background has been thresholded out. This is particulary important here, as integration of this image in cell objects allows for background correction according to the tissue-matched secondary-only and isotype controls.
Select the input image c2 (from NamesAndTypes)
Name the output image thresh_CD3_greyscale
Use objects or an image as a mask? Image
Select image for mask thresh_CD3_binary (from Threshold #06)
Invert the mask? Yes No
Output Settings View Workspace
Adjust modules: + - ^ v
Start Test Mode
Analyze Images
Found 7 rows

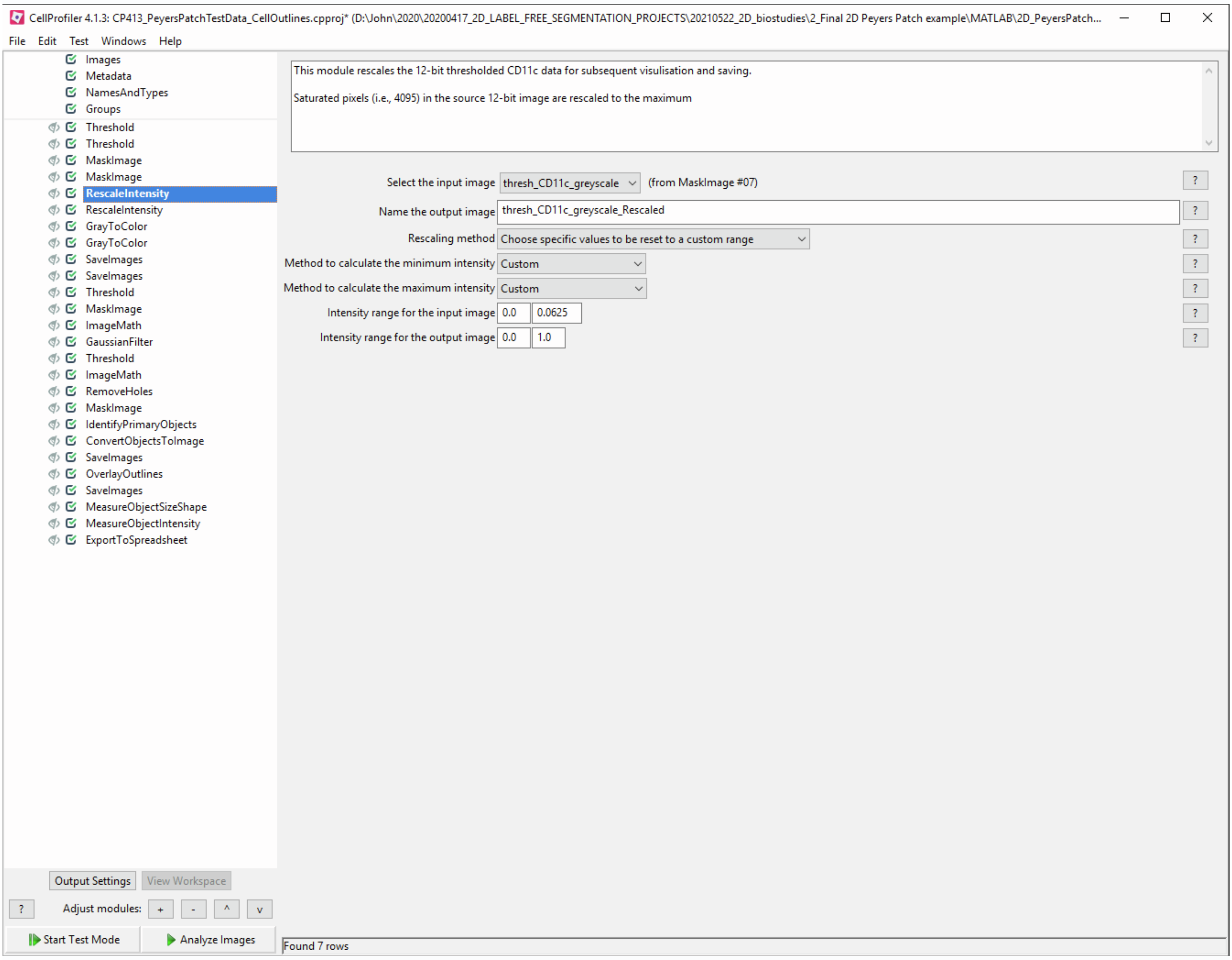
CellProfiler 4.1.3: CP413_PeyersPatchTestData_CellOutlines.cpproj* (D:\John\2020\20200417_2D_LABEL_FREE_SEGMENTATION_PROJECTS\20210522_2D_biostudies\2_Final 2D Peyers Patch example\MATLAB\2D_PeyersPatch...
File Edit Test Windows Help
Images
Metadata
NamesAndTypes
Groups
Threshold
Threshold
MaskImage
MaskImage
RescaleIntensity
RescaleIntensity
GrayToColor
GrayToColor
SaveImages
SaveImages
Threshold
MaskImage
ImageMath
GaussianFilter
Threshold
ImageMath
RemoveHoles
MaskImage
IdentifyPrimaryObjects
ConvertObjectsToImage
SaveImages
OverlayOutlines
SaveImages
MeasureObjectSizeShape
MeasureObjectIntensity
ExportToSpreadsheet
This module rescales the 12-bit thresholded CD11c data for subsequent visulisation and saving.
Saturated pixels (i.e., 4095) in the source 12-bit image are rescaled to the maximum
Select the input image thresh_CD11c_greyscale (from MaskImage #07)
Name the output image thresh_CD11c_greyscale_Rescaled
Rescaling method Choose specific values to be reset to a custom range
Method to calculate the minimum intensity Custom
Method to calculate the maximum intensity Custom
Intensity range for the input image 0.0 0.0625
Intensity range for the output image 0.0 1.0
Output Settings
View Workspace
Adjust modules: + - ^ v
Start Test Mode
Analyze Images
Found 7 rows

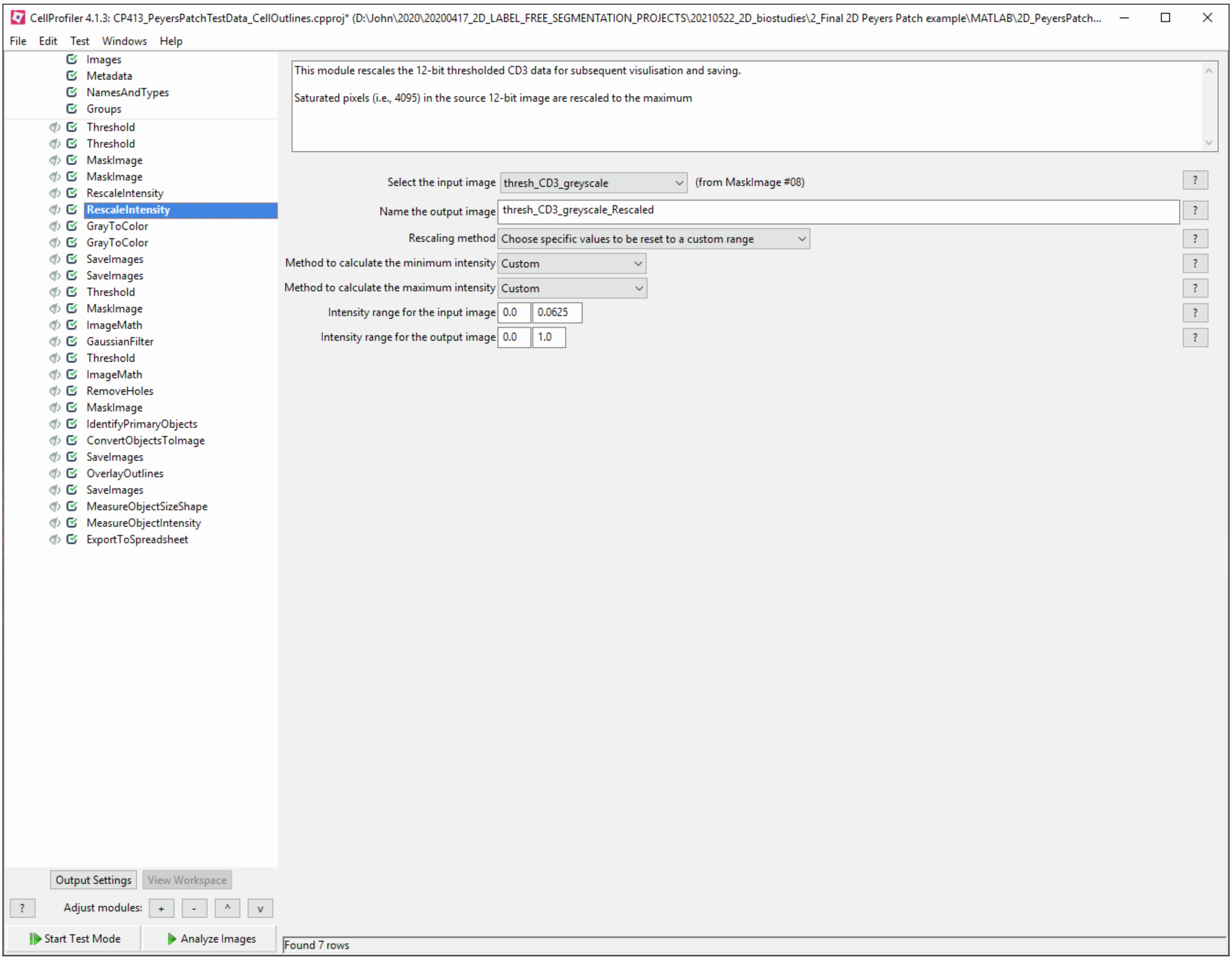
CellProfiler 4.1.3: CP413_PeyersPatchTestData_CellOutlines.cpproj* (D:\John\2020\20200417_2D_LABEL_FREE_SEGMENTATION_PROJECTS\20210522_2D_biostudies\2_Final 2D Peyers Patch example\MATLAB\2D_PeyersPatch...
File Edit Test Windows Help
Images
Metadata
NamesAndTypes
Groups
Threshold
Threshold
MaskImage
MaskImage
RescaleIntensity
RescaleIntensity
GrayToColor
GrayToColor
SaveImages
SaveImages
Threshold
MaskImage
ImageMath
GaussianFilter
Threshold
ImageMath
RemoveHoles
MaskImage
IdentifyPrimaryObjects
ConvertObjectsToImage
SaveImages
OverlayOutlines
SaveImages
MeasureObjectSizeShape
MeasureObjectIntensity
ExportToSpreadsheet
This module rescales the 12-bit thresholded CD3 data for subsequent visulisation and saving.
Saturated pixels (i.e., 4095) in the source 12-bit image are rescaled to the maximum
Select the input image
thresh_CD3_greyscale
(from MaskImage #08)
Name the output image
thresh_CD3_greyscale_Rescaled
Rescaling method
Choose specific values to be reset to a custom range
Method to calculate the minimum intensity
Custom
Method to calculate the maximum intensity
Custom
Intensity range for the input image
0.0
0.0625
Intensity range for the output image
0.0
1.0
Output Settings
View Workspace
Adjust modules:
Start Test Mode
Analyze Images
Found 7 rows

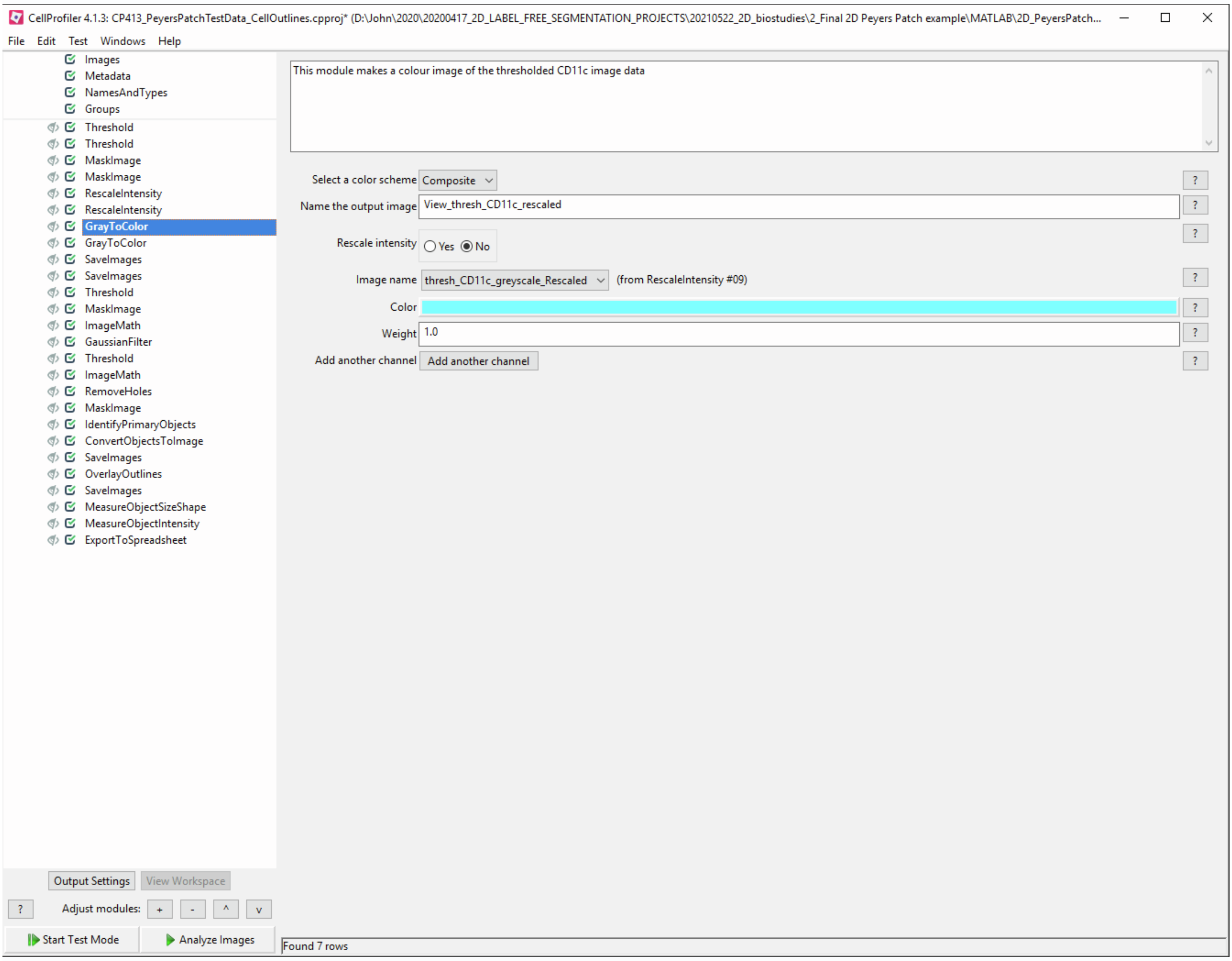
CellProfiler 4.1.3: CP413_PeyersPatchTestData_CellOutlines.cpproj* (D:\John\2020\20200417_2D_LABEL_FREE_SEGMENTATION_PROJECTS\20210522_2D_biostudies\2_Final 2D Peyers Patch example\MATLAB\2D_PeyersPatch...
File Edit Test Windows Help
Images
Metadata
NamesAndTypes
Groups
Threshold
Threshold
MaskImage
MaskImage
RescaleIntensity
RescaleIntensity
GrayToColor
GrayToColor
SaveImages
SaveImages
Threshold
MaskImage
ImageMath
GaussianFilter
Threshold
ImageMath
RemoveHoles
MaskImage
IdentifyPrimaryObjects
ConvertObjectsToImage
SaveImages
OverlayOutlines
SaveImages
MeasureObjectSizeShape
MeasureObjectIntensity
ExportToSpreadsheet
This module makes a colour image of the thresholded CD11c image data
Select a color scheme Composite
Name the output image View_thresh_CD11c_rescaled
Rescale intensity Yes No
Image name thresh_CD11c_greyscale_Rescaled (from RescaleIntensity #09)
Color
Weight 1.0
Add another channel Add another channel
Output Settings View Workspace
Adjust modules: + - ^ v
Start Test Mode
Analyze Images
Found 7 rows

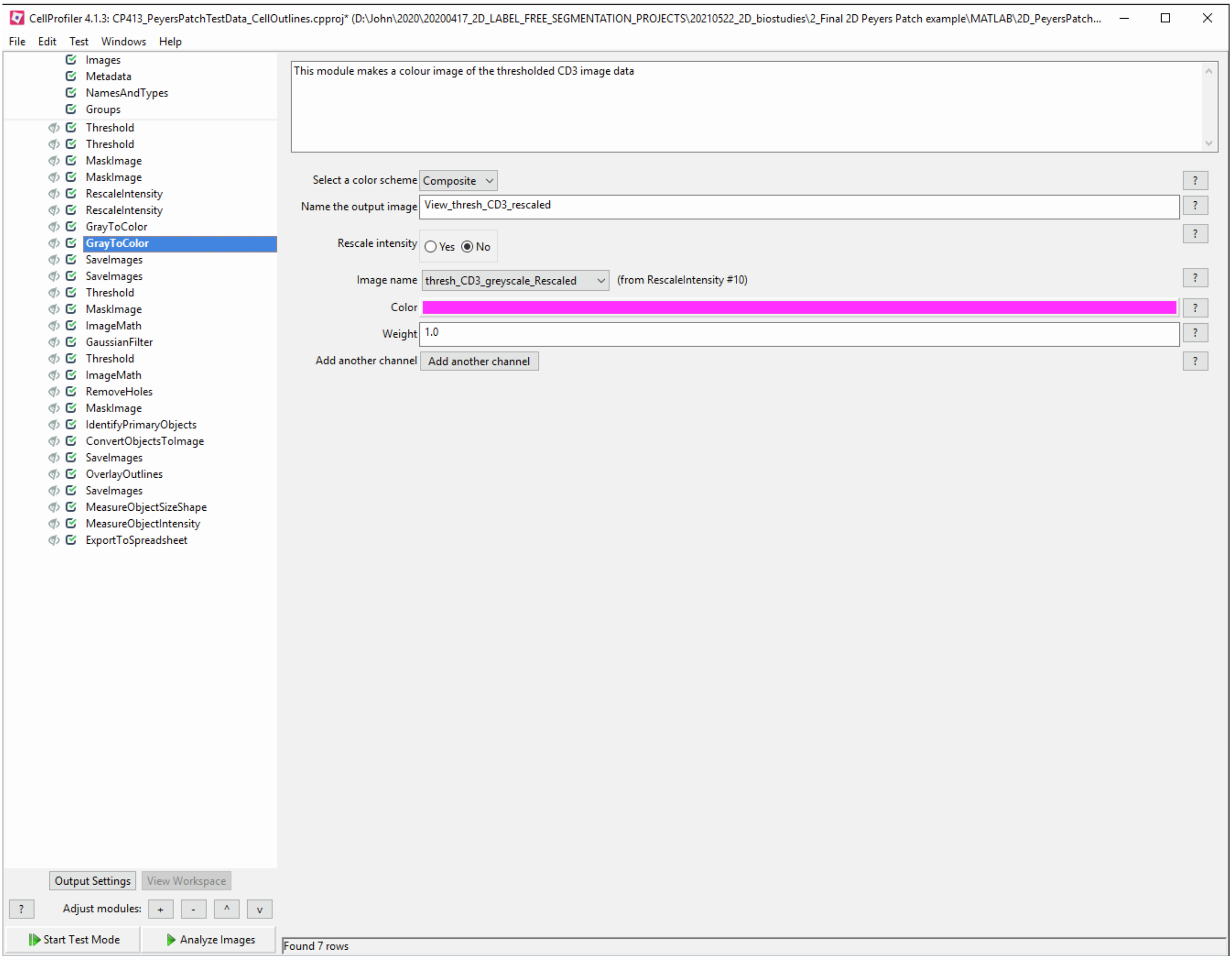
CellProfiler 4.1.3: CP413_PeyersPatchTestData_CellOutlines.cpproj* (D:\John\2020\20200417_2D_LABEL_FREE_SEGMENTATION_PROJECTS\20210522_2D_biostudies\2_Final 2D Peyers Patch example\MATLAB\2D_PeyersPatch...
File Edit Test Windows Help
Images
Metadata
NamesAndTypes
Groups
Threshold
Threshold
MaskImage
MaskImage
RescaleIntensity
RescaleIntensity
GrayToColor
GrayToColor
SaveImages
SaveImages
Threshold
MaskImage
ImageMath
GaussianFilter
Threshold
ImageMath
RemoveHoles
MaskImage
IdentifyPrimaryObjects
ConvertObjectsToImage
SaveImages
OverlayOutlines
SaveImages
MeasureObjectSizeShape
MeasureObjectIntensity
ExportToSpreadsheet
This module makes a colour image of the thresholded CD3 image data
Select a color scheme Composite
Name the output image View_thresh_CD3_rescaled
Rescale intensity Yes No
Image name thresh_CD3_greyscale_Rescaled (from RescaleIntensity #10)
Color
Weight 1.0
Add another channel Add another channel
Output Settings View Workspace
Adjust modules: + - ^ v
Start Test Mode
Analyze Images
Found 7 rows

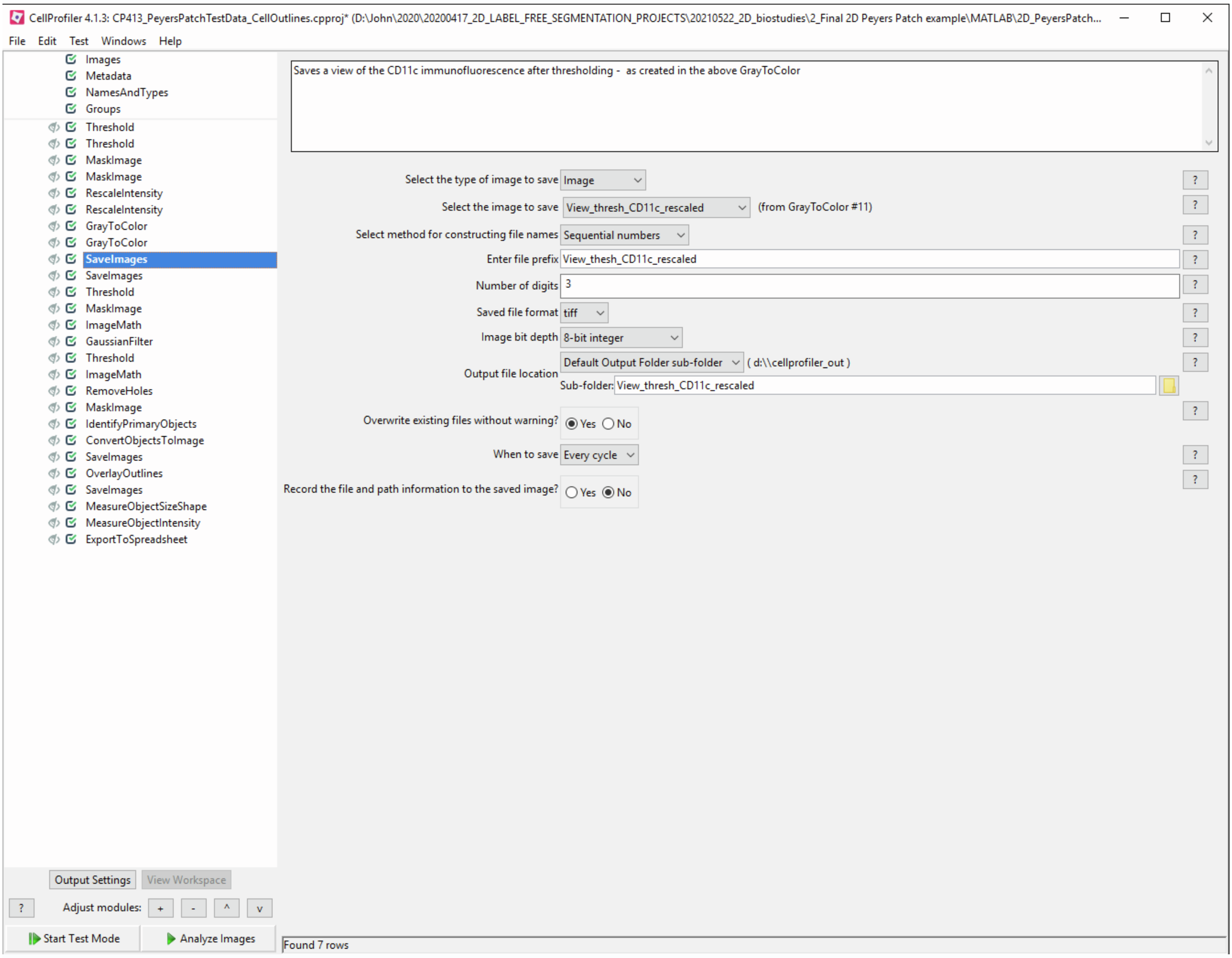
CellProfiler 4.1.3: CP413_PeyersPatchTestData_CellOutlines.cpproj* (D:\John\2020\20200417_2D_LABEL_FREE_SEGMENTATION_PROJECTS\20210522_2D_biostudies\2_Final 2D Peyers Patch example\MATLAB\2D_PeyersPatch...
File Edit Test Windows Help
Images
Metadata
NamesAndTypes
Groups
Threshold
Threshold
MaskImage
MaskImage
RescaleIntensity
RescaleIntensity
GrayToColor
GrayToColor
SaveImages
SaveImages
Threshold
MaskImage
ImageMath
GaussianFilter
Threshold
ImageMath
RemoveHoles
MaskImage
IdentifyPrimaryObjects
ConvertObjectsToImage
SaveImages
OverlayOutlines
SaveImages
MeasureObjectSizeShape
MeasureObjectIntensity
ExportToSpreadsheet
Saves a view of the CD11c immunofluorescence after thresholding - as created in the above GrayToColor
Select the type of image to save Image
Select the image to save View_thresh_CD11c_rescaled (from GrayToColor #11)
Select method for constructing file names Sequential numbers
Enter file prefix View_thesh_CD11c_rescaled
Number of digits 3
Saved file format tiff
Image bit depth 8-bit integer
Output file location Default Output Folder sub-folder (d:\\cellprofiler_out)
Sub-folder: View_thresh_CD11c_rescaled
Overwrite existing files without warning? Yes No
When to save Every cycle
Record the file and path information to the saved image? Yes No
Output Settings
View Workspace
Adjust modules: + - ^ v
Start Test Mode
Analyze Images
Found 7 rows

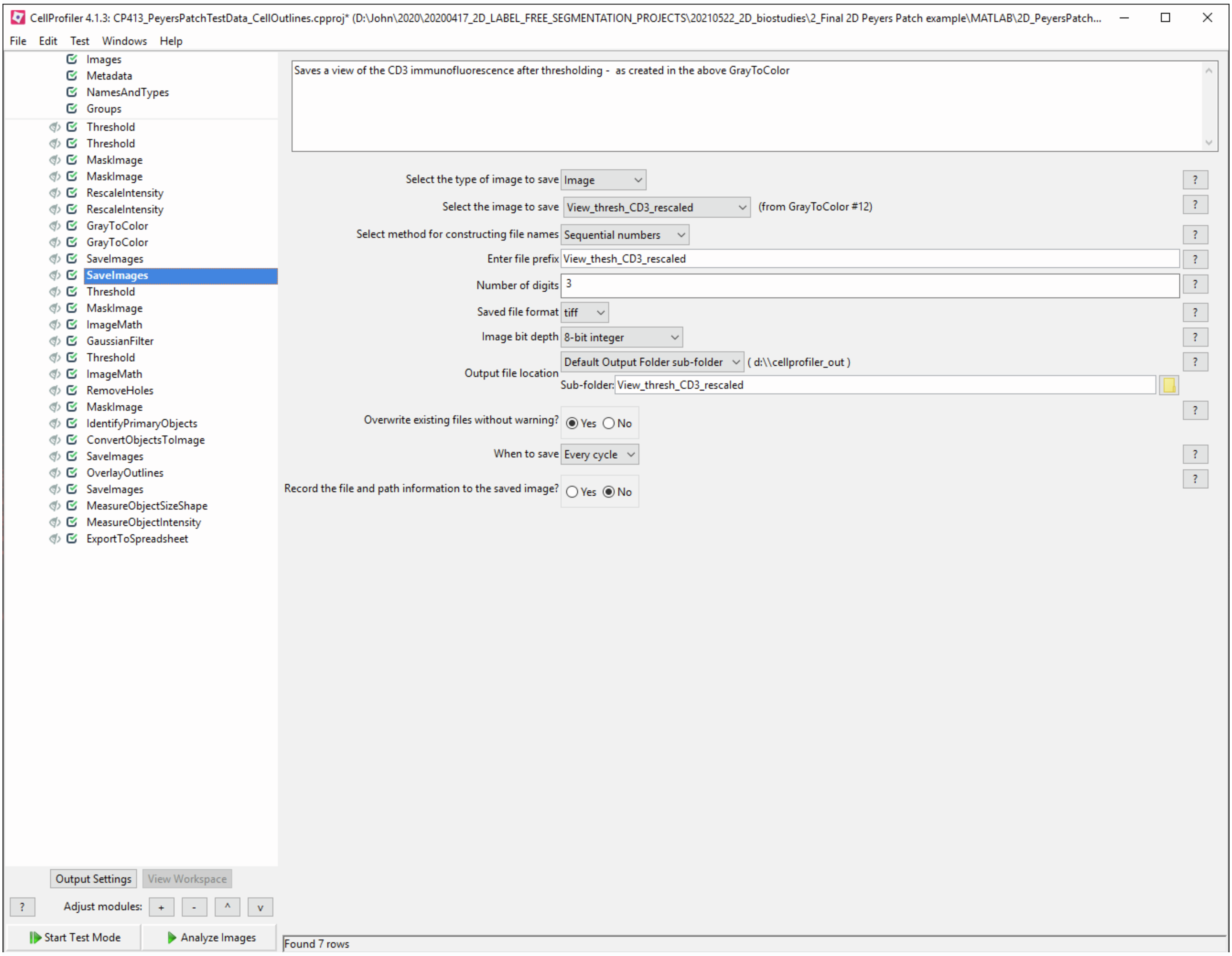
CellProfiler 4.1.3: CP413_PeyersPatchTestData_CellOutlines.cpproj* (D:\John\2020\20200417_2D_LABEL_FREE_SEGMENTATION_PROJECTS\20210522_2D_biostudies\2_Final 2D Peyers Patch example\MATLAB\2D_PeyersPatch...
File Edit Test Windows Help
Images
Metadata
NamesAndTypes
Groups
Threshold
Threshold
MaskImage
MaskImage
RescaleIntensity
RescaleIntensity
GrayToColor
GrayToColor
SaveImages
SaveImages
Threshold
MaskImage
ImageMath
GaussianFilter
Threshold
ImageMath
RemoveHoles
MaskImage
IdentifyPrimaryObjects
ConvertObjectsToImage
SaveImages
OverlayOutlines
SaveImages
MeasureObjectSizeShape
MeasureObjectIntensity
ExportToSpreadsheet
Saves a view of the CD3 immunofluorescence after thresholding - as created in the above GrayToColor
Select the type of image to save Image
Select the image to save View_thresh_CD3_rescaled (from GrayToColor #12)
Select method for constructing file names Sequential numbers
Enter file prefix View_thesh_CD3_rescaled
Number of digits 3
Saved file format tiff
Image bit depth 8-bit integer
Output file location Default Output Folder sub-folder (d:\\cellprofiler_out)
Sub-folder: View_thresh_CD3_rescaled
Overwrite existing files without warning? Yes No
When to save Every cycle
Record the file and path information to the saved image? Yes No
Output Settings View Workspace
Adjust modules: + - ^ v
Start Test Mode
Analyze Images
Found 7 rows

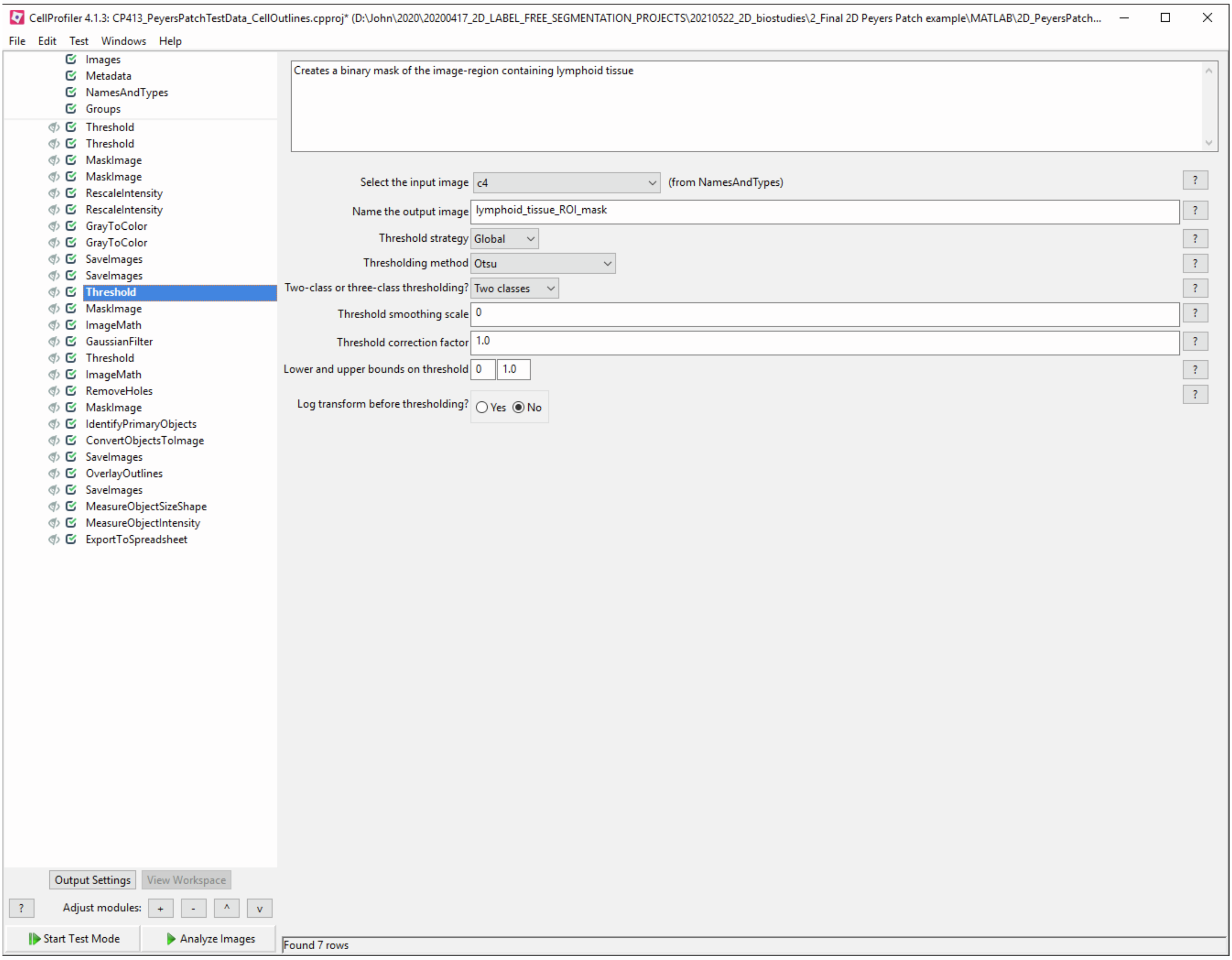
CellProfiler 4.1.3: CP413_PeyersPatchTestData_CellOutlines.cpproj* (D:\John\2020\20200417_2D_LABEL_FREE_SEGMENTATION_PROJECTS\20210522_2D_biostudies\2_Final 2D Peyers Patch example\MATLAB\2D_PeyersPatch...
File Edit Test Windows Help
Images
Metadata
NamesAndTypes
Groups
Threshold
Threshold
MaskImage
MaskImage
RescaleIntensity
RescaleIntensity
GrayToColor
GrayToColor
SaveImages
SaveImages
Threshold
MaskImage
ImageMath
GaussianFilter
Threshold
ImageMath
RemoveHoles
MaskImage
IdentifyPrimaryObjects
ConvertObjectsToImage
SaveImages
OverlayOutlines
SaveImages
MeasureObjectSizeShape
MeasureObjectIntensity
ExportToSpreadsheet
Creates a binary mask of the image-region containing lymphoid tissue
Select the input image c4 (from NamesAndTypes)
Name the output image lymphoid_tissue_ROI_mask
Threshold strategy Global
Thresholding method Otsu
Two-class or three-class thresholding? Two classes
Threshold smoothing scale 0
Threshold correction factor 1.0
Lower and upper bounds on threshold 0 1.0
Log transform before thresholding? Yes No
Output Settings
View Workspace
Adjust modules: + - ^ v
Start Test Mode
Analyze Images
Found 7 rows

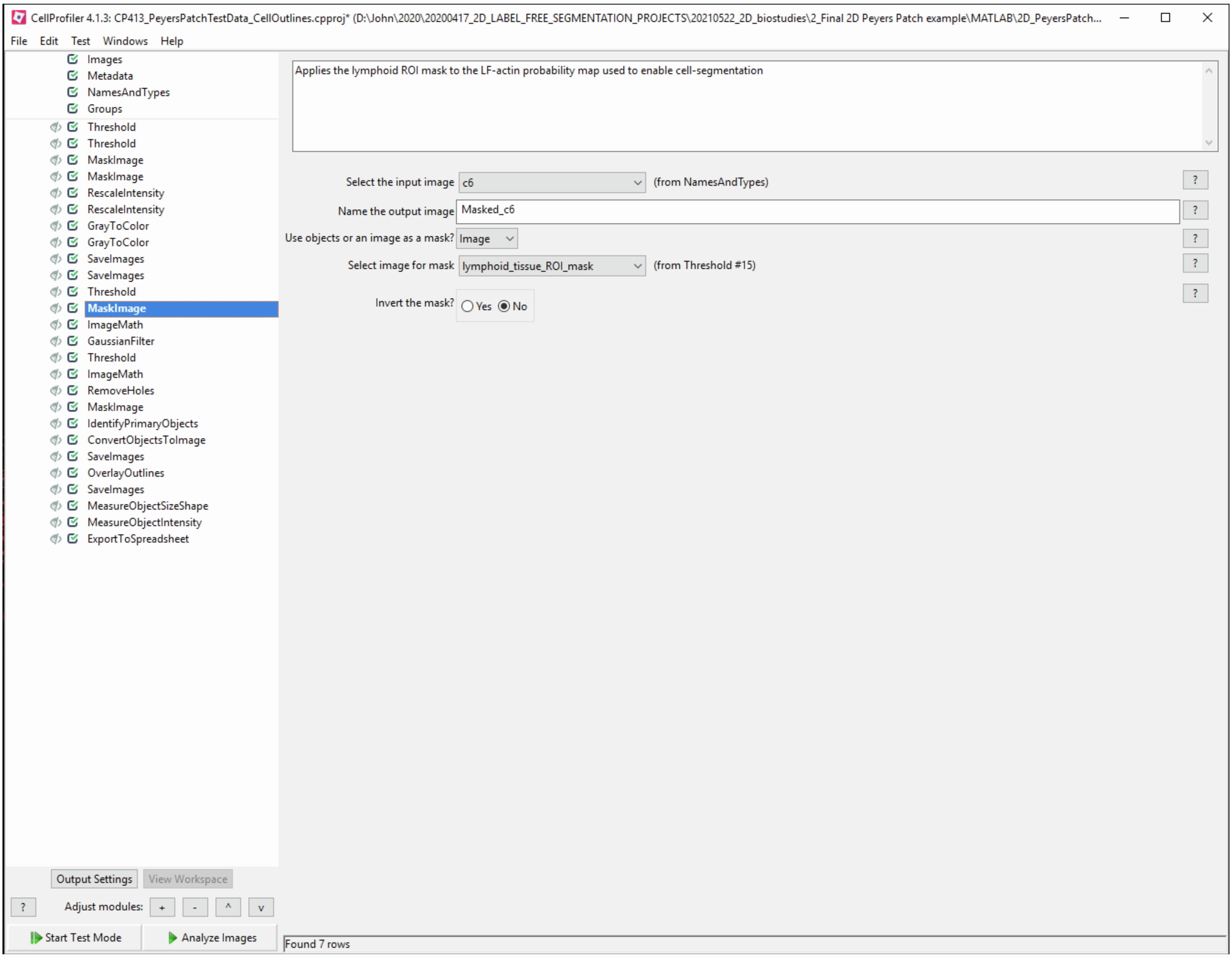
CellProfiler 4.1.3: CP413_PeyersPatchTestData_CellOutlines.cpproj* (D:\John\2020\20200417_2D_LABEL_FREE_SEGMENTATION_PROJECTS\20210522_2D_biostudies\2_Final 2D Peyers Patch example\MATLAB\2D_PeyersPatch...
File Edit Test Windows Help
Images
Metadata
NamesAndTypes
Groups
Threshold
Threshold
MaskImage
MaskImage
RescaleIntensity
RescaleIntensity
GrayToColor
GrayToColor
SaveImages
SaveImages
Threshold
MaskImage
ImageMath
GaussianFilter
Threshold
ImageMath
RemoveHoles
MaskImage
IdentifyPrimaryObjects
ConvertObjectsToImage
SaveImages
OverlayOutlines
SaveImages
MeasureObjectSizeShape
MeasureObjectIntensity
ExportToSpreadsheet
Applies the lymphoid ROI mask to the LF-actin probability map used to enable cell-segmentation
Select the input image c6 (from NamesAndTypes)
Name the output image Masked_c6
Use objects or an image as a mask? Image
Select image for mask lymphoid_tissue_ROI_mask (from Threshold #15)
Invert the mask? Yes No
Output Settings
View Workspace
Adjust modules: + - ^ v
Start Test Mode
Analyze Images
Found 7 rows

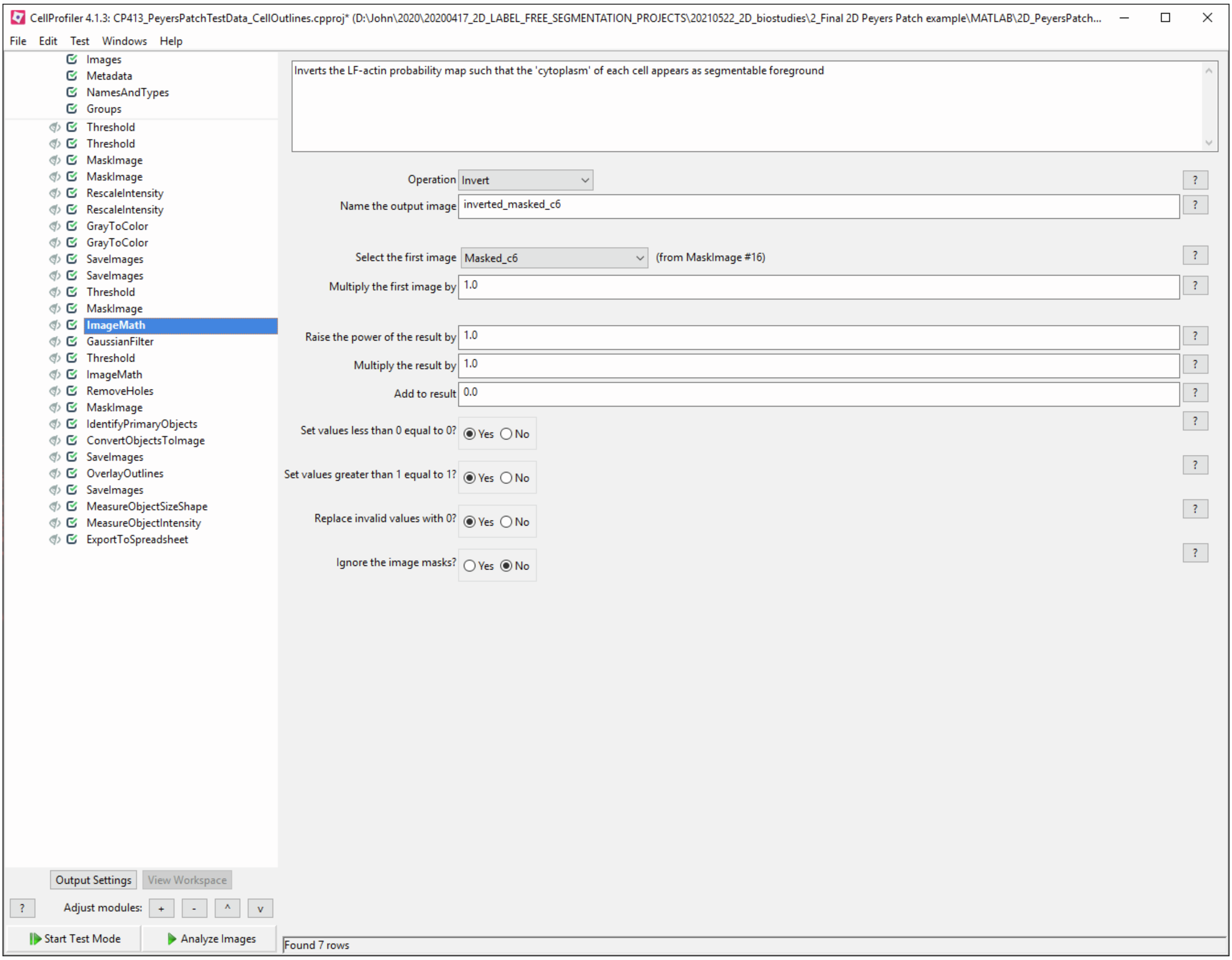
CellProfiler 4.1.3: CP413_PeyersPatchTestData_CellOutlines.cpproj* (D:\John\2020\20200417_2D_LABEL_FREE_SEGMENTATION_PROJECTS\20210522_2D_biostudies\2_Final 2D Peyers Patch example\MATLAB\2D_PeyersPatch...
File
Edit
Test
Windows
Help
Images
Metadata
NamesAndTypes
Groups
Threshold
Threshold
MaskImage
MaskImage
RescaleIntensity
RescaleIntensity
GrayToColor
GrayToColor
SaveImages
SaveImages
Threshold
MaskImage
ImageMath
GaussianFilter
Threshold
ImageMath
RemoveHoles
MaskImage
IdentifyPrimaryObjects
ConvertObjectsToImage
SaveImages
OverlayOutlines
SaveImages
MeasureObjectSizeShape
MeasureObjectIntensity
ExportToSpreadsheet
Inverts the LF-actin probability map such that the 'cytoplasm' of each cell appears as segmentable foreground
Operation
Invert
Name the output image
inverted_masked_c6
Select the first image
Masked_c6
(from MaskImage #16)
Multiply the first image by
1.0
Raise the power of the result by
1.0
Multiply the result by
1.0
Add to result
0.0
Set values less than 0 equal to 0?
Yes
No
Set values greater than 1 equal to 1?
Yes
No
Replace invalid values with 0?
Yes
No
Ignore the image masks?
Yes
No
Output Settings
View Workspace
Adjust modules:
Start Test Mode
Analyze Images
Found 7 rows

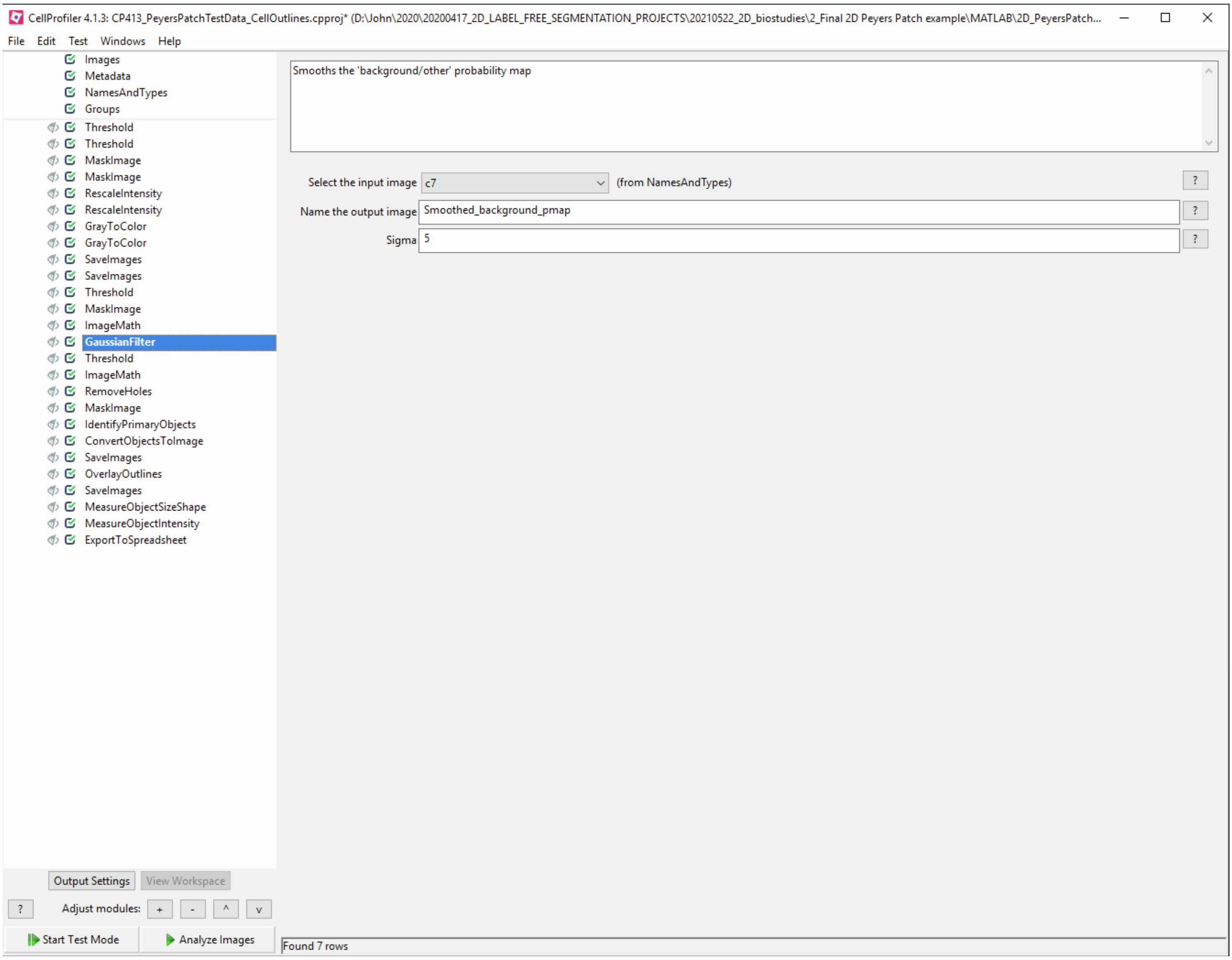
CellProfiler 4.1.3: CP413_PeyersPatchTestData_CellOutlines.cpproj* (D:\John\2020\20200417_2D_LABEL_FREE_SEGMENTATION_PROJECTS\20210522_2D_biostudies\2_Final 2D Peyers Patch example\MATLAB\2D_PeyersPatch...
File Edit Test Windows Help
Images
Metadata
NamesAndTypes
Groups
Threshold
Threshold
MaskImage
MaskImage
RescaleIntensity
RescaleIntensity
GrayToColor
GrayToColor
SaveImages
SaveImages
Threshold
MaskImage
ImageMath
GaussianFilter
Threshold
ImageMath
RemoveHoles
MaskImage
IdentifyPrimaryObjects
ConvertObjectsToImage
SaveImages
OverlayOutlines
SaveImages
MeasureObjectSizeShape
MeasureObjectIntensity
ExportToSpreadsheet
Smooths the 'background/other' probability map
Select the input image
c7
(from NamesAndTypes)
Name the output image
Smoothed_background_pmap
Sigma
5
Output Settings
View Workspace
Adjust modules:
Start Test Mode
Analyze Images
Found 7 rows

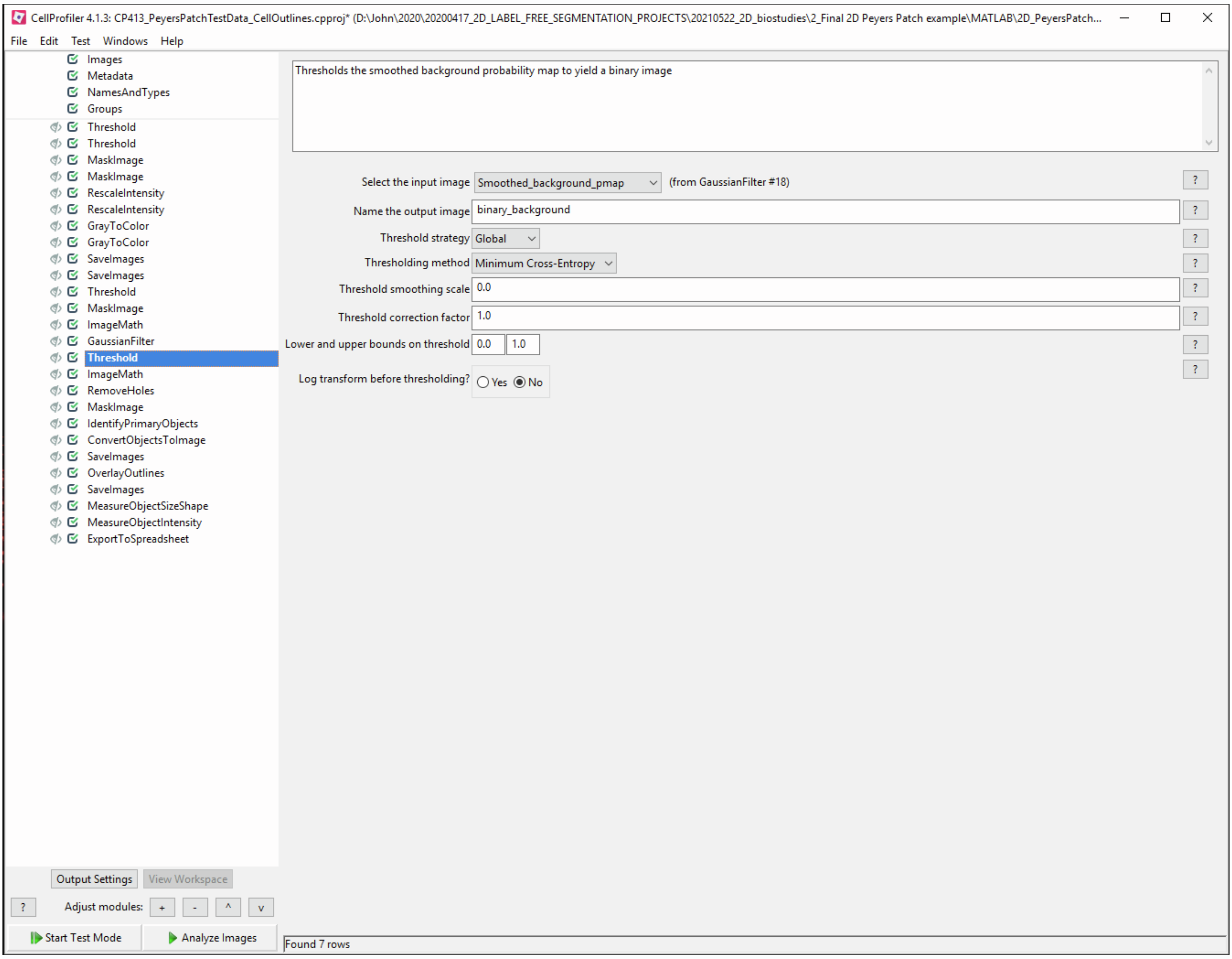
CellProfiler 4.1.3: CP413_PeyersPatchTestData_CellOutlines.cpproj* (D:\John\2020\20200417_2D_LABEL_FREE_SEGMENTATION_PROJECTS\20210522_2D_biostudies\2_Final 2D Peyers Patch example\MATLAB\2D_PeyersPatch...
File Edit Test Windows Help
Images
Metadata
NamesAndTypes
Groups
Threshold
Threshold
MaskImage
MaskImage
RescaleIntensity
RescaleIntensity
GrayToColor
GrayToColor
SaveImages
SaveImages
Threshold
MaskImage
ImageMath
GaussianFilter
Threshold
ImageMath
RemoveHoles
MaskImage
IdentifyPrimaryObjects
ConvertObjectsToImage
SaveImages
OverlayOutlines
SaveImages
MeasureObjectSizeShape
MeasureObjectIntensity
ExportToSpreadsheet
Thresholds the smoothed background probability map to yield a binary image
Select the input image
Smoothed_background_pmap
(from GaussianFilter #18)
Name the output image
binary_background
Threshold strategy
Global
Thresholding method
Minimum Cross-Entropy
Threshold smoothing scale
0.0
Threshold correction factor
1.0
Lower and upper bounds on threshold
0.0
1.0
Log transform before thresholding?
Yes
No
Output Settings
View Workspace
Adjust modules:
Start Test Mode
Analyze Images
Found 7 rows

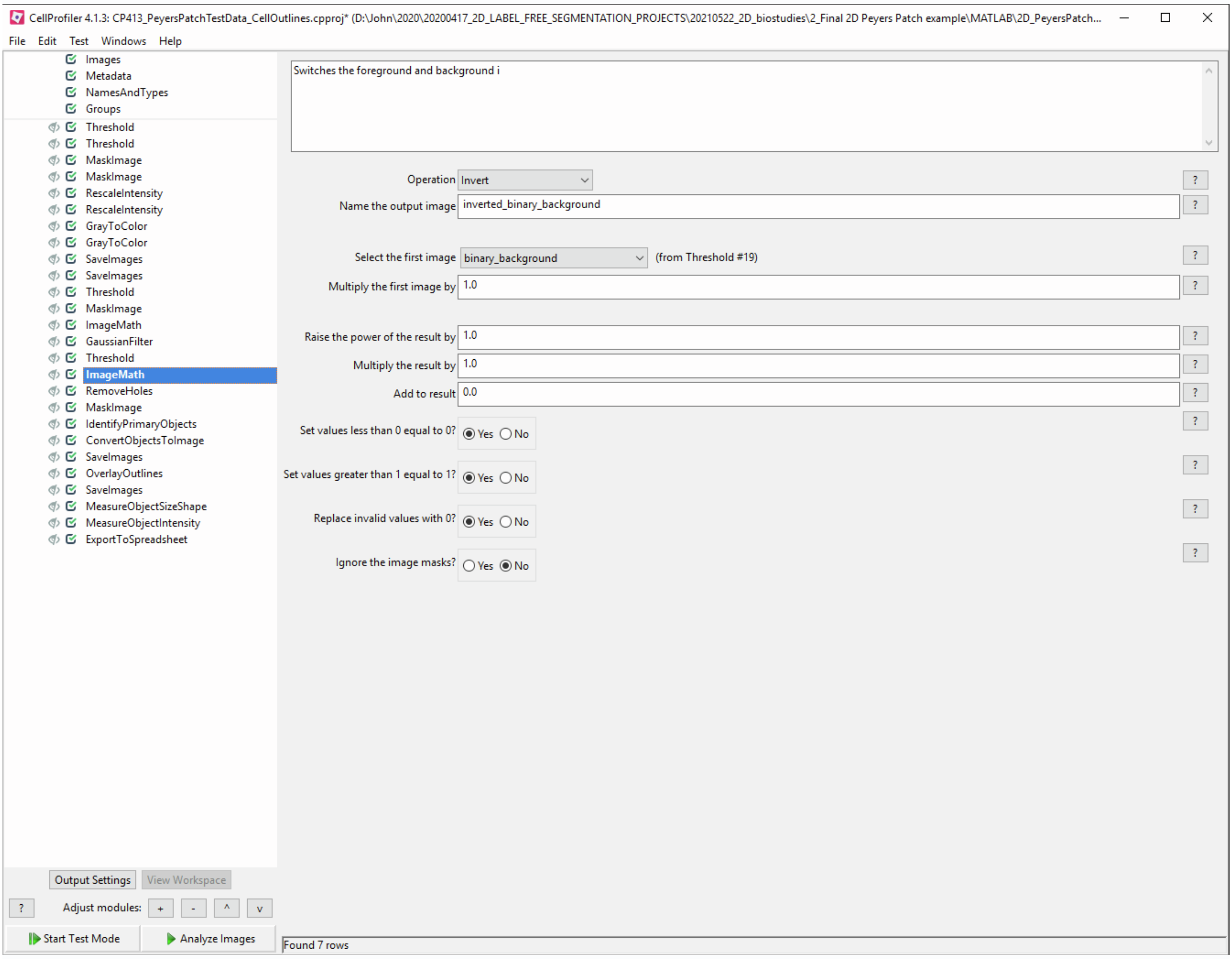
CellProfiler 4.1.3: CP413_PeyersPatchTestData_CellOutlines.cpproj* (D:\John\2020\20200417_2D_LABEL_FREE_SEGMENTATION_PROJECTS\20210522_2D_biostudies\2_Final 2D Peyers Patch example\MATLAB\2D_PeyersPatch...
File Edit Test Windows Help
Images
Metadata
NamesAndTypes
Groups
Threshold
Threshold
MaskImage
MaskImage
RescaleIntensity
RescaleIntensity
GrayToColor
GrayToColor
SaveImages
SaveImages
Threshold
MaskImage
ImageMath
GaussianFilter
Threshold
ImageMath
RemoveHoles
MaskImage
IdentifyPrimaryObjects
ConvertObjectsToImage
SaveImages
OverlayOutlines
SaveImages
MeasureObjectSizeShape
MeasureObjectIntensity
ExportToSpreadsheet
Switches the foreground and background i
Operation Invert
Name the output image inverted_binary_background
Select the first image binary_background (from Threshold #19)
Multiply the first image by 1.0
Raise the power of the result by 1.0
Multiply the result by 1.0
Add to result 0.0
Set values less than 0 equal to 0? Yes No
Set values greater than 1 equal to 1? Yes No
Replace invalid values with 0? Yes No
Ignore the image masks? Yes No
Output Settings
View Workspace
Adjust modules: + - ^ v
Start Test Mode
Analyze Images
Found 7 rows

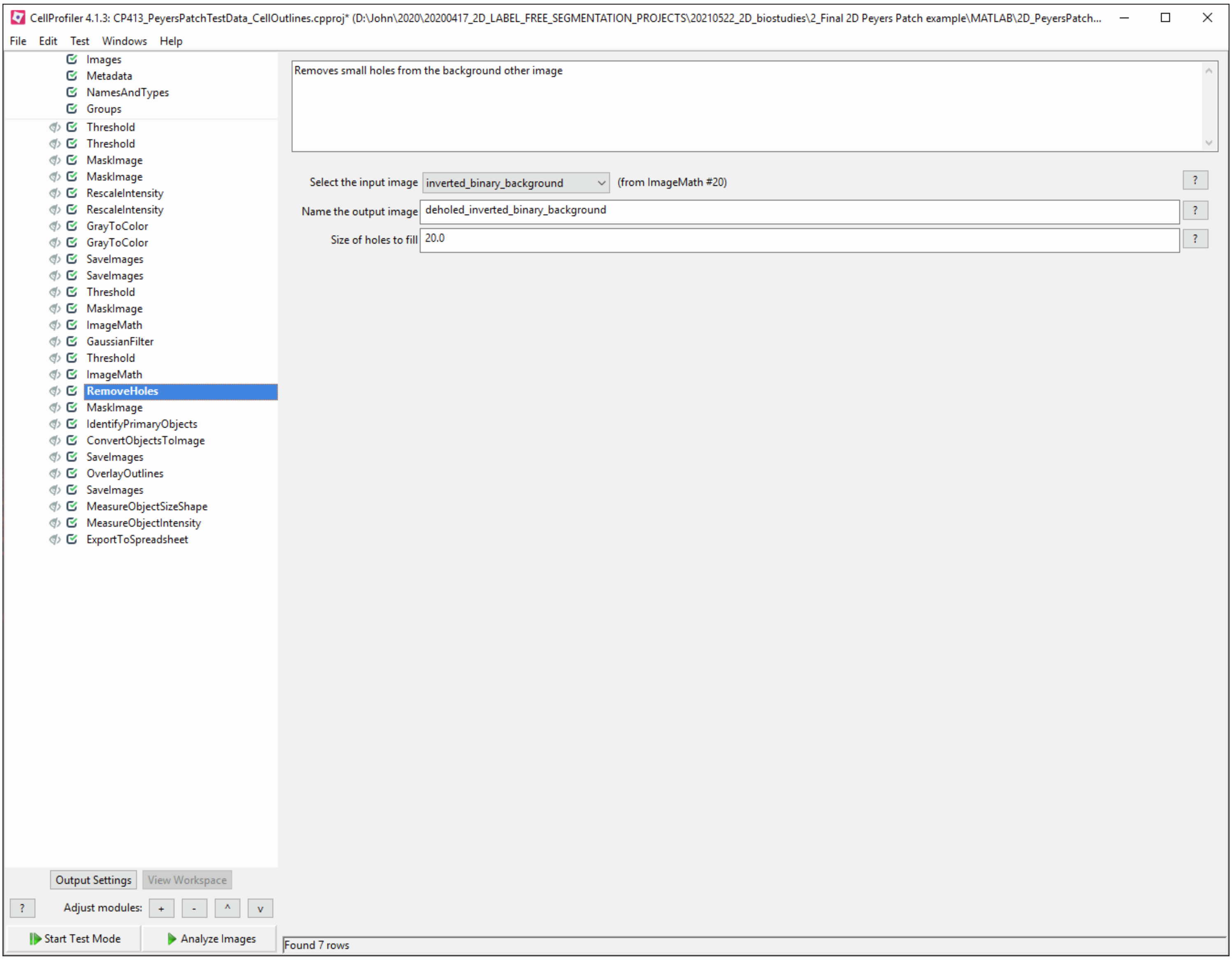
CellProfiler 4.1.3: CP413_PeyersPatchTestData_CellOutlines.cpproj* (D:\John\2020\20200417_2D_LABEL_FREE_SEGMENTATION_PROJECTS\20210522_2D_biostudies\2_Final 2D Peyers Patch example\MATLAB\2D_PeyersPatch...
File Edit Test Windows Help
Images
Metadata
NamesAndTypes
Groups
Threshold
Threshold
MaskImage
MaskImage
RescaleIntensity
RescaleIntensity
GrayToColor
GrayToColor
SaveImages
SaveImages
Threshold
MaskImage
ImageMath
GaussianFilter
Threshold
ImageMath
RemoveHoles
MaskImage
IdentifyPrimaryObjects
ConvertObjectsToImage
SaveImages
OverlayOutlines
SaveImages
MeasureObjectSizeShape
MeasureObjectIntensity
ExportToSpreadsheet
Removes small holes from the background other image
Select the input image
inverted_binary_background
(from ImageMath #20)
Name the output image
deholed_inverted_binary_background
Size of holes to fill
20.0
Output Settings
View Workspace
Adjust modules:
Start Test Mode
Analyze Images
Found 7 rows

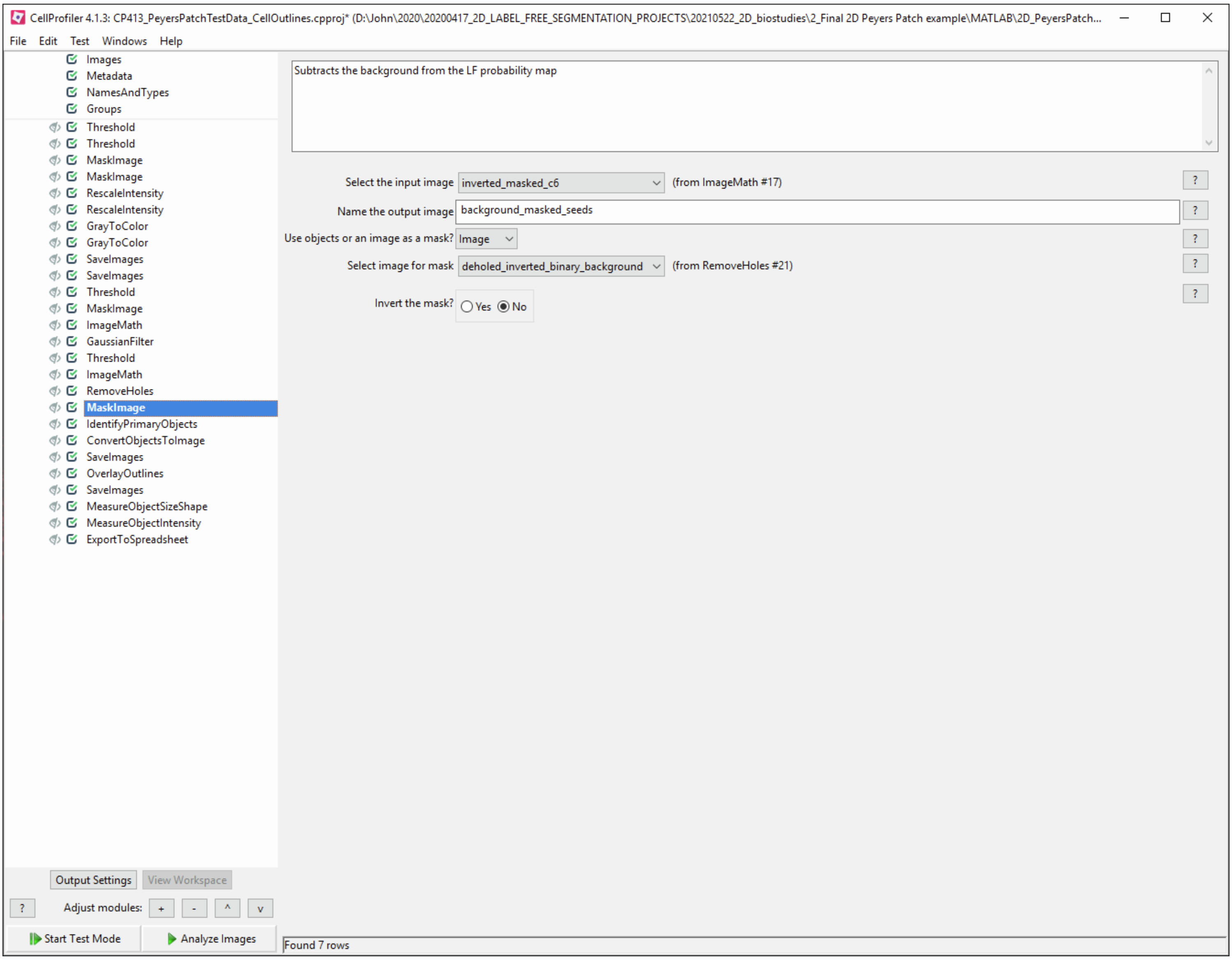
CellProfiler 4.1.3: CP413_PeyersPatchTestData_CellOutlines.cpproj* (D:\John\2020\20200417_2D_LABEL_FREE_SEGMENTATION_PROJECTS\20210522_2D_biostudies\2_Final 2D Peyers Patch example\MATLAB\2D_PeyersPatch...
File Edit Test Windows Help
Images
Metadata
NamesAndTypes
Groups
Threshold
Threshold
MaskImage
MaskImage
RescaleIntensity
RescaleIntensity
GrayToColor
GrayToColor
SaveImages
SaveImages
Threshold
MaskImage
ImageMath
GaussianFilter
Threshold
ImageMath
RemoveHoles
MaskImage
IdentifyPrimaryObjects
ConvertObjectsToImage
SaveImages
OverlayOutlines
SaveImages
MeasureObjectSizeShape
MeasureObjectIntensity
ExportToSpreadsheet
Subtracts the background from the LF probability map
Select the input image
inverted_masked_c6
(from ImageMath #17)
Name the output image
background_masked_seeds
Use objects or an image as a mask?
Image
Select image for mask
deholed_inverted_binary_background
(from RemoveHoles #21)
Invert the mask?
Yes
No
Output Settings
View Workspace
Adjust modules:
Start Test Mode
Analyze Images
Found 7 rows

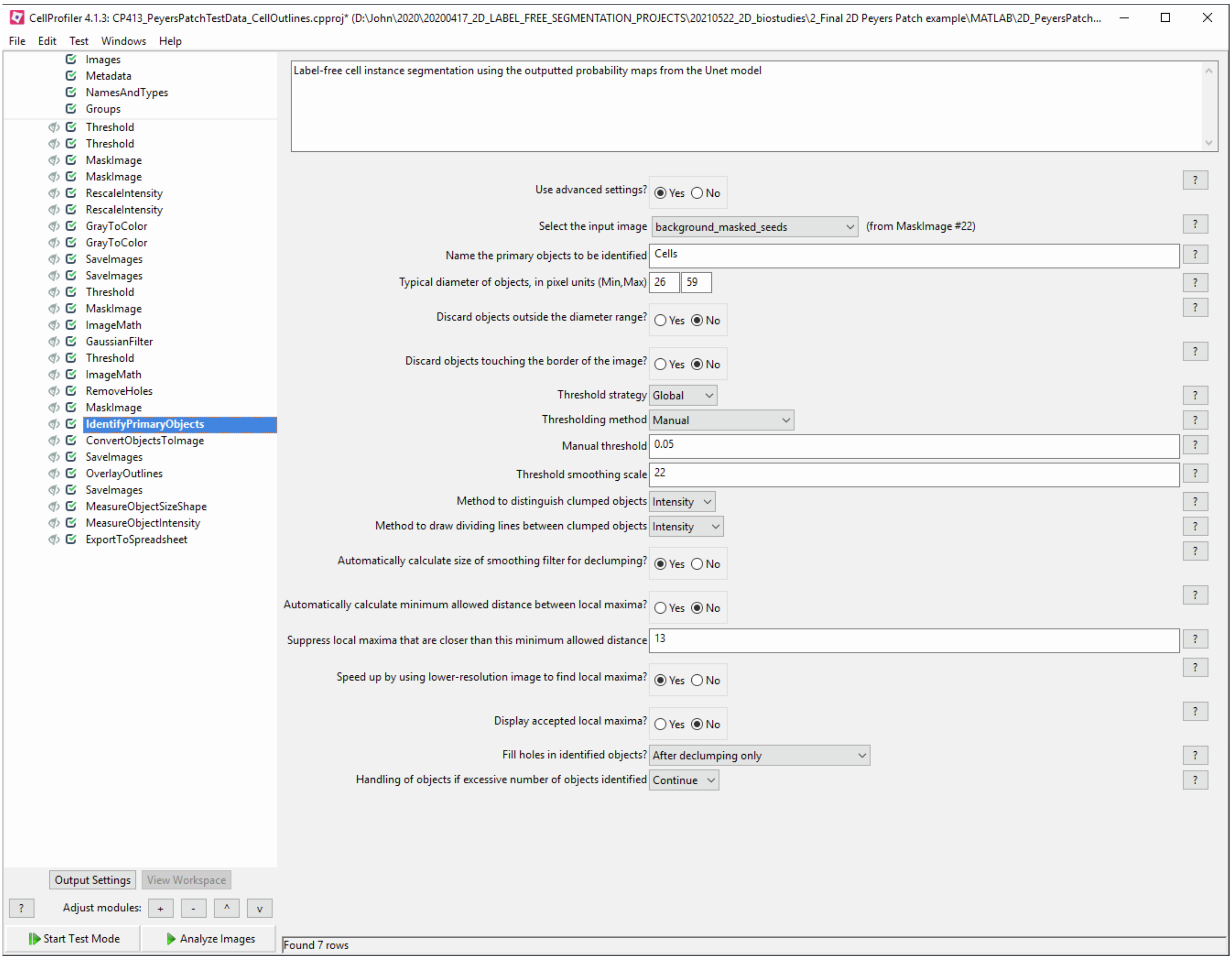
CellProfiler 4.1.3: CP413_PeyersPatchTestData_CellOutlines.cpproj* (D:\John\2020\20200417_2D_LABEL_FREE_SEGMENTATION_PROJECTS\20210522_2D_biostudies\2_Final 2D Peyers Patch example\MATLAB\2D_PeyersPatch...
File Edit Test Windows Help
Images
Metadata
NamesAndTypes
Groups
Threshold
Threshold
MaskImage
MaskImage
RescaleIntensity
RescaleIntensity
GrayToColor
GrayToColor
SaveImages
SaveImages
Threshold
MaskImage
ImageMath
GaussianFilter
Threshold
ImageMath
RemoveHoles
MaskImage
IdentifyPrimaryObjects
ConvertObjectsToImage
SaveImages
OverlayOutlines
SaveImages
MeasureObjectSizeShape
MeasureObjectIntensity
ExportToSpreadsheet
Label-free cell instance segmentation using the outputted probability maps from the Unet model
Use advanced settings? Yes No
Select the input image background_masked_seeds (from MaskImage #22)
Name the primary objects to be identified Cells
Typical diameter of objects, in pixel units (Min,Max) 26 59
Discard objects outside the diameter range? Yes No
Discard objects touching the border of the image? Yes No
Threshold strategy Global
Thresholding method Manual
Manual threshold 0.05
Threshold smoothing scale 22
Method to distinguish clumped objects Intensity
Method to draw dividing lines between clumped objects Intensity
Automatically calculate size of smoothing filter for declumping? Yes No
Automatically calculate minimum allowed distance between local maxima? Yes No
Suppress local maxima that are closer than this minimum allowed distance 13
Speed up by using lower-resolution image to find local maxima? Yes No
Display accepted local maxima? Yes No
Fill holes in identified objects? After declumping only
Handling of objects if excessive number of objects identified Continue
Output Settings
View Workspace
Adjust modules: + - ^ v
Start Test Mode
Analyze Images
Found 7 rows

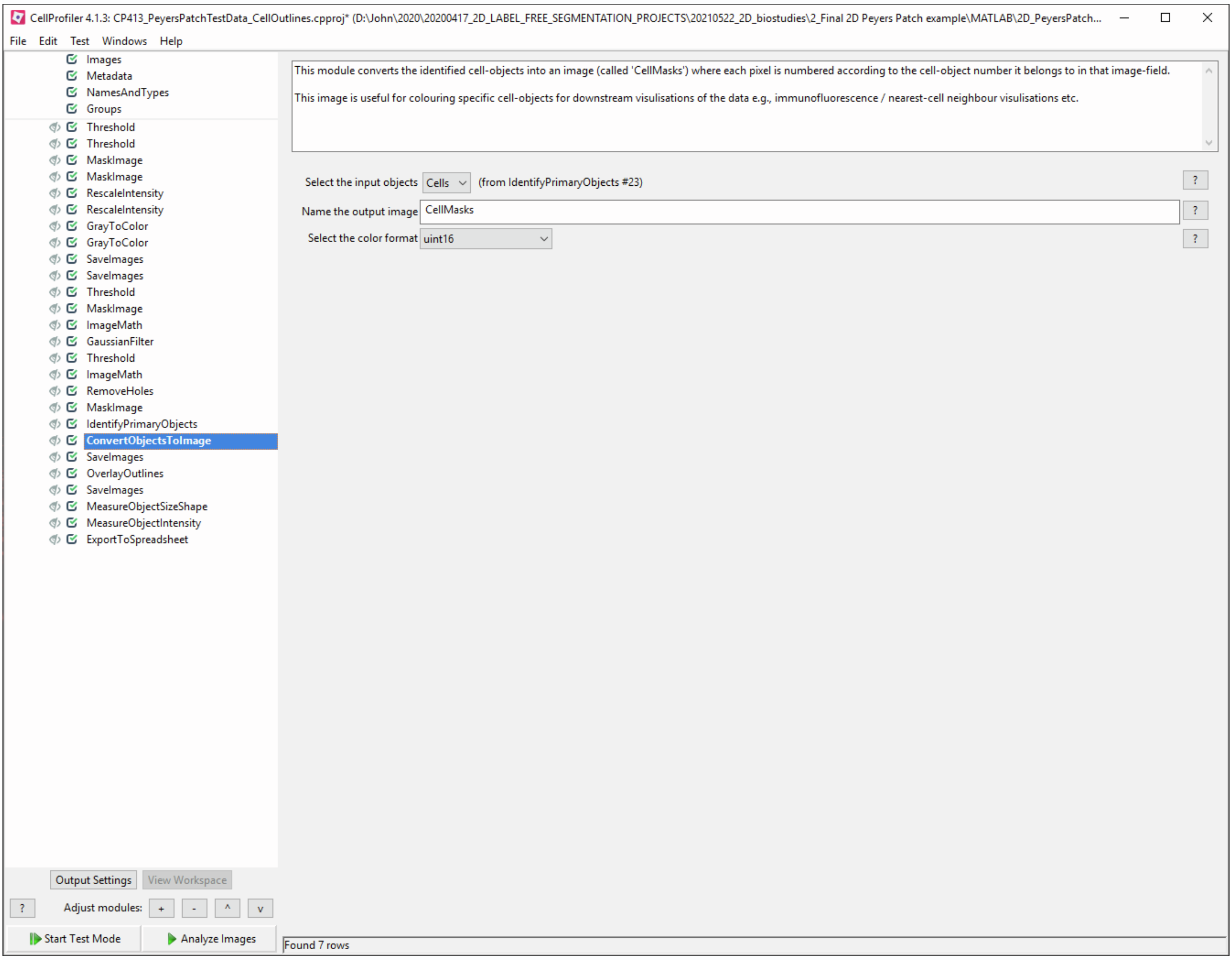
CellProfiler 4.1.3: CP413_PeyersPatchTestData_CellOutlines.cpproj* (D:\John\2020\20200417_2D_LABEL_FREE_SEGMENTATION_PROJECTS\20210522_2D_biostudies\2_Final 2D Peyers Patch example\MATLAB\2D_PeyersPatch...
File Edit Test Windows Help
Images
Metadata
NamesAndTypes
Groups
Threshold
Threshold
MaskImage
MaskImage
RescaleIntensity
RescaleIntensity
GrayToColor
GrayToColor
SaveImages
SaveImages
Threshold
MaskImage
ImageMath
GaussianFilter
Threshold
ImageMath
RemoveHoles
MaskImage
IdentifyPrimaryObjects
ConvertObjectsToImage
SaveImages
OverlayOutlines
SaveImages
MeasureObjectSizeShape
MeasureObjectIntensity
ExportToSpreadsheet
This module converts the identified cell-objects into an image (called 'CellMasks') where each pixel is numbered according to the cell-object number it belongs to in that image-field.
This image is useful for colouring specific cell-objects for downstream visulisations of the data e.g., immunofluorescence / nearest-cell neighbour visulisations etc.
Select the input objects
Cells
(from IdentifyPrimaryObjects #23)
Name the output image
CellMasks
Select the color format
uint16
Output Settings
View Workspace
Adjust modules:
Start Test Mode
Analyze Images
Found 7 rows

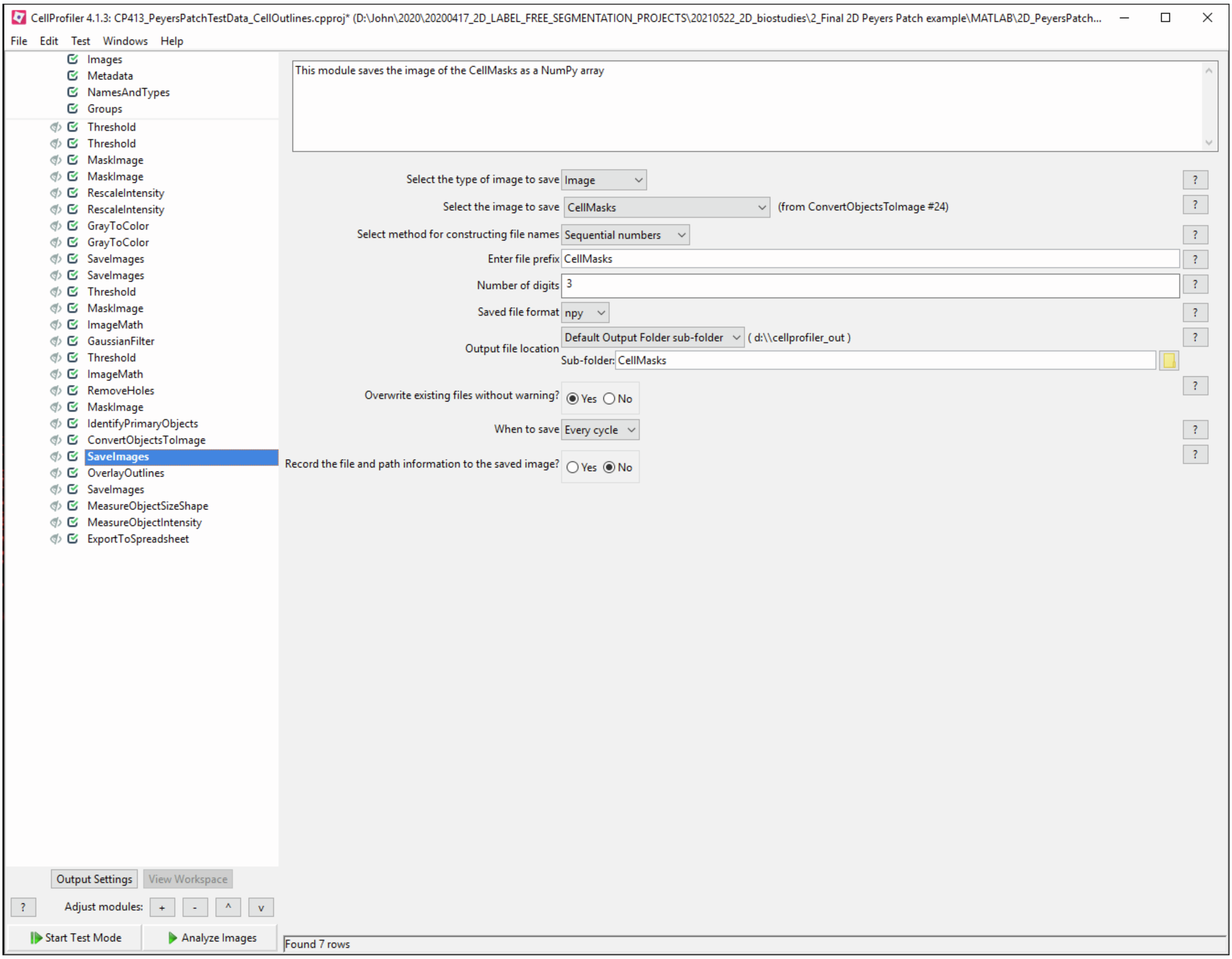
CellProfiler 4.1.3: CP413_PeyersPatchTestData_CellOutlines.cpproj* (D:\John\2020\20200417_2D_LABEL_FREE_SEGMENTATION_PROJECTS\20210522_2D_biostudies\2_Final 2D Peyers Patch example\MATLAB\2D_PeyersPatch...
File Edit Test Windows Help
Images
Metadata
NamesAndTypes
Groups
Threshold
Threshold
MaskImage
MaskImage
RescaleIntensity
RescaleIntensity
GrayToColor
GrayToColor
SaveImages
SaveImages
Threshold
MaskImage
ImageMath
GaussianFilter
Threshold
ImageMath
RemoveHoles
MaskImage
IdentifyPrimaryObjects
ConvertObjectsToImage
SaveImages
OverlayOutlines
SaveImages
MeasureObjectSizeShape
MeasureObjectIntensity
ExportToSpreadsheet
This module saves the image of the CellMasks as a NumPy array
Select the type of image to save Image
Select the image to save CellMasks (from ConvertObjectsToImage #24)
Select method for constructing file names Sequential numbers
Enter file prefix CellMasks
Number of digits 3
Saved file format npy
Output file location Default Output Folder sub-folder (d:\\cellprofiler_out)
Sub-folder: CellMasks
Overwrite existing files without warning? Yes No
When to save Every cycle
Record the file and path information to the saved image? Yes No
Output Settings
View Workspace
Adjust modules: + - ^ v
Start Test Mode
Analyze Images
Found 7 rows

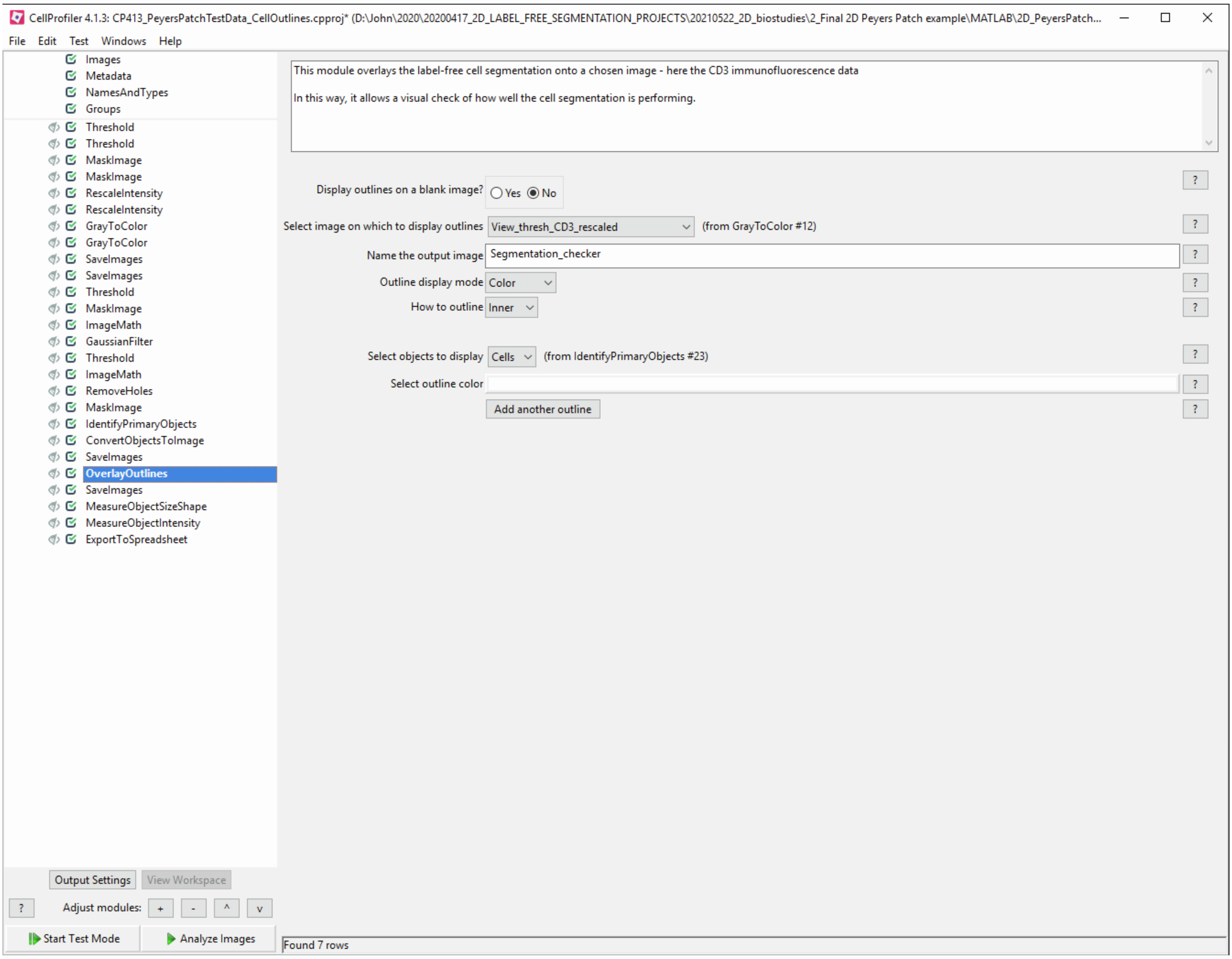
CellProfiler 4.1.3: CP413_PeyersPatchTestData_CellOutlines.cpproj* (D:\John\2020\20200417_2D_LABEL_FREE_SEGMENTATION_PROJECTS\20210522_2D_biostudies\2_Final 2D Peyers Patch example\MATLAB\2D_PeyersPatch...
File Edit Test Windows Help
Images
Metadata
NamesAndTypes
Groups
Threshold
Threshold
MaskImage
MaskImage
RescaleIntensity
RescaleIntensity
GrayToColor
GrayToColor
SaveImages
SaveImages
Threshold
MaskImage
ImageMath
GaussianFilter
Threshold
ImageMath
RemoveHoles
MaskImage
IdentifyPrimaryObjects
ConvertObjectsToImage
SaveImages
OverlayOutlines
SaveImages
MeasureObjectSizeShape
MeasureObjectIntensity
ExportToSpreadsheet
This module overlays the label-free cell segmentation onto a chosen image - here the CD3 immunofluorescence data
In this way, it allows a visual check of how well the cell segmentation is performing.
Display outlines on a blank image? Yes No
Select image on which to display outlines View_thresh_CD3_rescaled (from GrayToColor #12)
Name the output image Segmentation_checker
Outline display mode Color
How to outline Inner
Select objects to display Cells (from IdentifyPrimaryObjects #23)
Select outline color
Add another outline
Output Settings
View Workspace
Adjust modules: + - ^ v
Start Test Mode
Analyze Images
Found 7 rows

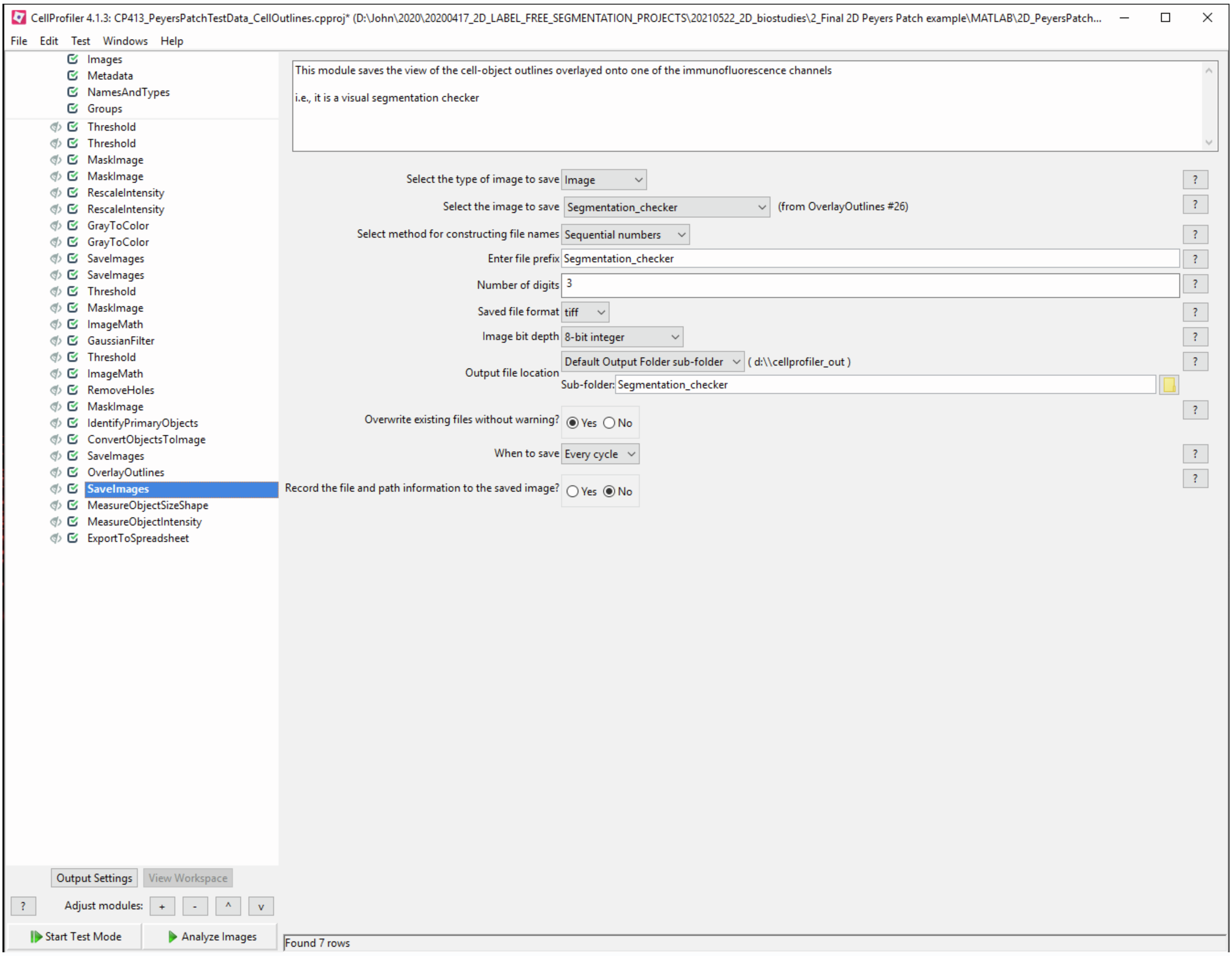
CellProfiler 4.1.3: CP413_PeyersPatchTestData_CellOutlines.cpproj* (D:\John\2020\20200417_2D_LABEL_FREE_SEGMENTATION_PROJECTS\20210522_2D_biostudies\2_Final 2D Peyers Patch example\MATLAB\2D_PeyersPatch...
File Edit Test Windows Help
Images
Metadata
NamesAndTypes
Groups
Threshold
Threshold
MaskImage
MaskImage
RescaleIntensity
RescaleIntensity
GrayToColor
GrayToColor
SaveImages
SaveImages
Threshold
MaskImage
ImageMath
GaussianFilter
Threshold
ImageMath
RemoveHoles
MaskImage
IdentifyPrimaryObjects
ConvertObjectsToImage
SaveImages
OverlayOutlines
SaveImages
MeasureObjectSizeShape
MeasureObjectIntensity
ExportToSpreadsheet
This module saves the view of the cell-object outlines overlayed onto one of the immunofluorescence channels
i.e., it is a visual segmentation checker
Select the type of image to save Image
Select the image to save Segmentation_checker (from OverlayOutlines #26)
Select method for constructing file names Sequential numbers
Enter file prefix Segmentation_checker
Number of digits 3
Saved file format tiff
Image bit depth 8-bit integer
Output file location Default Output Folder sub-folder (d:\\cellprofiler_out)
Sub-folder: Segmentation_checker
Overwrite existing files without warning? Yes No
When to save Every cycle
Record the file and path information to the saved image? Yes No
Output Settings
View Workspace
Adjust modules: + - ^ v
Start Test Mode
Analyze Images
Found 7 rows

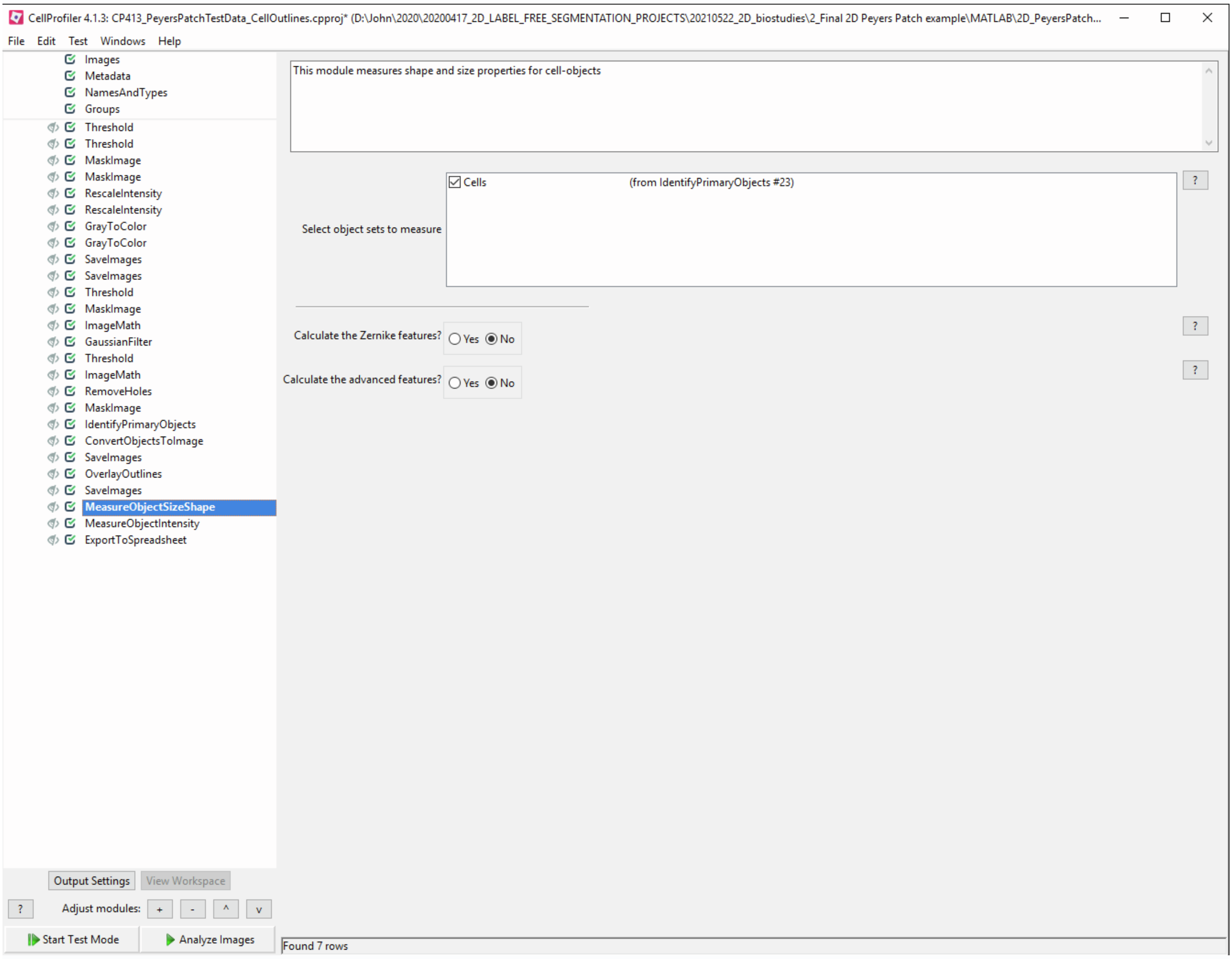
CellProfiler 4.1.3: CP413_PeyersPatchTestData_CellOutlines.cpproj* (D:\John\2020\20200417_2D_LABEL_FREE_SEGMENTATION_PROJECTS\20210522_2D_biostudies\2_Final 2D Peyers Patch example\MATLAB\2D_PeyersPatch...
File Edit Test Windows Help
Images
Metadata
NamesAndTypes
Groups
Threshold
Threshold
MaskImage
MaskImage
RescaleIntensity
RescaleIntensity
GrayToColor
GrayToColor
SaveImages
SaveImages
Threshold
MaskImage
ImageMath
GaussianFilter
Threshold
ImageMath
RemoveHoles
MaskImage
IdentifyPrimaryObjects
ConvertObjectsToImage
SaveImages
OverlayOutlines
SaveImages
MeasureObjectSizeShape
MeasureObjectIntensity
ExportToSpreadsheet
This module measures shape and size properties for cell-objects
Select object sets to measure
Cells (from IdentifyPrimaryObjects #23)
Calculate the Zernike features? Yes No
Calculate the advanced features? Yes No
Output Settings
View Workspace
Adjust modules: + - ^ v
Start Test Mode
Analyze Images
Found 7 rows

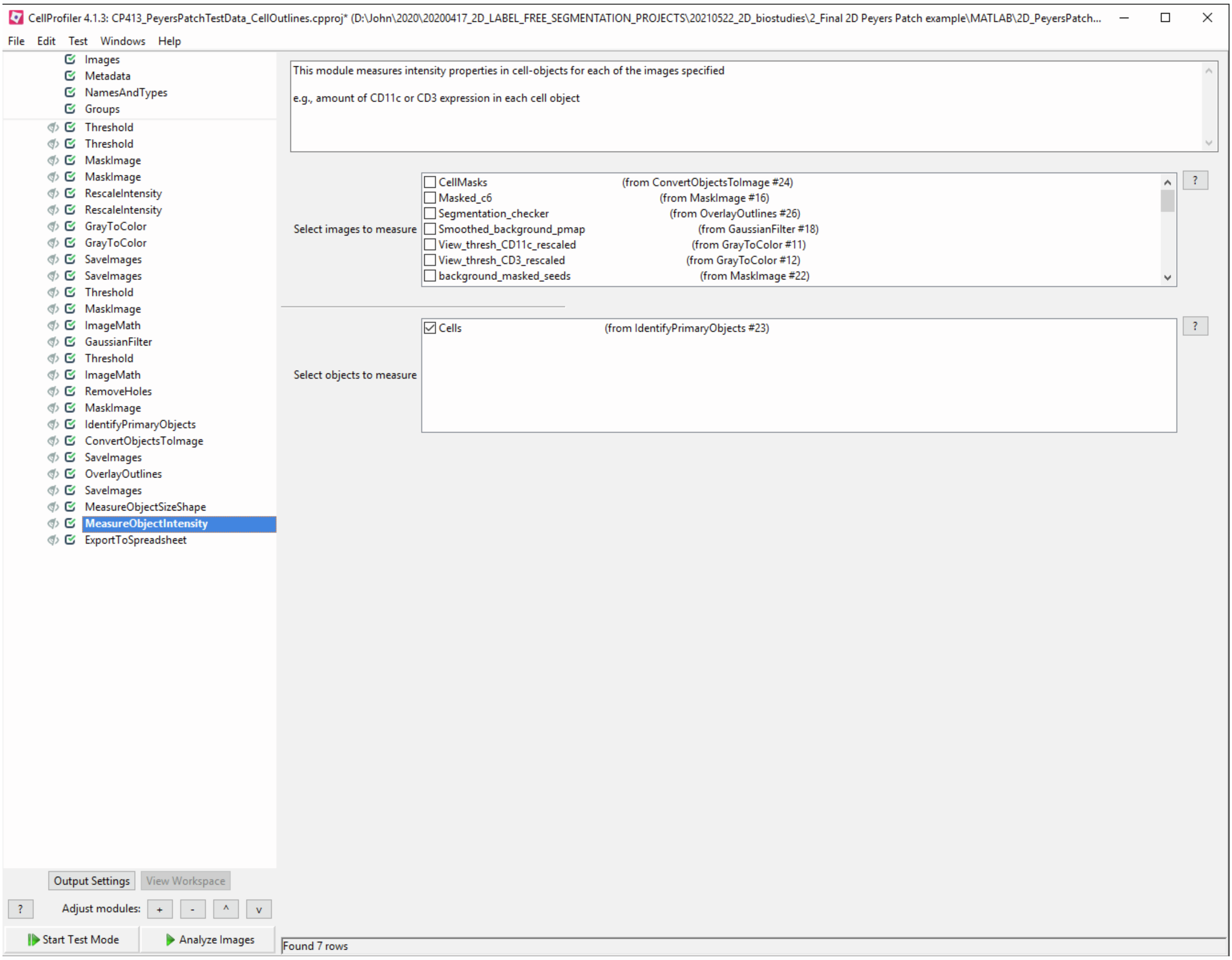
CellProfiler 4.1.3: CP413_PeyersPatchTestData_CellOutlines.cpproj* (D:\John\2020\20200417_2D_LABEL_FREE_SEGMENTATION_PROJECTS\20210522_2D_biostudies\2_Final 2D Peyers Patch example\MATLAB\2D_PeyersPatch...
File Edit Test Windows Help
Images
Metadata
NamesAndTypes
Groups
Threshold
Threshold
MaskImage
MaskImage
RescaleIntensity
RescaleIntensity
GrayToColor
GrayToColor
SaveImages
SaveImages
Threshold
MaskImage
ImageMath
GaussianFilter
Threshold
ImageMath
RemoveHoles
MaskImage
IdentifyPrimaryObjects
ConvertObjectsToImage
SaveImages
OverlayOutlines
SaveImages
MeasureObjectSizeShape
MeasureObjectIntensity
ExportToSpreadsheet
This module measures intensity properties in cell-objects for each of the images specified
e.g., amount of CD11c or CD3 expression in each cell object
Select images to measure
CellMasks (from ConvertObjectsToImage #24)
Masked_c6 (from MaskImage #16)
Segmentation_checker (from OverlayOutlines #26)
Smoothed_background_pmap (from GaussianFilter #18)
View_thresh_CD11c_rescaled (from GrayToColor #11)
View_thresh_CD3_rescaled (from GrayToColor #12)
background_masked_seeds (from MaskImage #22)
Select objects to measure
Cells (from IdentifyPrimaryObjects #23)
Output Settings
View Workspace
Adjust modules:
Start Test Mode
Analyze Images
Found 7 rows

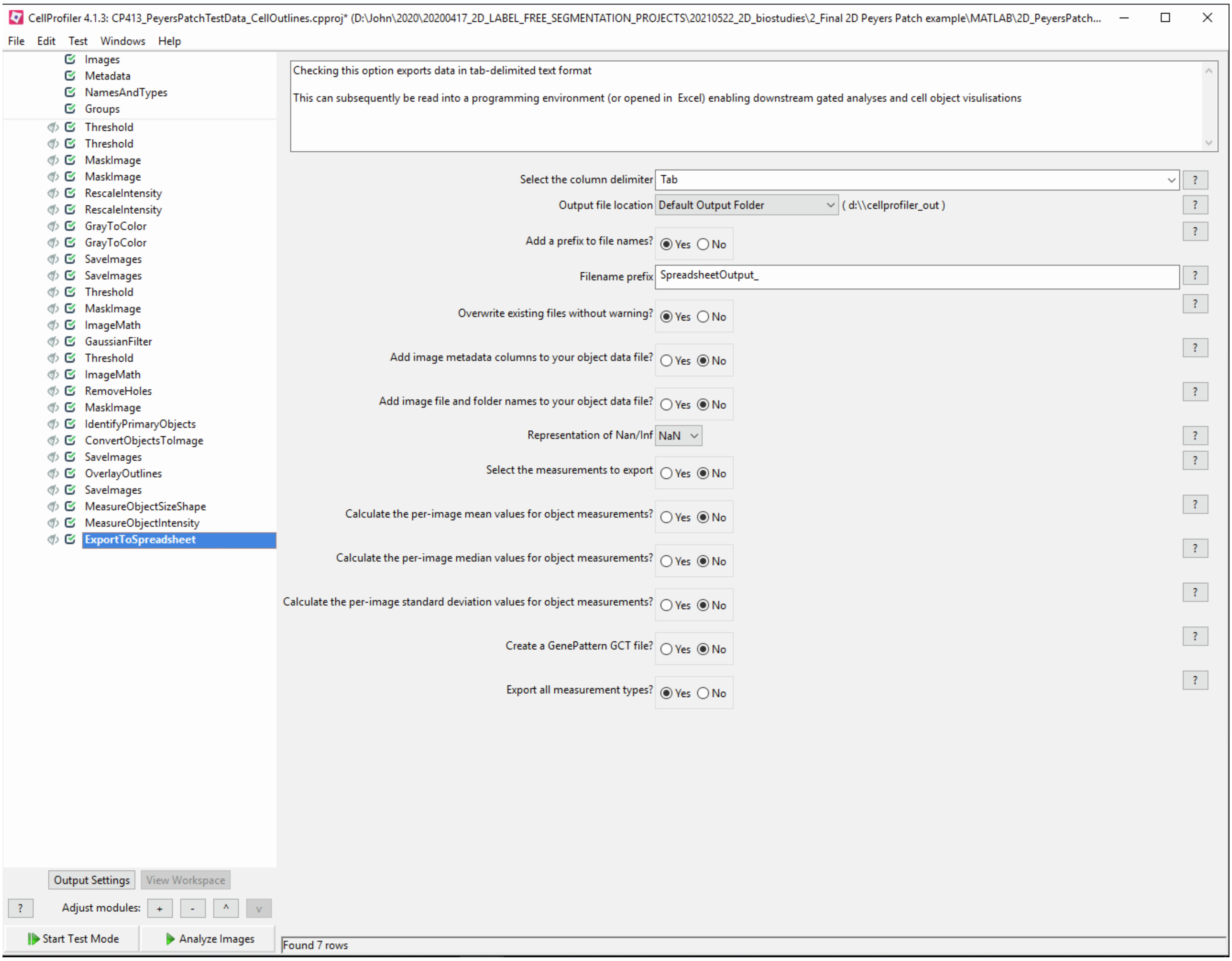
CellProfiler 4.1.3: CP413_PeyersPatchTestData_CellOutlines.cpproj* (D:\John\2020\20200417_2D_LABEL_FREE_SEGMENTATION_PROJECTS\20210522_2D_biostudies\2_Final 2D Peyers Patch example\MATLAB\2D_PeyersPatch...
File Edit Test Windows Help
Images
Metadata
NamesAndTypes
Groups
Threshold
Threshold
MaskImage
MaskImage
RescaleIntensity
RescaleIntensity
GrayToColor
GrayToColor
SaveImages
SaveImages
Threshold
MaskImage
ImageMath
GaussianFilter
Threshold
ImageMath
RemoveHoles
MaskImage
IdentifyPrimaryObjects
ConvertObjectsToImage
SaveImages
OverlayOutlines
SaveImages
MeasureObjectSizeShape
MeasureObjectIntensity
ExportToSpreadsheet
Checking this option exports data in tab-delimited text format
This can subsequently be read into a programming environment (or opened in Excel) enabling downstream gated analyses and cell object visulisations
Select the column delimiter Tab
Output file location Default Output Folder (d:\\cellprofiler_out)
Add a prefix to file names? Yes No
Filename prefix SpreadsheetOutput_
Overwrite existing files without warning? Yes No
Add image metadata columns to your object data file? Yes No
Add image file and folder names to your object data file? Yes No
Representation of Nan/Inf NaN
Select the measurements to export Yes No
Calculate the per-image mean values for object measurements? Yes No
Calculate the per-image median values for object measurements? Yes No
Calculate the per-image standard deviation values for object measurements? Yes No
Create a GenePattern GCT file? Yes No
Export all measurement types? Yes No
Output Settings
View Workspace
Adjust modules:
Start Test Mode
Analyze Images
Found 7 rows

3-D CellProfiler pipeline. This section presents screenshots of a CellProfiler image analysis pipeline used to achieve label-free cell segmentation in 3-D from the Unet network outputs and to measure the intensity and size/shape features of identified cell-objects. To use the image analysis pipeline with new image data, the 'IdentifyPrimaryObjects' module simply needs adjusting so that the 'typical diameter of objects' size-range matches the pixel scaling of the new images. For newcomers to CellProfiler, we recommend downloading the image-data and pipeline from BioStudies database https://www.ebi.ac.uk/biostudies/ under accession number S-BSST742. This enables the pipeline to be run with the data described in the manuscript and allows the user to see how each module works.

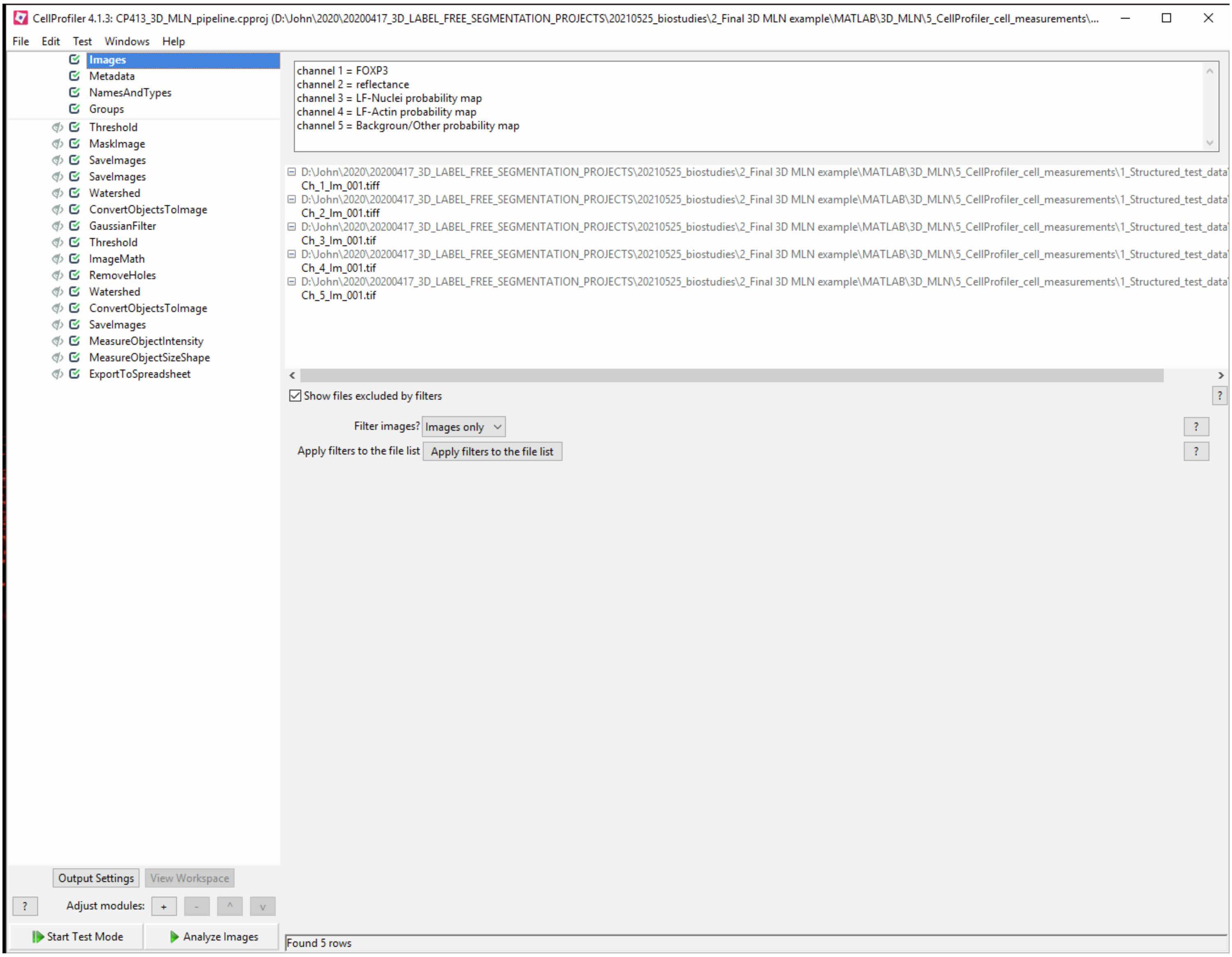

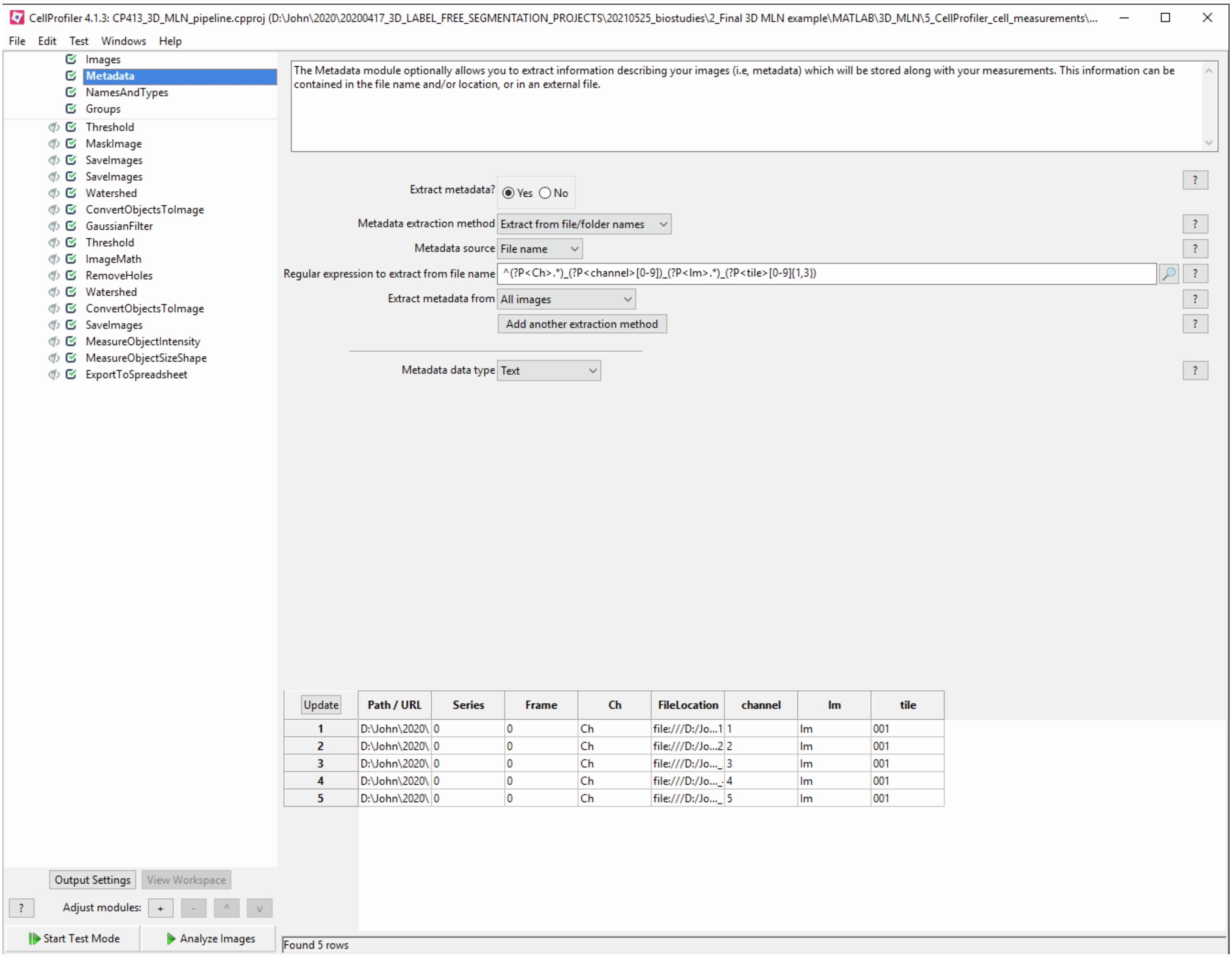
CellProfiler 4.1.3: CP413_3D_MLN_pipeline.cpproj (D:\John\2020\20200417_3D_LABEL_FREE_SEGMENTATION_PROJECTS\20210525_biostudies\2_Final 3D MLN example\MATLAB\3D_MLN\5_CellProfiler_cell_measurements\...
File Edit Test Windows Help
Images
Metadata
NamesAndTypes
Groups
Threshold
MaskImage
SaveImages
SaveImages
Watershed
ConvertObjectsToImage
GaussianFilter
Threshold
ImageMath
RemoveHoles
Watershed
ConvertObjectsToImage
SaveImages
MeasureObjectIntensity
MeasureObjectSizeShape
ExportToSpreadsheet
The Metadata module optionally allows you to extract information describing your images (i.e, metadata) which will be stored along with your measurements. This information can be contained in the file name and/or location, or in an external file.
Extract metadata? Yes No
Metadata extraction method Extract from file/folder names
Metadata source File name
Regular expression to extract from file name ^(?P<Ch>.*)_(?P<channel>[0-9])_(?P<Im>.*)_(?P<tile>[0-9]{1,3})
Extract metadata from All images
Add another extraction method
Metadata data type Text
Update Path / URL Series Frame Ch FileLocation channel Im tile
1 D:\John\2020\ 0 0 Ch file:///D:/Jo...1 1 Im 001
2 D:\John\2020\ 0 0 Ch file:///D:/Jo...2 2 Im 001
3 D:\John\2020\ 0 0 Ch file:///D:/Jo..._ 3 Im 001
4 D:\John\2020\ 0 0 Ch file:///D:/Jo..._ 4 Im 001
5 D:\John\2020\ 0 0 Ch file:///D:/Jo..._ 5 Im 001
Output Settings View Workspace
Adjust modules: + - ^ v
Start Test Mode Analyze Images
Found 5 rows

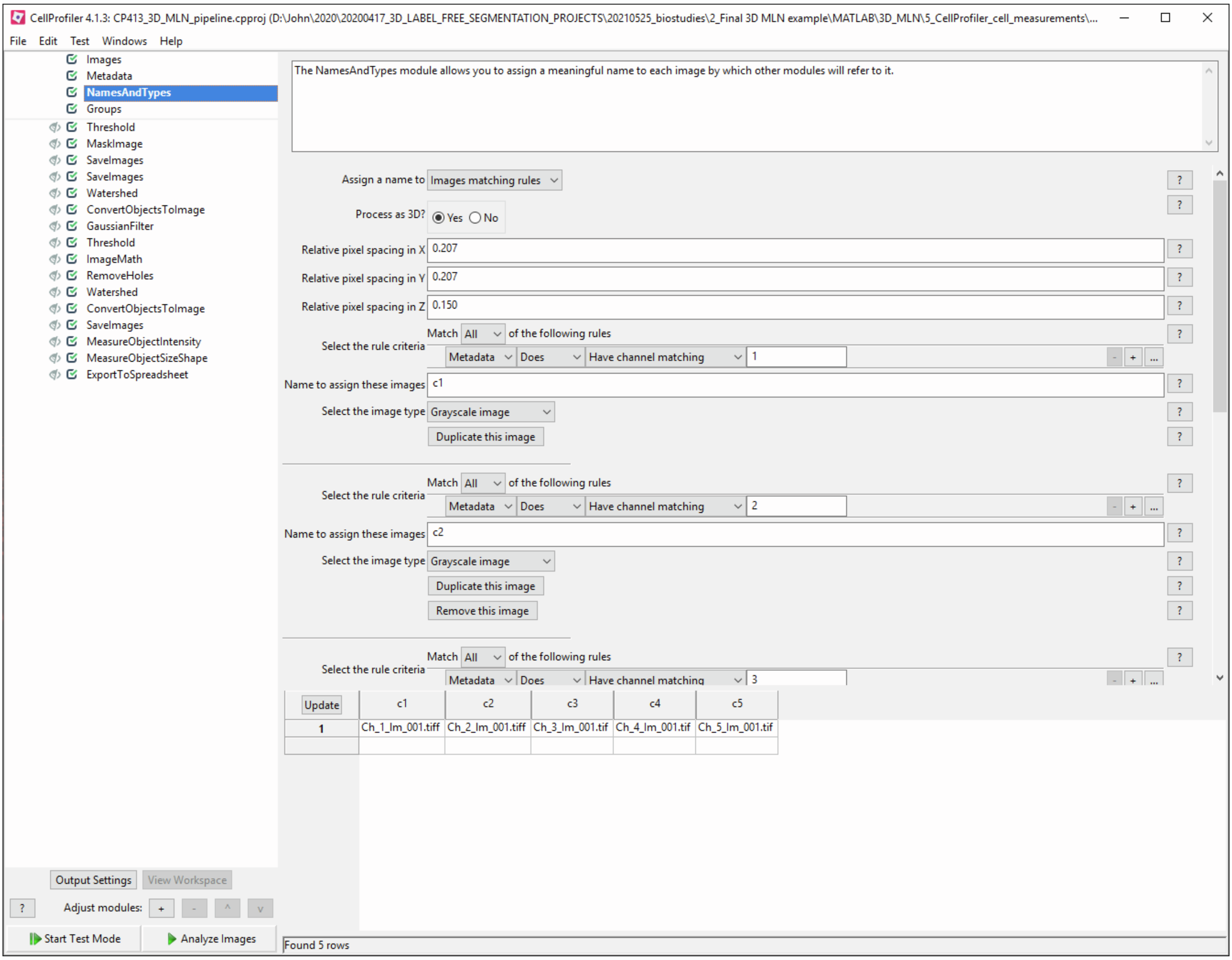
CellProfiler 4.1.3: CP413_3D_MLN_pipeline.cpproj (D:\John\2020\20200417_3D_LABEL_FREE_SEGMENTATION_PROJECTS\20210525_biostudies\2_Final 3D MLN example\MATLAB\3D_MLN\5_CellProfiler_cell_measurements\...
File
Edit
Test
Windows
Help
Images
Metadata
NamesAndTypes
Groups
Threshold
MaskImage
SaveImages
SaveImages
Watershed
ConvertObjectsToImage
GaussianFilter
Threshold
ImageMath
RemoveHoles
Watershed
ConvertObjectsToImage
SaveImages
MeasureObjectIntensity
MeasureObjectSizeShape
ExportToSpreadsheet
The NamesAndTypes module allows you to assign a meaningful name to each image by which other modules will refer to it.
Assign a name to
Images matching rules
Process as 3D?
Yes
No
Relative pixel spacing in X
0.207
Relative pixel spacing in Y
0.207
Relative pixel spacing in Z
0.150
Select the rule criteria
Match
All
of the following rules
Metadata
Does
Have channel matching
1
Name to assign these images
c1
Select the image type
Grayscale image
Duplicate this image
Select the rule criteria
Match
All
of the following rules
Metadata
Does
Have channel matching
2
Name to assign these images
c2
Select the image type
Grayscale image
Duplicate this image
Remove this image
Select the rule criteria
Match
All
of the following rules
Metadata
Does
Have channel matching
3
Update
c1
c2
c3
c4
c5
1
Ch_1_Im_001.tiff
Ch_2_Im_001.tiff
Ch_3_Im_001.tif
Ch_4_Im_001.tif
Ch_5_Im_001.tif
Output Settings
View Workspace
Adjust modules:
Start Test Mode
Analyze Images
Found 5 rows

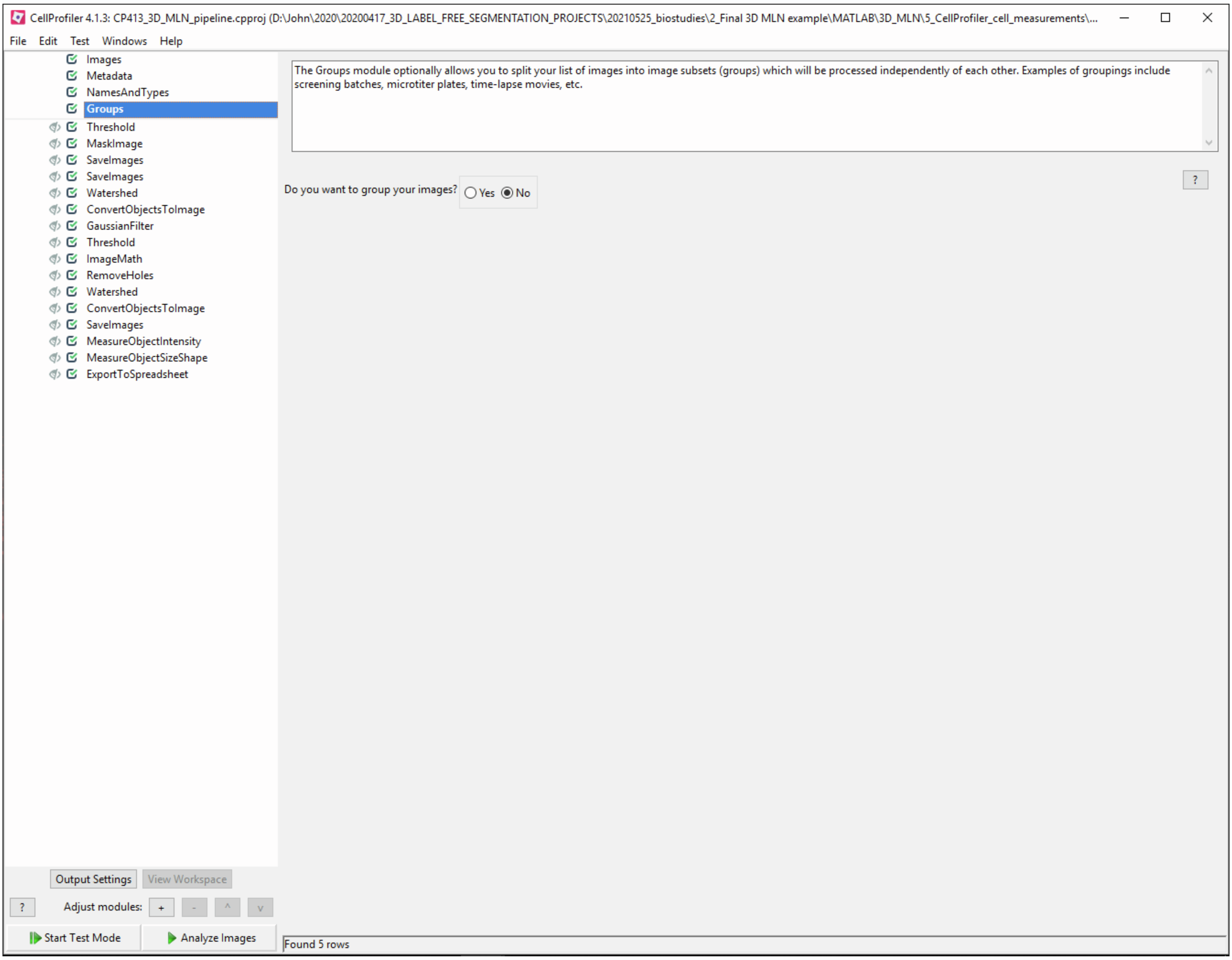
CellProfiler 4.1.3: CP413_3D_MLN_pipeline.cpproj (D:\John\2020\20200417_3D_LABEL_FREE_SEGMENTATION_PROJECTS\20210525_biostudies\2_Final 3D MLN example\MATLAB\3D_MLN\5_CellProfiler_cell_measurements\...
File Edit Test Windows Help
Images
Metadata
NamesAndTypes
Groups
Threshold
MaskImage
SaveImages
SaveImages
Watershed
ConvertObjectsToImage
GaussianFilter
Threshold
ImageMath
RemoveHoles
Watershed
ConvertObjectsToImage
SaveImages
MeasureObjectIntensity
MeasureObjectSizeShape
ExportToSpreadsheet
The Groups module optionally allows you to split your list of images into image subsets (groups) which will be processed independently of each other. Examples of groupings include screening batches, microtiter plates, time-lapse movies, etc.
Do you want to group your images?
Yes
No
Output Settings
View Workspace
Adjust modules:
Start Test Mode
Analyze Images
Found 5 rows

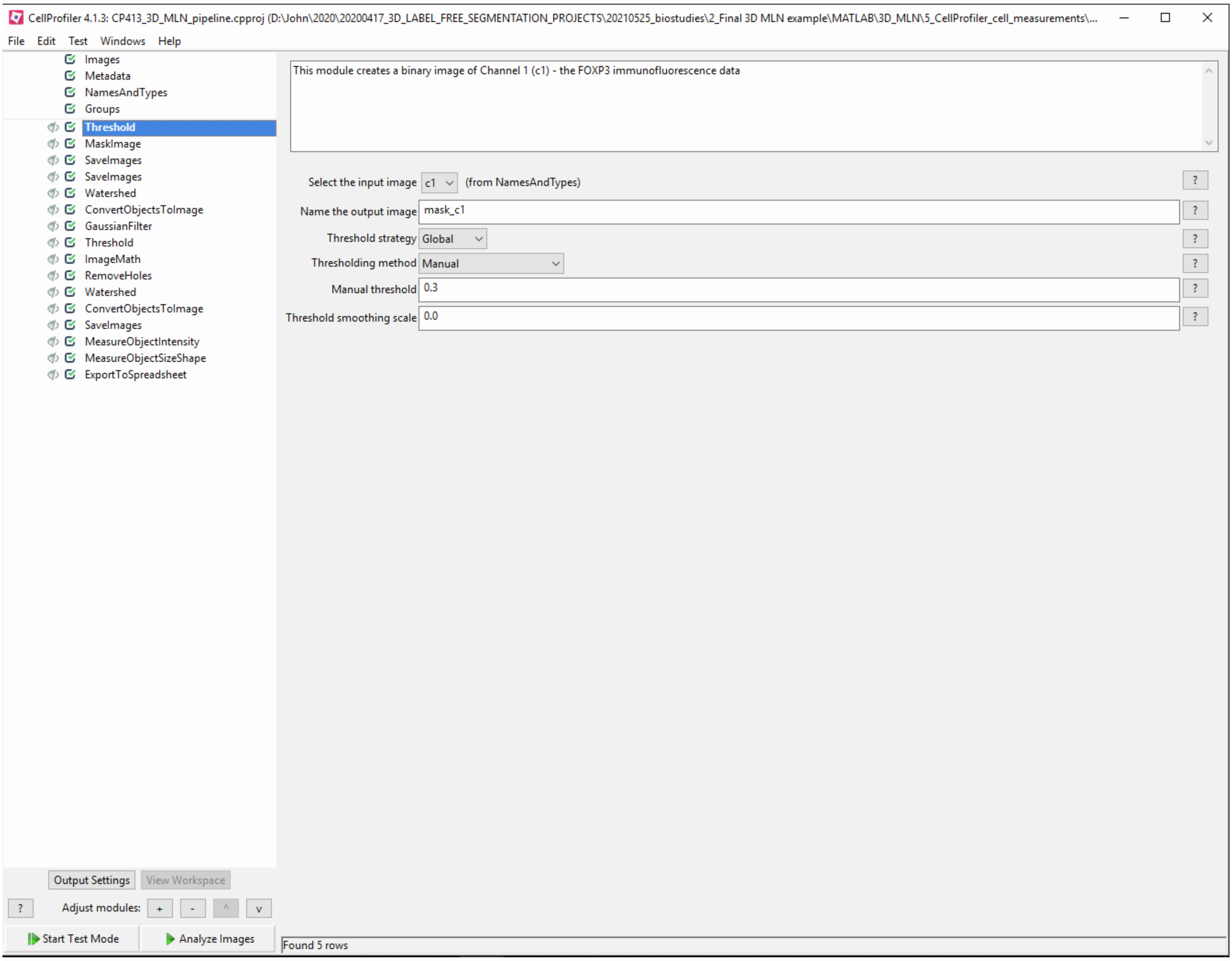
CellProfiler 4.1.3: CP413_3D_MLN_pipeline.cpproj (D:\John\2020\20200417_3D_LABEL_FREE_SEGMENTATION_PROJECTS\20210525_biostudies\2_Final 3D MLN example\MATLAB\3D_MLN\5_CellProfiler_cell_measurements\...
File Edit Test Windows Help
Images
Metadata
NamesAndTypes
Groups
Threshold
MaskImage
SaveImages
SaveImages
Watershed
ConvertObjectsToImage
GaussianFilter
Threshold
ImageMath
RemoveHoles
Watershed
ConvertObjectsToImage
SaveImages
MeasureObjectIntensity
MeasureObjectSizeShape
ExportToSpreadsheet
This module creates a binary image of Channel 1 (c1) - the FOXP3 immunofluorescence data
Select the input image c1 (from NamesAndTypes)
Name the output image mask_c1
Threshold strategy Global
Thresholding method Manual
Manual threshold 0.3
Threshold smoothing scale 0.0
Output Settings
View Workspace
Adjust modules: + - ^ v
Start Test Mode
Analyze Images
Found 5 rows

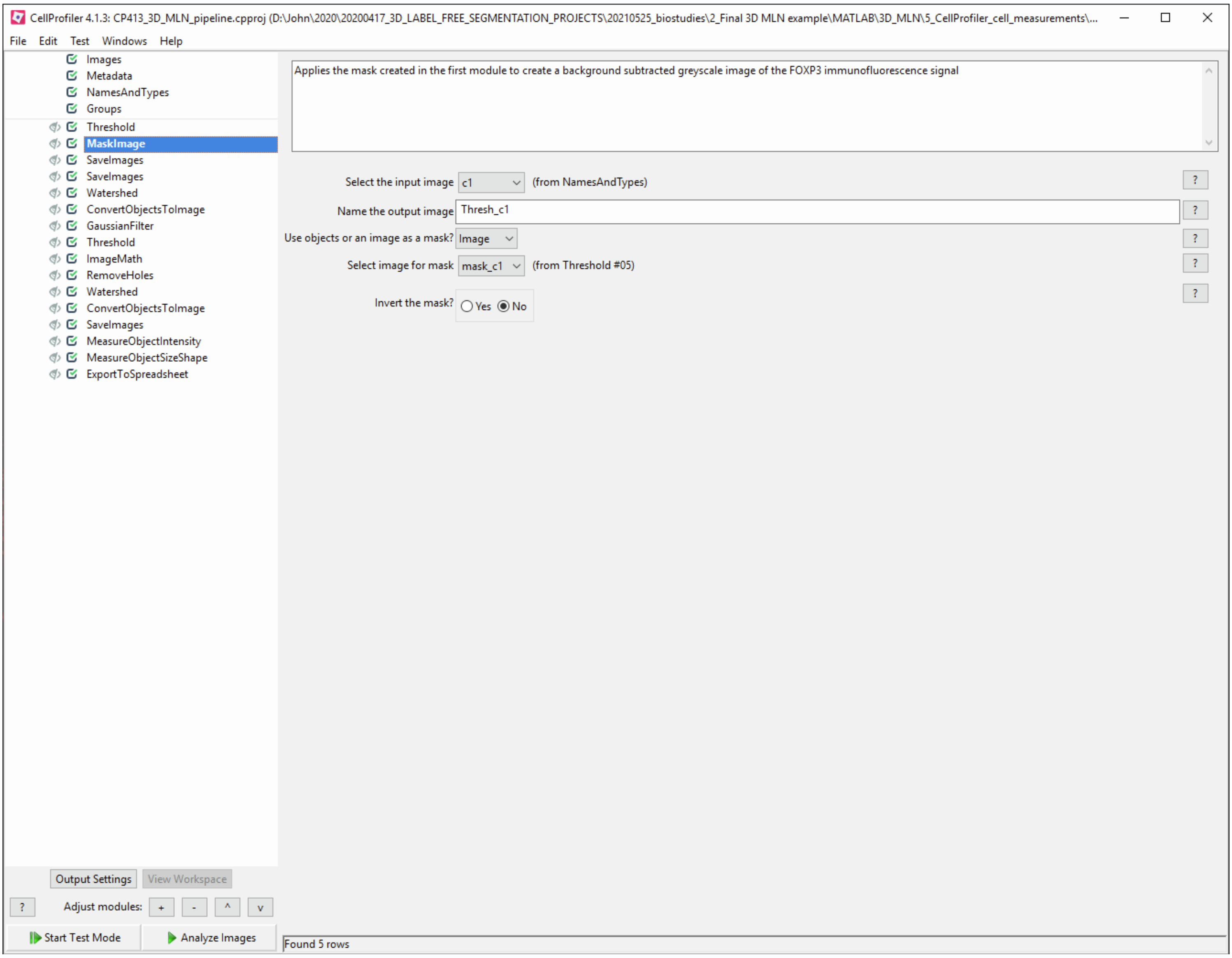
CellProfiler 4.1.3: CP413_3D_MLN_pipeline.cpproj (D:\John\2020\20200417_3D_LABEL_FREE_SEGMENTATION_PROJECTS\20210525_biostudies\2_Final 3D MLN example\MATLAB\3D_MLN\5_CellProfiler_cell_measurements\...
File Edit Test Windows Help
Images
Metadata
NamesAndTypes
Groups
Threshold
MaskImage
SaveImages
SaveImages
Watershed
ConvertObjectsToImage
GaussianFilter
Threshold
ImageMath
RemoveHoles
Watershed
ConvertObjectsToImage
SaveImages
MeasureObjectIntensity
MeasureObjectSizeShape
ExportToSpreadsheet
Applies the mask created in the first module to create a background subtracted greyscale image of the FOXP3 immunofluorescence signal
Select the input image c1 (from NamesAndTypes)
Name the output image Thresh_c1
Use objects or an image as a mask? Image
Select image for mask mask_c1 (from Threshold #05)
Invert the mask? Yes No
Output Settings
View Workspace
Adjust modules: + - ^ v
Start Test Mode
Analyze Images
Found 5 rows

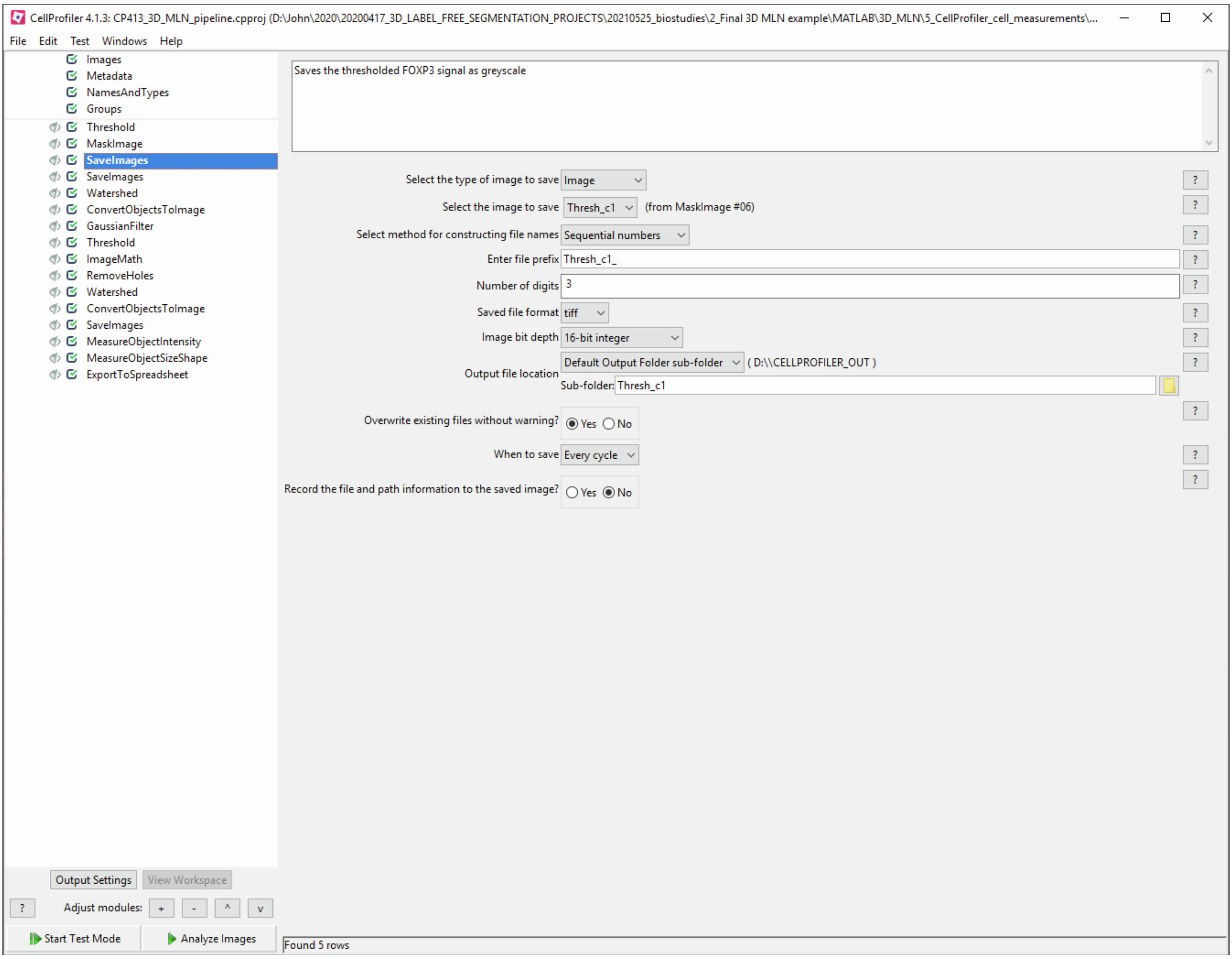
CellProfiler 4.1.3: CP413_3D_MLN_pipeline.cpproj (D:\John\2020\20200417_3D_LABEL_FREE_SEGMENTATION_PROJECTS\20210525_biostudies\2_Final 3D MLN example\MATLAB\3D_MLN\5_CellProfiler_cell_measurements\...
File Edit Test Windows Help
Images
Metadata
NamesAndTypes
Groups
Threshold
MaskImage
SaveImages
SaveImages
Watershed
ConvertObjectsToImage
GaussianFilter
Threshold
ImageMath
RemoveHoles
Watershed
ConvertObjectsToImage
SaveImages
MeasureObjectIntensity
MeasureObjectSizeShape
ExportToSpreadsheet
Saves the thresholded FOXP3 signal as greyscale
Select the type of image to save Image
Select the image to save Thresh_c1 (from MaskImage #06)
Select method for constructing file names Sequential numbers
Enter file prefix Thresh_c1_
Number of digits 3
Saved file format tiff
Image bit depth 16-bit integer
Output file location Default Output Folder sub-folder (D:\\CELLPROFILER_OUT)
Sub-folder: Thresh_c1
Overwrite existing files without warning? Yes No
When to save Every cycle
Record the file and path information to the saved image? Yes No
Output Settings
View Workspace
Adjust modules: + - ^ v
Start Test Mode
Analyze Images
Found 5 rows

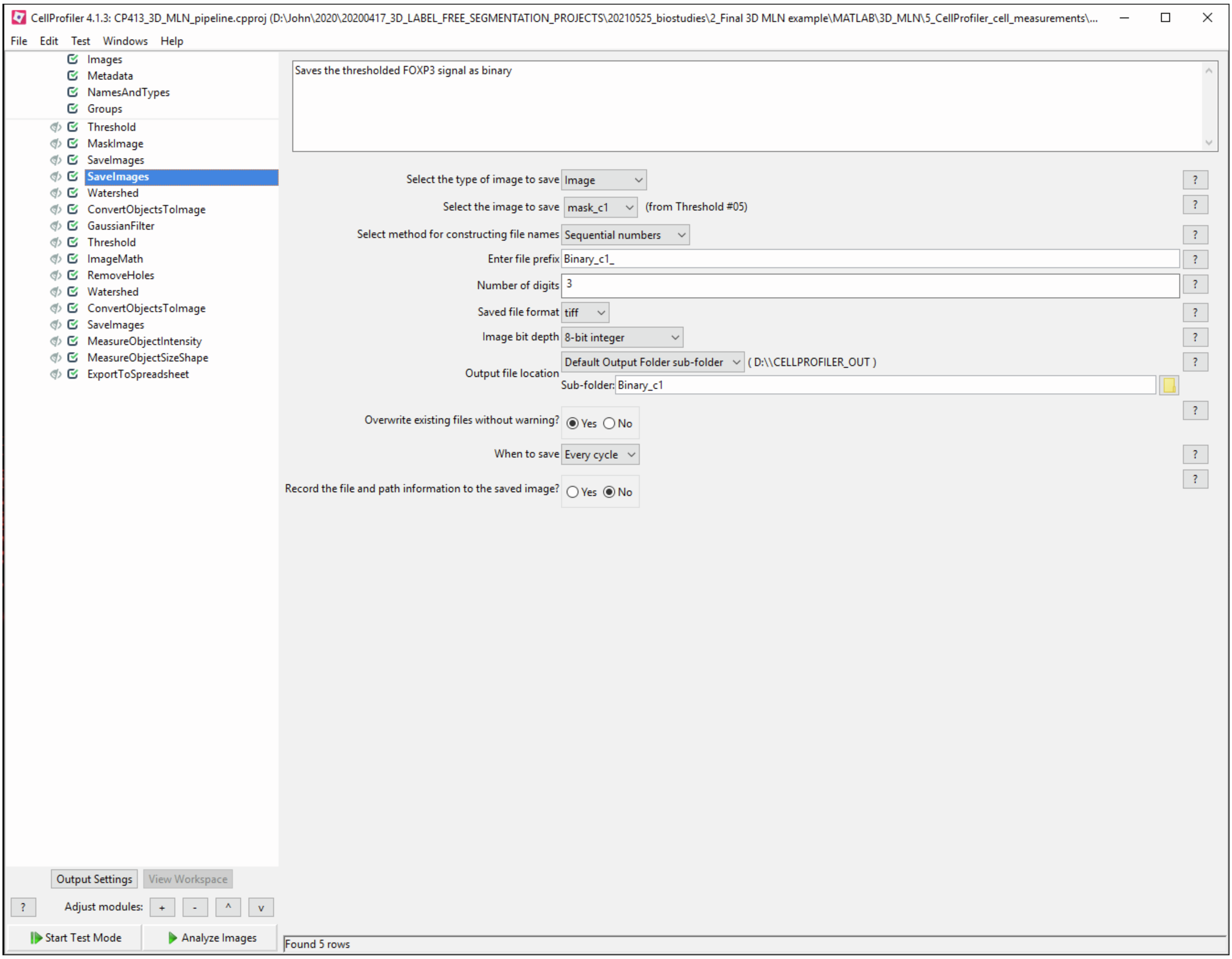
CellProfiler 4.1.3: CP413_3D_MLN_pipeline.cpproj (D:\John\2020\20200417_3D_LABEL_FREE_SEGMENTATION_PROJECTS\20210525_biostudies\2_Final 3D MLN example\MATLAB\3D_MLN\5_CellProfiler_cell_measurements\...
File Edit Test Windows Help
Images
Metadata
NamesAndTypes
Groups
Threshold
MaskImage
SaveImages
SaveImages
Watershed
ConvertObjectsToImage
GaussianFilter
Threshold
ImageMath
RemoveHoles
Watershed
ConvertObjectsToImage
SaveImages
MeasureObjectIntensity
MeasureObjectSizeShape
ExportToSpreadsheet
Saves the thresholded FOXP3 signal as binary
Select the type of image to save Image
Select the image to save mask_c1 (from Threshold #05)
Select method for constructing file names Sequential numbers
Enter file prefix Binary_c1_
Number of digits 3
Saved file format tiff
Image bit depth 8-bit integer
Output file location Default Output Folder sub-folder (D:\\CELLPROFILER_OUT)
Sub-folder: Binary_c1
Overwrite existing files without warning? Yes No
When to save Every cycle
Record the file and path information to the saved image? Yes No
Output Settings
View Workspace
Adjust modules: + - ^ v
Start Test Mode
Analyze Images
Found 5 rows

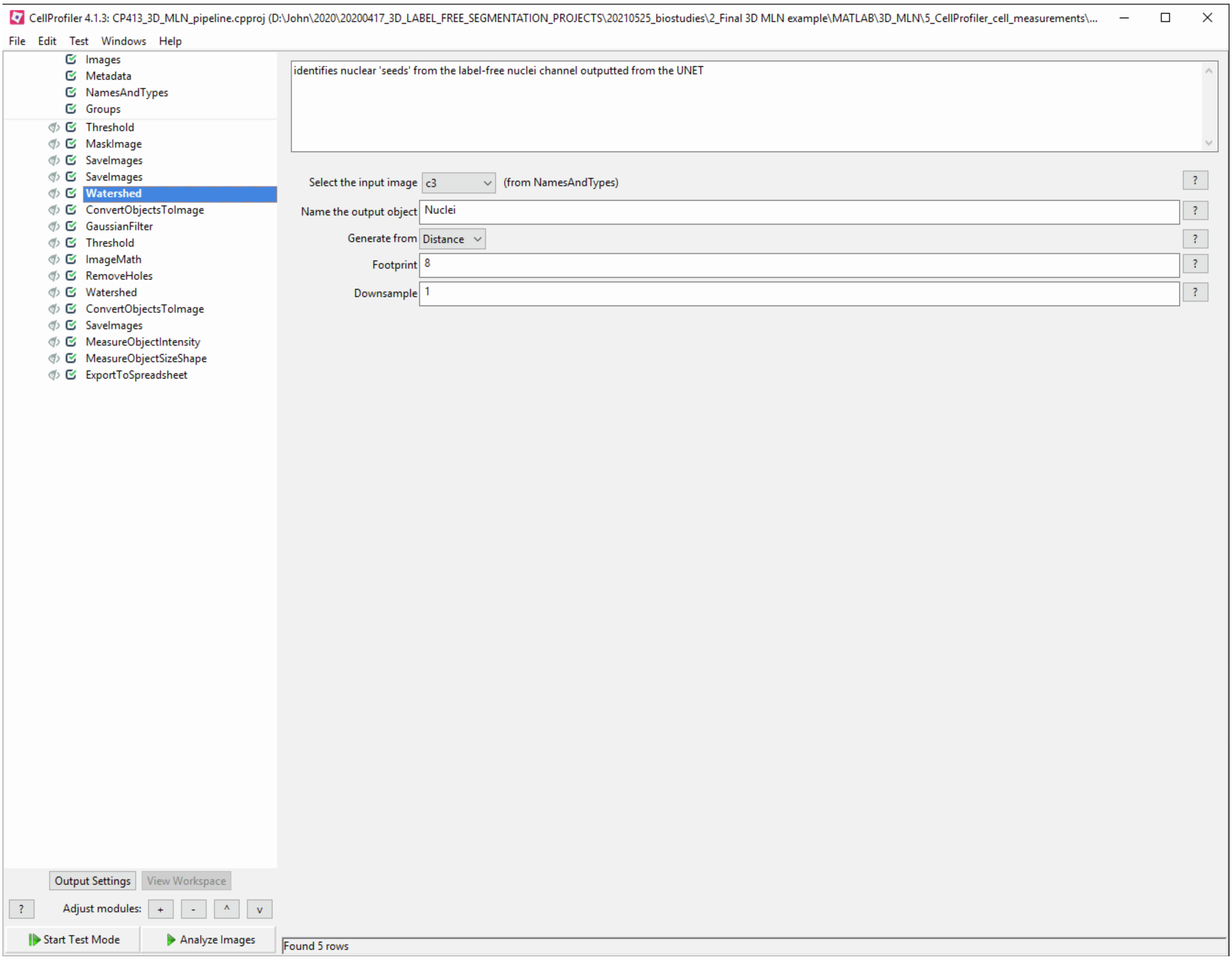
CellProfiler 4.1.3: CP413_3D_MLN_pipeline.cpproj (D:\John\2020\20200417_3D_LABEL_FREE_SEGMENTATION_PROJECTS\20210525_biostudies\2_Final 3D MLN example\MATLAB\3D_MLN\5_CellProfiler_cell_measurements\...
File Edit Test Windows Help
Images
Metadata
NamesAndTypes
Groups
Threshold
MaskImage
SaveImages
SaveImages
Watershed
ConvertObjectsToImage
GaussianFilter
Threshold
ImageMath
RemoveHoles
Watershed
ConvertObjectsToImage
SaveImages
MeasureObjectIntensity
MeasureObjectSizeShape
ExportToSpreadsheet
identifies nuclear 'seeds' from the label-free nuclei channel outputted from the UNET
Select the input image c3 (from NamesAndTypes)
Name the output object Nuclei
Generate from Distance
Footprint 8
Downsample 1
Output Settings
View Workspace
Adjust modules: + - ^ v
Start Test Mode
Analyze Images
Found 5 rows

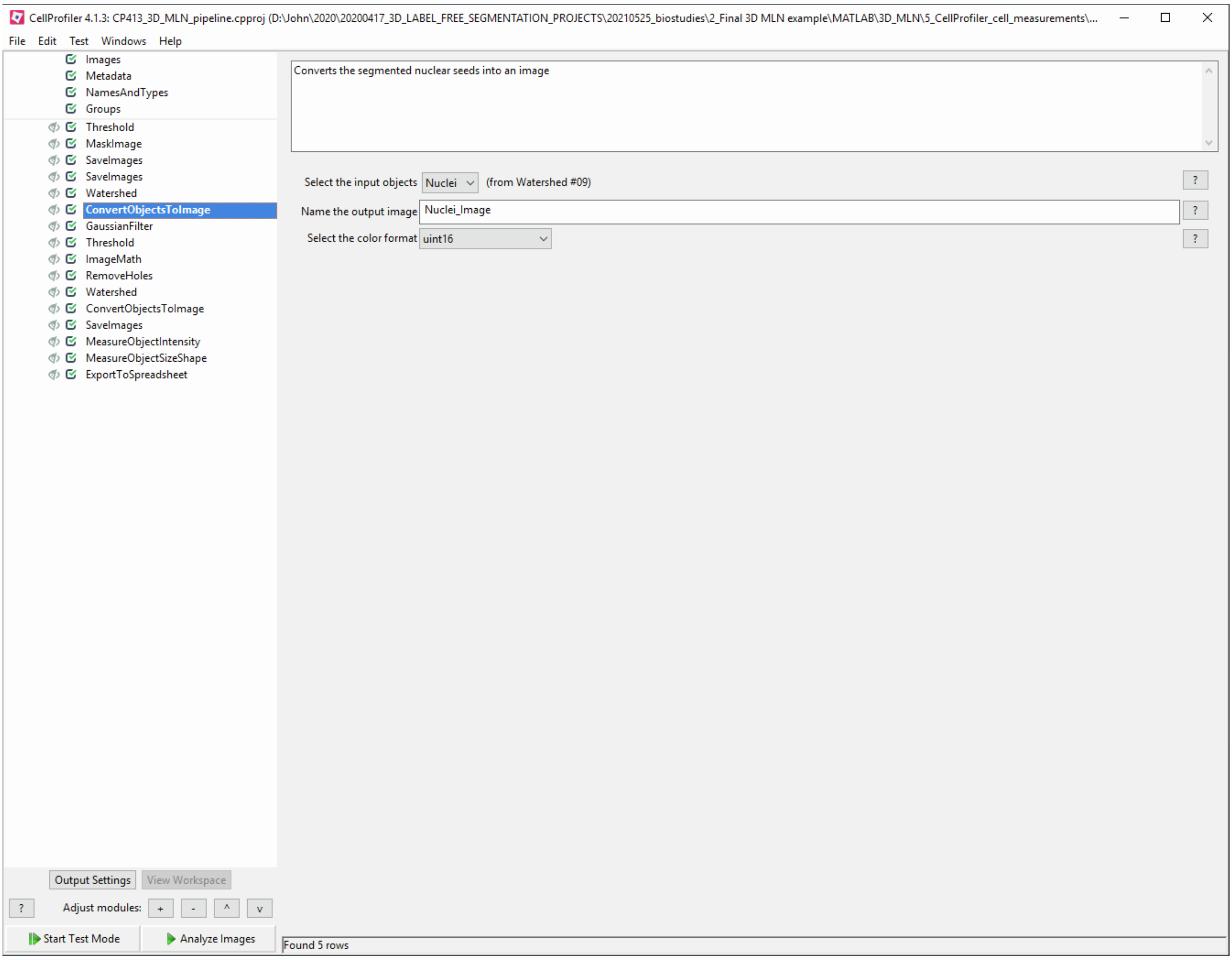
CellProfiler 4.1.3: CP413_3D_MLN_pipeline.cpproj (D:\John\2020\20200417_3D_LABEL_FREE_SEGMENTATION_PROJECTS\20210525_biostudies\2_Final 3D MLN example\MATLAB\3D_MLN\5_CellProfiler_cell_measurements\...
File Edit Test Windows Help
Images
Metadata
NamesAndTypes
Groups
Threshold
MaskImage
SaveImages
SaveImages
Watershed
ConvertObjectsToImage
GaussianFilter
Threshold
ImageMath
RemoveHoles
Watershed
ConvertObjectsToImage
SaveImages
MeasureObjectIntensity
MeasureObjectSizeShape
ExportToSpreadsheet
Converts the segmented nuclear seeds into an image
Select the input objects
Nuclei
(from Watershed #09)
Name the output image
Nuclei_Image
Select the color format
uint16
Output Settings
View Workspace
Adjust modules:
Start Test Mode
Analyze Images
Found 5 rows

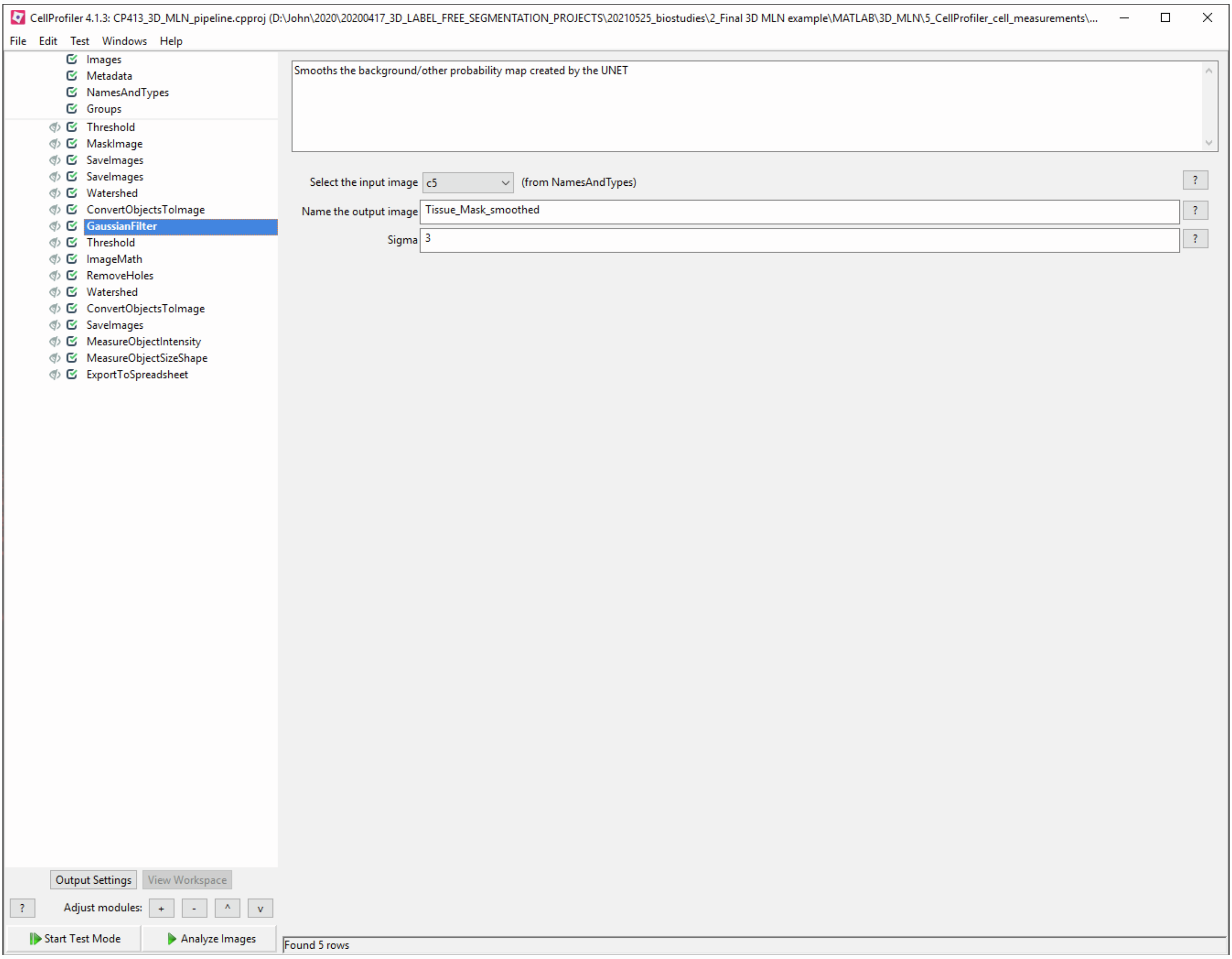
CellProfiler 4.1.3: CP413_3D_MLN_pipeline.cpproj (D:\John\2020\20200417_3D_LABEL_FREE_SEGMENTATION_PROJECTS\20210525_biostudies\2_Final 3D MLN example\MATLAB\3D_MLN\5_CellProfiler_cell_measurements\...
File Edit Test Windows Help
Images
Metadata
NamesAndTypes
Groups
Threshold
MaskImage
SaveImages
SaveImages
Watershed
ConvertObjectsToImage
GaussianFilter
Threshold
ImageMath
RemoveHoles
Watershed
ConvertObjectsToImage
SaveImages
MeasureObjectIntensity
MeasureObjectSizeShape
ExportToSpreadsheet
Smooths the background/other probability map created by the UNET
Select the input image c5 (from NamesAndTypes)
Name the output image Tissue_Mask_smoothed
Sigma 3
Output Settings
View Workspace
Adjust modules:
Start Test Mode
Analyze Images
Found 5 rows

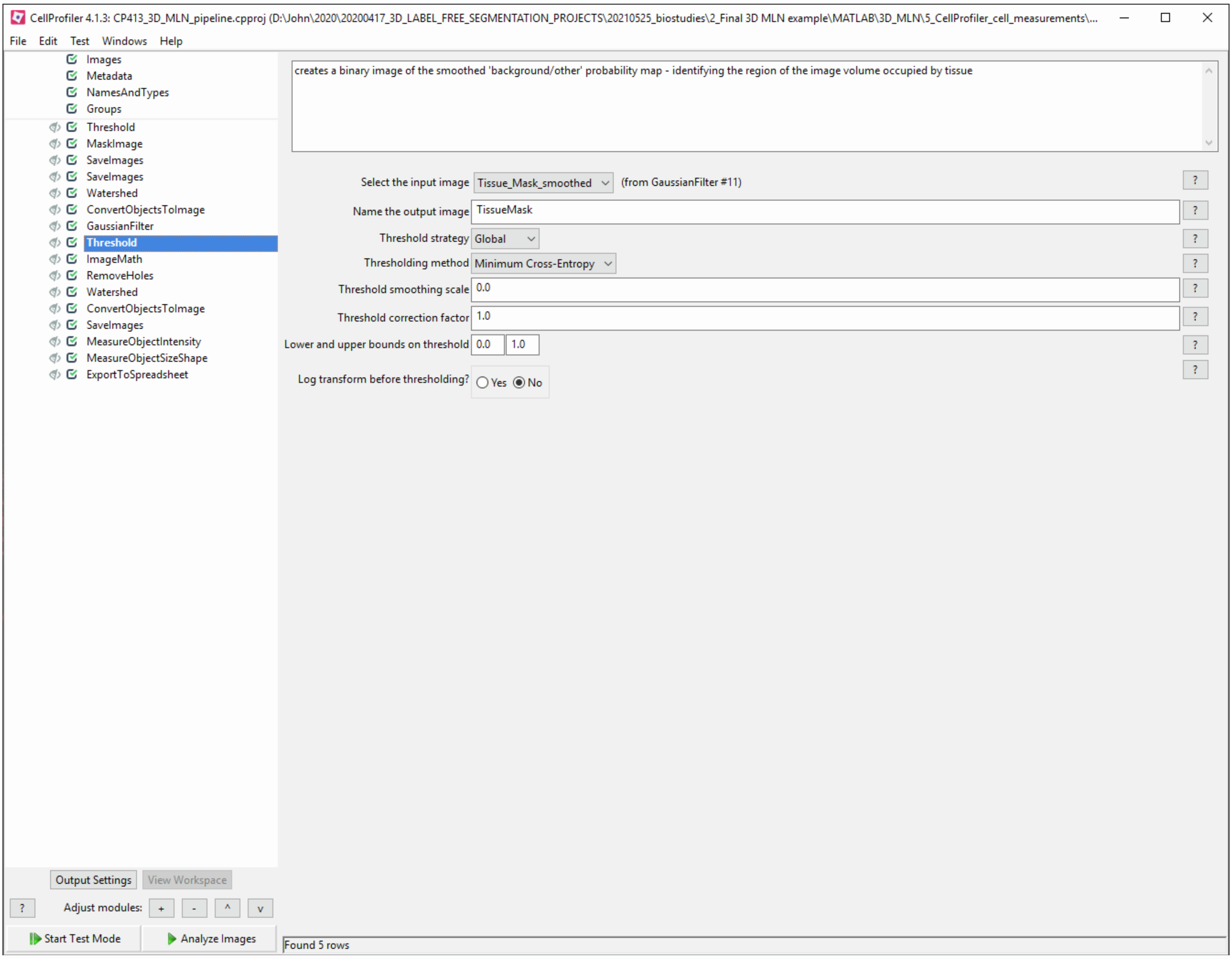
CellProfiler 4.1.3: CP413_3D_MLN_pipeline.cpproj (D:\John\2020\20200417_3D_LABEL_FREE_SEGMENTATION_PROJECTS\20210525_biostudies\2_Final 3D MLN example\MATLAB\3D_MLN\5_CellProfiler_cell_measurements\...
File Edit Test Windows Help
Images
Metadata
NamesAndTypes
Groups
Threshold
MaskImage
SaveImages
SaveImages
Watershed
ConvertObjectsToImage
GaussianFilter
Threshold
ImageMath
RemoveHoles
Watershed
ConvertObjectsToImage
SaveImages
MeasureObjectIntensity
MeasureObjectSizeShape
ExportToSpreadsheet
creates a binary image of the smoothed 'background/other' probability map - identifying the region of the image volume occupied by tissue
Select the input image Tissue_Mask_smoothed (from GaussianFilter #11)
Name the output image TissueMask
Threshold strategy Global
Thresholding method Minimum Cross-Entropy
Threshold smoothing scale 0.0
Threshold correction factor 1.0
Lower and upper bounds on threshold 0.0 1.0
Log transform before thresholding? Yes No
Output Settings
View Workspace
Adjust modules: + - ^ v
Start Test Mode
Analyze Images
Found 5 rows

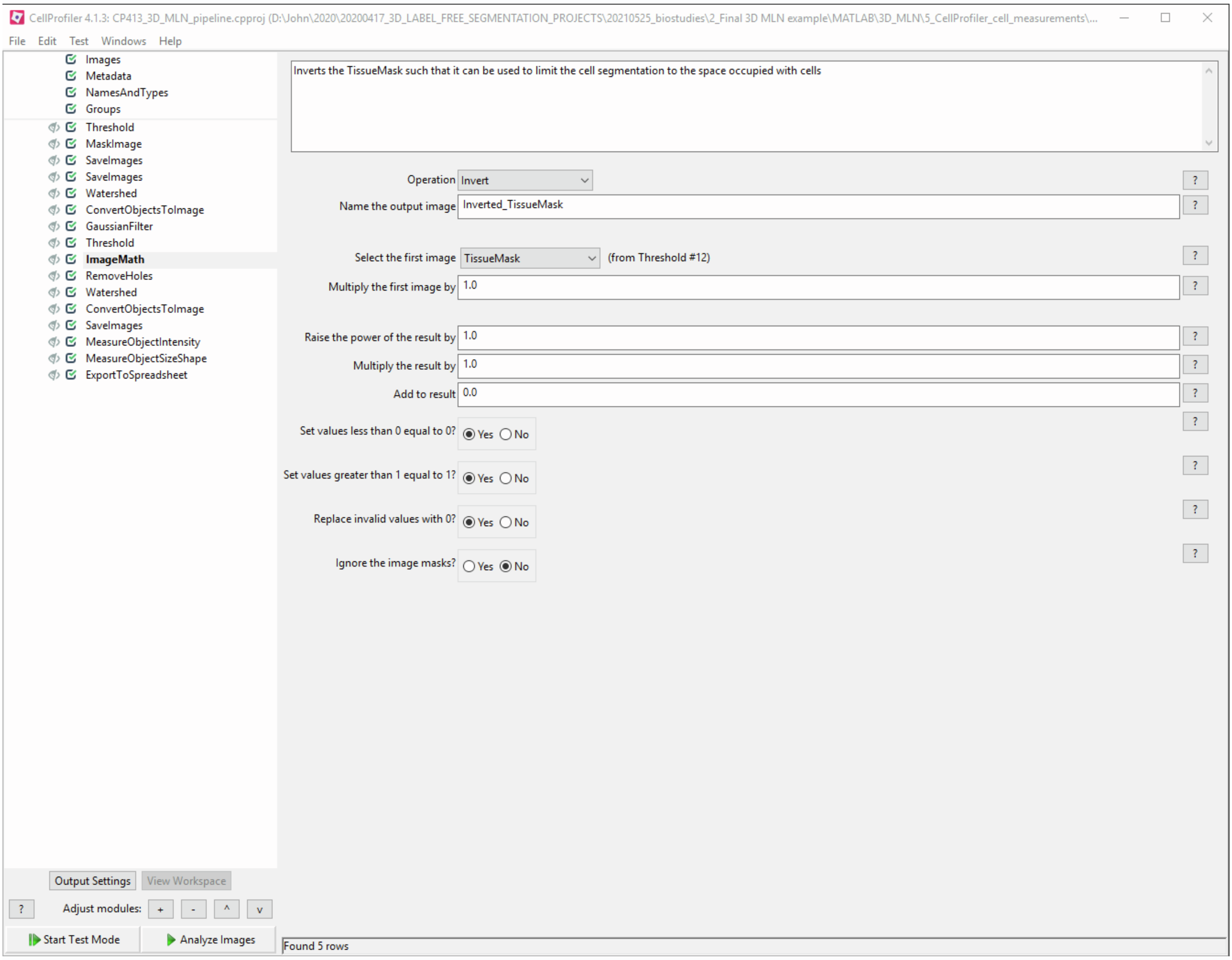

CellProfiler 4.1.3: CP413_3D_MLN_pipeline.cpproj (D:\John\2020\20200417_3D_LABEL_FREE_SEGMENTATION_PROJECTS\20210525_biostudies\2_Final 3D MLN example\MATLAB\3D_MLN\5_CellProfiler_cell_measurements\...
File Edit Test Windows Help
Images
Metadata
NamesAndTypes
Groups
Threshold
MaskImage
SaveImages
SaveImages
Watershed
ConvertObjectsToImage
GaussianFilter
Threshold
ImageMath
RemoveHoles
Watershed
ConvertObjectsToImage
SaveImages
MeasureObjectIntensity
MeasureObjectSizeShape
ExportToSpreadsheet
Inverts the TissueMask such that it can be used to limit the cell segmentation to the space occupied with cells
Operation Invert
Name the output image Inverted_TissueMask
Select the first image TissueMask (from Threshold #12)
Multiply the first image by 1.0
Raise the power of the result by 1.0
Multiply the result by 1.0
Add to result 0.0
Set values less than 0 equal to 0? Yes No
Set values greater than 1 equal to 1? Yes No
Replace invalid values with 0? Yes No
Ignore the image masks? Yes No
Output Settings
View Workspace
Adjust modules: + - ^ v
Start Test Mode
Analyze Images
Found 5 rows

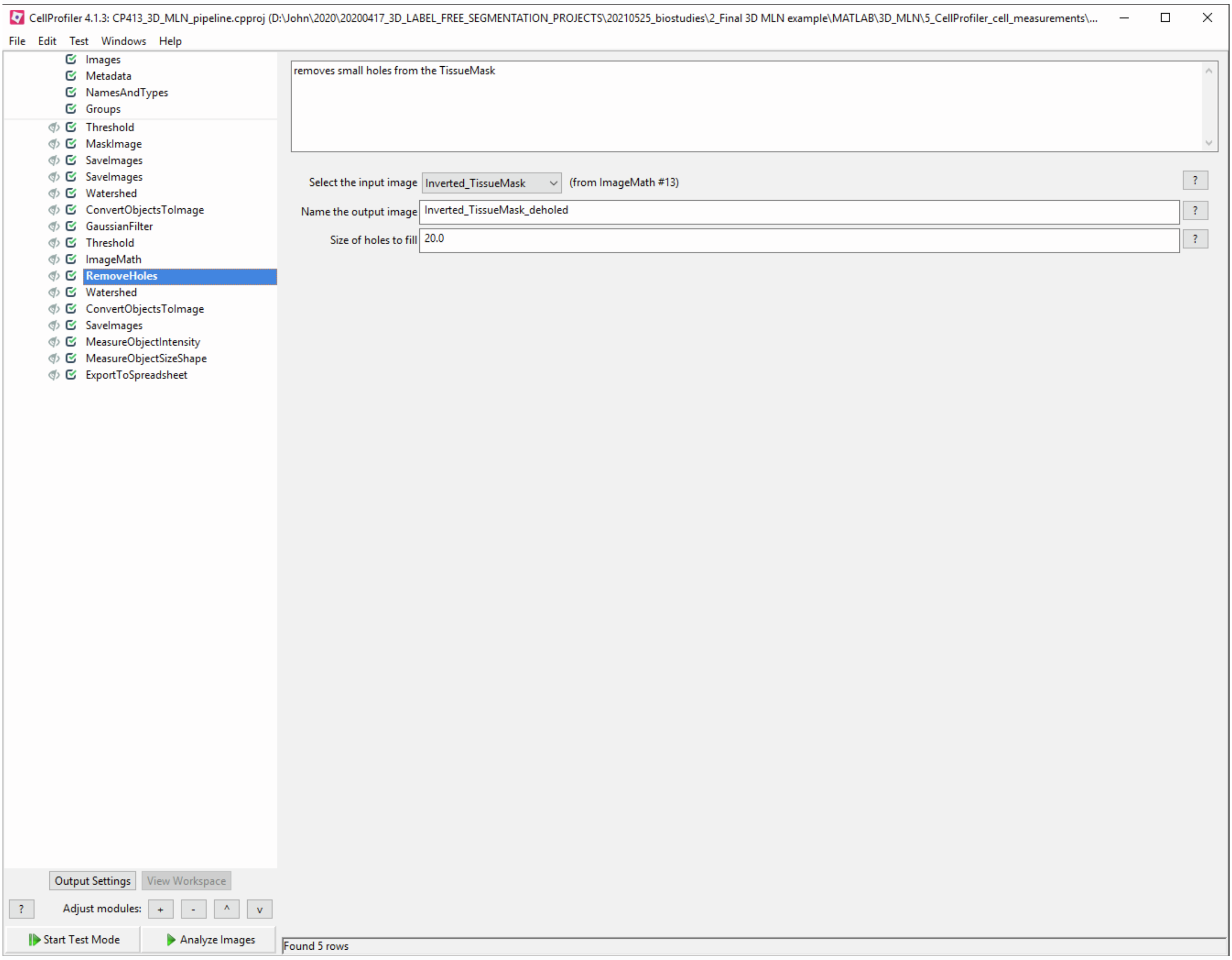
CellProfiler 4.1.3: CP413_3D_MLN_pipeline.cpproj (D:\John\2020\20200417_3D_LABEL_FREE_SEGMENTATION_PROJECTS\20210525_biostudies\2_Final 3D MLN example\MATLAB\3D_MLN\5_CellProfiler_cell_measurements\...
File Edit Test Windows Help
Images
Metadata
NamesAndTypes
Groups
Threshold
MaskImage
SaveImages
SaveImages
Watershed
ConvertObjectsToImage
GaussianFilter
Threshold
ImageMath
RemoveHoles
Watershed
ConvertObjectsToImage
SaveImages
MeasureObjectIntensity
MeasureObjectSizeShape
ExportToSpreadsheet
removes small holes from the TissueMask
Select the input image
Inverted_TissueMask
(from ImageMath #13)
Name the output image
Inverted_TissueMask_deholed
Size of holes to fill
20.0
Output Settings
View Workspace
Adjust modules:
Start Test Mode
Analyze Images
Found 5 rows

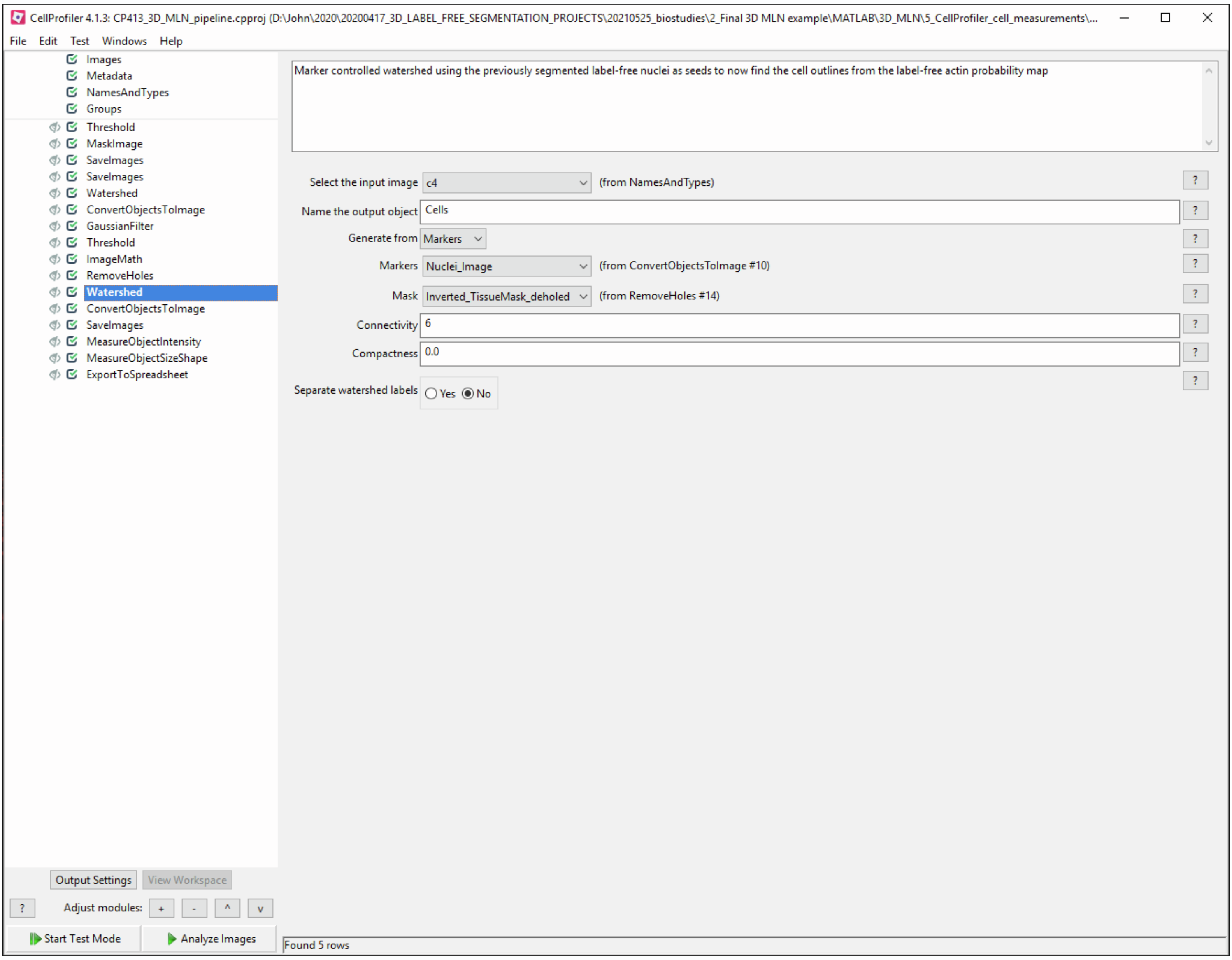
CellProfiler 4.1.3: CP413_3D_MLN_pipeline.cpproj (D:\John\2020\20200417_3D_LABEL_FREE_SEGMENTATION_PROJECTS\20210525_biostudies\2_Final 3D MLN example\MATLAB\3D_MLN\5_CellProfiler_cell_measurements\...
File Edit Test Windows Help
Images
Metadata
NamesAndTypes
Groups
Threshold
MaskImage
SaveImages
SaveImages
Watershed
ConvertObjectsToImage
GaussianFilter
Threshold
ImageMath
RemoveHoles
Watershed
ConvertObjectsToImage
SaveImages
MeasureObjectIntensity
MeasureObjectSizeShape
ExportToSpreadsheet
Marker controlled watershed using the previously segmented label-free nuclei as seeds to now find the cell outlines from the label-free actin probability map
Select the input image c4 (from NamesAndTypes)
Name the output object Cells
Generate from Markers
Markers Nuclei_Image (from ConvertObjectsToImage #10)
Mask Inverted_TissueMask_deholed (from RemoveHoles #14)
Connectivity 6
Compactness 0.0
Separate watershed labels Yes No
Output Settings
View Workspace
Adjust modules: + - ^ v
Start Test Mode
Analyze Images
Found 5 rows

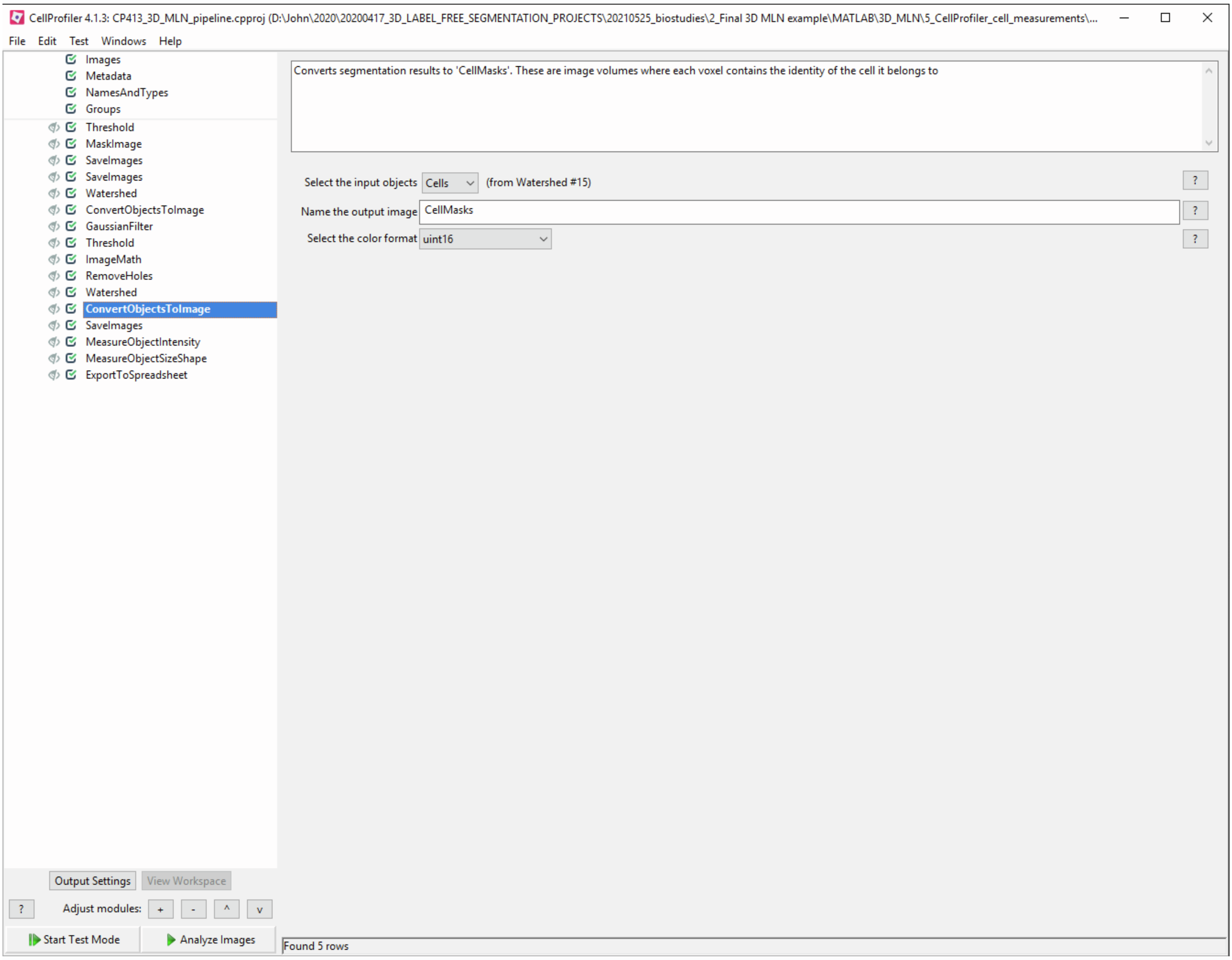

CellProfiler 4.1.3: CP413_3D_MLN_pipeline.cpproj (D:\John\2020\20200417_3D_LABEL_FREE_SEGMENTATION_PROJECTS\20210525_biostudies\2_Final 3D MLN example\MATLAB\3D_MLN\5_CellProfiler_cell_measurements\...
File Edit Test Windows Help
Images
Metadata
NamesAndTypes
Groups
Threshold
MaskImage
SaveImages
SaveImages
Watershed
ConvertObjectsToImage
GaussianFilter
Threshold
ImageMath
RemoveHoles
Watershed
ConvertObjectsToImage
SaveImages
MeasureObjectIntensity
MeasureObjectSizeShape
ExportToSpreadsheet
Converts segmentation results to 'CellMasks'. These are image volumes where each voxel contains the identity of the cell it belongs to
Select the input objects Cells (from Watershed #15)
Name the output image CellMasks
Select the color format uint16
Output Settings
View Workspace
Adjust modules:
Start Test Mode
Analyze Images
Found 5 rows

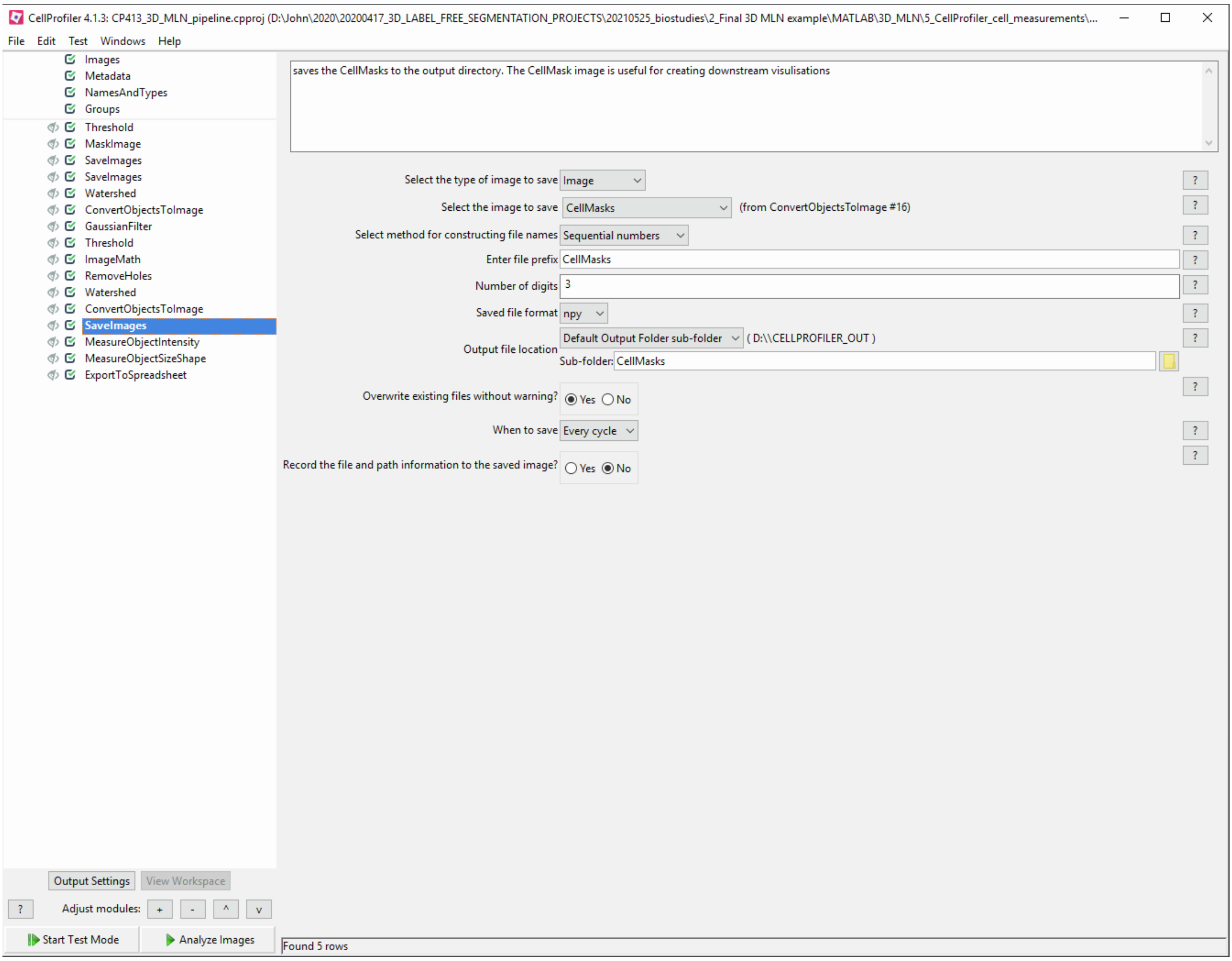
CellProfiler 4.1.3: CP413_3D_MLN_pipeline.cpproj (D:\John\2020\20200417_3D_LABEL_FREE_SEGMENTATION_PROJECTS\20210525_biostudies\2_Final 3D MLN example\MATLAB\3D_MLN\5_CellProfiler_cell_measurements\...
File
Edit
Test
Windows
Help
Images
Metadata
NamesAndTypes
Groups
Threshold
MaskImage
SaveImages
SaveImages
Watershed
ConvertObjectsToImage
GaussianFilter
Threshold
ImageMath
RemoveHoles
Watershed
ConvertObjectsToImage
SaveImages
MeasureObjectIntensity
MeasureObjectSizeShape
ExportToSpreadsheet
saves the CellMasks to the output directory. The CellMask image is useful for creating downstream visulisations
Select the type of image to save
Image
Select the image to save
CellMasks
(from ConvertObjectsToImage #16)
Select method for constructing file names
Sequential numbers
Enter file prefix
CellMasks
Number of digits
3
Saved file format
npy
Output file location
Default Output Folder sub-folder
(D:\\CELLPROFILER_OUT)
Sub-folder:
CellMasks
Overwrite existing files without warning?
Yes
No
When to save
Every cycle
Record the file and path information to the saved image?
Yes
No
Output Settings
View Workspace
Adjust modules:
Start Test Mode
Analyze Images
Found 5 rows

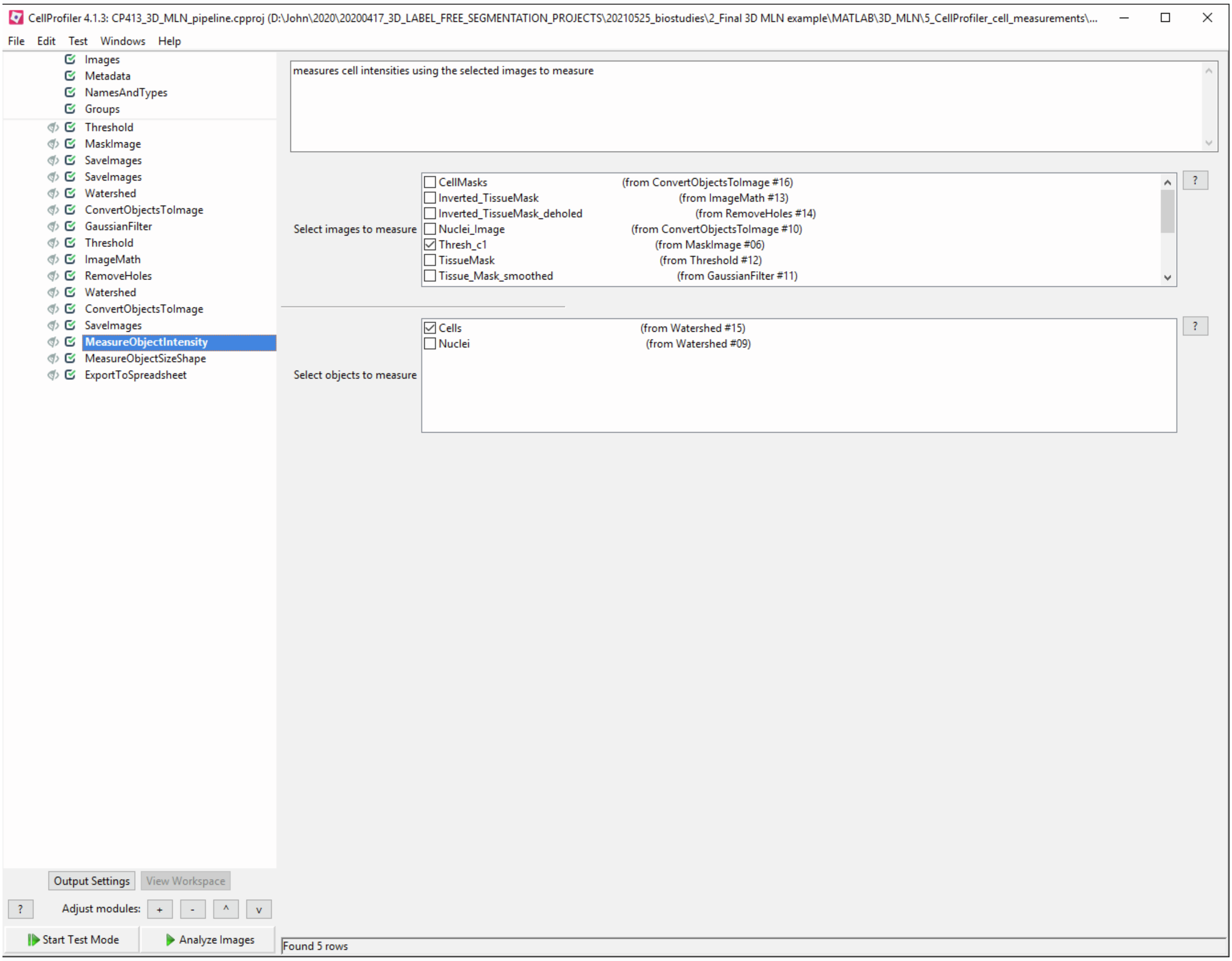
CellProfiler 4.1.3: CP413_3D_MLN_pipeline.cpproj (D:\John\2020\20200417_3D_LABEL_FREE_SEGMENTATION_PROJECTS\20210525_biostudies\2_Final 3D MLN example\MATLAB\3D_MLN\5_CellProfiler_cell_measurements\...
File Edit Test Windows Help
Images
Metadata
NamesAndTypes
Groups
Threshold
MaskImage
SaveImages
SaveImages
Watershed
ConvertObjectsToImage
GaussianFilter
Threshold
ImageMath
RemoveHoles
Watershed
ConvertObjectsToImage
SaveImages
MeasureObjectIntensity
MeasureObjectSizeShape
ExportToSpreadsheet
measures cell intensities using the selected images to measure
Select images to measure
CellMasks (from ConvertObjectsToImage #16)
Inverted_TissueMask (from ImageMath #13)
Inverted_TissueMask_deholed (from RemoveHoles #14)
Nuclei_Image (from ConvertObjectsToImage #10)
Thresh_c1 (from MaskImage #06)
TissueMask (from Threshold #12)
Tissue_Mask_smoothed (from GaussianFilter #11)
Select objects to measure
Cells (from Watershed #15)
Nuclei (from Watershed #09)
Output Settings
View Workspace
Adjust modules:
Start Test Mode
Analyze Images
Found 5 rows

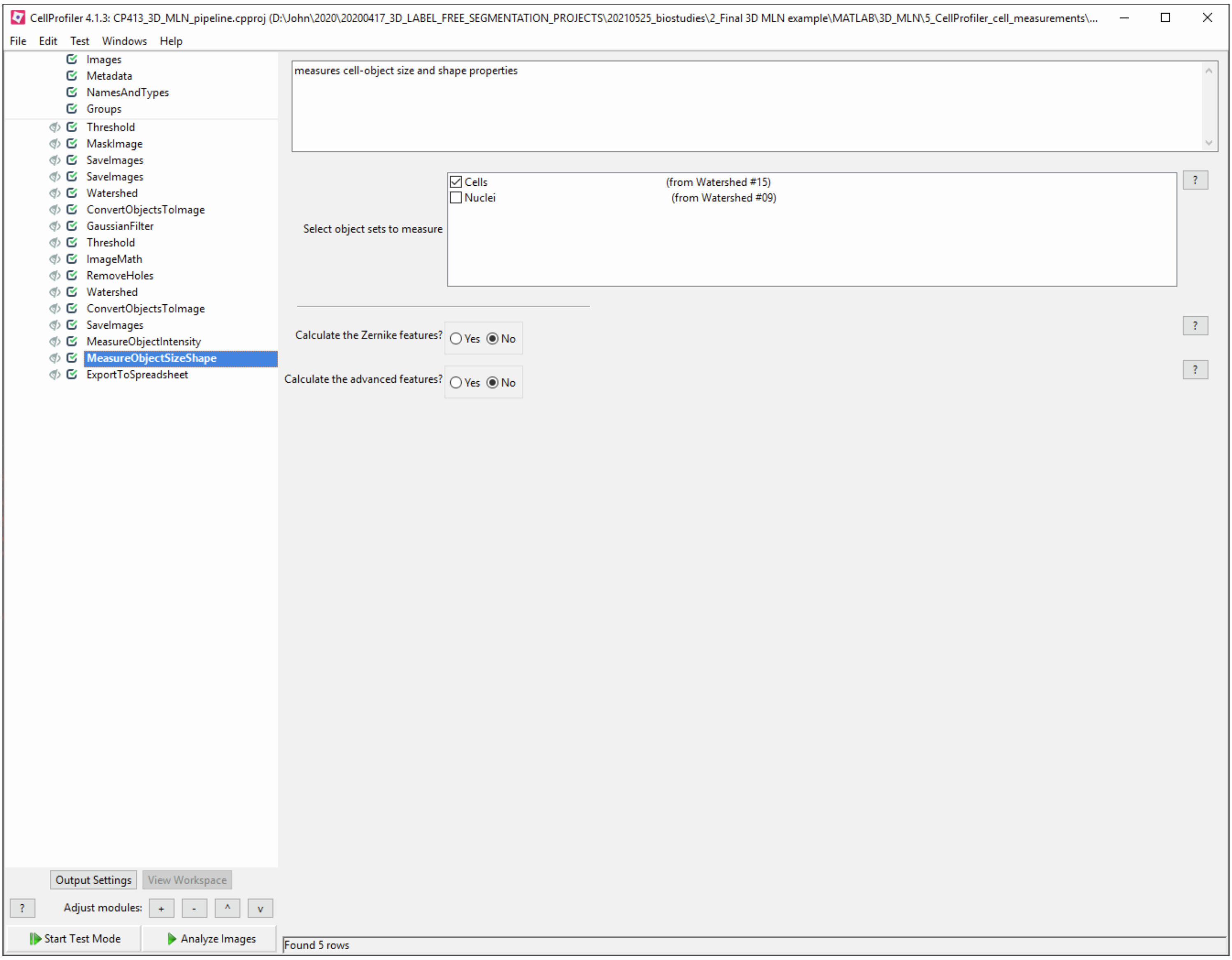
CellProfiler 4.1.3: CP413_3D_MLN_pipeline.cpproj (D:\John\2020\20200417_3D_LABEL_FREE_SEGMENTATION_PROJECTS\20210525_biostudies\2_Final 3D MLN example\MATLAB\3D_MLN\5_CellProfiler_cell_measurements\...
File Edit Test Windows Help
Images
Metadata
NamesAndTypes
Groups
Threshold
MaskImage
SaveImages
SaveImages
Watershed
ConvertObjectsToImage
GaussianFilter
Threshold
ImageMath
RemoveHoles
Watershed
ConvertObjectsToImage
SaveImages
MeasureObjectIntensity
MeasureObjectSizeShape
ExportToSpreadsheet
measures cell-object size and shape properties
Select object sets to measure
Cells (from Watershed #15)
Nuclei (from Watershed #09)
Calculate the Zernike features? Yes No
Calculate the advanced features? Yes No
Output Settings
View Workspace
Adjust modules: + - ^ v
Start Test Mode
Analyze Images
Found 5 rows

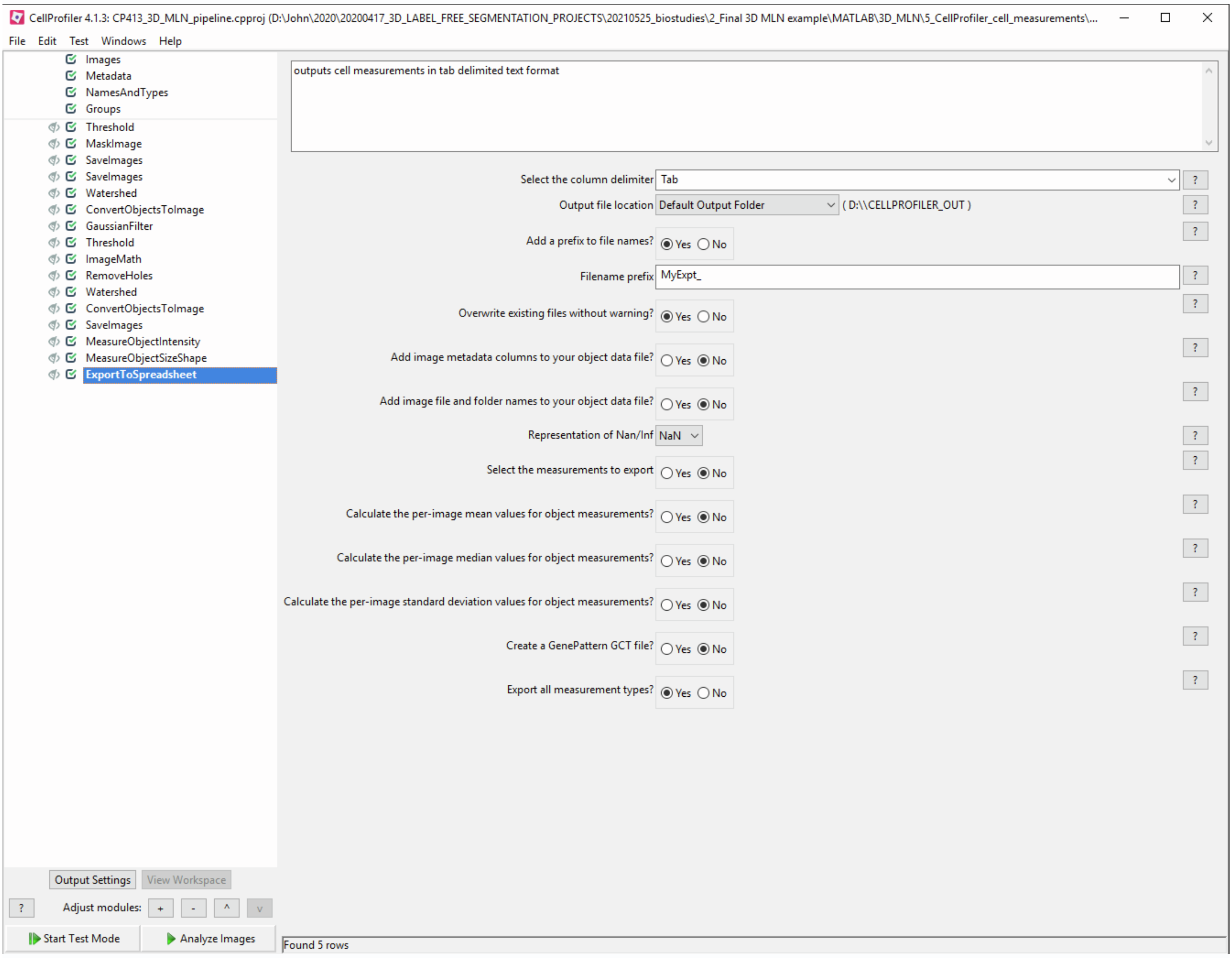
CellProfiler 4.1.3: CP413_3D_MLN_pipeline.cpproj (D:\John\2020\20200417_3D_LABEL_FREE_SEGMENTATION_PROJECTS\20210525_biostudies\2_Final 3D MLN example\MATLAB\3D_MLN\5_CellProfiler_cell_measurements\...
File Edit Test Windows Help
Images
Metadata
NamesAndTypes
Groups
Threshold
MaskImage
SaveImages
SaveImages
Watershed
ConvertObjectsToImage
GaussianFilter
Threshold
ImageMath
RemoveHoles
Watershed
ConvertObjectsToImage
SaveImages
MeasureObjectIntensity
MeasureObjectSizeShape
ExportToSpreadsheet
outputs cell measurements in tab delimited text format
Select the column delimiter Tab
Output file location Default Output Folder (D:\\CELLPROFILER_OUT)
Add a prefix to file names? Yes No
Filename prefix MyExpt_
Overwrite existing files without warning? Yes No
Add image metadata columns to your object data file? Yes No
Add image file and folder names to your object data file? Yes No
Representation of Nan/Inf NaN
Select the measurements to export Yes No
Calculate the per-image mean values for object measurements? Yes No
Calculate the per-image median values for object measurements? Yes No
Calculate the per-image standard deviation values for object measurements? Yes No
Create a GenePattern GCT file? Yes No
Export all measurement types? Yes No
Output Settings
View Workspace
Adjust modules:
Start Test Mode
Analyze Images
Found 5 rows
